# In-depth proteomic profiling of left ventricular tissues in human end-stage dilated cardiomyopathy

**DOI:** 10.18632/oncotarget.15689

**Published:** 2017-02-25

**Authors:** Shanshan Liu, Yan Xia, Xiaohui Liu, Yi Wang, Zhangwei Chen, Juanjuan Xie, Juying Qian, Huali Shen, Pengyuan Yang

**Affiliations:** ^1^ Institutes of Biomedical Sciences of Shanghai Medical School and Minhang Hospital, Fudan University, Shanghai, China; ^2^ Department of Systems Biology for Medicine and School of Basic Medical Sciences, Fudan University, Shanghai, China; ^3^ Department of chemistry, Fudan University, Shanghai, China; ^4^ Department of Cardiology, Shanghai Institute of Cardiovascular Diseases, Zhongshan Hospital, Fudan University, Shanghai, China

**Keywords:** dilated cardiomyopathy, left ventricle, cell death, S100A1, eEF2

## Abstract

Dilated cardiomyopathy (DCM) is caused by reduced left ventricular (LV) myocardial function, which is one of the most common causes of heart failure (HF). We performed iTRAQ-coupled 2D-LC-MS/MS to profile the cardiac proteome of LV tissues from healthy controls and patients with end-stage DCM. We identified 4263 proteins, of which 125 were differentially expressed in DCM tissues compared to LV controls. The majority of these were membrane proteins related to cellular junctions and neuronal metabolism. In addition, these proteins were involved in membrane organization, mitochondrial organization, translation, protein transport, and cell death process. Four key proteins involved in the cell death process were also detected by western blotting, indicated that cell death was activated in DCM tissues. Furthermore, S100A1 and eEF2 were enriched in the “cellular assembly and organization” and “cell cycle” networks, respectively. We verified decreases in these two proteins in end-stage DCM LV samples through multiple reaction monitoring (MRM). These observations demonstrate that our understanding of the mechanisms underlying DCM can be deepened through comparison of the proteomes of normal LV tissues with that from end-stage DCM in humans.

## INTRODUCTION

Dilated cardiomyopathy (DCM) is a type of chronic cardiovascular disease characterized by a dilation of the left or both ventricles [1]. DCM remains asymptomatic at an early stage and deteriorates gradually into heart failure (HF) without timely and effective treatment [2]. Severe cases require one or more surgical procedures, which causes an enormous burden to families and society. The progression from DCM to HF involves multiple causes and complex mechanisms [3, 4]. Remarkable progress has been made in understanding the genetic basis of DCM. Nevertheless, the set of studied genes related to DCM (~30) and their associated variants account for less than half of the genomic cause of DCM [5].

Most previous studies on molecular changes related to DCM focused on the transcriptome [6, 7]. Comparative proteomic analysis is a powerful diagnostic tool to determine the onset, progression, and prognosis of human diseases [8]. 2-D gel proteomics analysis was also used to reveal alterations in DCM [9]; however, the data scale was not large enough. The recent development of liquid chromatography coupled with tandem mass spectrometry (LC-MS/MS) technology in high-resolution platforms allowed for better detection of proteins expressed in low abundance [10, 11]. MS-based quantitative proteomics approaches, such as iTRAQ [12], SILAC (stable isotope labeling with amino acids in cell cultures [13], MRM [14] and label-free techniques, have become increasingly useful to cardiovascular disease research [15–18]. At present, there are only a handful used biomarkers to guide clinical decision for DCM such as brain natriuretic peptide (BNP) [19, 20] and ST2 [21, 22], which can be used to predict risk of decompensation. Finding effective biomarkers and elucidating the pathogenic mechanism underlying DCM could improve the prognosis of patients. Recently, many studies have reported a large-scale cardiac proteome from whole heart [23], left ventricle (LV) [24, 25] and fetal heart [26], which revealed heart-specific proteins and differences between the human cardiac chambers (atrium-enriched intracellular transport and ventricle-enriched muscle contraction). In addition, only a preliminary study on proteomic and transcriptomic alterations in DCM compared to normal LV was reported, which found 16 molecules changed conformably by proteomic and transcriptomic analysis [27].

Here, we used iTRAQ-coupled 2D LC-MS/MS to conduct an in-depth quantitative profiling of the cardiac proteome of left ventricular tissues from normal and end-stage dilated cardiomyopathy hearts. Understanding the proteome will further our knowledge of the function of the cardiovascular system, thereby revealing new potential diagnostic biomarkers or therapeutic targets to treat DCM.

## RESULTS

### Analysis of left ventricular proteomic profiling from normal human and end-stage DCM patients

**Figure 1:**
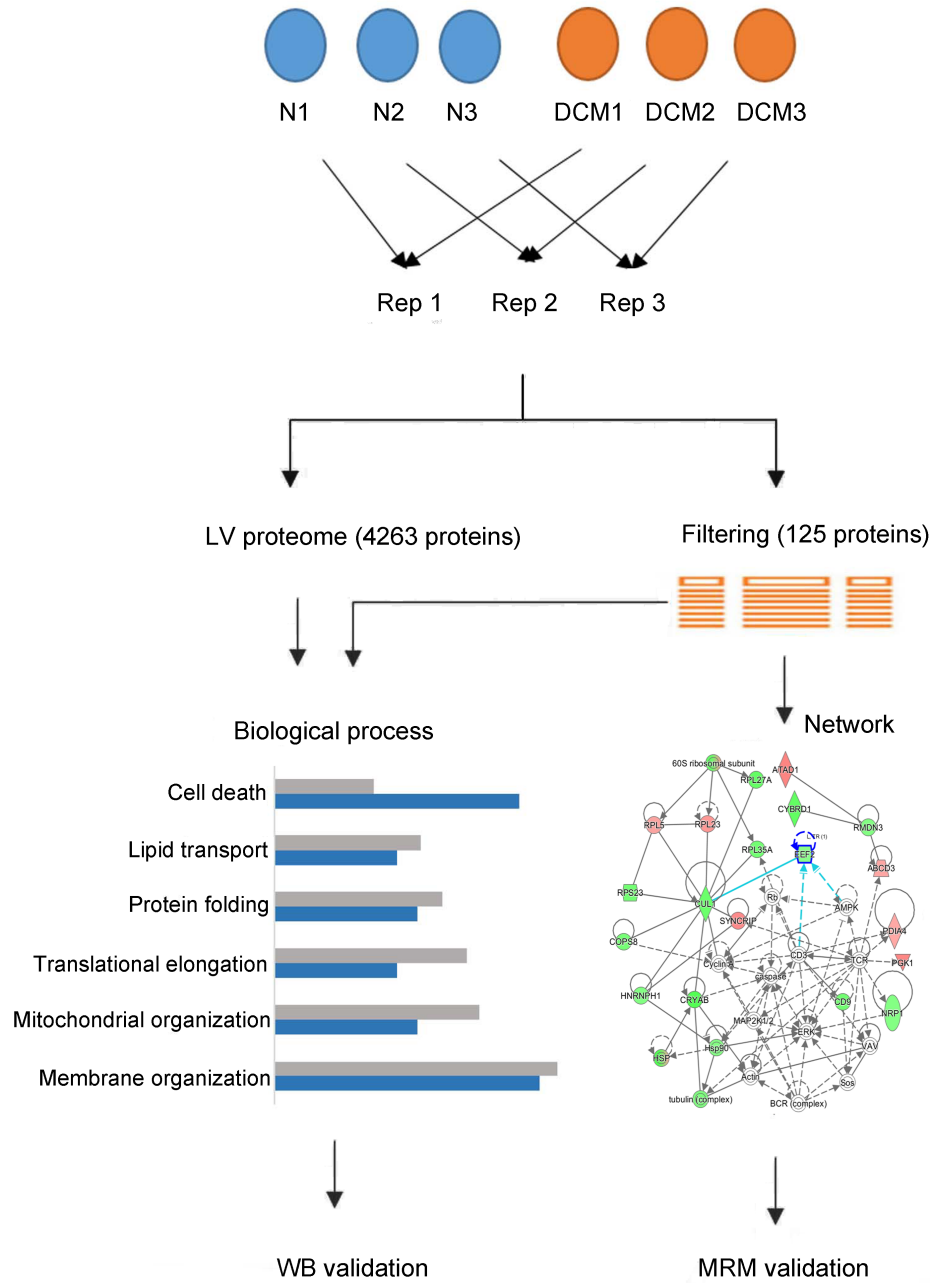
Schematic of cardiac proteomic analysis of the left ventricular tissues from normal and end-stage dilated cardiomyopathy hearts

To investigate the cardiac protein alterations in end-stage DCM patients, we used quantitative proteomic analysis on normal and end-stage DCM human LVs (three samples per group), applying the iTRAQ-coupled 2D LC-MS/MS method. The workflow of this study was presented in Figure 1. In total, 4263 proteins were identified and 4099 proteins were successfully quantified in the normal and end-stage DCM LVs, of which 125 proteins showed significant differences (Supplementary Table S1 & Supplementary Table S2). The identified proteins, especially those with differential expression in end-stage DCM versus normal LVs, were imported to ExPasy, DAVID and IPA software to analyze their physicochemical properties and biological characteristics. Proteins known to participate in major biological functions were further analyzed by western blot and MRM.

**Figure 2:**
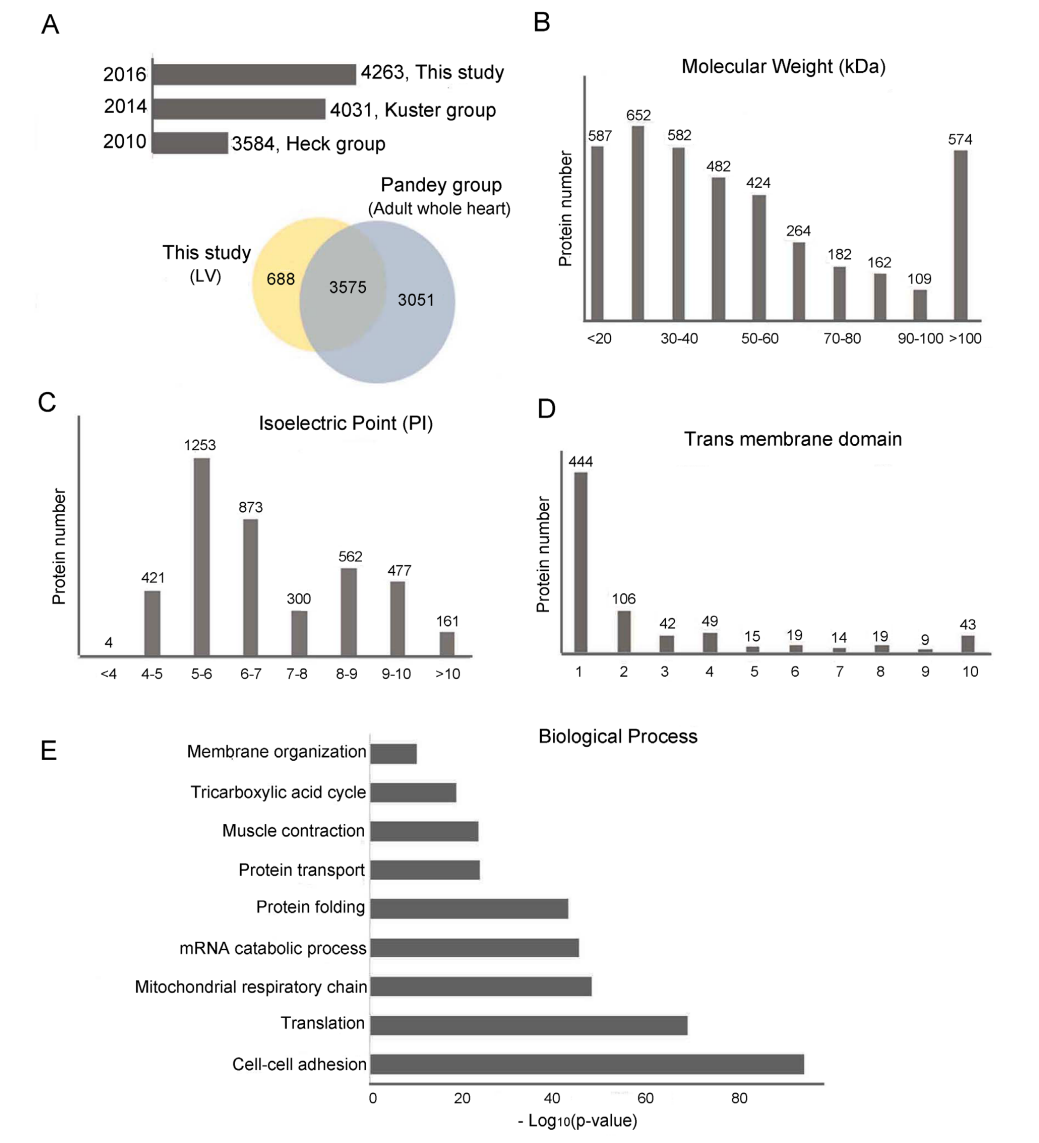
Physico-chemical and biological function analyses of LV proteome **A**. Histogram plot and Venn diagram comparing the cardiac proteome of LV from this study with those from Heck *et al*., Kuster et *al*., and Pandey *et al*. **B**. Molecular weight (MW, in kDa) distribution of LV proteome. **C**. Isoelectric focusing point (pI) distributions of LV proteome. Molecular weight and isoelectric point were calculated using the online ProtParam tool available through ExPASY. **D**. Transmembrane domains were predicted using TMHMM 2.0. **E**. Biological processes of LV proteome.

Figure 2A shows a comparison between our data and several other heart proteome datasets. The histogram plot shows the comparison between our data and human LV proteome data from Heck's work and Kuster's work, which identified 3584 proteins and 4031 proteins, respectively [24, 25] in healthy human LV. To date, our LV proteomic profiling is one of the largest datasets for human LV. The Venn diagram shows the comparison between our data and Pandey's Human proteome map (available as an interactive web-based resource at http://www.humanproteomemap.org), which identified 6626 proteins from adult whole heart tissue [23]. Our dataset, which focused only on LV, identified 688 extra proteins compared with Pandey's in-depth heart proteomic profiling. To further investigate human LV proteins, we analyzed their physicochemical properties and biological processes. We found that 45.3% of the identified proteins had a molecular weight (MW)<=40 kDa (Figure 2B), 52.5% were weakly acidic (pI 5.0-7.0) (Figure 2C), and 17.8% had at least one transmembrane domain (58.4% of which had a single trans membrane domain) (Figure 2D). These LV proteins can be grossly classified in three categories: energy production (mitochondrial organization and tricarboxylic acid cycle), cardiac contraction (cellular adhesion, protein transport and muscle contraction), and protein synthesis (protein translation, mRNA catabolic process, and protein folding). Figure 2E listed the most representative biological processes of the LV proteins we identified.

### Gene ontology enrichment analysis of changed cardiac proteins in end-stage DCM

**Figure 3:**
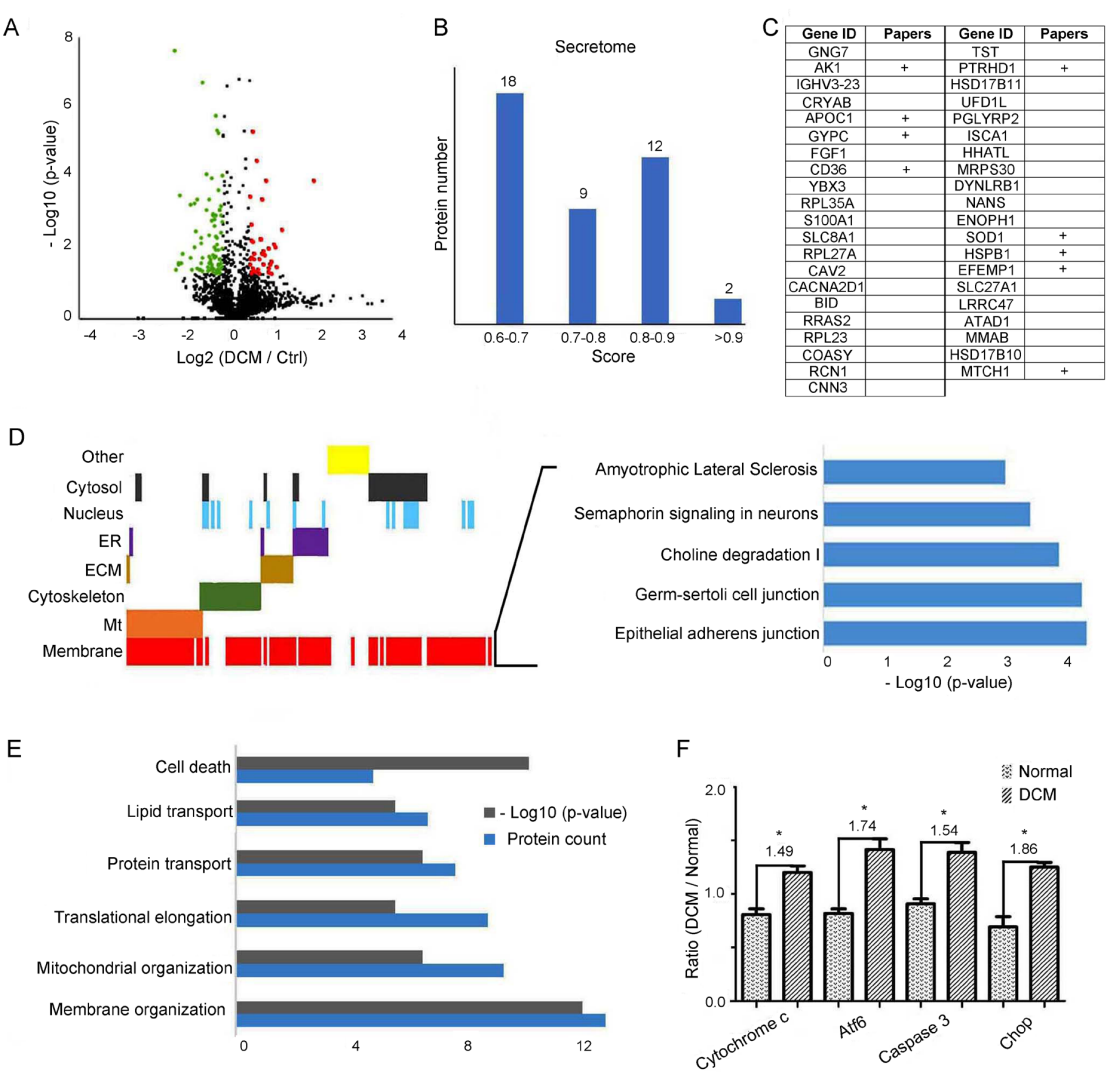
GO analysis of proteins differentially expressed in end-stage DCM LV compared to normal LV **A**. Volcano of differentially expressed proteins. The horizontal coordinates represent the proteins with a log_2_^ratio (DCM/Control)^. The vertical axis is the -log_10_^(p-value)^ (p-value <0.05, ratio >1.20 shown in red and ratio<0.83 shown in green). Each point represents an individual protein. **B**. Prediction of potentially secreted by SecretomeP 2.0 from 125 differentially expressed proteins. The recommended threshold is 0.6 for mammalian sequences. **C**. The research status of 41 predicted secreted proteins in plasma/serum. “+” indicates that the protein has been studied in plasma/serum by literature retrieval. **D**. Enriched cellular compartments of differentially expressed proteins in end-stage DCM and top five enriched canonical pathways of membrane-associated proteins. **E**. Enriched biological processes of differentially expressed proteins in end-stage DCM. The grey column represents the number of proteins in corresponding biological process, and the blue column represents the -log_10_^(p-value)^. **F**. Western blot validation of four key proteins, Atf6, Caspase 3, Cytochrome c, and Chop in cell death process. “*” indicates that protein alteration was significant (p-value<0.05).

Proteins showing over 1.2-fold in increase or at least 0.83-fold in decrease, with p-value<0.05 were considered as differentially expressed. In total, 40 upregulated and 85 downregulated proteins were screened out for further analyses (Figure 3A). Since proteins secreted by cells in response to various stimuli are the most likely to be found in blood/plasma, it is a promising and practical approach to look for potential biomarkers in secretomes. We used SecretomeP to predict potential secreted proteins from 125 differentially the expressed proteins. We found 41 potential secreted proteins, with ten of them being previously reported as potential plasma/serum biomarkers for different diseases (Figure 3B-3C). Detailed protein descriptions and relevant literature are summarized in Supplementary Table S3. We performed gene ontology analysis on these differentially expressed proteins to elucidate their biological functions in end-stage DCM. Of all altered proteins, 20.8% functioned in mitochondria (Mt), 16.8% were related to the cytoskeleton, 21.6% were cytoplasmic proteins, 9.6% functioned in the extracellular matrix (ECM), 11.2% in the endoplasmic reticulum (ER), and 14.4% in the nucleus. Notably, we found that 77.6% of all these changed proteins are membrane proteins (Figure 3D). We then used IPA to investigate the top five enriched canonical pathways in which these differentially expressed proteins participate, including the Amyotrophic Lateral Sclerosis pathway, semaphoring signaling in neurons, Choline degradation I, Germ-sertoli cell-cell junction, and Epithelial adherens junction (Figure 3D). Our results show that most differentially expressed proteins in end-stage DCM versus normal LVs contributed to membrane organization. Furthermore, mitochondrial organization, translational elongation and protein folding processes were also prominent in DCM, followed by lipid transport and cell death processes (Figure 3E). Enrichment of changed proteins in cell death processes indicated that the DCM samples might have developed to the end point of heart failure. We used western blot to measure the levels of four proteins relevant to cell death in every LV sample, namely Cytochrome c, Atf6, Caspase 3, and Chop. The targeted protein bands of the four proteins in LV are shown in Supplementary Figure S1. We found that these four proteins were upregulated in LV, suggesting the activation of cell death processes in our DCM samples (Figure 3F).

### Network analysis of changed proteins in end-stage DCM and the verification of S100A1 and eEF2 expression

**Figure 4:**
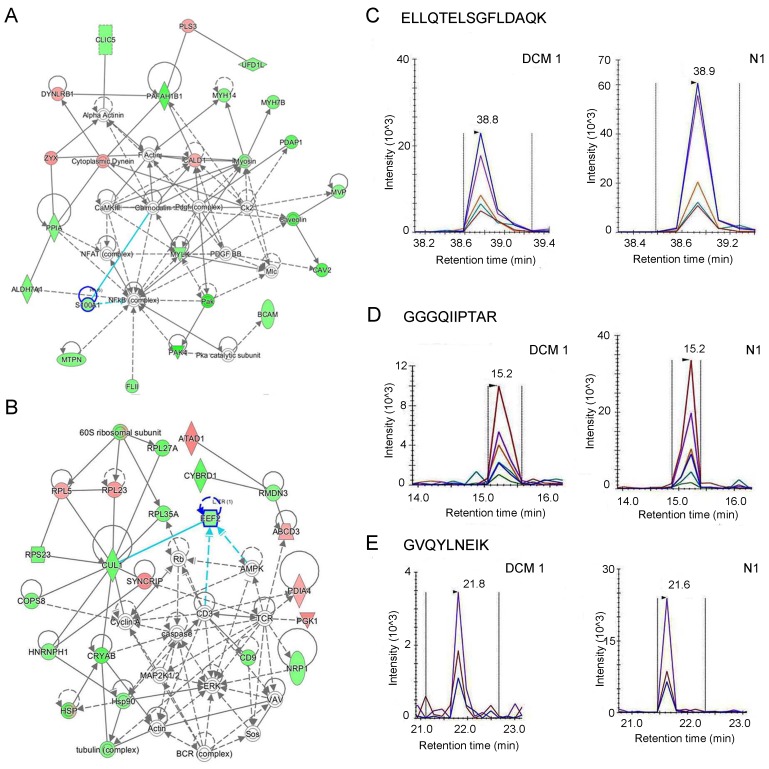
Verification of S100A1 and eEF2 focused on cellular assembly and organization network and cell cycle network, respectively **A**. Enriched cellular assembly and organization network. Green represents downregulated proteins while red indicates upregulated ones. Color intensity correlates with fold change. Straight and dashed lines represent direct or indirect gene-to-gene interactions, respectively. **B**. Enriched cell cycle network. **C**. Skyline display of the ion chromatograms for a S100A1 peptide ELLQTELSGFLDAQK. **D**.-**E**. Skyline display of the ion chromatograms of two unique peptides, GGGQIIPTAR and GVQYLNEIK, from eEF.

To evaluate the potential associations among the 125 differentially expressed proteins, we analyzed their possible protein-protein interactions using literature-curated databases. Then, the differentially expressed proteins were mainly focused on five networks (Supplementary Figure S2). The five networks can be divided into four categories: cell death and DNA replication, cellular assembly and organization, cell cycle, and lipid metabolism. We paid more attention to the two networks with the highest scores, namely the “cellular assembly and organization” and the “cell cycle” networks (Figure 4A-4B). Twenty differential proteins participated in the cellular assembly and organization network. Four proteins displayed increased expressional trend, and sixteen proteins exhibited decreased expression in end-stage DCM. Nineteen differential proteins were involved in the cell cycle network. Four proteins of which were increased, and fifteen proteins were decreased. Literature retrieval to explore the relationships of these proteins in the two networks with major cardiovascular diseases are shown in Supplementary Table S4, revealing that nine of the twenty proteins in the “cellular assembly and organization” network and six of the nineteen proteins in the “cell cycle” network have been reported to be associated with Myocardial Infarction (MI), Ischemia Reperfusion (I/R), Atherosclerosis (As), Coronary Syndrome (CS) or DCM. Two down-regulated proteins in these networks, S100A1 (S100 calcium binding protein A1) and eEF2 (eukaryotic Elongation Factor 2), were validated with the MRM method. The unique peptide ELLQTELSGFLDAQK from S100A1 was screened out by following criteria established by the SRMAtlas platform (without modification, without missed cleavage, without share with others). Figure 4C & Supplementary Figure S3 show the Skyline-extracted ion chromatograms for the peptide on six samples with its transition peaks (y8/865.4414, y7/778.4094, y5/574.3195, y4/461.2354). The two unique peptides GGGQIIPTAR and GVQYLNEIK from eEF2 were found using the same criteria. Figure 4D-4E & Supplementary Figure S4, show the Skyline-extracted ion chromatograms for the two peptide on six samples with their transitions, (y6/670.4246, y5/557.3406, y4/444.2565, b4/300.1302, b5/413.2143, b6/526.298) and (y7/907.4884, y6/779.4298, y4/503.2824), respectively. A summary of Skyline quantitation data for these product ions from the three peptides is shown in Supplementary Table S5. The average ratio (DCM/Normal) of the peptide from S100A1 is 0.85 with a 21.45% coefficient of variation (CV). The average ratios (DCM/Normal) of the two peptides from eEF2 are 0.62 (CV=6.27%) for GGGQIIPTAR and 0.41 (CV=31.22%) for GVQYLNEIK. Thus, in the end-stage DCM samples, S100A1 and eEF2 were downregulated according to MRM results, which are consistent with the iTRAQ quantification results.

## DISCUSSION

Heart has four different chambers, which are responsible for different functions. Reduced systolic and diastolic ability of LV is one of the major causes for DCM, yet the pathogenic mechanisms of DCM are not fully understood. Thus, we investigated alterations in left ventricular protein expression in end-stage DCM using iTRAQ-coupled 2D LC-MS/MS method. We identified a total of 4263 proteins in LV and found 125 proteins that were differentially expressed in end-stage DCM versus normal LVs. So far, our dataset is one of the largest datasets for human LV so far. Gene ontology analysis of the LV proteome highlighted the characteristic functions of heart tissue, such as muscle contraction, cell adhesion, and mitochondrial organization, etc. Cardiac contraction to deliver blood throughout the body is the core function of heart tissue. Initially, pacemaker cells in the sinoatrial node of the heart generate an action potential. Then, transmission of contractile force from one cell to another is accomplished by gap junctions and a steady supply of ATP is required to sustain cardiac contraction. Researches have shown many mutations in proteins found in junctional structures and mitochondria, which resulted in increased deterioration of the heart's pump function [28, 29].

In addition, we found many proteins differentially expressed in end-stage DCM are membrane proteins (Figure 3D) through quantification of more than 4000 proteins. Unsurprisingly, DCM-associated proteins were enriched in membrane organization processes when compared with the whole LV proteome (Figure 2E & Figure 3E). Membrane proteins included ion channels and receptors, etc., which are critical for calcium storage and transmission of force among cardio-myocytes. These changed membrane proteins in end-stage DCM are involved in Amyotrophic lateral sclerosis, Semaphoring signaling in neurons and Choline degradation I. Amyotrophic lateral sclerosis can cause the death of neurons that control voluntary muscles, and is also associated with sudden cardiac death and stress-induced cardiomyopathy [30]. Semaphorins are a large family of secreted or membrane-associated glycoproteins and are usually cues to deflect axons from inappropriate regions, especially important in neural system development. Vital roles for semaphorin signaling in vascular patterning and cardiac morphogenesis have been demonstrated [31]. Choline is the precursor molecule for the neurotransmitter acetylcholine, which is involved in many functions including memory and muscle control. Choline deficiency in the clinical setting could affect the overall recovery of a patient with heart problems [32]. Previous studies have demonstrated that over-activation of the nervous system was associated with worsening heart failure and the development of both atrial and ventricular arrhythmias [33]. Thus, appropriate membrane protein-targeted mechanical exploration could be performed to modulate the cardiac nervous metabolism in DCM.

Cellular assembly and organization can provide a macromolecular infrastructure to integrate mechanical and electrical coupling within the heart, which mainly involves assembly of the cardiac disk and organization of adherens junctions [34]. Some core proteins in the CaMK II and Calmodulin networks are important for calcium signaling to maintain cardiac contraction [35, 36]. The other core proteins in the network- PDGF complex (Platelet Derived Growth Factor) and NFκB (Nuclear factor kappa B), were important factors that participate in cellular response to extracellular stimuli [37, 38] (Figure 4A). In our study, S100A1 was downregulated in end-stage DCM patients, which was verified by MRM. S100A1 was predominantly found in LV with the highest protein levels [39–40]. In 1996, Remppis *et al*. first demonstrated that diminished S100A1 protein levels were related to dysfunctional human myocardium in congestive heart failure [41]. Subsequently, the relationship between S100A1 and cardiovascular events was examined in several population-based studies. A variety of animal heart failure models provided further evidence for decreased cardiac S100A1 protein levels as a molecular signature of failing myocardium *in vivo* [42–45]. Uncontrolled S100A1 expression has been linked to HF in human dilated cardiomyopathies as well as in various HF animal models [46]. However, the role of S100A1 in DCM remains elusive. Our findings highlight S100A1's effects in human cardiomyocytes and the potential benefits of an S100A1-targeted mechanical exploration in DCM.

The cell cycle network is related to proliferation and apoptosis and ensures appropriate heart size and development. Decreased levels of cellular cycle proteins prevent the regeneration of cardiomyocytes and result in decreased contractile ability in DCM [47]. In our study, core proteins in the cell cycle network that were differentially expressed include caspases, ERK (Extracellular signal-regulated kinase), and TCR (T cell receptor) (Figure 4B). The modulation of ERK cascades can protect the heart from cell death to control the cellular growth and the development of pathological hypertrophy [48]. Caspases regulate many cellular processes from cell death to signal propagation [49]. T cell proliferation can be triggered by antigen-TCR ligation to regulate a series of immunological responses [50]. The 19 proteins we found to be differentially expressed in the cell cycle network have not been studied in the context of DCM. In our study, one of these proteins, eEF2, was related to multiple cardiovascular diseases (Supplementary Table S4). The relationships between the cell cycle and eEF2 have been examined in several population-based studies. Overexpression of eEF2 in gastrointestinal cancers impacted G2/M progression in the cell cycle [51]. Previous studies have also demonstrated that eEF2 is inhibited during myocardial ischemia [52]. Furthermore, the inhibition of cell growth could be impacted via the AMPK/eEF2 kinase/eEF2 signaling pathway and slow the progression of cardiac hypertrophy [53]. In our study, the eukaryotic elongation factor eEF2 was downregulated in end-stage DCM patients as measured by iTRAQ LC-MS/MS analysis, which was verified by MRM. The functions of eEF2 have been rarely studied in the context of cardiovascular diseases and deserve further investigation in DCM.

In summary, we performed an in-depth quantitative profiling of the cardiac proteome of left ventricular tissues from normal and end-stage dilated cardiomyopathy hearts using iTRAQ-coupled 2D LC-MS/MS. The analysis of our comprehensive proteomic data established a reference for understanding pathogenic mechanisms underlying DCM. The differentially expressed proteins we found may be valuable candidates for future biomarker discovery. Furthermore, we showed that S100A1 and eEF2 were downregulated in end-stage DCM. Future experiments should be performed to gain more insight into how these proteins impact DCM progression.

## MATERIALS AND METHODS

### Ethics statement and tissue collection

The study was conducted in accordance with the guidelines of the Declaration of Helsinki. All samples were obtained from Zhongshan hospital from November 2015 to January 2016, which included three end-stage DCM patients’ hearts suffering from heart transplantation. Clinical history, blood tests, and electrocardiography were available from all the patients (Table 1). Three donor hearts that could not be transplanted for technical reasons (either because of blood type or size incompatibility) were used as controls. All donors had normal LV function and no history of myocardial disease or active infection at the time. All samples were procured from identical myocardial loci and were immediately frozen in liquid nitrogen and stored at -80°C.

**Table 1:** Clinical and echocardiographic characteristics of end-stage DCM patients

Index	DCM 1	DCM 2	DCM 3	Average
Age (years)	47	42	49	46±3.60
Gender male (%)	1	1	0	66.70%
Prior hypertension (%)	0	0	0	0
Diabetes mellitus (%)	0	0	0	0
NYHA class	4	3	3	3.3±0.58
Hemoglobin (g/L)	117	109	107	111±5.29
Hematocrit (%)	35.1	31.9	30.6	32.5±2.32
Total cholesterol (mmol/L)	3.12	3.34	3.97	3.5±0.44
Duration of disease (months)	72	60	52	61.3±10.01
Ejection fraction (%)	20	40	36	32±10.58
Left ventricular end systolic diameter (mm)	71	54	56	60.3±9.29
Left ventricular end diastolic diameter (mm)	77	68	68	71.0±5.20
Left artial diameter (mm)	57	70	34	53.7±18.23
Pulmonary hypertension (mmHg)	39	75	33	49.0±22.72

### Protein extraction, in-solution digestion and pre-separation

Heart tissues were washed and cut in PBS, then were ground under liquid nitrogen. Five times as much lysis buffer (2% SDS, 20 mM HEPES, pH=8.0) and 2 μL Benzonase (Sigma Aldrich, USA) were added in the powdered tissues. Then, the suspension was ultra-sonicated for 3 min at 3 s on and 5 s off intervals, centrifuged at 14000×g for 1 h at 4 °C. The supernatant was collected and quantified. The proteins were reduced by 5 mM TCEP at 56 °C for 30 min and alkylated by 10 mM MMTS at room temperature for 30 min, precipitated using acetone and resuspended using 50 mM TEAB (triethylammoniun m bicarbonate). Lys-C (Wako, Japan) was added at a mass ratio of 1:50 (enzyme:protein) for 3 h at 37 °C. Then, trypsin (Promega, USA) was added to the sample at a mass ratio of 1:50 (enzyme:protein) for 12 h. The digested peptides were desalted using a Sep-Pak C18 (Waters, USA) and concentrated using a SpeedVac. The dried peptides were resuspended in 500 mM TEAB. After 2 h of iTRAQ labeling, the samples were mixed, dried, and desalted. At last, the mixture was fractionated by high pH reversed phase liquid chromatography (Waters, USA) and dried.

### Mass spectrometry analysis and database search

The dried peptides were dissolved in buffer A (0.1% FA, H_2_O). Separation was performed on a reverse C_18_ column (C_18_ 3 μm, 100Å 75 μm×25 cm) from Thermo, with elution gradient from 8% to 38% buffer B (0.1% FA, ACN) with a flow rate of 300 nL/min for 2 hours by Eksigent 1D plus. The peptides were eluted into Triple TOF5600 (AB Sciex, USA) operated in positive mode with an ion spray voltage at 2.3 kV. Survey scans were acquired from 350 to 1500 m/z while MS/MS scans were from 100 to 1250 m/z in high sensitivity mode. The 20 most intensive precursors were separately selected for fragmentation per cycle. For iTRAQ experiments, protein identification and quantification were performed with ProteinPilot 4.5 software. Trypsin digestion was selected. Carbamidomethyl of cysteine was specified as a fixed modification. Oxidations of methionine and acetyl of the protein N-terminus were specified as variable modifications. A decoy database search strategy was adopted to estimate the FDR < 1% for peptide and protein identification.

### Biological functions and pathway analysis

In this study, we considered only proteins that showed a 1.2-fold increase or 0.83-fold decrease in expression, with *p*-value <0.05 (calculated by Student's t-test) considered as statistically significant. Annotations of biological processes and components were based on the analysis using the DAVID software (http://www.david.niaid.nih.gov). We used ingenuity pathways analysis (IPA, http://www.ingenuity.com) and calculations/assignments for imported proteins based on the literature. IPA revealed interactive networks for a set of focus proteins and biological pathways. Scores of ≧2 had at least a 99% confidence of not being generated by random chance alone.

### LC-MRM-MS analysis

The analyses for all the experiments were performed on a Q-TRAP 6500 mass spectrometer (AB SCIEX, USA) equipped with a Eksigent nano LC system. MRM-MS was performed on individual samples including three normal and three DCM-associated LV. High-confidence unique peptides of the target proteins were determined from SRMAtlas database for MRMs. The equimolar calibration mixture containing 15 heavy isotope-labeled peptides (Thermo, USA) were added into each sample to normalize results for variation in retention times and peak intensities between runs. All peak area integration was performed by Skyline software version 3.0.

### Western blotting

Proteins were electrophoresed in 10% polyacrylamide gel and transferred to a polyvinylidene fluoride membrane (Millipore). Protein expression was detected by immunoblotting with antibody against Atf6 (Abcam, Cambridge, UK), Caspase 3, Cytochrome c and Chop (Cell Signaling Technology, USA). After three washes, the blot was incubated with horseradish peroxidase-conjugated rabbit secondary antibody immunoglobulin G (Cell Signaling Technology, USA). GAPDH (Kangchen Biotechnology) was used as the internal control. The antigen–antibody complexes were detected using Pierce ECL Western Blotting Substrate (Thermo Fisher Scientific, Rockford, IL, USA) and visualized densitometry was performed with LAS-300 Image software (FUJIFILM, Kanagawa, Japan).

## SUPPLEMENTARY MATERIALS FIGURES AND TABLES













## Abbreviations

DCM: Dilated cardiomyopathy
HF: Heart failure
iTRAQ: Isobaric tags for relative and absolute quantitation
SILAC: Stable isotope labeling with amino acids in cell cultures
MRM: Multiple reaction monitoring
LV: Left ventricle
RV: Right ventricle
LA: Left atrium
RA: Right atrium
WB: Western blotting
Mt: Mitochonrion
ER: Endoplasmic reticulum
ECM: Extracellular matrix
S100A1: S100 calcium binding protein A1
eEF2: Eukaryotic elongation factor 2
ERK: extracellular signal-regulated kinase
TCR: T cell receptor
NFκB: Nuclear factor kappa B
PDGF: Platelet Derived Growth Factor
