# Supplementary material for: In-depth proteomic profiling of left ventricular tissues in human end-stage dilated cardiomyopathy
Source: Oncotarget. 2017 Feb 25;8(29):48321–32. doi: 10.18632/oncotarget.15689 (PMC5564650; doi:10.18632/oncotarget.15689)
Supplement: Supplementary file 1 [file oncotarget-08-48321-s001.pdf]

In-depth proteomic profiling of left ventricular tissues in human end-stage dilated cardiomyopathy

Supplementary Material

A

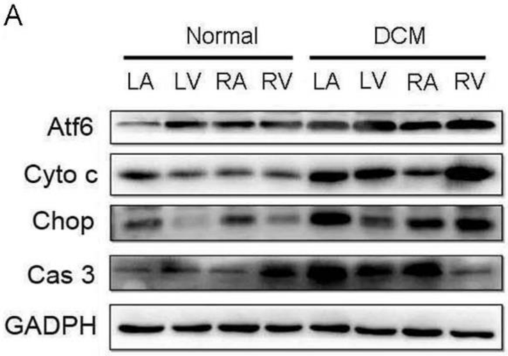

B

Chamber	Cytochrome c		Atf6		Caspase 3		Chop	
	Ratio	p	Ratio	p	Ratio	p	Ratio	p
LA	1.52	0.20	1.80	0.12	1.71	0.01	1.43	0.11
LV	1.49	0.00	1.74	0.02	1.54	0.02	1.86	0.01
RA	2.05	0.03	1.55	0.10	1.86	0.08	1.33	0.00
RV	1.82	0.02	1.72	0.27	0.79	0.14	1.12	0.73

Supplementary Figure S1: The targeted protein bands of the four proteins from end-stage DCM and normal LV. A, Immuno-blot probed with anti-Atf6, anti-chop, anti-caspase 3 and anti-cytochrome c antibody and equal loading was checked by probing the membrane with anti-GADPH antibody in in LV, RV, LA, and RA. B, The Ratio (DCM/control) and p (p-value) in three group of samples.

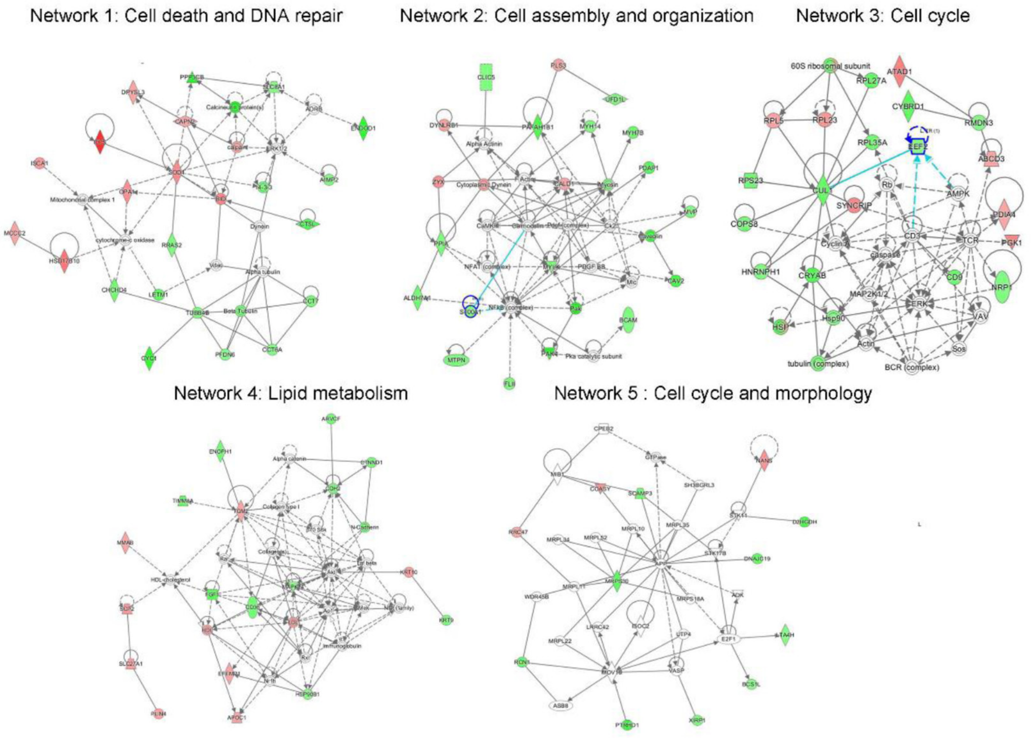

Supplementary Figure S2: Enriched networks of differentially expressed proteins by IPA.

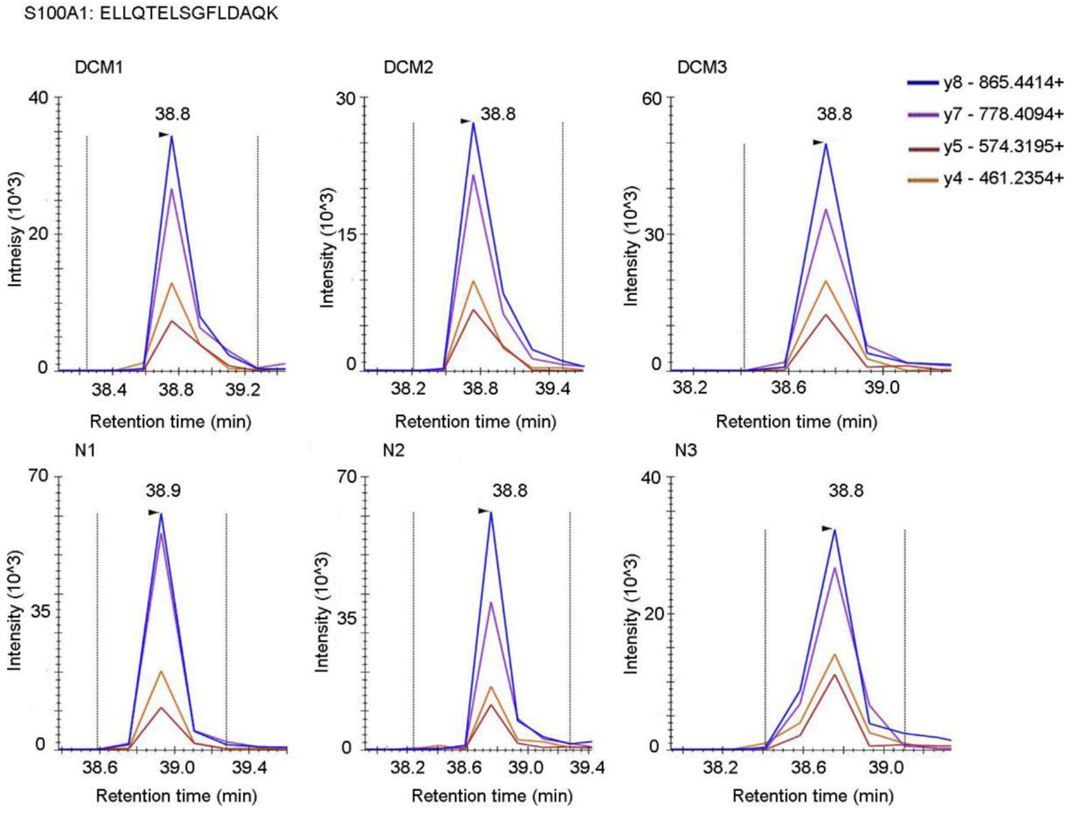

Supplementary Figure S3: Skyline extracted the ion chromatograms for a S100A1 peptide ELLQTELSGFLDAQK on six samples with their transitions.

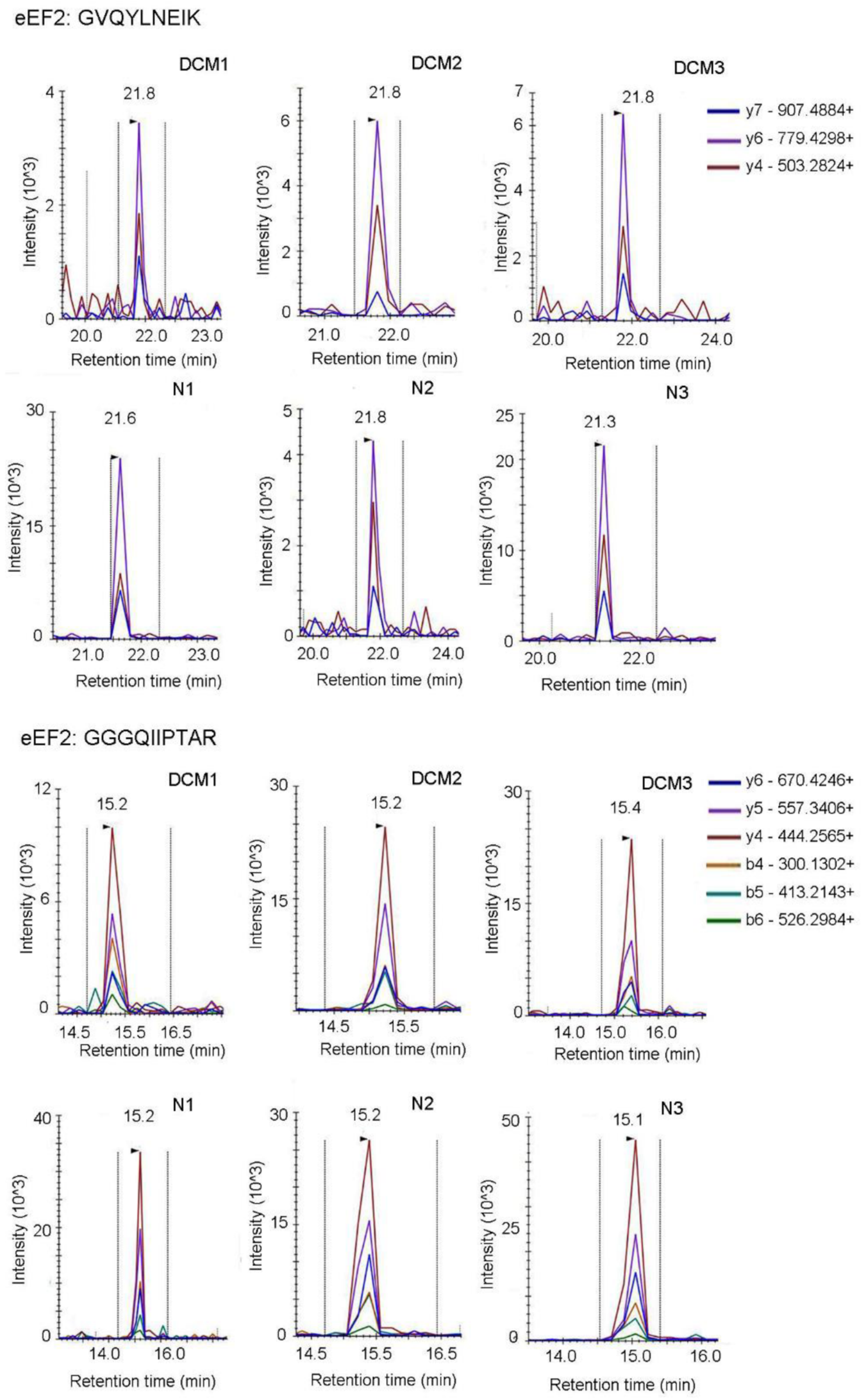

Supplementary Figure S4: Skyline extracted the ion chromatograms for two unique peptide GGGQIIPTAR and GVQYLNEIK from eEF2 on six samples with their transitions.
