# Supplementary material for: In-depth proteomic profiling of left ventricular tissues in human end-stage dilated cardiomyopathy
Source: Oncotarget. 2017 Feb 25;8(29):48321–32. doi: 10.18632/oncotarget.15689 (PMC5564650; doi:10.18632/oncotarget.15689)
Supplement: Supplementary file 2 [file oncotarget-08-48321-s002.docx]

**Table S1. All identified Left ventricular proteins in end-stage DCM versus normal control**

| **Number** | **ID** | **Description** | **Accession** | **Ratio 1** | **Ratio 2** | **Ratio 3** |
| --- | --- | --- | --- | --- | --- | --- |
| 1 | 1433B_HUMAN | 14-3-3 protein beta/alpha | P31946 | 1.12 | 0.90 | 0.73 |
| 2 | 1433E_HUMAN | 14-3-3 protein epsilon | P62258 | 0.93 | 0.67 | 0.99 |
| 3 | 1433F_HUMAN | 14-3-3 protein eta | Q04917 | 0.89 | 1.16 | 1.21 |
| 4 | 1433G_HUMAN | 14-3-3 protein gamma | P61981 | 0.60 | 0.68 | 0.91 |
| 5 | 1433T_HUMAN | 14-3-3 protein theta | P27348 | 1.77 | 1.15 | 0.87 |
| 6 | 1433Z_HUMAN | 14-3-3 protein zeta/delta | P63104 | 0.86 | 1.12 | 1.04 |
| 7 | 1A11_HUMAN | HLA class I histocompatibility antigen, A-11 alpha chain | P13746 |  |  | 1.01 |
| 8 | 1B14_HUMAN | HLA class I histocompatibility antigen, B-14 alpha chain | P30462 | 1.12 |  | 0.75 |
| 9 | 2A5A_HUMAN | Serine/threonine-protein phosphatase 2A 56 kDa regulatory subunit alpha isoform | Q15172 | 1.17 |  | 0.94 |
| 10 | 2A5D_HUMAN | Serine/threonine-protein phosphatase 2A 56 kDa regulatory subunit delta isoform | Q14738 | 1.02 |  | 1.03 |
| 11 | 2AAA_HUMAN | Serine/threonine-protein phosphatase 2A 65 kDa regulatory subunit A alpha isoform | P30153 | 0.87 | 1.12 | 1.08 |
| 12 | 2ABA_HUMAN | Serine/threonine-protein phosphatase 2A 55 kDa regulatory subunit B alpha isoform | P63151 | 0.66 | 1.22 | 1.00 |
| 13 | 3HIDH_HUMAN | 3-hydroxyisobutyrate dehydrogenase, mitochondrial | P31937 | 0.87 | 0.69 | 1.51 |
| 14 | 41_HUMAN | Protein 4.1 | P11171 |  | 1.07 |  |
| 15 | 4F2_HUMAN | 4F2 cell-surface antigen heavy chain | P08195 |  |  |  |
| 16 | 5NT1A_HUMAN | Cytosolic 5'-nucleotidase 1A | Q9BXI3 | 0.93 | 0.95 | 0.95 |
| 17 | 5NTD_HUMAN | 5'-nucleotidase | P21589 |  | 0.86 | 1.08 |
| 18 | 68MP_HUMAN | 6.8 kDa mitochondrial proteolipid | P56378 | 0.79 | 0.74 | 0.68 |
| 19 | 6PGD_HUMAN | 6-phosphogluconate dehydrogenase, decarboxylating | P52209 | 1.21 | 0.79 | 0.74 |
| 20 | 6PGL_HUMAN | 6-phosphogluconolactonase | O95336 | 1.24 | 2.27 | 1.42 |
| 21 | A1AG1_HUMAN | Alpha-1-acid glycoprotein 1 | P02763 | 0.54 | 1.17 | 1.07 |
| 22 | A1AG2_HUMAN | Alpha-1-acid glycoprotein 2 | P19652 |  |  | 0.80 |
| 23 | A1AT_HUMAN | Alpha-1-antitrypsin | P01009 |  | 0.94 | 0.78 |
| 24 | A1BG_HUMAN | Alpha-1B-glycoprotein | P04217 | 0.63 | 0.82 | 1.15 |
| 25 | A2AP_HUMAN | Alpha-2-antiplasmin | P08697 | 0.98 | 0.70 | 0.99 |
| 26 | A2GL_HUMAN | Leucine-rich alpha-2-glycoprotein | P02750 | 0.70 |  | 1.16 |
| 27 | A2MG_HUMAN | Alpha-2-macroglobulin | P01023 | 0.89 |  | 0.60 |
| 28 | AACT_HUMAN | Alpha-1-antichymotrypsin | P01011 | 2.27 | 0.82 | 0.93 |
| 29 | AAK1_HUMAN | AP2-associated protein kinase 1 | Q2M2I8 |  | 4.53 | 1.08 |
| 30 | AAKB2_HUMAN | 5'-AMP-activated protein kinase subunit beta-2 | O43741 | 0.95 |  | 0.87 |
| 31 | AAKG1_HUMAN | 5'-AMP-activated protein kinase subunit gamma-1 | P54619 | 0.14 |  | 1.52 |
| 32 | AAKG2_HUMAN | 5'-AMP-activated protein kinase subunit gamma-2 | Q9UGJ0 | 1.06 | 0.82 | 0.82 |
| 33 | AAMDC_HUMAN | Mth938 domain-containing protein | Q9H7C9 | 1.14 | 0.60 | 1.72 |
| 34 | AAPK1_HUMAN | 5'-AMP-activated protein kinase catalytic subunit alpha-1 | Q13131 |  | 2.86 | 1.13 |
| 35 | AAPK2_HUMAN | 5'-AMP-activated protein kinase catalytic subunit alpha-2 | P54646 | 2.65 | 0.53 | 0.88 |
| 36 | AASD1_HUMAN | Alanyl-tRNA editing protein Aarsd1 | Q9BTE6 |  | 1.00 | 0.32 |
| 37 | AASS_HUMAN | Alpha-aminoadipic semialdehyde synthase, mitochondrial | Q9UDR5 | 0.87 | 0.63 | 1.06 |
| 38 | AATC_HUMAN | Aspartate aminotransferase, cytoplasmic | P17174 | 0.61 |  | 1.13 |
| 39 | AATM_HUMAN | Aspartate aminotransferase, mitochondrial | P00505 |  |  | 0.65 |
| 40 | ABCA8_HUMAN | ATP-binding cassette sub-family A member 8 | O94911 | 0.81 | 2.21 | 1.04 |
| 41 | ABCB7_HUMAN | ATP-binding cassette sub-family B member 7, mitochondrial | O75027 | 1.46 | 0.40 | 0.83 |
| 42 | ABCB8_HUMAN | ATP-binding cassette sub-family B member 8, mitochondrial | Q9NUT2 | 0.77 | 1.34 | 0.99 |
| 43 | ABCC9_HUMAN | ATP-binding cassette sub-family C member 9 | O60706 |  | 1.54 | 1.28 |
| 44 | ABCD3_HUMAN | ATP-binding cassette sub-family D member 3 | P28288 | 1.19 |  | 1.28 |
| 45 | ABCE1_HUMAN | ATP-binding cassette sub-family E member 1 | P61221 | 0.43 |  |  |
| 46 | ABEC2_HUMAN | Probable C->U-editing enzyme APOBEC-2 | Q9Y235 |  | 0.66 | 1.21 |
| 47 | ABHD5_HUMAN | 1-acylglycerol-3-phosphate O-acyltransferase ABHD5 | Q8WTS1 | 0.95 | 1.01 | 0.76 |
| 48 | ABHDA_HUMAN | Mycophenolic acid acyl-glucuronide esterase, mitochondrial | Q9NUJ1 | 0.60 |  | 0.84 |
| 49 | ABHDB_HUMAN | Alpha/beta hydrolase domain-containing protein 11 | Q8NFV4 | 1.43 | 0.95 | 1.07 |
| 50 | ABHGA_HUMAN | Abhydrolase domain-containing protein 16A | O95870 |  | 1.54 | 1.11 |
| 51 | ABLM1_HUMAN | Actin-binding LIM protein 1 | O14639 | 1.24 |  | 1.11 |
| 52 | ABLM3_HUMAN | Actin-binding LIM protein 3 | O94929 | 1.05 |  | 0.87 |
| 53 | ACACB_HUMAN | Acetyl-CoA carboxylase 2 | O00763 |  | 0.45 | 1.12 |
| 54 | ACAD8_HUMAN | Isobutyryl-CoA dehydrogenase, mitochondrial | Q9UKU7 | 4.02 | 0.79 | 1.00 |
| 55 | ACAD9_HUMAN | Acyl-CoA dehydrogenase family member 9, mitochondrial | Q9H845 |  | 1.12 | 1.22 |
| 56 | ACADM_HUMAN | Medium-chain specific acyl-CoA dehydrogenase, mitochondrial | P11310 | 1.06 | 1.20 | 0.97 |
| 57 | ACADS_HUMAN | Short-chain specific acyl-CoA dehydrogenase, mitochondrial | P16219 |  | 0.65 | 1.03 |
| 58 | ACADV_HUMAN | Very long-chain specific acyl-CoA dehydrogenase, mitochondrial | P49748 | 1.49 |  | 1.13 |
| 59 | ACBD5_HUMAN | Acyl-CoA-binding domain-containing protein 5 | Q5T8D3 |  | 0.34 | 1.05 |
| 60 | ACBP_HUMAN | Acyl-CoA-binding protein | P07108 | 1.02 | 1.10 | 0.78 |
| 61 | ACD10_HUMAN | Acyl-CoA dehydrogenase family member 10 | Q6JQN1 |  | 0.96 | 0.58 |
| 62 | ACDSB_HUMAN | Short/branched chain specific acyl-CoA dehydrogenase, mitochondrial | P45954 | 0.60 | 1.01 | 0.95 |
| 63 | ACLY_HUMAN | ATP-citrate synthase | P53396 | 1.14 | 1.00 |  |
| 64 | ACO11_HUMAN | Acyl-coenzyme A thioesterase 11 | Q8WXI4 |  | 1.92 | 1.01 |
| 65 | ACO13_HUMAN | Acyl-coenzyme A thioesterase 13 | Q9NPJ3 | 0.84 | 1.75 | 1.46 |
| 66 | ACOC_HUMAN | Cytoplasmic aconitate hydratase | P21399 |  | 0.86 | 1.22 |
| 67 | ACON_HUMAN | Aconitate hydratase, mitochondrial | Q99798 | 1.42 | 0.99 | 0.93 |
| 68 | ACOT1_HUMAN | Acyl-coenzyme A thioesterase 1 | Q86TX2 | 1.00 | 1.14 | 1.00 |
| 69 | ACOT2_HUMAN | Acyl-coenzyme A thioesterase 2, mitochondrial | P49753 | 0.95 | 1.54 |  |
| 70 | ACOT9_HUMAN | Acyl-coenzyme A thioesterase 9, mitochondrial | Q9Y305 | 1.17 |  | 0.96 |
| 71 | ACOX1_HUMAN | Peroxisomal acyl-coenzyme A oxidase 1 | Q15067 |  |  |  |
| 72 | ACOX2_HUMAN | Peroxisomal acyl-coenzyme A oxidase 2 | Q99424 |  | 1.04 | 2.01 |
| 73 | ACOX3_HUMAN | Peroxisomal acyl-coenzyme A oxidase 3 | O15254 | 1.10 |  | 1.02 |
| 74 | ACPH_HUMAN | Acylamino-acid-releasing enzyme | P13798 | 0.41 | 1.46 |  |
| 75 | ACPM_HUMAN | Acyl carrier protein, mitochondrial | O14561 | 0.86 | 1.85 | 1.18 |
| 76 | ACS2L_HUMAN | Acetyl-coenzyme A synthetase 2-like, mitochondrial | Q9NUB1 |  |  | 1.00 |
| 77 | ACSA_HUMAN | Acetyl-coenzyme A synthetase, cytoplasmic | Q9NR19 | 0.70 |  | 0.82 |
| 78 | ACSF2_HUMAN | Acyl-CoA synthetase family member 2, mitochondrial | Q96CM8 |  | 0.96 | 1.32 |
| 79 | ACSF3_HUMAN | Acyl-CoA synthetase family member 3, mitochondrial | Q4G176 | 1.31 | 0.46 | 0.96 |
| 80 | ACSL1_HUMAN | Long-chain-fatty-acid--CoA ligase 1 | P33121 | 1.03 |  | 1.44 |
| 81 | ACSL3_HUMAN | Long-chain-fatty-acid--CoA ligase 3 | O95573 | 1.13 | 1.05 | 1.35 |
| 82 | ACSS3_HUMAN | Acyl-CoA synthetase short-chain family member 3, mitochondrial | Q9H6R3 | 1.56 | 1.11 | 1.12 |
| 83 | ACTB_HUMAN | Actin, cytoplasmic 1 | P60709 | 0.79 | 0.98 | 1.02 |
| 84 | ACTBL_HUMAN | Beta-actin-like protein 2 | Q562R1 | 1.80 | 0.91 | 1.09 |
| 85 | ACTC_HUMAN | Actin, alpha cardiac muscle 1 | P68032 | 1.39 | 0.77 | 1.64 |
| 86 | ACTG_HUMAN | Actin, cytoplasmic 2 | P63261 | 1.21 | 0.99 | 1.26 |
| 87 | ACTH_HUMAN | Actin, gamma-enteric smooth muscle | P63267 | 0.94 | 1.87 | 1.14 |
| 88 | ACTN1_HUMAN | Alpha-actinin-1 | P12814 | 0.76 |  | 1.08 |
| 89 | ACTN2_HUMAN | Alpha-actinin-2 | P35609 | 1.27 | 0.55 | 1.00 |
| 90 | ACTN4_HUMAN | Alpha-actinin-4 | O43707 |  |  | 0.88 |
| 91 | ACTS_HUMAN | Actin, alpha skeletal muscle | P68133 | 1.49 | 0.94 | 0.85 |
| 92 | ACTY_HUMAN | Beta-centractin | P42025 | 0.68 | 0.56 | 1.03 |
| 93 | ACTZ_HUMAN | Alpha-centractin | P61163 | 0.49 |  | 1.46 |
| 94 | ACY1_HUMAN | Aminoacylase-1 | Q03154 | 0.69 | 1.18 | 0.93 |
| 95 | ACYP2_HUMAN | Acylphosphatase-2 | P14621 | 0.57 | 0.77 | 1.17 |
| 96 | ADAL_HUMAN | Adenosine deaminase-like protein | Q6DHV7 | 1.66 |  | 0.89 |
| 97 | ADCK3_HUMAN | Chaperone activity of bc1 complex-like, mitochondrial | Q8NI60 | 0.72 |  | 0.95 |
| 98 | ADCY5_HUMAN | Adenylate cyclase type 5 | O95622 | 1.22 | 1.07 | 0.91 |
| 99 | ADCY7_HUMAN | Adenylate cyclase type 7 | P51828 | 0.37 |  | 1.17 |
| 100 | ADDA_HUMAN | Alpha-adducin | P35611 | 1.01 | 0.97 | 0.77 |
| 101 | ADDG_HUMAN | Gamma-adducin | Q9UEY8 | 0.35 | 1.04 | 1.08 |
| 102 | ADH1B_HUMAN | Alcohol dehydrogenase 1B | P00325 | 1.09 |  | 1.02 |
| 103 | ADHX_HUMAN | Alcohol dehydrogenase class-3 | P11766 | 0.76 | 0.87 | 1.33 |
| 104 | ADIPO_HUMAN | Adiponectin | Q15848 | 0.61 | 0.98 | 0.89 |
| 105 | ADIRF_HUMAN | Adipogenesis regulatory factor | Q15847 | 1.75 | 0.90 | 0.45 |
| 106 | ADK_HUMAN | Adenosine kinase | P55263 | 0.80 | 1.17 | 1.23 |
| 107 | ADPRH_HUMAN | [Protein ADP-ribosylarginine] hydrolase | P54922 | 0.20 |  | 0.81 |
| 108 | ADRM1_HUMAN | Proteasomal ubiquitin receptor ADRM1 | Q16186 |  | 1.09 | 1.27 |
| 109 | ADRO_HUMAN | NADPH:adrenodoxin oxidoreductase, mitochondrial | P22570 |  | 0.69 | 0.96 |
| 110 | ADT1_HUMAN | ADP/ATP translocase 1 | P12235 | 0.96 |  | 1.09 |
| 111 | ADT2_HUMAN | ADP/ATP translocase 2 | P05141 | 0.70 | 0.96 | 0.69 |
| 112 | ADT3_HUMAN | ADP/ATP translocase 3 | P12236 | 0.19 | 0.74 | 0.45 |
| 113 | ADX_HUMAN | Adrenodoxin, mitochondrial | P10109 | 0.48 |  | 1.65 |
| 114 | AEBP1_HUMAN | Adipocyte enhancer-binding protein 1 | Q8IUX7 | 1.22 | 1.54 | 1.10 |
| 115 | AFAD_HUMAN | Afadin | P55196 | 1.38 | 1.63 | 0.65 |
| 116 | AFAM_HUMAN | Afamin | P43652 | 0.97 | 0.89 | 1.14 |
| 117 | AFG32_HUMAN | AFG3-like protein 2 | Q9Y4W6 | 1.05 | 0.59 | 1.30 |
| 118 | AGFG1_HUMAN | Arf-GAP domain and FG repeat-containing protein 1 | P52594 | 1.32 | 1.04 |  |
| 119 | AGK_HUMAN | Acylglycerol kinase, mitochondrial | Q53H12 | 0.84 | 0.89 | 0.79 |
| 120 | AGM1_HUMAN | Phosphoacetylglucosamine mutase | O95394 | 2.03 |  | 0.96 |
| 121 | AGO2_HUMAN | Protein argonaute-2 | Q9UKV8 |  | 1.17 | 1.36 |
| 122 | AGRIN_HUMAN | Agrin | O00468 | 0.44 | 1.25 | 1.06 |
| 123 | AHNK_HUMAN | Neuroblast differentiation-associated protein AHNAK | Q09666 |  |  | 0.97 |
| 124 | AHSA1_HUMAN | Activator of 90 kDa heat shock protein ATPase homolog 1 | O95433 | 1.16 | 0.97 | 1.02 |
| 125 | AIFM1_HUMAN | Apoptosis-inducing factor 1, mitochondrial | O95831 | 1.33 | 0.87 |  |
| 126 | AIFM2_HUMAN | Apoptosis-inducing factor 2 | Q9BRQ8 |  | 0.59 | 0.96 |
| 127 | AIMP1_HUMAN | Aminoacyl tRNA synthase complex-interacting multifunctional protein 1 | Q12904 |  | 0.84 | 1.29 |
| 128 | AIMP2_HUMAN | Aminoacyl tRNA synthase complex-interacting multifunctional protein 2 | Q13155 | 0.77 |  | 0.77 |
| 129 | AIP_HUMAN | AH receptor-interacting protein | O00170 | 1.26 | 5.65 | 1.04 |
| 130 | AK1A1_HUMAN | Alcohol dehydrogenase [NADP(+)] | P14550 | 0.90 | 1.32 | 1.00 |
| 131 | AK1C1_HUMAN | Aldo-keto reductase family 1 member C1 | Q04828 | 0.86 | 0.96 | 0.90 |
| 132 | AKA12_HUMAN | A-kinase anchor protein 12 | Q02952 | 0.62 |  | 0.96 |
| 133 | AKAP1_HUMAN | A-kinase anchor protein 1, mitochondrial | Q92667 |  | 0.28 | 1.27 |
| 134 | AKAP2_HUMAN | A-kinase anchor protein 2 | Q9Y2D5 | 0.95 | 0.95 | 1.10 |
| 135 | AKP13_HUMAN | A-kinase anchor protein 13 | Q12802 | 1.71 | 1.39 | 1.03 |
| 136 | AL1A1_HUMAN | Retinal dehydrogenase 1 | P00352 | 0.66 |  | 1.02 |
| 137 | AL1A2_HUMAN | Retinal dehydrogenase 2 | O94788 | 0.75 | 0.74 | 1.00 |
| 138 | AL1B1_HUMAN | Aldehyde dehydrogenase X, mitochondrial | P30837 | 2.42 | 0.81 | 1.89 |
| 139 | AL1L1_HUMAN | Cytosolic 10-formyltetrahydrofolate dehydrogenase | O75891 | 1.17 | 0.98 | 0.89 |
| 140 | AL3A2_HUMAN | Fatty aldehyde dehydrogenase | P51648 | 0.98 | 0.64 | 0.94 |
| 141 | AL4A1_HUMAN | Delta-1-pyrroline-5-carboxylate dehydrogenase, mitochondrial | P30038 | 0.64 | 0.88 | 1.10 |
| 142 | AL7A1_HUMAN | Alpha-aminoadipic semialdehyde dehydrogenase | P49419 | 0.84 | 0.73 | 0.65 |
| 143 | AL9A1_HUMAN | 4-trimethylaminobutyraldehyde dehydrogenase | P49189 | 1.69 | 1.22 | 1.03 |
| 144 | ALAT1_HUMAN | Alanine aminotransferase 1 | P24298 | 1.27 |  |  |
| 145 | ALBU_HUMAN | Serum albumin | P02768 | 1.10 | 1.10 | 0.96 |
| 146 | ALDH2_HUMAN | Aldehyde dehydrogenase, mitochondrial | P05091 | 0.86 |  | 1.10 |
| 147 | ALDOA_HUMAN | Fructose-bisphosphate aldolase A | P04075 | 1.05 |  | 0.86 |
| 148 | ALDOC_HUMAN | Fructose-bisphosphate aldolase C | P09972 |  | 1.37 | 1.00 |
| 149 | ALDR_HUMAN | Aldose reductase | P15121 | 1.31 | 2.17 | 0.99 |
| 150 | ALG2_HUMAN | Alpha-1,3/1,6-mannosyltransferase ALG2 | Q9H553 |  |  | 0.67 |
| 151 | ALG9_HUMAN | Alpha-1,2-mannosyltransferase ALG9 | Q9H6U8 | 1.20 |  | 0.55 |
| 152 | ALPK3_HUMAN | Alpha-protein kinase 3 | Q96L96 | 0.09 |  | 0.97 |
| 153 | ALR_HUMAN | FAD-linked sulfhydryl oxidase ALR | P55789 |  | 0.90 | 0.89 |
| 154 | ALS_HUMAN | Insulin-like growth factor-binding protein complex acid labile subunit | P35858 | 1.53 | 1.06 | 1.15 |
| 155 | AMBP_HUMAN | Protein AMBP | P02760 | 1.11 |  | 0.84 |
| 156 | AMD_HUMAN | Peptidyl-glycine alpha-amidating monooxygenase | P19021 | 1.12 | 0.65 | 0.95 |
| 157 | AMPB_HUMAN | Aminopeptidase B | Q9H4A4 | 1.26 | 2.27 | 0.62 |
| 158 | AMPL_HUMAN | Cytosol aminopeptidase | P28838 |  | 1.14 | 1.45 |
| 159 | AMRP_HUMAN | Alpha-2-macroglobulin receptor-associated protein | P30533 | 0.80 | 0.95 | 1.03 |
| 160 | AN32A_HUMAN | Acidic leucine-rich nuclear phosphoprotein 32 family member A | P39687 | 2.86 | 1.17 | 1.07 |
| 161 | AN32E_HUMAN | Acidic leucine-rich nuclear phosphoprotein 32 family member E | Q9BTT0 |  | 1.34 | 1.23 |
| 162 | ANF_HUMAN | Natriuretic peptides A | P01160 | 1.31 |  | 0.76 |
| 163 | ANGI_HUMAN | Angiogenin | P03950 | 0.44 |  | 0.60 |
| 164 | ANGT_HUMAN | Angiotensinogen | P01019 | 0.63 | 1.92 | 0.81 |
| 165 | ANK1_HUMAN | Ankyrin-1 | P16157 | 0.72 |  |  |
| 166 | ANK2_HUMAN | Ankyrin-2 | Q01484 | 0.77 | 0.90 | 0.97 |
| 167 | ANK3_HUMAN | Ankyrin-3 | Q12955 | 1.00 |  | 0.94 |
| 168 | ANKR1_HUMAN | Ankyrin repeat domain-containing protein 1 | Q15327 |  |  | 0.56 |
| 169 | ANKR2_HUMAN | Ankyrin repeat domain-containing protein 2 | Q9GZV1 | 0.74 | 2.01 | 0.94 |
| 170 | ANKY2_HUMAN | Ankyrin repeat and MYND domain-containing protein 2 | Q8IV38 | 0.86 | 0.87 | 0.83 |
| 171 | ANM1_HUMAN | Protein arginine N-methyltransferase 1 | Q99873 | 1.05 | 1.25 | 0.78 |
| 172 | ANM5_HUMAN | Protein arginine N-methyltransferase 5 | O14744 | 1.06 | 0.19 | 1.10 |
| 173 | ANO6_HUMAN | Anoctamin-6 | Q4KMQ2 |  | 0.97 |  |
| 174 | ANT3_HUMAN | Antithrombin-III | P01008 |  |  | 0.92 |
| 175 | ANX11_HUMAN | Annexin A11 | P50995 | 1.21 |  | 1.09 |
| 176 | ANXA1_HUMAN | Annexin A1 | P04083 | 1.22 | 1.21 | 0.95 |
| 177 | ANXA2_HUMAN | Annexin A2 | P07355 | 0.58 | 0.86 | 0.89 |
| 178 | ANXA3_HUMAN | Annexin A3 | P12429 | 1.38 |  | 1.01 |
| 179 | ANXA4_HUMAN | Annexin A4 | P09525 | 1.27 | 0.56 | 0.88 |
| 180 | ANXA5_HUMAN | Annexin A5 | P08758 | 1.25 | 0.77 | 1.00 |
| 181 | ANXA6_HUMAN | Annexin A6 | P08133 | 1.17 |  | 1.20 |
| 182 | ANXA7_HUMAN | Annexin A7 | P20073 | 1.41 |  | 1.08 |
| 183 | AOC3_HUMAN | Membrane primary amine oxidase | Q16853 | 1.32 | 1.07 | 1.21 |
| 184 | AOFA_HUMAN | Amine oxidase [flavin-containing] A | P21397 | 1.89 | 3.10 | 1.01 |
| 185 | AOFB_HUMAN | Amine oxidase [flavin-containing] B | P27338 | 1.26 | 1.04 | 0.94 |
| 186 | AP1B1_HUMAN | AP-1 complex subunit beta-1 | Q10567 | 1.18 | 1.01 | 1.45 |
| 187 | AP1G1_HUMAN | AP-1 complex subunit gamma-1 | O43747 |  | 1.09 | 0.77 |
| 188 | AP1M1_HUMAN | AP-1 complex subunit mu-1 | Q9BXS5 |  | 1.05 | 0.95 |
| 189 | AP2A1_HUMAN | AP-2 complex subunit alpha-1 | O95782 | 1.24 | 0.89 | 1.13 |
| 190 | AP2A2_HUMAN | AP-2 complex subunit alpha-2 | O94973 | 0.64 | 1.26 | 0.84 |
| 191 | AP2B1_HUMAN | AP-2 complex subunit beta | P63010 | 0.58 | 1.08 | 0.94 |
| 192 | AP2M1_HUMAN | AP-2 complex subunit mu | Q96CW1 | 0.71 | 1.08 | 1.03 |
| 193 | AP2S1_HUMAN | AP-2 complex subunit sigma | P53680 | 0.39 | 2.07 | 1.09 |
| 194 | AP3B1_HUMAN | AP-3 complex subunit beta-1 | O00203 | 0.84 |  | 0.94 |
| 195 | AP3D1_HUMAN | AP-3 complex subunit delta-1 | O14617 |  | 0.82 | 1.09 |
| 196 | AP4A_HUMAN | Bis(5'-nucleosyl)-tetraphosphatase [asymmetrical] | P50583 | 0.90 | 3.13 | 0.63 |
| 197 | APEX1_HUMAN | DNA-(apurinic or apyrimidinic site) lyase | P27695 | 0.39 |  | 1.12 |
| 198 | API5_HUMAN | Apoptosis inhibitor 5 | Q9BZZ5 | 0.94 | 1.01 | 0.59 |
| 199 | APMAP_HUMAN | Adipocyte plasma membrane-associated protein | Q9HDC9 | 4.21 | 0.85 | 1.17 |
| 200 | APOA1_HUMAN | Apolipoprotein A-I | P02647 | 0.74 | 0.95 | 0.93 |
| 201 | APOA2_HUMAN | Apolipoprotein A-II | P02652 | 3.77 | 2.03 | 0.77 |
| 202 | APOA4_HUMAN | Apolipoprotein A-IV | P06727 |  | 1.09 | 0.80 |
| 203 | APOB_HUMAN | Apolipoprotein B-100 | P04114 |  | 0.59 | 1.08 |
| 204 | APOC1_HUMAN | Apolipoprotein C-I | P02654 |  | 1.33 | 1.20 |
| 205 | APOC2_HUMAN | Apolipoprotein C-II | P02655 | 1.49 | 0.70 | 1.30 |
| 206 | APOC3_HUMAN | Apolipoprotein C-III | P02656 | 1.08 | 0.94 | 1.18 |
| 207 | APOE_HUMAN | Apolipoprotein E | P02649 | 1.10 | 0.82 | 1.07 |
| 208 | APOH_HUMAN | Beta-2-glycoprotein 1 | P02749 | 1.28 | 1.04 | 0.95 |
| 209 | APOL2_HUMAN | Apolipoprotein L2 | Q9BQE5 | 1.92 | 1.61 | 0.83 |
| 210 | APT_HUMAN | Adenine phosphoribosyltransferase | P07741 | 0.71 | 1.33 | 0.79 |
| 211 | AQP1_HUMAN | Aquaporin-1 | P29972 | 0.98 | 1.17 | 1.39 |
| 212 | ARC1A_HUMAN | Actin-related protein 2/3 complex subunit 1A | Q92747 | 0.89 | 1.37 | 1.10 |
| 213 | ARF1_HUMAN | ADP-ribosylation factor 1 | P84077 | 0.77 |  | 1.06 |
| 214 | ARF4_HUMAN | ADP-ribosylation factor 4 | P18085 | 1.91 | 0.95 | 0.78 |
| 215 | ARF5_HUMAN | ADP-ribosylation factor 5 | P84085 | 1.47 |  | 1.01 |
| 216 | ARF6_HUMAN | ADP-ribosylation factor 6 | P62330 | 0.42 | 0.67 | 1.11 |
| 217 | ARHG6_HUMAN | Rho guanine nucleotide exchange factor 6 | Q15052 | 0.93 | 0.96 | 0.71 |
| 218 | ARHL1_HUMAN | [Protein ADP-ribosylarginine] hydrolase-like protein 1 | Q8NDY3 | 0.69 |  | 1.14 |
| 219 | ARHL2_HUMAN | Poly(ADP-ribose) glycohydrolase ARH3 | Q9NX46 | 0.24 |  | 0.70 |
| 220 | ARI2_HUMAN | E3 ubiquitin-protein ligase ARIH2 | O95376 | 2.21 | 1.16 |  |
| 221 | ARK72_HUMAN | Aflatoxin B1 aldehyde reductase member 2 | O43488 | 1.49 | 0.55 | 0.93 |
| 222 | ARL1_HUMAN | ADP-ribosylation factor-like protein 1 | P40616 | 0.30 | 2.42 | 1.08 |
| 223 | ARL2_HUMAN | ADP-ribosylation factor-like protein 2 | P36404 | 0.13 | 0.47 | 1.12 |
| 224 | ARL3_HUMAN | ADP-ribosylation factor-like protein 3 | P36405 | 0.38 | 1.31 | 0.92 |
| 225 | ARL8B_HUMAN | ADP-ribosylation factor-like protein 8B | Q9NVJ2 | 1.20 | 0.86 | 0.93 |
| 226 | ARMC1_HUMAN | Armadillo repeat-containing protein 1 | Q9NVT9 |  | 2.21 | 0.74 |
| 227 | ARMC8_HUMAN | Armadillo repeat-containing protein 8 | Q8IUR7 | 1.17 |  | 1.08 |
| 228 | ARMX1_HUMAN | Armadillo repeat-containing X-linked protein 1 | Q9P291 |  | 1.91 | 0.84 |
| 229 | ARMX2_HUMAN | Armadillo repeat-containing X-linked protein 2 | Q7L311 | 1.14 | 1.10 | 0.90 |
| 230 | ARP10_HUMAN | Actin-related protein 10 | Q9NZ32 | 0.45 | 1.24 | 1.04 |
| 231 | ARP19_HUMAN | cAMP-regulated phosphoprotein 19 | P56211 | 0.71 |  | 0.87 |
| 232 | ARP2_HUMAN | Actin-related protein 2 | P61160 | 0.93 | 0.68 | 1.20 |
| 233 | ARP3_HUMAN | Actin-related protein 3 | P61158 |  |  | 1.11 |
| 234 | ARP5L_HUMAN | Actin-related protein 2/3 complex subunit 5-like protein | Q9BPX5 | 0.71 | 1.08 | 1.50 |
| 235 | ARPC2_HUMAN | Actin-related protein 2/3 complex subunit 2 | O15144 | 0.77 |  | 0.93 |
| 236 | ARPC3_HUMAN | Actin-related protein 2/3 complex subunit 3 | O15145 | 1.21 | 1.46 | 1.12 |
| 237 | ARPC4_HUMAN | Actin-related protein 2/3 complex subunit 4 | P59998 | 1.15 | 1.03 | 1.13 |
| 238 | ARPC5_HUMAN | Actin-related protein 2/3 complex subunit 5 | O15511 | 0.96 | 1.18 | 0.64 |
| 239 | ARSA_HUMAN | Arylsulfatase A | P15289 |  | 0.59 | 0.86 |
| 240 | ARVC_HUMAN | Armadillo repeat protein deleted in velo-cardio-facial syndrome | O00192 | 0.62 |  | 0.79 |
| 241 | ASAH1_HUMAN | Acid ceramidase | Q13510 |  | 0.75 | 1.06 |
| 242 | ASNA_HUMAN | ATPase ASNA1 | O43681 | 1.04 | 2.73 | 1.18 |
| 243 | ASPH_HUMAN | Aspartyl/asparaginyl beta-hydroxylase | Q12797 |  |  | 0.99 |
| 244 | ASPN_HUMAN | Asporin | Q9BXN1 | 0.74 | 2.05 | 1.07 |
| 245 | ASSY_HUMAN | Argininosuccinate synthase | P00966 | 0.63 | 0.60 | 1.11 |
| 246 | AT1A1_HUMAN | Sodium/potassium-transporting ATPase subunit alpha-1 | P05023 | 0.42 | 1.22 | 1.02 |
| 247 | AT1A2_HUMAN | Sodium/potassium-transporting ATPase subunit alpha-2 | P50993 | 0.56 | 0.47 | 0.95 |
| 248 | AT1A3_HUMAN | Sodium/potassium-transporting ATPase subunit alpha-3 | P13637 |  |  | 1.04 |
| 249 | AT1B1_HUMAN | Sodium/potassium-transporting ATPase subunit beta-1 | P05026 | 0.91 | 1.25 | 1.07 |
| 250 | AT1B3_HUMAN | Sodium/potassium-transporting ATPase subunit beta-3 | P54709 |  | 0.95 | 1.19 |
| 251 | AT2A2_HUMAN | Sarcoplasmic/endoplasmic reticulum calcium ATPase 2 | P16615 |  | 0.95 | 1.09 |
| 252 | AT2B4_HUMAN | Plasma membrane calcium-transporting ATPase 4 | P23634 | 1.12 | 0.90 | 0.63 |
| 253 | AT5EL_HUMAN | ATP synthase subunit epsilon-like protein, mitochondrial | Q5VTU8 | 4.41 | 10.86 | 0.84 |
| 254 | AT5F1_HUMAN | ATP synthase F(0) complex subunit B1, mitochondrial | P24539 | 0.95 | 0.44 | 1.22 |
| 255 | AT5G3_HUMAN | ATP synthase F(0) complex subunit C3, mitochondrial | P48201 | 1.51 |  | 0.84 |
| 256 | AT8A1_HUMAN | Phospholipid-transporting ATPase IA | Q9Y2Q0 |  | 0.95 | 0.75 |
| 257 | ATAD1_HUMAN | ATPase family AAA domain-containing protein 1 | Q8NBU5 | 1.94 |  | 1.45 |
| 258 | ATD3A_HUMAN | ATPase family AAA domain-containing protein 3A | Q9NVI7 |  | 0.83 | 1.17 |
| 259 | ATE1_HUMAN | Arginyl-tRNA--protein transferase 1 | O95260 | 1.16 | 0.91 | 0.99 |
| 260 | ATG3_HUMAN | Ubiquitin-like-conjugating enzyme ATG3 | Q9NT62 | 1.63 | 0.99 | 1.08 |
| 261 | ATG4B_HUMAN | Cysteine protease ATG4B | Q9Y4P1 | 1.98 | 0.98 | 0.91 |
| 262 | ATIF1_HUMAN | ATPase inhibitor, mitochondrial | Q9UII2 | 0.08 | 1.49 | 0.92 |
| 263 | ATLA3_HUMAN | Atlastin-3 | Q6DD88 | 1.56 | 1.06 | 0.68 |
| 264 | ATP5E_HUMAN | ATP synthase subunit epsilon, mitochondrial | P56381 | 1.84 | 0.23 | 1.18 |
| 265 | ATP5H_HUMAN | ATP synthase subunit d, mitochondrial | O75947 | 0.80 | 3.77 |  |
| 266 | ATP5I_HUMAN | ATP synthase subunit e, mitochondrial | P56385 | 1.27 | 0.55 | 0.84 |
| 267 | ATP5J_HUMAN | ATP synthase-coupling factor 6, mitochondrial | P18859 | 0.36 | 1.03 | 1.05 |
| 268 | ATP5L_HUMAN | ATP synthase subunit g, mitochondrial | O75964 | 1.11 |  | 1.00 |
| 269 | ATP5S_HUMAN | ATP synthase subunit s, mitochondrial | Q99766 | 1.64 | 0.91 | 1.05 |
| 270 | ATP6_HUMAN | ATP synthase subunit a | P00846 | 2.29 | 0.73 | 0.85 |
| 271 | ATPA_HUMAN | ATP synthase subunit alpha, mitochondrial | P25705 | 0.79 |  | 1.21 |
| 272 | ATPB_HUMAN | ATP synthase subunit beta, mitochondrial | P06576 | 0.82 |  | 0.94 |
| 273 | ATPD_HUMAN | ATP synthase subunit delta, mitochondrial | P30049 | 0.86 |  | 1.02 |
| 274 | ATPF1_HUMAN | ATP synthase mitochondrial F1 complex assembly factor 1 | Q5TC12 | 0.39 |  | 0.91 |
| 275 | ATPF2_HUMAN | ATP synthase mitochondrial F1 complex assembly factor 2 | Q8N5M1 | 0.63 | 1.94 | 0.90 |
| 276 | ATPG_HUMAN | ATP synthase subunit gamma, mitochondrial | P36542 | 1.13 |  | 1.03 |
| 277 | ATPK_HUMAN | ATP synthase subunit f, mitochondrial | P56134 | 1.33 | 7.94 | 1.33 |
| 278 | ATPO_HUMAN | ATP synthase subunit O, mitochondrial | P48047 | 0.97 | 0.74 | 1.11 |
| 279 | ATRAP_HUMAN | Type-1 angiotensin II receptor-associated protein | Q6RW13 | 1.15 | 1.26 | 0.45 |
| 280 | ATX10_HUMAN | Ataxin-10 | Q9UBB4 | 0.79 | 2.09 | 0.56 |
| 281 | AUHM_HUMAN | Methylglutaconyl-CoA hydratase, mitochondrial | Q13825 | 0.86 | 0.95 | 1.04 |
| 282 | B2L13_HUMAN | Bcl-2-like protein 13 | Q9BXK5 | 1.03 | 0.79 | 1.55 |
| 283 | B2MG_HUMAN | Beta-2-microglobulin | P61769 | 1.46 | 0.99 |  |
| 284 | B3A3_HUMAN | Anion exchange protein 3 | P48751 | 2.00 | 0.90 | 1.10 |
| 285 | B3AT_HUMAN | Band 3 anion transport protein | P02730 | 2.27 | 1.09 | 1.11 |
| 286 | B3GLT_HUMAN | Beta-1,3-glucosyltransferase | Q6Y288 | 2.21 | 0.64 | 0.85 |
| 287 | BAF_HUMAN | Barrier-to-autointegration factor | O75531 | 1.00 |  |  |
| 288 | BAG2_HUMAN | BAG family molecular chaperone regulator 2 | O95816 | 1.00 | 2.17 | 0.65 |
| 289 | BAG3_HUMAN | BAG family molecular chaperone regulator 3 | O95817 | 0.59 |  | 0.98 |
| 290 | BAG5_HUMAN | BAG family molecular chaperone regulator 5 | Q9UL15 |  | 1.06 | 0.69 |
| 291 | BAG6_HUMAN | Large proline-rich protein BAG6 | P46379 | 0.90 | 0.40 | 1.23 |
| 292 | BAP29_HUMAN | B-cell receptor-associated protein 29 | Q9UHQ4 |  | 0.85 | 0.98 |
| 293 | BAP31_HUMAN | B-cell receptor-associated protein 31 | P51572 | 1.05 | 1.17 | 0.78 |
| 294 | BASI_HUMAN | Basigin | P35613 | 0.77 | 1.41 | 0.83 |
| 295 | BAX_HUMAN | Apoptosis regulator BAX | Q07812 |  | 1.32 | 0.74 |
| 296 | BCAM_HUMAN | Basal cell adhesion molecule | P50895 | 0.64 | 0.84 | 0.87 |
| 297 | BCAT2_HUMAN | Branched-chain-amino-acid aminotransferase, mitochondrial | O15382 | 0.72 | 2.63 | 1.01 |
| 298 | BCDO2_HUMAN | Beta,beta-carotene 9',10'-oxygenase | Q9BYV7 |  | 1.25 | 1.30 |
| 299 | BCKD_HUMAN | [3-methyl-2-oxobutanoate dehydrogenase [lipoamide]] kinase, mitochondrial | O14874 | 0.98 | 0.88 | 1.17 |
| 300 | BCS1_HUMAN | Mitochondrial chaperone BCS1 | Q9Y276 | 0.80 | 0.69 | 0.93 |
| 301 | BDH_HUMAN | D-beta-hydroxybutyrate dehydrogenase, mitochondrial | Q02338 | 0.51 |  | 1.13 |
| 302 | BDH2_HUMAN | 3-hydroxybutyrate dehydrogenase type 2 | Q9BUT1 |  | 0.70 | 1.02 |
| 303 | BGH3_HUMAN | Transforming growth factor-beta-induced protein ig-h3 | Q15582 |  | 0.95 | 1.05 |
| 304 | BID_HUMAN | BH3-interacting domain death agonist | P55957 | 1.49 | 1.94 | 1.58 |
| 305 | BIEA_HUMAN | Biliverdin reductase A | P53004 | 2.42 | 0.35 | 0.86 |
| 306 | BINCA_HUMAN | Bcl10-interacting CARD protein | Q96LW7 |  | 1.31 | 0.60 |
| 307 | BLMH_HUMAN | Bleomycin hydrolase | Q13867 |  | 1.15 | 0.76 |
| 308 | BLVRB_HUMAN | Flavin reductase (NADPH) | P30043 | 0.86 | 2.61 | 0.89 |
| 309 | BMP10_HUMAN | Bone morphogenetic protein 10 | O95393 | 0.81 | 1.33 | 0.97 |
| 310 | BNIP3_HUMAN | BCL2/adenovirus E1B 19 kDa protein-interacting protein 3 | Q12983 | 2.54 |  | 1.15 |
| 311 | BOLA1_HUMAN | BolA-like protein 1 | Q9Y3E2 | 0.27 |  | 0.91 |
| 312 | BOLA2_HUMAN | BolA-like protein 2 | Q9H3K6 | 0.95 |  | 0.97 |
| 313 | BORG4_HUMAN | Cdc42 effector protein 4 | Q9H3Q1 | 1.33 |  | 0.51 |
| 314 | BPHL_HUMAN | Valacyclovir hydrolase | Q86WA6 | 0.86 | 1.03 | 0.86 |
| 315 | BPNT1_HUMAN | 3'(2'),5'-bisphosphate nucleotidase 1 | O95861 | 0.96 |  | 1.32 |
| 316 | BST1_HUMAN | ADP-ribosyl cyclase/cyclic ADP-ribose hydrolase 2 | Q10588 |  | 0.91 | 1.39 |
| 317 | BST2_HUMAN | Bone marrow stromal antigen 2 | Q10589 | 0.39 | 2.23 | 0.86 |
| 318 | BT3L4_HUMAN | Transcription factor BTF3 homolog 4 | Q96K17 | 1.14 | 0.99 | 1.52 |
| 319 | BTD_HUMAN | Biotinidase | P43251 | 1.69 | 0.71 |  |
| 320 | BTF3_HUMAN | Transcription factor BTF3 | P20290 | 0.84 | 0.97 | 0.85 |
| 321 | BZW1_HUMAN | Basic leucine zipper and W2 domain-containing protein 1 | Q7L1Q6 |  | 1.14 | 1.25 |
| 322 | BZW2_HUMAN | Basic leucine zipper and W2 domain-containing protein 2 | Q9Y6E2 | 1.15 | 0.81 | 0.84 |
| 323 | C10_HUMAN | Protein C10 | Q99622 | 0.91 | 0.98 | 0.76 |
| 324 | C1QB_HUMAN | Complement C1q subcomponent subunit B | P02746 | 1.46 | 1.09 | 0.77 |
| 325 | C1QBP_HUMAN | Complement component 1 Q subcomponent-binding protein, mitochondrial | Q07021 | 1.12 | 1.17 |  |
| 326 | C1QC_HUMAN | Complement C1q subcomponent subunit C | P02747 | 0.26 | 1.39 | 1.36 |
| 327 | C1QR1_HUMAN | Complement component C1q receptor | Q9NPY3 |  | 1.10 | 1.22 |
| 328 | C1R_HUMAN | Complement C1r subcomponent | P00736 |  | 1.71 | 0.89 |
| 329 | C1S_HUMAN | Complement C1s subcomponent | P09871 |  | 0.59 |  |
| 330 | C1TC_HUMAN | C-1-tetrahydrofolate synthase, cytoplasmic | P11586 | 0.69 | 0.79 | 1.00 |
| 331 | C560_HUMAN | Succinate dehydrogenase cytochrome b560 subunit, mitochondrial | Q99643 | 1.05 | 0.93 | 1.15 |
| 332 | CA123_HUMAN | UPF0587 protein C1orf123 | Q9NWV4 | 1.47 | 1.25 | 1.09 |
| 333 | CA2D1_HUMAN | Voltage-dependent calcium channel subunit alpha-2/delta-1 | P54289 | 0.83 | 0.86 | 0.76 |
| 334 | CA2D2_HUMAN | Voltage-dependent calcium channel subunit alpha-2/delta-2 | Q9NY47 | 1.39 |  | 1.07 |
| 335 | CAB39_HUMAN | Calcium-binding protein 39 | Q9Y376 | 0.59 | 0.90 | 1.05 |
| 336 | CACP_HUMAN | Carnitine O-acetyltransferase | P43155 | 0.62 | 0.95 | 2.09 |
| 337 | CAD13_HUMAN | Cadherin-13 | P55290 | 1.17 | 0.04 | 0.97 |
| 338 | CADH2_HUMAN | Cadherin-2 | P19022 | 0.61 |  | 0.80 |
| 339 | CADH5_HUMAN | Cadherin-5 | P33151 | 1.28 | 1.04 | 0.93 |
| 340 | CADM1_HUMAN | Cell adhesion molecule 1 | Q9BY67 |  | 0.41 |  |
| 341 | CAF17_HUMAN | Putative transferase CAF17, mitochondrial | Q5T440 | 0.92 | 0.72 | 1.03 |
| 342 | CAH1_HUMAN | Carbonic anhydrase 1 | P00915 | 1.51 | 1.26 | 1.11 |
| 343 | CAH2_HUMAN | Carbonic anhydrase 2 | P00918 | 1.01 | 0.44 | 1.14 |
| 344 | CAH4_HUMAN | Carbonic anhydrase 4 | P22748 | 0.79 | 2.07 | 0.96 |
| 345 | CALD1_HUMAN | Caldesmon | Q05682 | 1.24 | 1.33 | 1.07 |
| 346 | CALM_HUMAN | Calmodulin | P62158 | 1.05 |  | 0.62 |
| 347 | CALR_HUMAN | Calreticulin | P27797 | 1.29 | 0.70 | 0.90 |
| 348 | CALU_HUMAN | Calumenin | O43852 | 0.35 |  | 1.21 |
| 349 | CALX_HUMAN | Calnexin | P27824 | 0.70 | 0.95 | 0.95 |
| 350 | CAN1_HUMAN | Calpain-1 catalytic subunit | P07384 | 0.70 | 1.33 | 0.97 |
| 351 | CAN2_HUMAN | Calpain-2 catalytic subunit | P17655 | 1.18 | 1.41 | 1.16 |
| 352 | CANB1_HUMAN | Calcineurin subunit B type 1 | P63098 | 0.72 | 0.78 | 1.01 |
| 353 | CAND1_HUMAN | Cullin-associated NEDD8-dissociated protein 1 | Q86VP6 |  | 0.51 | 1.01 |
| 354 | CAND2_HUMAN | Cullin-associated NEDD8-dissociated protein 2 | O75155 | 0.91 | 0.90 | 1.03 |
| 355 | CAP1_HUMAN | Adenylyl cyclase-associated protein 1 | Q01518 | 1.14 |  | 0.64 |
| 356 | CAP2_HUMAN | Adenylyl cyclase-associated protein 2 | P40123 |  | 0.61 | 1.46 |
| 357 | CAPR1_HUMAN | Caprin-1 | Q14444 | 0.59 |  | 1.23 |
| 358 | CAPS1_HUMAN | Calcium-dependent secretion activator 1 | Q9ULU8 |  | 0.39 | 0.89 |
| 359 | CAPZB_HUMAN | F-actin-capping protein subunit beta | P47756 |  |  | 1.69 |
| 360 | CASQ2_HUMAN | Calsequestrin-2 | O14958 |  |  | 0.89 |
| 361 | CATA_HUMAN | Catalase | P04040 | 0.47 | 1.36 |  |
| 362 | CATB_HUMAN | Cathepsin B | P07858 | 0.61 |  | 0.95 |
| 363 | CATD_HUMAN | Cathepsin D | P07339 | 0.78 | 1.07 | 1.06 |
| 364 | CATG_HUMAN | Cathepsin G | P08311 | 0.95 | 0.42 | 1.19 |
| 365 | CATL1_HUMAN | Cathepsin L1 | P07711 | 0.26 | 0.69 | 0.70 |
| 366 | CATZ_HUMAN | Cathepsin Z | Q9UBR2 | 0.29 | 0.79 | 1.05 |
| 367 | CAV1_HUMAN | Caveolin-1 | Q03135 | 1.98 | 0.74 | 1.28 |
| 368 | CAV2_HUMAN | Caveolin-2 | P51636 | 0.51 | 0.59 |  |
| 369 | CAV3_HUMAN | Caveolin-3 | P56539 |  | 0.90 | 0.90 |
| 370 | CAZA1_HUMAN | F-actin-capping protein subunit alpha-1 | P52907 | 1.00 | 1.67 | 0.90 |
| 371 | CAZA2_HUMAN | F-actin-capping protein subunit alpha-2 | P47755 | 0.82 |  | 1.01 |
| 372 | CB047_HUMAN | Uncharacterized protein C2orf47, mitochondrial | Q8WWC4 | 0.50 |  | 1.00 |
| 373 | CBG_HUMAN | Corticosteroid-binding globulin | P08185 | 2.68 |  | 0.73 |
| 374 | CBPA3_HUMAN | Mast cell carboxypeptidase A | P15088 | 1.14 |  | 0.63 |
| 375 | CBPQ_HUMAN | Carboxypeptidase Q | Q9Y646 | 1.06 | 0.95 | 1.05 |
| 376 | CBR1_HUMAN | Carbonyl reductase [NADPH] 1 | P16152 | 2.36 | 1.08 | 0.98 |
| 377 | CBR4_HUMAN | Carbonyl reductase family member 4 | Q8N4T8 | 0.65 | 1.22 | 0.69 |
| 378 | CBX1_HUMAN | Chromobox protein homolog 1 | P83916 | 2.47 |  | 0.88 |
| 379 | CBX3_HUMAN | Chromobox protein homolog 3 | Q13185 | 1.09 | 1.60 | 1.26 |
| 380 | CC127_HUMAN | Coiled-coil domain-containing protein 127 | Q96BQ5 | 1.19 | 0.97 | 1.07 |
| 381 | CC141_HUMAN | Coiled-coil domain-containing protein 141 | Q6ZP82 | 0.96 | 1.74 | 1.05 |
| 382 | CC90B_HUMAN | Coiled-coil domain-containing protein 90B, mitochondrial | Q9GZT6 | 0.79 | 2.31 | 1.10 |
| 383 | CCAR2_HUMAN | Cell cycle and apoptosis regulator protein 2 | Q8N163 |  | 0.95 | 0.94 |
| 384 | CCD22_HUMAN | Coiled-coil domain-containing protein 22 | O60826 | 0.95 | 0.76 | 1.01 |
| 385 | CCD47_HUMAN | Coiled-coil domain-containing protein 47 | Q96A33 |  | 0.45 | 0.94 |
| 386 | CCD50_HUMAN | Coiled-coil domain-containing protein 50 | Q8IVM0 | 0.97 |  | 1.14 |
| 387 | CCD58_HUMAN | Coiled-coil domain-containing protein 58 | Q4VC31 | 0.90 | 2.56 | 1.43 |
| 388 | CCD69_HUMAN | Coiled-coil domain-containing protein 69 | A6NI79 |  | 1.07 | 0.96 |
| 389 | CCHL_HUMAN | Cytochrome c-type heme lyase | P53701 | 0.84 | 0.90 | 1.28 |
| 390 | CCS_HUMAN | Copper chaperone for superoxide dismutase | O14618 | 2.73 | 2.91 |  |
| 391 | CD109_HUMAN | CD109 antigen | Q6YHK3 | 1.06 | 0.82 | 0.96 |
| 392 | CD14_HUMAN | Monocyte differentiation antigen CD14 | P08571 |  | 1.02 | 0.96 |
| 393 | CD36_HUMAN | Platelet glycoprotein 4 | P16671 | 0.67 | 0.61 | 0.91 |
| 394 | CD44_HUMAN | CD44 antigen | P16070 | 0.90 |  | 1.22 |
| 395 | CD47_HUMAN | Leukocyte surface antigen CD47 | Q08722 | 0.85 | 1.06 | 0.84 |
| 396 | CD59_HUMAN | CD59 glycoprotein | P13987 | 2.31 | 1.07 | 5.58 |
| 397 | CD63_HUMAN | CD63 antigen | P08962 | 4.29 |  | 1.34 |
| 398 | CD81_HUMAN | CD81 antigen | P60033 | 1.01 | 0.74 | 0.87 |
| 399 | CD9_HUMAN | CD9 antigen | P21926 | 0.50 | 0.82 | 0.74 |
| 400 | CD99_HUMAN | CD99 antigen | P14209 | 0.81 | 1.41 | 0.82 |
| 401 | CDC37_HUMAN | Hsp90 co-chaperone Cdc37 | Q16543 | 0.54 | 0.68 | 1.24 |
| 402 | CDC42_HUMAN | Cell division control protein 42 homolog | P60953 | 0.89 | 0.49 | 1.00 |
| 403 | CDIPT_HUMAN | CDP-diacylglycerol--inositol 3-phosphatidyltransferase | O14735 | 1.09 |  | 0.84 |
| 404 | CDK18_HUMAN | Cyclin-dependent kinase 18 | Q07002 | 0.87 |  | 1.79 |
| 405 | CDK4_HUMAN | Cyclin-dependent kinase 4 | P11802 | 0.87 | 6.03 | 1.16 |
| 406 | CDS2_HUMAN | Phosphatidate cytidylyltransferase 2 | O95674 | 1.89 | 1.85 | 0.68 |
| 407 | CDV3_HUMAN | Protein CDV3 homolog | Q9UKY7 | 1.39 | 1.08 | 0.98 |
| 408 | CECR5_HUMAN | Cat eye syndrome critical region protein 5 | Q9BXW7 | 0.27 | 1.56 | 0.92 |
| 409 | CERU_HUMAN | Ceruloplasmin | P00450 | 1.14 | 0.85 | 1.71 |
| 410 | CF136_HUMAN | Uncharacterized protein C6orf136 | Q5SQH8 | 1.64 |  |  |
| 411 | CFAB_HUMAN | Complement factor B | P00751 |  |  |  |
| 412 | CFAD_HUMAN | Complement factor D | P00746 | 1.34 |  | 0.64 |
| 413 | CFAH_HUMAN | Complement factor H | P08603 | 1.28 | 0.86 | 0.83 |
| 414 | CFAI_HUMAN | Complement factor I | P05156 | 1.04 | 0.56 | 0.65 |
| 415 | CG055_HUMAN | UPF0562 protein C7orf55 | Q96HJ9 | 0.97 |  | 1.36 |
| 416 | CH082_HUMAN | UPF0598 protein C8orf82 | Q6P1X6 | 1.28 | 0.67 | 1.09 |
| 417 | CH10_HUMAN | 10 kDa heat shock protein, mitochondrial | P61604 | 0.83 | 1.31 | 0.58 |
| 418 | CH60_HUMAN | 60 kDa heat shock protein, mitochondrial | P10809 | 0.95 |  | 1.06 |
| 419 | CHCH7_HUMAN | Coiled-coil-helix-coiled-coil-helix domain-containing protein 7 | Q9BUK0 |  | 1.87 | 1.61 |
| 420 | CHDH_HUMAN | Choline dehydrogenase, mitochondrial | Q8NE62 | 0.87 | 0.80 | 0.80 |
| 421 | CHIP_HUMAN | E3 ubiquitin-protein ligase CHIP | Q9UNE7 |  | 0.56 | 0.72 |
| 422 | CHM2A_HUMAN | Charged multivesicular body protein 2a | O43633 | 0.92 |  | 1.03 |
| 423 | CHM4B_HUMAN | Charged multivesicular body protein 4b | Q9H444 | 1.11 | 1.11 | 0.92 |
| 424 | CHMP3_HUMAN | Charged multivesicular body protein 3 | Q9Y3E7 |  | 3.34 | 0.98 |
| 425 | CHMP5_HUMAN | Charged multivesicular body protein 5 | Q9NZZ3 | 0.90 | 1.94 | 0.89 |
| 426 | CHMP6_HUMAN | Charged multivesicular body protein 6 | Q96FZ7 | 0.39 |  | 1.36 |
| 427 | CHP1_HUMAN | Calcineurin B homologous protein 1 | Q99653 | 0.95 | 1.28 | 0.74 |
| 428 | CI064_HUMAN | UPF0553 protein C9orf64 | Q5T6V5 |  | 1.71 | 1.00 |
| 429 | CIA30_HUMAN | Complex I intermediate-associated protein 30, mitochondrial | Q9Y375 |  | 1.26 | 1.04 |
| 430 | CILP1_HUMAN | Cartilage intermediate layer protein 1 | O75339 | 0.19 | 0.81 | 0.77 |
| 431 | CIP4_HUMAN | Cdc42-interacting protein 4 | Q15642 |  | 0.52 | 1.04 |
| 432 | CIRBP_HUMAN | Cold-inducible RNA-binding protein | Q14011 | 0.72 | 1.12 | 0.76 |
| 433 | CISD1_HUMAN | CDGSH iron-sulfur domain-containing protein 1 | Q9NZ45 | 0.95 | 1.28 | 0.58 |
| 434 | CISD3_HUMAN | CDGSH iron-sulfur domain-containing protein 3, mitochondrial | P0C7P0 |  |  |  |
| 435 | CISY_HUMAN | Citrate synthase, mitochondrial | O75390 |  |  | 1.04 |
| 436 | CJ071_HUMAN | Uncharacterized protein C10orf71 | Q711Q0 | 1.10 | 0.77 |  |
| 437 | CK054_HUMAN | Ester hydrolase C11orf54 | Q9H0W9 |  | 1.04 | 0.92 |
| 438 | CK5P3_HUMAN | CDK5 regulatory subunit-associated protein 3 | Q96JB5 | 1.20 | 0.58 | 0.96 |
| 439 | CKAP4_HUMAN | Cytoskeleton-associated protein 4 | Q07065 | 0.29 |  | 1.00 |
| 440 | CKAP5_HUMAN | Cytoskeleton-associated protein 5 | Q14008 | 2.40 | 1.02 | 0.98 |
| 441 | CLAP1_HUMAN | CLIP-associating protein 1 | Q7Z460 | 1.10 | 1.01 | 1.02 |
| 442 | CLAP2_HUMAN | CLIP-associating protein 2 | O75122 |  |  | 1.30 |
| 443 | CLC14_HUMAN | C-type lectin domain family 14 member A | Q86T13 | 3.22 |  | 0.79 |
| 444 | CLCB_HUMAN | Clathrin light chain B | P09497 | 0.80 | 67.30 | 0.69 |
| 445 | CLCC1_HUMAN | Chloride channel CLIC-like protein 1 | Q96S66 | 3.98 |  | 1.37 |
| 446 | CLGN_HUMAN | Calmegin | O14967 | 0.91 |  | 1.15 |
| 447 | CLH1_HUMAN | Clathrin heavy chain 1 | Q00610 |  |  | 1.13 |
| 448 | CLIC1_HUMAN | Chloride intracellular channel protein 1 | O00299 | 0.79 |  | 1.12 |
| 449 | CLIC2_HUMAN | Chloride intracellular channel protein 2 | O15247 |  | 0.35 | 0.67 |
| 450 | CLIC4_HUMAN | Chloride intracellular channel protein 4 | Q9Y696 | 0.51 |  | 0.99 |
| 451 | CLIC5_HUMAN | Chloride intracellular channel protein 5 | Q9NZA1 | 0.72 |  | 0.86 |
| 452 | CLIP1_HUMAN | CAP-Gly domain-containing linker protein 1 | P30622 | 1.16 | 1.21 | 1.00 |
| 453 | CLPB_HUMAN | Caseinolytic peptidase B protein homolog | Q9H078 | 1.10 | 0.91 | 0.84 |
| 454 | CLPP_HUMAN | ATP-dependent Clp protease proteolytic subunit, mitochondrial | Q16740 |  | 1.33 | 0.85 |
| 455 | CLPT1_HUMAN | Cleft lip and palate transmembrane protein 1 | O96005 | 0.74 | 1.06 | 1.59 |
| 456 | CLPX_HUMAN | ATP-dependent Clp protease ATP-binding subunit clpX-like, mitochondrial | O76031 | 1.63 | 0.61 |  |
| 457 | CLU_HUMAN | Clustered mitochondria protein homolog | O75153 | 1.06 | 1.12 | 1.20 |
| 458 | CLUS_HUMAN | Clusterin | P10909 | 1.10 |  | 1.11 |
| 459 | CLYBL_HUMAN | Citrate lyase subunit beta-like protein, mitochondrial | Q8N0X4 |  |  | 1.04 |
| 460 | CMA1_HUMAN | Chymase | P23946 | 1.13 | 4.29 | 0.72 |
| 461 | CMBL_HUMAN | Carboxymethylenebutenolidase homolog | Q96DG6 | 1.18 | 1.10 | 1.00 |
| 462 | CMC1_HUMAN | Calcium-binding mitochondrial carrier protein Aralar1 | O75746 | 0.67 |  | 1.04 |
| 463 | CMC2_HUMAN | Calcium-binding mitochondrial carrier protein Aralar2 | Q9UJS0 | 1.71 | 1.18 | 1.14 |
| 464 | CMTD1_HUMAN | Catechol O-methyltransferase domain-containing protein 1 | Q86VU5 | 0.48 | 1.29 | 0.64 |
| 465 | CMYA5_HUMAN | Cardiomyopathy-associated protein 5 | Q8N3K9 | 0.82 | 1.11 | 1.47 |
| 466 | CN159_HUMAN | UPF0317 protein C14orf159, mitochondrial | Q7Z3D6 | 0.63 | 1.87 | 1.01 |
| 467 | CN166_HUMAN | UPF0568 protein C14orf166 | Q9Y224 | 0.54 | 0.55 | 1.17 |
| 468 | CN37_HUMAN | 2',3'-cyclic-nucleotide 3'-phosphodiesterase | P09543 | 0.61 | 0.67 | 0.93 |
| 469 | CNBP1_HUMAN | Beta-catenin-interacting protein 1 | Q9NSA3 | 0.79 |  | 0.63 |
| 470 | CNDP2_HUMAN | Cytosolic non-specific dipeptidase | Q96KP4 | 1.20 | 1.06 | 0.82 |
| 471 | CNN1_HUMAN | Calponin-1 | P51911 | 0.95 |  | 1.25 |
| 472 | CNN2_HUMAN | Calponin-2 | Q99439 | 1.16 | 1.85 | 0.83 |
| 473 | CNN3_HUMAN | Calponin-3 | Q15417 | 0.77 |  | 0.76 |
| 474 | CO1A1_HUMAN | Collagen alpha-1(I) chain | P02452 | 1.19 |  | 1.07 |
| 475 | CO1A2_HUMAN | Collagen alpha-2(I) chain | P08123 |  |  | 0.93 |
| 476 | CO2_HUMAN | Complement C2 | P06681 | 0.56 | 1.00 | 1.19 |
| 477 | CO3_HUMAN | Complement C3 | P01024 | 1.87 |  | 1.06 |
| 478 | CO3A1_HUMAN | Collagen alpha-1(III) chain | P02461 | 0.69 |  | 0.93 |
| 479 | CO4A_HUMAN | Complement C4-A | P0C0L4 | 0.98 | 0.57 | 0.65 |
| 480 | CO4A1_HUMAN | Collagen alpha-1(IV) chain | P02462 |  | 2.83 | 1.03 |
| 481 | CO4A2_HUMAN | Collagen alpha-2(IV) chain | P08572 | 0.90 |  | 0.90 |
| 482 | CO4B_HUMAN | Complement C4-B | P0C0L5 | 1.14 | 1.05 | 0.98 |
| 483 | CO5_HUMAN | Complement C5 | P01031 | 0.69 |  | 1.07 |
| 484 | CO6_HUMAN | Complement component C6 | P13671 | 1.41 |  | 0.86 |
| 485 | CO6A1_HUMAN | Collagen alpha-1(VI) chain | P12109 |  |  | 1.06 |
| 486 | CO6A2_HUMAN | Collagen alpha-2(VI) chain | P12110 | 2.00 |  | 0.66 |
| 487 | CO6A3_HUMAN | Collagen alpha-3(VI) chain | P12111 | 0.86 |  | 0.87 |
| 488 | CO6A6_HUMAN | Collagen alpha-6(VI) chain | A6NMZ7 |  |  | 0.99 |
| 489 | CO7_HUMAN | Complement component C7 | P10643 | 0.58 | 1.87 | 1.19 |
| 490 | CO8A_HUMAN | Complement component C8 alpha chain | P07357 |  | 1.72 | 1.03 |
| 491 | CO8A1_HUMAN | Collagen alpha-1(VIII) chain | P27658 | 0.98 |  | 0.71 |
| 492 | CO8B_HUMAN | Complement component C8 beta chain | P07358 | 1.77 | 1.13 | 0.74 |
| 493 | CO8G_HUMAN | Complement component C8 gamma chain | P07360 |  | 0.81 | 0.85 |
| 494 | CO9_HUMAN | Complement component C9 | P02748 | 0.83 | 1.47 | 1.06 |
| 495 | COA1_HUMAN | Cytochrome c oxidase assembly factor 1 homolog | Q9GZY4 | 1.36 |  | 0.67 |
| 496 | COA3_HUMAN | Cytochrome c oxidase assembly factor 3 homolog, mitochondrial | Q9Y2R0 | 0.15 | 1.29 |  |
| 497 | COA6_HUMAN | Cytochrome c oxidase assembly factor 6 homolog | Q5JTJ3 | 0.91 | 1.34 | 0.64 |
| 498 | COASY_HUMAN | Bifunctional coenzyme A synthase | Q13057 | 1.89 | 1.53 | 1.23 |
| 499 | COBL_HUMAN | Protein cordon-bleu | O75128 | 0.50 | 0.85 | 1.13 |
| 500 | COCA1_HUMAN | Collagen alpha-1(XII) chain | Q99715 | 0.61 | 0.67 | 0.90 |
| 501 | COEA1_HUMAN | Collagen alpha-1(XIV) chain | Q05707 | 1.42 | 1.56 | 0.94 |
| 502 | COF1_HUMAN | Cofilin-1 | P23528 | 0.63 | 1.03 | 1.05 |
| 503 | COF2_HUMAN | Cofilin-2 | Q9Y281 | 0.75 |  | 1.13 |
| 504 | COFA1_HUMAN | Collagen alpha-1(XV) chain | P39059 |  |  | 1.05 |
| 505 | COIA1_HUMAN | Collagen alpha-1(XVIII) chain | P39060 | 0.92 | 1.49 | 1.22 |
| 506 | COMD1_HUMAN | COMM domain-containing protein 1 | Q8N668 | 0.96 | 1.34 | 1.18 |
| 507 | COMD4_HUMAN | COMM domain-containing protein 4 | Q9H0A8 | 1.36 |  | 1.40 |
| 508 | COMP_HUMAN | Cartilage oligomeric matrix protein | P49747 | 0.54 | 1.79 | 0.78 |
| 509 | COMT_HUMAN | Catechol O-methyltransferase | P21964 | 1.08 | 0.69 | 1.23 |
| 510 | COPA_HUMAN | Coatomer subunit alpha | P53621 | 1.29 | 0.98 | 1.23 |
| 511 | COPB_HUMAN | Coatomer subunit beta | P53618 | 1.06 | 0.50 | 0.38 |
| 512 | COPB2_HUMAN | Coatomer subunit beta' | P35606 | 1.10 | 0.82 | 1.04 |
| 513 | COPD_HUMAN | Coatomer subunit delta | P48444 | 1.07 | 1.14 | 0.87 |
| 514 | COPE_HUMAN | Coatomer subunit epsilon | O14579 | 1.27 | 0.87 | 1.10 |
| 515 | COPG1_HUMAN | Coatomer subunit gamma-1 | Q9Y678 | 0.95 | 0.67 | 0.77 |
| 516 | COPG2_HUMAN | Coatomer subunit gamma-2 | Q9UBF2 | 0.93 |  | 0.52 |
| 517 | COQ3_HUMAN | Hexaprenyldihydroxybenzoate methyltransferase, mitochondrial | Q9NZJ6 | 0.78 |  |  |
| 518 | COQ5_HUMAN | 2-methoxy-6-polyprenyl-1,4-benzoquinol methylase, mitochondrial | Q5HYK3 | 0.42 |  | 1.14 |
| 519 | COQ6_HUMAN | Ubiquinone biosynthesis monooxygenase COQ6 | Q9Y2Z9 | 1.58 | 1.34 | 1.11 |
| 520 | COQ7_HUMAN | Ubiquinone biosynthesis protein COQ7 homolog | Q99807 | 0.80 | 0.31 | 0.75 |
| 521 | COQ9_HUMAN | Ubiquinone biosynthesis protein COQ9, mitochondrial | O75208 |  | 1.42 | 1.02 |
| 522 | COR1A_HUMAN | Coronin-1A | P31146 | 1.32 | 0.41 | 2.68 |
| 523 | COR1C_HUMAN | Coronin-1C | Q9ULV4 | 0.86 | 0.94 | 0.79 |
| 524 | CORO6_HUMAN | Coronin-6 | Q6QEF8 | 0.70 | 1.21 | 0.83 |
| 525 | COSA1_HUMAN | Collagen alpha-1(XXVIII) chain | Q2UY09 |  | 1.34 | 1.04 |
| 526 | COTL1_HUMAN | Coactosin-like protein | Q14019 | 0.86 |  | 0.62 |
| 527 | COX1_HUMAN | Cytochrome c oxidase subunit 1 | P00395 | 0.86 | 1.24 | 0.92 |
| 528 | COX11_HUMAN | Cytochrome c oxidase assembly protein COX11, mitochondrial | Q9Y6N1 | 1.26 |  | 1.20 |
| 529 | COX14_HUMAN | Cytochrome c oxidase assembly protein COX14 | Q96I36 | 0.15 |  | 1.05 |
| 530 | COX15_HUMAN | Cytochrome c oxidase assembly protein COX15 homolog | Q7KZN9 | 1.11 | 1.03 | 1.17 |
| 531 | COX2_HUMAN | Cytochrome c oxidase subunit 2 | P00403 | 1.09 | 0.93 | 1.06 |
| 532 | COX20_HUMAN | Cytochrome c oxidase protein 20 homolog | Q5RI15 | 1.31 | 1.14 | 0.92 |
| 533 | COX3_HUMAN | Cytochrome c oxidase subunit 3 | P00414 | 1.75 |  | 0.89 |
| 534 | COX41_HUMAN | Cytochrome c oxidase subunit 4 isoform 1, mitochondrial | P13073 | 0.36 | 1.79 | 1.09 |
| 535 | COX5A_HUMAN | Cytochrome c oxidase subunit 5A, mitochondrial | P20674 | 2.00 | 0.79 | 1.09 |
| 536 | COX5B_HUMAN | Cytochrome c oxidase subunit 5B, mitochondrial | P10606 | 1.27 | 1.16 | 1.04 |
| 537 | COX6C_HUMAN | Cytochrome c oxidase subunit 6C | P09669 | 0.96 | 0.92 | 1.30 |
| 538 | COX7B_HUMAN | Cytochrome c oxidase subunit 7B, mitochondrial | P24311 | 0.17 | 2.15 | 1.00 |
| 539 | COX7C_HUMAN | Cytochrome c oxidase subunit 7C, mitochondrial | P15954 | 1.19 | 1.24 | 1.00 |
| 540 | COX7R_HUMAN | Cytochrome c oxidase subunit 7A-related protein, mitochondrial | O14548 | 0.90 | 0.76 | 1.10 |
| 541 | COX8A_HUMAN | Cytochrome c oxidase subunit 8A, mitochondrial | P10176 | 0.50 | 0.22 | 1.42 |
| 542 | COXM1_HUMAN | COX assembly mitochondrial protein homolog | Q7Z7K0 | 1.27 | 1.05 | 1.13 |
| 543 | CP013_HUMAN | UPF0585 protein C16orf13 | Q96S19 |  | 0.33 | 0.99 |
| 544 | CP2J2_HUMAN | Cytochrome P450 2J2 | P51589 | 1.01 |  | 0.81 |
| 545 | CPIN1_HUMAN | Anamorsin | Q6FI81 | 1.45 |  | 0.88 |
| 546 | CPNE1_HUMAN | Copine-1 | Q99829 | 1.84 | 0.79 | 1.20 |
| 547 | CPNE3_HUMAN | Copine-3 | O75131 |  |  | 1.10 |
| 548 | CPNS1_HUMAN | Calpain small subunit 1 | P04632 |  |  | 0.82 |
| 549 | CPPED_HUMAN | Serine/threonine-protein phosphatase CPPED1 | Q9BRF8 | 1.31 | 0.71 | 0.88 |
| 550 | CPSF5_HUMAN | Cleavage and polyadenylation specificity factor subunit 5 | O43809 | 0.95 | 1.67 | 1.04 |
| 551 | CPT1A_HUMAN | Carnitine O-palmitoyltransferase 1, liver isoform | P50416 |  | 1.24 | 0.93 |
| 552 | CPT1B_HUMAN | Carnitine O-palmitoyltransferase 1, muscle isoform | Q92523 | 0.72 |  | 1.18 |
| 553 | CPT2_HUMAN | Carnitine O-palmitoyltransferase 2, mitochondrial | P23786 | 0.52 | 0.69 | 1.14 |
| 554 | CPTP_HUMAN | Ceramide-1-phosphate transfer protein | Q5TA50 |  | 0.67 | 1.72 |
| 555 | CPVL_HUMAN | Probable serine carboxypeptidase CPVL | Q9H3G5 | 1.28 |  | 0.69 |
| 556 | CQ10A_HUMAN | Coenzyme Q-binding protein COQ10 homolog A, mitochondrial | Q96MF6 | 1.57 | 0.65 | 1.16 |
| 557 | CQ10B_HUMAN | Coenzyme Q-binding protein COQ10 homolog B, mitochondrial | Q9H8M1 | 0.66 |  | 1.11 |
| 558 | CRADD_HUMAN | Death domain-containing protein CRADD | P78560 | 1.66 | 0.90 | 0.86 |
| 559 | CRBN_HUMAN | Protein cereblon | Q96SW2 | 0.58 |  | 1.04 |
| 560 | CREL1_HUMAN | Cysteine-rich with EGF-like domain protein 1 | Q96HD1 |  | 0.90 | 0.77 |
| 561 | CRIP2_HUMAN | Cysteine-rich protein 2 | P52943 | 0.72 | 1.17 | 0.86 |
| 562 | CRK_HUMAN | Adapter molecule crk | P46108 | 1.01 | 1.29 | 1.11 |
| 563 | CRKL_HUMAN | Crk-like protein | P46109 | 0.30 | 1.42 | 1.19 |
| 564 | CRYAB_HUMAN | Alpha-crystallin B chain | P02511 |  | 0.67 | 0.48 |
| 565 | CRYL1_HUMAN | Lambda-crystallin homolog | Q9Y2S2 | 0.59 | 0.75 | 1.00 |
| 566 | CRYM_HUMAN | Ketimine reductase mu-crystallin | Q14894 |  | 0.90 | 1.17 |
| 567 | CSDE1_HUMAN | Cold shock domain-containing protein E1 | O75534 | 0.77 | 1.17 | 1.19 |
| 568 | CSK_HUMAN | Tyrosine-protein kinase CSK | P41240 | 0.95 |  | 0.73 |
| 569 | CSK21_HUMAN | Casein kinase II subunit alpha | P68400 | 0.40 | 0.75 | 0.91 |
| 570 | CSK2B_HUMAN | Casein kinase II subunit beta | P67870 | 0.91 | 0.94 | 1.16 |
| 571 | CSN1_HUMAN | COP9 signalosome complex subunit 1 | Q13098 | 0.92 | 0.92 | 1.06 |
| 572 | CSN2_HUMAN | COP9 signalosome complex subunit 2 | P61201 | 0.95 | 1.13 | 1.25 |
| 573 | CSN3_HUMAN | COP9 signalosome complex subunit 3 | Q9UNS2 | 3.25 | 0.99 | 1.15 |
| 574 | CSN4_HUMAN | COP9 signalosome complex subunit 4 | Q9BT78 | 0.93 | 0.91 | 0.72 |
| 575 | CSN5_HUMAN | COP9 signalosome complex subunit 5 | Q92905 | 1.21 | 1.15 |  |
| 576 | CSN6_HUMAN | COP9 signalosome complex subunit 6 | Q7L5N1 | 1.74 | 1.56 | 0.93 |
| 577 | CSN7A_HUMAN | COP9 signalosome complex subunit 7a | Q9UBW8 | 1.20 | 0.52 | 1.16 |
| 578 | CSN8_HUMAN | COP9 signalosome complex subunit 8 | Q99627 | 0.72 | 0.75 | 0.69 |
| 579 | CSPG2_HUMAN | Versican core protein | P13611 | 0.80 | 1.06 | 1.01 |
| 580 | CSPG4_HUMAN | Chondroitin sulfate proteoglycan 4 | Q6UVK1 | 0.52 | 0.29 | 1.13 |
| 581 | CSRP1_HUMAN | Cysteine and glycine-rich protein 1 | P21291 | 0.61 | 1.02 | 0.76 |
| 582 | CSRP3_HUMAN | Cysteine and glycine-rich protein 3 | P50461 | 0.79 | 0.92 | 1.00 |
| 583 | CSTF2_HUMAN | Cleavage stimulation factor subunit 2 | P33240 |  | 1.28 | 0.92 |
| 584 | CTBP1_HUMAN | C-terminal-binding protein 1 | Q13363 | 0.72 |  | 1.45 |
| 585 | CTGF_HUMAN | Connective tissue growth factor | P29279 | 0.68 |  | 1.20 |
| 586 | CTNA1_HUMAN | Catenin alpha-1 | P35221 | 1.16 |  | 0.77 |
| 587 | CTNA3_HUMAN | Catenin alpha-3 | Q9UI47 | 0.86 | 2.29 | 0.99 |
| 588 | CTNB1_HUMAN | Catenin beta-1 | P35222 |  | 0.99 | 0.93 |
| 589 | CTND1_HUMAN | Catenin delta-1 | O60716 | 0.69 | 0.78 | 0.78 |
| 590 | CUL1_HUMAN | Cullin-1 | Q13616 | 0.70 |  | 0.64 |
| 591 | CUL2_HUMAN | Cullin-2 | Q13617 |  |  | 0.81 |
| 592 | CUL3_HUMAN | Cullin-3 | Q13618 | 0.12 | 1.25 | 0.98 |
| 593 | CUL4A_HUMAN | Cullin-4A | Q13619 | 0.79 | 0.72 | 1.07 |
| 594 | CUL5_HUMAN | Cullin-5 | Q93034 | 2.86 | 0.44 | 1.02 |
| 595 | CUTA_HUMAN | Protein CutA | O60888 | 0.77 | 0.64 | 1.12 |
| 596 | CX6B1_HUMAN | Cytochrome c oxidase subunit 6B1 | P14854 | 6.31 | 1.03 | 1.00 |
| 597 | CX7A1_HUMAN | Cytochrome c oxidase subunit 7A1, mitochondrial | P24310 | 1.02 | 1.26 | 0.99 |
| 598 | CX7A2_HUMAN | Cytochrome c oxidase subunit 7A2, mitochondrial | P14406 | 0.27 | 0.86 | 1.24 |
| 599 | CXA1_HUMAN | Gap junction alpha-1 protein | P17302 | 0.91 | 1.13 | 0.63 |
| 600 | CY1_HUMAN | Cytochrome c1, heme protein, mitochondrial | P08574 | 0.31 |  | 0.65 |
| 601 | CYB_HUMAN | Cytochrome b | P00156 | 1.27 | 0.64 | 1.22 |
| 602 | CYB5_HUMAN | Cytochrome b5 | P00167 | 0.31 | 1.02 | 1.07 |
| 603 | CYB5B_HUMAN | Cytochrome b5 type B | O43169 | 0.58 | 1.67 | 0.65 |
| 604 | CYBP_HUMAN | Calcyclin-binding protein | Q9HB71 | 1.05 | 0.72 | 0.71 |
| 605 | CYBR1_HUMAN | Cytochrome b reductase 1 | Q53TN4 | 0.43 | 0.57 | 0.86 |
| 606 | CYC_HUMAN | Cytochrome c | P99999 | 1.33 | 0.95 | 1.16 |
| 607 | CYFP1_HUMAN | Cytoplasmic FMR1-interacting protein 1 | Q7L576 | 1.66 | 1.15 | 1.29 |
| 608 | CYGB_HUMAN | Cytoglobin | Q8WWM9 | 1.66 | 0.96 | 1.18 |
| 609 | CYTB_HUMAN | Cystatin-B | P04080 |  | 1.49 | 2.28 |
| 610 | CYTC_HUMAN | Cystatin-C | P01034 | 1.28 | 0.79 | 1.02 |
| 611 | D2HDH_HUMAN | D-2-hydroxyglutarate dehydrogenase, mitochondrial | Q8N465 | 0.63 | 0.69 | 0.31 |
| 612 | D39U1_HUMAN | Epimerase family protein SDR39U1 | Q9NRG7 | 0.77 | 0.85 | 0.91 |
| 613 | DAD1_HUMAN | Dolichyl-diphosphooligosaccharide--protein glycosyltransferase subunit DAD1 | P61803 |  | 0.89 | 1.29 |
| 614 | DAG1_HUMAN | Dystroglycan | Q14118 |  | 0.72 | 1.09 |
| 615 | DAPK3_HUMAN | Death-associated protein kinase 3 | O43293 |  | 2.01 | 2.50 |
| 616 | DBLOH_HUMAN | Diablo homolog, mitochondrial | Q9NR28 | 0.82 | 1.16 | 1.04 |
| 617 | DBNL_HUMAN | Drebrin-like protein | Q9UJU6 | 1.98 | 1.04 | 0.98 |
| 618 | DC1I1_HUMAN | Cytoplasmic dynein 1 intermediate chain 1 | O14576 | 0.22 | 2.56 | 0.98 |
| 619 | DC1I2_HUMAN | Cytoplasmic dynein 1 intermediate chain 2 | Q13409 | 0.08 | 1.15 | 1.04 |
| 620 | DC1L1_HUMAN | Cytoplasmic dynein 1 light intermediate chain 1 | Q9Y6G9 | 1.10 | 0.58 | 1.11 |
| 621 | DC1L2_HUMAN | Cytoplasmic dynein 1 light intermediate chain 2 | O43237 | 1.00 |  | 1.00 |
| 622 | DCAF8_HUMAN | DDB1- and CUL4-associated factor 8 | Q5TAQ9 |  | 1.02 | 1.20 |
| 623 | DCAKD_HUMAN | Dephospho-CoA kinase domain-containing protein | Q8WVC6 | 0.86 |  | 0.76 |
| 624 | DCLK1_HUMAN | Serine/threonine-protein kinase DCLK1 | O15075 | 1.57 |  | 1.25 |
| 625 | DCMC_HUMAN | Malonyl-CoA decarboxylase, mitochondrial | O95822 | 0.90 | 0.83 | 1.21 |
| 626 | DCNL1_HUMAN | DCN1-like protein 1 | Q96GG9 | 0.26 | 0.99 | 1.44 |
| 627 | DCPS_HUMAN | m7GpppX diphosphatase | Q96C86 |  | 0.99 | 1.62 |
| 628 | DCTN1_HUMAN | Dynactin subunit 1 | Q14203 | 0.39 | 1.94 | 1.05 |
| 629 | DCTN2_HUMAN | Dynactin subunit 2 | Q13561 | 0.39 |  | 0.97 |
| 630 | DCTN4_HUMAN | Dynactin subunit 4 | Q9UJW0 | 1.09 | 0.45 | 0.77 |
| 631 | DCXR_HUMAN | L-xylulose reductase | Q7Z4W1 |  | 1.72 | 0.83 |
| 632 | DD19A_HUMAN | ATP-dependent RNA helicase DDX19A | Q9NUU7 | 0.97 | 0.58 | 1.04 |
| 633 | DD19B_HUMAN | ATP-dependent RNA helicase DDX19B | Q9UMR2 | 0.45 |  | 1.04 |
| 634 | DDAH1_HUMAN | N(G),N(G)-dimethylarginine dimethylaminohydrolase 1 | O94760 | 0.91 | 1.34 | 0.97 |
| 635 | DDAH2_HUMAN | N(G),N(G)-dimethylarginine dimethylaminohydrolase 2 | O95865 | 1.19 | 1.36 | 0.99 |
| 636 | DDB1_HUMAN | DNA damage-binding protein 1 | Q16531 | 2.40 | 1.22 | 0.97 |
| 637 | DDRGK_HUMAN | DDRGK domain-containing protein 1 | Q96HY6 | 0.44 | 1.22 | 1.61 |
| 638 | DDX1_HUMAN | ATP-dependent RNA helicase DDX1 | Q92499 |  | 1.06 | 0.85 |
| 639 | DDX17_HUMAN | Probable ATP-dependent RNA helicase DDX17 | Q92841 |  | 0.77 |  |
| 640 | DDX3X_HUMAN | ATP-dependent RNA helicase DDX3X | O00571 |  | 1.13 | 1.14 |
| 641 | DDX42_HUMAN | ATP-dependent RNA helicase DDX42 | Q86XP3 |  | 0.17 | 0.78 |
| 642 | DDX5_HUMAN | Probable ATP-dependent RNA helicase DDX5 | P17844 | 1.17 | 1.64 | 1.00 |
| 643 | DDX6_HUMAN | Probable ATP-dependent RNA helicase DDX6 | P26196 | 1.42 | 1.15 | 0.72 |
| 644 | DECR_HUMAN | 2,4-dienoyl-CoA reductase, mitochondrial | Q16698 | 2.40 |  | 0.93 |
| 645 | DEK_HUMAN | Protein DEK | P35659 |  | 0.79 | 0.89 |
| 646 | DERL1_HUMAN | Derlin-1 | Q9BUN8 |  | 0.51 | 0.09 |
| 647 | DERM_HUMAN | Dermatopontin | Q07507 | 0.98 | 1.49 | 0.65 |
| 648 | DESM_HUMAN | Desmin | P17661 | 0.38 | 0.83 | 1.28 |
| 649 | DESP_HUMAN | Desmoplakin | P15924 | 1.27 | 0.76 | 0.85 |
| 650 | DEST_HUMAN | Destrin | P60981 | 1.80 | 0.72 | 0.66 |
| 651 | DFFA_HUMAN | DNA fragmentation factor subunit alpha | O00273 | 1.85 | 0.90 | 0.88 |
| 652 | DHB11_HUMAN | Estradiol 17-beta-dehydrogenase 11 | Q8NBQ5 | 1.27 | 1.51 | 1.39 |
| 653 | DHB12_HUMAN | Estradiol 17-beta-dehydrogenase 12 | Q53GQ0 | 1.13 | 1.17 | 1.09 |
| 654 | DHB4_HUMAN | Peroxisomal multifunctional enzyme type 2 | P51659 |  |  | 1.01 |
| 655 | DHB8_HUMAN | Estradiol 17-beta-dehydrogenase 8 | Q92506 | 6.19 | 1.08 | 0.88 |
| 656 | DHE3_HUMAN | Glutamate dehydrogenase 1, mitochondrial | P00367 | 1.01 | 1.09 | 1.02 |
| 657 | DHPR_HUMAN | Dihydropteridine reductase | P09417 | 0.84 | 1.28 | 1.07 |
| 658 | DHRS4_HUMAN | Dehydrogenase/reductase SDR family member 4 | Q9BTZ2 |  | 1.14 | 1.06 |
| 659 | DHRS7_HUMAN | Dehydrogenase/reductase SDR family member 7 | Q9Y394 | 0.68 |  |  |
| 660 | DHSD_HUMAN | Succinate dehydrogenase [ubiquinone] cytochrome b small subunit, mitochondrial | O14521 | 0.97 | 1.31 | 0.72 |
| 661 | DHSO_HUMAN | Sorbitol dehydrogenase | Q00796 | 1.15 | 0.51 | 0.80 |
| 662 | DHTK1_HUMAN | Probable 2-oxoglutarate dehydrogenase E1 component DHKTD1, mitochondrial | Q96HY7 | 4.29 | 1.46 | 0.69 |
| 663 | DHX15_HUMAN | Putative pre-mRNA-splicing factor ATP-dependent RNA helicase DHX15 | O43143 |  | 0.87 | 1.01 |
| 664 | DHX9_HUMAN | ATP-dependent RNA helicase A | Q08211 |  |  | 0.95 |
| 665 | DIC_HUMAN | Mitochondrial dicarboxylate carrier | Q9UBX3 | 1.53 |  | 1.03 |
| 666 | DJB11_HUMAN | DnaJ homolog subfamily B member 11 | Q9UBS4 | 0.73 | 0.96 | 1.13 |
| 667 | DJC10_HUMAN | DnaJ homolog subfamily C member 10 | Q8IXB1 |  | 1.03 | 0.90 |
| 668 | DJC11_HUMAN | DnaJ homolog subfamily C member 11 | Q9NVH1 | 1.38 | 1.14 | 0.98 |
| 669 | DJC13_HUMAN | DnaJ homolog subfamily C member 13 | O75165 | 1.10 |  | 0.68 |
| 670 | DJC30_HUMAN | DnaJ homolog subfamily C member 30 | Q96LL9 |  | 0.66 | 1.32 |
| 671 | DKK3_HUMAN | Dickkopf-related protein 3 | Q9UBP4 | 0.07 | 1.33 | 0.76 |
| 672 | DLDH_HUMAN | Dihydrolipoyl dehydrogenase, mitochondrial | P09622 |  | 5.92 | 1.38 |
| 673 | DLRB1_HUMAN | Dynein light chain roadblock-type 1 | Q9NP97 | 1.29 | 1.54 | 1.17 |
| 674 | DMD_HUMAN | Dystrophin | P11532 |  | 2.01 | 1.13 |
| 675 | DMPK_HUMAN | Myotonin-protein kinase | Q09013 | 0.98 | 1.34 | 1.07 |
| 676 | DNJA1_HUMAN | DnaJ homolog subfamily A member 1 | P31689 | 0.51 |  | 1.47 |
| 677 | DNJA2_HUMAN | DnaJ homolog subfamily A member 2 | O60884 | 1.29 |  | 0.94 |
| 678 | DNJA3_HUMAN | DnaJ homolog subfamily A member 3, mitochondrial | Q96EY1 | 0.46 | 1.51 | 0.85 |
| 679 | DNJA4_HUMAN | DnaJ homolog subfamily A member 4 | Q8WW22 | 0.74 | 1.22 | 0.88 |
| 680 | DNJB4_HUMAN | DnaJ homolog subfamily B member 4 | Q9UDY4 | 1.27 | 1.12 | 1.03 |
| 681 | DNJB6_HUMAN | DnaJ homolog subfamily B member 6 | O75190 | 0.90 | 0.97 | 0.88 |
| 682 | DNJC3_HUMAN | DnaJ homolog subfamily C member 3 | Q13217 | 1.07 | 0.85 | 1.18 |
| 683 | DNJC7_HUMAN | DnaJ homolog subfamily C member 7 | Q99615 | 1.12 |  | 1.09 |
| 684 | DNM1L_HUMAN | Dynamin-1-like protein | O00429 | 0.72 | 1.20 | 0.88 |
| 685 | DNPEP_HUMAN | Aspartyl aminopeptidase | Q9ULA0 | 0.69 | 1.51 | 0.98 |
| 686 | DNPH1_HUMAN | 2'-deoxynucleoside 5'-phosphate N-hydrolase 1 | O43598 | 0.75 | 1.42 | 0.80 |
| 687 | DOPD_HUMAN | D-dopachrome decarboxylase | P30046 | 0.86 |  | 1.04 |
| 688 | DP13A_HUMAN | DCC-interacting protein 13-alpha | Q9UKG1 | 0.95 | 1.49 | 0.89 |
| 689 | DPH5_HUMAN | Diphthine synthase | Q9H2P9 |  |  | 0.75 |
| 690 | DPM1_HUMAN | Dolichol-phosphate mannosyltransferase subunit 1 | O60762 | 0.37 |  | 0.57 |
| 691 | DPP2_HUMAN | Dipeptidyl peptidase 2 | Q9UHL4 | 0.94 | 0.86 | 0.81 |
| 692 | DPP3_HUMAN | Dipeptidyl peptidase 3 | Q9NY33 | 1.06 | 1.20 | 0.36 |
| 693 | DPP9_HUMAN | Dipeptidyl peptidase 9 | Q86TI2 |  | 0.94 | 0.93 |
| 694 | DPY30_HUMAN | Protein dpy-30 homolog | Q9C005 | 3.50 |  |  |
| 695 | DPYL2_HUMAN | Dihydropyrimidinase-related protein 2 | Q16555 | 1.82 | 1.07 | 1.06 |
| 696 | DPYL3_HUMAN | Dihydropyrimidinase-related protein 3 | Q14195 | 1.39 | 1.14 | 1.16 |
| 697 | DRA_HUMAN | HLA class II histocompatibility antigen, DR alpha chain | P01903 |  | 2.17 | 0.99 |
| 698 | DREB_HUMAN | Drebrin | Q16643 | 0.18 | 0.95 | 1.12 |
| 699 | DRG2_HUMAN | Developmentally-regulated GTP-binding protein 2 | P55039 | 0.98 |  | 0.28 |
| 700 | DRS7B_HUMAN | Dehydrogenase/reductase SDR family member 7B | Q6IAN0 | 0.80 | 1.24 | 1.06 |
| 701 | DRS7C_HUMAN | Dehydrogenase/reductase SDR family member 7C | A6NNS2 | 0.73 | 1.14 | 0.74 |
| 702 | DSC2_HUMAN | Desmocollin-2 | Q02487 | 1.60 | 0.80 | 1.22 |
| 703 | DSG2_HUMAN | Desmoglein-2 | Q14126 | 103.75 |  | 0.89 |
| 704 | DTNA_HUMAN | Dystrobrevin alpha | Q9Y4J8 | 0.53 | 1.82 | 0.87 |
| 705 | DUS27_HUMAN | Inactive dual specificity phosphatase 27 | Q5VZP5 | 0.65 |  | 1.04 |
| 706 | DUS3_HUMAN | Dual specificity protein phosphatase 3 | P51452 | 0.99 | 0.72 | 0.98 |
| 707 | DUT_HUMAN | Deoxyuridine 5'-triphosphate nucleotidohydrolase, mitochondrial | P33316 | 0.46 | 1.38 | 1.30 |
| 708 | DX39B_HUMAN | Spliceosome RNA helicase DDX39B | Q13838 | 4.13 | 0.74 | 0.68 |
| 709 | DYHC1_HUMAN | Cytoplasmic dynein 1 heavy chain 1 | Q14204 | 0.95 |  | 0.91 |
| 710 | DYL1_HUMAN | Dynein light chain 1, cytoplasmic | P63167 | 0.80 | 0.69 | 1.21 |
| 711 | DYL2_HUMAN | Dynein light chain 2, cytoplasmic | Q96FJ2 |  | 0.94 | 1.04 |
| 712 | DYLT1_HUMAN | Dynein light chain Tctex-type 1 | P63172 | 0.29 |  |  |
| 713 | DYN2_HUMAN | Dynamin-2 | P50570 | 1.51 | 1.11 | 0.89 |
| 714 | DYN3_HUMAN | Dynamin-3 | Q9UQ16 | 1.22 |  | 0.92 |
| 715 | DYSF_HUMAN | Dysferlin | O75923 | 1.22 |  | 0.87 |
| 716 | DYST_HUMAN | Dystonin | Q03001 | 1.12 | 1.19 | 0.96 |
| 717 | E41L2_HUMAN | Band 4.1-like protein 2 | O43491 | 1.22 |  | 1.00 |
| 718 | ECH1_HUMAN | Delta(3,5)-Delta(2,4)-dienoyl-CoA isomerase, mitochondrial | Q13011 | 1.20 |  | 1.10 |
| 719 | ECHA_HUMAN | Trifunctional enzyme subunit alpha, mitochondrial | P40939 | 1.10 |  | 0.89 |
| 720 | ECHB_HUMAN | Trifunctional enzyme subunit beta, mitochondrial | P55084 | 0.79 |  | 1.04 |
| 721 | ECHD1_HUMAN | Ethylmalonyl-CoA decarboxylase | Q9NTX5 | 2.88 | 1.49 | 1.08 |
| 722 | ECHD2_HUMAN | Enoyl-CoA hydratase domain-containing protein 2, mitochondrial | Q86YB7 | 0.99 | 1.05 | 1.07 |
| 723 | ECHD3_HUMAN | Enoyl-CoA hydratase domain-containing protein 3, mitochondrial | Q96DC8 | 1.04 | 0.96 | 0.94 |
| 724 | ECHM_HUMAN | Enoyl-CoA hydratase, mitochondrial | P30084 |  |  | 0.88 |
| 725 | ECHP_HUMAN | Peroxisomal bifunctional enzyme | Q08426 | 1.43 | 1.03 | 1.04 |
| 726 | ECI1_HUMAN | Enoyl-CoA delta isomerase 1, mitochondrial | P42126 |  | 1.20 | 1.03 |
| 727 | ECI2_HUMAN | Enoyl-CoA delta isomerase 2, mitochondrial | O75521 | 0.67 | 1.66 | 1.03 |
| 728 | ECM29_HUMAN | Proteasome-associated protein ECM29 homolog | Q5VYK3 |  | 1.45 | 0.97 |
| 729 | ECSIT_HUMAN | Evolutionarily conserved signaling intermediate in Toll pathway, mitochondrial | Q9BQ95 | 1.00 | 1.41 | 1.32 |
| 730 | EEA1_HUMAN | Early endosome antigen 1 | Q15075 | 1.09 | 1.84 | 0.99 |
| 731 | EF1A1_HUMAN | Elongation factor 1-alpha 1 | P68104 | 0.69 | 1.36 | 0.73 |
| 732 | EF1A2_HUMAN | Elongation factor 1-alpha 2 | Q05639 | 0.98 |  | 1.10 |
| 733 | EF1B_HUMAN | Elongation factor 1-beta | P24534 | 1.02 | 1.34 | 0.69 |
| 734 | EF1D_HUMAN | Elongation factor 1-delta | P29692 | 0.94 | 1.34 | 0.93 |
| 735 | EF1G_HUMAN | Elongation factor 1-gamma | P26641 | 0.65 | 0.86 | 1.25 |
| 736 | EF2_HUMAN | Elongation factor 2 | P13639 | 0.89 |  | 0.76 |
| 737 | EFGM_HUMAN | Elongation factor G, mitochondrial | Q96RP9 | 1.32 | 0.78 | 1.13 |
| 738 | EFR3A_HUMAN | Protein EFR3 homolog A | Q14156 | 0.61 |  | 0.86 |
| 739 | EFTS_HUMAN | Elongation factor Ts, mitochondrial | P43897 | 0.38 | 2.31 | 1.00 |
| 740 | EFTU_HUMAN | Elongation factor Tu, mitochondrial | P49411 | 1.51 |  | 1.16 |
| 741 | EGLN_HUMAN | Endoglin | P17813 | 2.17 | 0.84 |  |
| 742 | EHD1_HUMAN | EH domain-containing protein 1 | Q9H4M9 | 0.70 | 1.46 | 0.87 |
| 743 | EHD2_HUMAN | EH domain-containing protein 2 | Q9NZN4 | 0.82 |  | 0.97 |
| 744 | EHD3_HUMAN | EH domain-containing protein 3 | Q9NZN3 | 1.01 | 1.20 | 0.80 |
| 745 | EHD4_HUMAN | EH domain-containing protein 4 | Q9H223 | 0.61 | 1.36 | 1.03 |
| 746 | EI2BB_HUMAN | Translation initiation factor eIF-2B subunit beta | P49770 |  | 1.07 | 0.55 |
| 747 | EI2BE_HUMAN | Translation initiation factor eIF-2B subunit epsilon | Q13144 |  | 1.07 |  |
| 748 | EI2BG_HUMAN | Translation initiation factor eIF-2B subunit gamma | Q9NR50 |  | 0.61 | 1.22 |
| 749 | EIF1B_HUMAN | Eukaryotic translation initiation factor 1b | O60739 | 1.38 | 0.53 | 0.74 |
| 750 | EIF2A_HUMAN | Eukaryotic translation initiation factor 2A | Q9BY44 | 0.84 | 0.34 |  |
| 751 | EIF3A_HUMAN | Eukaryotic translation initiation factor 3 subunit A | Q14152 | 1.22 | 1.15 | 1.53 |
| 752 | EIF3B_HUMAN | Eukaryotic translation initiation factor 3 subunit B | P55884 |  |  | 1.05 |
| 753 | EIF3C_HUMAN | Eukaryotic translation initiation factor 3 subunit C | Q99613 | 1.27 |  | 0.99 |
| 754 | EIF3D_HUMAN | Eukaryotic translation initiation factor 3 subunit D | O15371 | 0.94 | 1.18 | 1.04 |
| 755 | EIF3E_HUMAN | Eukaryotic translation initiation factor 3 subunit E | P60228 | 2.05 | 2.13 | 0.91 |
| 756 | EIF3F_HUMAN | Eukaryotic translation initiation factor 3 subunit F | O00303 | 2.01 | 1.10 | 0.78 |
| 757 | EIF3G_HUMAN | Eukaryotic translation initiation factor 3 subunit G | O75821 | 1.19 | 1.12 | 0.96 |
| 758 | EIF3H_HUMAN | Eukaryotic translation initiation factor 3 subunit H | O15372 | 0.81 |  | 0.81 |
| 759 | EIF3I_HUMAN | Eukaryotic translation initiation factor 3 subunit I | Q13347 | 0.87 | 1.09 | 0.96 |
| 760 | EIF3J_HUMAN | Eukaryotic translation initiation factor 3 subunit J | O75822 |  | 3.28 | 0.97 |
| 761 | EIF3K_HUMAN | Eukaryotic translation initiation factor 3 subunit K | Q9UBQ5 | 1.17 | 0.77 | 0.84 |
| 762 | EIF3L_HUMAN | Eukaryotic translation initiation factor 3 subunit L | Q9Y262 | 0.65 | 1.00 | 1.10 |
| 763 | EIF3M_HUMAN | Eukaryotic translation initiation factor 3 subunit M | Q7L2H7 | 1.14 |  | 1.09 |
| 764 | ELAV1_HUMAN | ELAV-like protein 1 | Q15717 | 2.68 | 0.54 | 0.73 |
| 765 | ELMD2_HUMAN | ELMO domain-containing protein 2 | Q8IZ81 | 0.95 |  | 1.13 |
| 766 | ELOB_HUMAN | Transcription elongation factor B polypeptide 2 | Q15370 | 1.16 |  | 0.96 |
| 767 | ELOC_HUMAN | Transcription elongation factor B polypeptide 1 | Q15369 | 0.08 | 0.61 | 0.65 |
| 768 | EMAL1_HUMAN | Echinoderm microtubule-associated protein-like 1 | O00423 | 0.48 |  | 1.26 |
| 769 | EMAL2_HUMAN | Echinoderm microtubule-associated protein-like 2 | O95834 | 1.03 | 0.72 | 1.04 |
| 770 | EMC1_HUMAN | ER membrane protein complex subunit 1 | Q8N766 | 1.13 | 0.94 | 0.96 |
| 771 | EMC10_HUMAN | ER membrane protein complex subunit 10 | Q5UCC4 | 0.79 |  | 0.79 |
| 772 | EMC2_HUMAN | ER membrane protein complex subunit 2 | Q15006 |  | 1.22 |  |
| 773 | EMC4_HUMAN | ER membrane protein complex subunit 4 | Q5J8M3 | 0.35 | 1.15 | 1.19 |
| 774 | EMC7_HUMAN | ER membrane protein complex subunit 7 | Q9NPA0 |  | 0.72 | 1.10 |
| 775 | EMC8_HUMAN | ER membrane protein complex subunit 8 | O43402 |  | 0.66 | 0.72 |
| 776 | EMD_HUMAN | Emerin | P50402 | 1.09 | 0.54 | 1.25 |
| 777 | EMIL1_HUMAN | EMILIN-1 | Q9Y6C2 | 1.28 | 0.84 | 1.02 |
| 778 | EMIL2_HUMAN | EMILIN-2 | Q9BXX0 |  | 0.73 | 1.87 |
| 779 | EMIL3_HUMAN | EMILIN-3 | Q9NT22 | 1.21 | 1.05 | 0.94 |
| 780 | ENAH_HUMAN | Protein enabled homolog | Q8N8S7 |  | 3.16 | 0.96 |
| 781 | ENDD1_HUMAN | Endonuclease domain-containing 1 protein | O94919 | 0.55 | 0.48 | 0.40 |
| 782 | ENOA_HUMAN | Alpha-enolase | P06733 | 0.78 |  | 0.79 |
| 783 | ENOB_HUMAN | Beta-enolase | P13929 | 1.24 | 1.06 | 1.11 |
| 784 | ENOG_HUMAN | Gamma-enolase | P09104 | 0.51 | 0.96 | 0.83 |
| 785 | ENOPH_HUMAN | Enolase-phosphatase E1 | Q9UHY7 |  | 0.66 | 0.80 |
| 786 | ENPL_HUMAN | Endoplasmin | P14625 | 0.70 |  | 0.87 |
| 787 | ENSA_HUMAN | Alpha-endosulfine | O43768 | 0.97 |  | 0.62 |
| 788 | EP15R_HUMAN | Epidermal growth factor receptor substrate 15-like 1 | Q9UBC2 | 0.94 | 1.36 | 0.98 |
| 789 | EPDR1_HUMAN | Mammalian ependymin-related protein 1 | Q9UM22 | 1.01 | 2.29 | 1.27 |
| 790 | EPM2A_HUMAN | Laforin | O95278 |  | 1.00 | 0.80 |
| 791 | EPMIP_HUMAN | EPM2A-interacting protein 1 | Q7L775 |  | 1.19 | 1.35 |
| 792 | EPN1_HUMAN | Epsin-1 | Q9Y6I3 | 1.12 | 0.99 | 0.91 |
| 793 | EPN2_HUMAN | Epsin-2 | O95208 | 2.17 | 0.74 | 1.03 |
| 794 | EPN4_HUMAN | Clathrin interactor 1 | Q14677 | 0.27 |  | 1.06 |
| 795 | EPS15_HUMAN | Epidermal growth factor receptor substrate 15 | P42566 | 0.85 | 0.82 | 1.12 |
| 796 | ERAP1_HUMAN | Endoplasmic reticulum aminopeptidase 1 | Q9NZ08 |  | 1.22 | 0.92 |
| 797 | ERF1_HUMAN | Eukaryotic peptide chain release factor subunit 1 | P62495 |  | 0.80 | 0.88 |
| 798 | ERF3A_HUMAN | Eukaryotic peptide chain release factor GTP-binding subunit ERF3A | P15170 | 2.29 | 1.89 | 1.21 |
| 799 | ERG24_HUMAN | Delta(14)-sterol reductase | O76062 | 0.61 | 0.74 | 1.84 |
| 800 | ERG7_HUMAN | Lanosterol synthase | P48449 |  | 0.63 | 1.13 |
| 801 | ERGI1_HUMAN | Endoplasmic reticulum-Golgi intermediate compartment protein 1 | Q969X5 |  | 1.75 | 0.54 |
| 802 | ERH_HUMAN | Enhancer of rudimentary homolog | P84090 | 0.60 | 1.17 |  |
| 803 | ERI3_HUMAN | ERI1 exoribonuclease 3 | O43414 |  | 0.65 | 1.02 |
| 804 | ERLN2_HUMAN | Erlin-2 | O94905 | 1.02 | 1.02 |  |
| 805 | ERMP1_HUMAN | Endoplasmic reticulum metallopeptidase 1 | Q7Z2K6 |  | 0.52 | 0.71 |
| 806 | ERO1A_HUMAN | ERO1-like protein alpha | Q96HE7 |  | 1.53 | 0.79 |
| 807 | ERP29_HUMAN | Endoplasmic reticulum resident protein 29 | P30040 | 0.85 | 1.21 | 0.90 |
| 808 | ERP44_HUMAN | Endoplasmic reticulum resident protein 44 | Q9BS26 | 1.08 | 0.22 | 0.99 |
| 809 | ES1_HUMAN | ES1 protein homolog, mitochondrial | P30042 | 0.51 | 0.97 | 1.03 |
| 810 | EST1_HUMAN | Liver carboxylesterase 1 | P23141 | 0.64 |  | 1.71 |
| 811 | EST2_HUMAN | Cocaine esterase | O00748 | 0.45 | 0.60 | 0.82 |
| 812 | ESTD_HUMAN | S-formylglutathione hydrolase | P10768 | 1.72 |  | 0.69 |
| 813 | ESYT1_HUMAN | Extended synaptotagmin-1 | Q9BSJ8 |  |  | 1.11 |
| 814 | ESYT2_HUMAN | Extended synaptotagmin-2 | A0FGR8 | 1.04 | 1.17 | 1.26 |
| 815 | ETFA_HUMAN | Electron transfer flavoprotein subunit alpha, mitochondrial | P13804 | 1.45 |  | 0.71 |
| 816 | ETFB_HUMAN | Electron transfer flavoprotein subunit beta | P38117 | 0.94 | 0.60 | 0.90 |
| 817 | ETFD_HUMAN | Electron transfer flavoprotein-ubiquinone oxidoreductase, mitochondrial | Q16134 | 0.55 | 0.70 | 1.06 |
| 818 | ETHE1_HUMAN | Persulfide dioxygenase ETHE1, mitochondrial | O95571 | 1.27 | 0.77 | 1.22 |
| 819 | EXOC2_HUMAN | Exocyst complex component 2 | Q96KP1 | 1.11 | 1.31 | 0.58 |
| 820 | EXOC4_HUMAN | Exocyst complex component 4 | Q96A65 | 1.13 | 1.00 | 0.68 |
| 821 | EXOC5_HUMAN | Exocyst complex component 5 | O00471 |  | 0.37 | 0.98 |
| 822 | EXOC7_HUMAN | Exocyst complex component 7 | Q9UPT5 | 0.85 |  | 1.00 |
| 823 | EXOC8_HUMAN | Exocyst complex component 8 | Q8IYI6 |  | 5.97 | 0.94 |
| 824 | EXOG_HUMAN | Nuclease EXOG, mitochondrial | Q9Y2C4 | 1.04 | 1.20 | 1.12 |
| 825 | EZRI_HUMAN | Ezrin | P15311 | 1.64 | 2.40 | 1.00 |
| 826 | F10A1_HUMAN | Hsc70-interacting protein | P50502 | 1.07 | 0.61 | 1.37 |
| 827 | F1142_HUMAN | Protein FAM114A2 | Q9NRY5 |  | 0.77 | 0.55 |
| 828 | F120A_HUMAN | Constitutive coactivator of PPAR-gamma-like protein 1 | Q9NZB2 | 3.19 | 0.84 | 0.97 |
| 829 | F136A_HUMAN | Protein FAM136A | Q96C01 | 1.04 | 1.32 | 0.45 |
| 830 | F13A_HUMAN | Coagulation factor XIII A chain | P00488 | 1.80 | 0.83 | 1.17 |
| 831 | F162A_HUMAN | Protein FAM162A | Q96A26 |  | 0.67 | 1.23 |
| 832 | F210A_HUMAN | Protein FAM210A | Q96ND0 | 1.60 | 4.79 | 0.86 |
| 833 | F213A_HUMAN | Redox-regulatory protein FAM213A | Q9BRX8 | 1.46 |  | 0.88 |
| 834 | F262_HUMAN | 6-phosphofructo-2-kinase/fructose-2,6-bisphosphatase 2 | O60825 | 0.38 | 0.74 | 0.93 |
| 835 | FA49B_HUMAN | Protein FAM49B | Q9NUQ9 | 1.15 | 0.98 | 1.38 |
| 836 | FA9_HUMAN | Coagulation factor IX | P00740 | 1.11 | 0.89 | 0.67 |
| 837 | FA98A_HUMAN | Protein FAM98A | Q8NCA5 | 1.58 | 0.96 |  |
| 838 | FA98B_HUMAN | Protein FAM98B | Q52LJ0 | 1.89 | 1.39 | 1.17 |
| 839 | FA98C_HUMAN | Protein FAM98C | Q17RN3 | 1.98 |  | 0.82 |
| 840 | FAAA_HUMAN | Fumarylacetoacetase | P16930 | 1.11 | 0.53 | 0.88 |
| 841 | FABD_HUMAN | Malonyl-CoA-acyl carrier protein transacylase, mitochondrial | Q8IVS2 | 0.64 |  | 0.90 |
| 842 | FABP4_HUMAN | Fatty acid-binding protein, adipocyte | P15090 | 1.39 | 1.14 | 0.80 |
| 843 | FABP5_HUMAN | Fatty acid-binding protein, epidermal | Q01469 | 0.24 | 1.31 | 1.32 |
| 844 | FABPH_HUMAN | Fatty acid-binding protein, heart | P05413 | 1.43 | 0.72 | 0.89 |
| 845 | FAF2_HUMAN | FAS-associated factor 2 | Q96CS3 | 0.61 | 0.78 | 0.94 |
| 846 | FAH2A_HUMAN | Fumarylacetoacetate hydrolase domain-containing protein 2A | Q96GK7 | 1.46 | 0.78 | 1.17 |
| 847 | FAHD1_HUMAN | Acylpyruvase FAHD1, mitochondrial | Q6P587 | 0.95 | 0.92 | 0.86 |
| 848 | FAK1_HUMAN | Focal adhesion kinase 1 | Q05397 |  | 2.15 | 1.38 |
| 849 | FAKD2_HUMAN | FAST kinase domain-containing protein 2 | Q9NYY8 | 0.55 |  | 1.43 |
| 850 | FAKD5_HUMAN | FAST kinase domain-containing protein 5 | Q7L8L6 |  | 0.90 | 1.57 |
| 851 | FAM3C_HUMAN | Protein FAM3C | Q92520 | 0.63 | 0.66 | 1.32 |
| 852 | FAS_HUMAN | Fatty acid synthase | P49327 | 0.45 | 1.01 | 0.86 |
| 853 | FBLI1_HUMAN | Filamin-binding LIM protein 1 | Q8WUP2 |  | 1.24 | 0.60 |
| 854 | FBLN1_HUMAN | Fibulin-1 | P23142 | 0.67 | 0.80 | 1.18 |
| 855 | FBLN2_HUMAN | Fibulin-2 | P98095 | 0.86 | 1.94 | 1.07 |
| 856 | FBLN3_HUMAN | EGF-containing fibulin-like extracellular matrix protein 1 | Q12805 | 1.19 | 1.38 | 1.11 |
| 857 | FBLN5_HUMAN | Fibulin-5 | Q9UBX5 |  |  | 0.97 |
| 858 | FBN1_HUMAN | Fibrillin-1 | P35555 | 0.95 | 1.34 | 1.34 |
| 859 | FBRL_HUMAN | rRNA 2'-O-methyltransferase fibrillarin | P22087 |  | 1.91 | 0.45 |
| 860 | FBX40_HUMAN | F-box only protein 40 | Q9UH90 | 0.28 | 0.70 | 0.98 |
| 861 | FCGRN_HUMAN | IgG receptor FcRn large subunit p51 | P55899 |  | 0.59 | 0.85 |
| 862 | FCL_HUMAN | GDP-L-fucose synthase | Q13630 | 1.16 | 1.77 | 1.04 |
| 863 | FERM2_HUMAN | Fermitin family homolog 2 | Q96AC1 | 1.03 |  | 1.45 |
| 864 | FETUA_HUMAN | Alpha-2-HS-glycoprotein | P02765 | 1.02 |  | 0.72 |
| 865 | FGF1_HUMAN | Fibroblast growth factor 1 | P05230 |  | 0.52 | 0.64 |
| 866 | FGF12_HUMAN | Fibroblast growth factor 12 | P61328 | 0.95 | 0.60 | 1.27 |
| 867 | FHIT_HUMAN | Bis(5'-adenosyl)-triphosphatase | P49789 | 1.66 | 0.98 | 1.05 |
| 868 | FHL1_HUMAN | Four and a half LIM domains protein 1 | Q13642 | 1.79 | 0.92 | 0.59 |
| 869 | FHL2_HUMAN | Four and a half LIM domains protein 2 | Q14192 | 1.13 | 1.02 | 0.91 |
| 870 | FHOD3_HUMAN | FH1/FH2 domain-containing protein 3 | Q2V2M9 | 1.14 |  |  |
| 871 | FHR1_HUMAN | Complement factor H-related protein 1 | Q03591 |  | 2.36 | 1.00 |
| 872 | FIBA_HUMAN | Fibrinogen alpha chain | P02671 | 0.96 | 2.11 | 0.89 |
| 873 | FIBB_HUMAN | Fibrinogen beta chain | P02675 | 1.07 | 0.96 | 0.95 |
| 874 | FIBG_HUMAN | Fibrinogen gamma chain | P02679 | 0.36 | 1.33 | 0.97 |
| 875 | FINC_HUMAN | Fibronectin | P02751 |  |  | 0.60 |
| 876 | FIS1_HUMAN | Mitochondrial fission 1 protein | Q9Y3D6 | 0.86 | 0.89 | 0.84 |
| 877 | FITM1_HUMAN | Fat storage-inducing transmembrane protein 1 | A5D6W6 | 0.65 | 1.17 |  |
| 878 | FKB1A_HUMAN | Peptidyl-prolyl cis-trans isomerase FKBP1A | P62942 | 0.89 | 1.36 | 1.91 |
| 879 | FKBP2_HUMAN | Peptidyl-prolyl cis-trans isomerase FKBP2 | P26885 | 0.46 | 1.21 | 1.57 |
| 880 | FKBP3_HUMAN | Peptidyl-prolyl cis-trans isomerase FKBP3 | Q00688 | 0.61 | 0.90 | 1.12 |
| 881 | FKBP4_HUMAN | Peptidyl-prolyl cis-trans isomerase FKBP4 | Q02790 |  | 1.67 | 1.23 |
| 882 | FKBP5_HUMAN | Peptidyl-prolyl cis-trans isomerase FKBP5 | Q13451 |  |  | 0.68 |
| 883 | FKBP8_HUMAN | Peptidyl-prolyl cis-trans isomerase FKBP8 | Q14318 | 1.42 | 0.85 | 0.63 |
| 884 | FKBP9_HUMAN | Peptidyl-prolyl cis-trans isomerase FKBP9 | O95302 |  | 4.97 | 1.28 |
| 885 | FLII_HUMAN | Protein flightless-1 homolog | Q13045 | 0.82 |  | 0.67 |
| 886 | FLIP1_HUMAN | Filamin-A-interacting protein 1 | Q7Z7B0 | 0.87 | 1.27 | 0.68 |
| 887 | FLNA_HUMAN | Filamin-A | P21333 | 1.14 | 0.48 | 1.02 |
| 888 | FLNB_HUMAN | Filamin-B | O75369 | 1.28 | 1.74 | 1.26 |
| 889 | FLNC_HUMAN | Filamin-C | Q14315 | 0.89 | 0.79 | 1.02 |
| 890 | FLOT1_HUMAN | Flotillin-1 | O75955 | 0.95 | 1.16 | 1.08 |
| 891 | FLOT2_HUMAN | Flotillin-2 | Q14254 | 0.98 | 0.78 | 0.53 |
| 892 | FMOD_HUMAN | Fibromodulin | Q06828 | 0.64 | 0.99 | 1.18 |
| 893 | FMR1_HUMAN | Fragile X mental retardation protein 1 | Q06787 | 0.56 | 1.07 | 1.48 |
| 894 | FN3K_HUMAN | Fructosamine-3-kinase | Q9H479 | 1.54 | 2.01 | 0.86 |
| 895 | FNTA_HUMAN | Protein farnesyltransferase/geranylgeranyltransferase type-1 subunit alpha | P49354 |  | 0.86 | 0.86 |
| 896 | FPPS_HUMAN | Farnesyl pyrophosphate synthase | P14324 |  | 0.87 | 1.07 |
| 897 | FPRP_HUMAN | Prostaglandin F2 receptor negative regulator | Q9P2B2 | 1.09 | 2.33 | 1.06 |
| 898 | FRDA_HUMAN | Frataxin, mitochondrial | Q16595 |  | 1.01 | 0.88 |
| 899 | FRIH_HUMAN | Ferritin heavy chain | P02794 | 0.72 | 0.97 | 1.52 |
| 900 | FRIL_HUMAN | Ferritin light chain | P02792 |  | 0.98 | 0.75 |
| 901 | FRMD5_HUMAN | FERM domain-containing protein 5 | Q7Z6J6 |  | 0.98 |  |
| 902 | FSCN1_HUMAN | Fascin | Q16658 | 0.81 |  | 0.94 |
| 903 | FSD2_HUMAN | Fibronectin type III and SPRY domain-containing protein 2 | A1L4K1 |  | 0.89 | 0.65 |
| 904 | FUBP1_HUMAN | Far upstream element-binding protein 1 | Q96AE4 | 2.96 | 0.88 | 0.74 |
| 905 | FUBP2_HUMAN | Far upstream element-binding protein 2 | Q92945 | 1.00 | 1.00 | 0.72 |
| 906 | FUCO_HUMAN | Tissue alpha-L-fucosidase | P04066 |  | 0.77 |  |
| 907 | FUMH_HUMAN | Fumarate hydratase, mitochondrial | P07954 | 1.07 |  | 1.15 |
| 908 | FUND2_HUMAN | FUN14 domain-containing protein 2 | Q9BWH2 | 1.27 | 1.09 | 0.85 |
| 909 | FXR1_HUMAN | Fragile X mental retardation syndrome-related protein 1 | P51114 | 1.29 | 0.71 | 1.15 |
| 910 | FXR2_HUMAN | Fragile X mental retardation syndrome-related protein 2 | P51116 |  | 0.31 | 0.77 |
| 911 | FYCO1_HUMAN | FYVE and coiled-coil domain-containing protein 1 | Q9BQS8 | 1.47 | 0.96 | 0.91 |
| 912 | G3BP1_HUMAN | Ras GTPase-activating protein-binding protein 1 | Q13283 |  | 0.86 | 1.24 |
| 913 | G3BP2_HUMAN | Ras GTPase-activating protein-binding protein 2 | Q9UN86 |  | 0.95 | 0.59 |
| 914 | G3P_HUMAN | Glyceraldehyde-3-phosphate dehydrogenase | P04406 |  |  | 1.23 |
| 915 | G45IP_HUMAN | Growth arrest and DNA damage-inducible proteins-interacting protein 1 | Q8TAE8 | 1.02 | 0.82 | 0.87 |
| 916 | G6PD_HUMAN | Glucose-6-phosphate 1-dehydrogenase | P11413 | 0.95 |  | 1.18 |
| 917 | G6PE_HUMAN | GDH/6PGL endoplasmic bifunctional protein | O95479 |  | 0.95 | 0.84 |
| 918 | G6PI_HUMAN | Glucose-6-phosphate isomerase | P06744 | 1.17 | 1.05 | 0.98 |
| 919 | GABT_HUMAN | 4-aminobutyrate aminotransferase, mitochondrial | P80404 | 1.05 | 1.42 | 0.99 |
| 920 | GALK1_HUMAN | Galactokinase | P51570 | 1.38 | 0.90 |  |
| 921 | GALM_HUMAN | Aldose 1-epimerase | Q96C23 |  |  | 0.86 |
| 922 | GALNS_HUMAN | N-acetylgalactosamine-6-sulfatase | P34059 | 1.60 |  |  |
| 923 | GALT_HUMAN | Galactose-1-phosphate uridylyltransferase | P07902 |  | 1.22 | 0.76 |
| 924 | GAMT_HUMAN | Guanidinoacetate N-methyltransferase | Q14353 | 0.64 | 1.01 | 1.15 |
| 925 | GANAB_HUMAN | Neutral alpha-glucosidase AB | Q14697 | 0.46 |  | 0.98 |
| 926 | GAPR1_HUMAN | Golgi-associated plant pathogenesis-related protein 1 | Q9H4G4 | 1.54 |  | 0.81 |
| 927 | GAS6_HUMAN | Growth arrest-specific protein 6 | Q14393 |  | 0.88 | 0.77 |
| 928 | GATB_HUMAN | Glutamyl-tRNA(Gln) amidotransferase subunit B, mitochondrial | O75879 |  | 2.70 | 1.88 |
| 929 | GATM_HUMAN | Glycine amidinotransferase, mitochondrial | P50440 | 1.08 | 0.32 | 0.98 |
| 930 | GBB1_HUMAN | Guanine nucleotide-binding protein G(I)/G(S)/G(T) subunit beta-1 | P62873 | 1.32 |  | 0.82 |
| 931 | GBG12_HUMAN | Guanine nucleotide-binding protein G(I)/G(S)/G(O) subunit gamma-12 | Q9UBI6 | 0.62 | 0.78 | 1.47 |
| 932 | GBG2_HUMAN | Guanine nucleotide-binding protein G(I)/G(S)/G(O) subunit gamma-2 | P59768 |  | 2.13 | 0.25 |
| 933 | GBG5_HUMAN | Guanine nucleotide-binding protein G(I)/G(S)/G(O) subunit gamma-5 | P63218 |  | 0.94 | 0.65 |
| 934 | GBG7_HUMAN | Guanine nucleotide-binding protein G(I)/G(S)/G(O) subunit gamma-7 | O60262 | 0.63 | 0.77 | 0.90 |
| 935 | GBLP_HUMAN | Guanine nucleotide-binding protein subunit beta-2-like 1 | P63244 | 3.47 |  | 1.02 |
| 936 | GBRL2_HUMAN | Gamma-aminobutyric acid receptor-associated protein-like 2 | P60520 | 0.86 | 2.88 | 0.90 |
| 937 | GCDH_HUMAN | Glutaryl-CoA dehydrogenase, mitochondrial | Q92947 | 1.42 |  | 1.16 |
| 938 | GCN1L_HUMAN | Translational activator GCN1 | Q92616 | 0.63 | 1.08 | 1.07 |
| 939 | GCP60_HUMAN | Golgi resident protein GCP60 | Q9H3P7 |  | 1.17 | 0.54 |
| 940 | GCST_HUMAN | Aminomethyltransferase, mitochondrial | P48728 | 1.05 | 1.06 | 1.04 |
| 941 | GCYB1_HUMAN | Guanylate cyclase soluble subunit beta-1 | Q02153 | 0.84 |  | 0.70 |
| 942 | GDE_HUMAN | Glycogen debranching enzyme | P35573 |  |  | 1.06 |
| 943 | GDIA_HUMAN | Rab GDP dissociation inhibitor alpha | P31150 | 1.27 | 0.92 | 0.98 |
| 944 | GDIB_HUMAN | Rab GDP dissociation inhibitor beta | P50395 | 0.93 |  | 0.86 |
| 945 | GDIR1_HUMAN | Rho GDP-dissociation inhibitor 1 | P52565 | 0.63 | 1.09 | 0.89 |
| 946 | GDIR2_HUMAN | Rho GDP-dissociation inhibitor 2 | P52566 | 0.07 | 1.20 | 1.16 |
| 947 | GELS_HUMAN | Gelsolin | P06396 | 1.03 |  | 0.94 |
| 948 | GEMI5_HUMAN | Gem-associated protein 5 | Q8TEQ6 |  | 1.01 |  |
| 949 | GEPH_HUMAN | Gephyrin | Q9NQX3 |  | 1.12 | 0.69 |
| 950 | GGCT_HUMAN | Gamma-glutamylcyclotransferase | O75223 | 0.74 | 1.60 | 1.13 |
| 951 | GGT5_HUMAN | Gamma-glutamyltransferase 5 | P36269 | 1.18 | 1.00 | 1.03 |
| 952 | GHITM_HUMAN | Growth hormone-inducible transmembrane protein | Q9H3K2 | 3.10 | 0.75 | 0.94 |
| 953 | GIMA1_HUMAN | GTPase IMAP family member 1 | Q8WWP7 |  | 5.45 | 0.79 |
| 954 | GIMA4_HUMAN | GTPase IMAP family member 4 | Q9NUV9 | 0.67 | 1.46 | 0.80 |
| 955 | GIPC1_HUMAN | PDZ domain-containing protein GIPC1 | O14908 |  | 1.11 | 0.92 |
| 956 | GIT1_HUMAN | ARF GTPase-activating protein GIT1 | Q9Y2X7 |  | 0.45 | 0.32 |
| 957 | GL1AD_HUMAN | DNA-directed RNA polymerase II subunit GRINL1A, isoforms 4/5 | Q6EEV4 | 1.47 | 0.40 | 0.67 |
| 958 | GLGB_HUMAN | 1,4-alpha-glucan-branching enzyme | Q04446 |  | 1.84 | 1.34 |
| 959 | GLO2_HUMAN | Hydroxyacylglutathione hydrolase, mitochondrial | Q16775 | 0.95 | 2.07 | 0.92 |
| 960 | GLOD4_HUMAN | Glyoxalase domain-containing protein 4 | Q9HC38 | 0.35 | 1.14 | 0.98 |
| 961 | GLPC_HUMAN | Glycophorin-C | P04921 |  | 1.34 | 1.74 |
| 962 | GLPK_HUMAN | Glycerol kinase | P32189 | 0.06 | 0.96 | 0.81 |
| 963 | GLRX1_HUMAN | Glutaredoxin-1 | P35754 | 1.08 | 0.98 |  |
| 964 | GLRX3_HUMAN | Glutaredoxin-3 | O76003 | 0.49 | 0.86 |  |
| 965 | GLRX5_HUMAN | Glutaredoxin-related protein 5, mitochondrial | Q86SX6 | 1.47 | 0.89 | 1.06 |
| 966 | GLSK_HUMAN | Glutaminase kidney isoform, mitochondrial | O94925 | 0.47 | 1.01 | 1.02 |
| 967 | GLU2B_HUMAN | Glucosidase 2 subunit beta | P14314 | 0.44 | 0.99 | 1.08 |
| 968 | GLYG_HUMAN | Glycogenin-1 | P46976 | 0.79 |  | 1.32 |
| 969 | GLYM_HUMAN | Serine hydroxymethyltransferase, mitochondrial | P34897 | 0.83 |  | 1.17 |
| 970 | GMFB_HUMAN | Glia maturation factor beta | P60983 |  |  | 0.59 |
| 971 | GMPPB_HUMAN | Mannose-1-phosphate guanyltransferase beta | Q9Y5P6 |  | 1.38 | 0.91 |
| 972 | GMPR1_HUMAN | GMP reductase 1 | P36959 |  | 0.58 | 0.69 |
| 973 | GNA11_HUMAN | Guanine nucleotide-binding protein subunit alpha-11 | P29992 | 0.95 | 1.28 | 1.12 |
| 974 | GNA13_HUMAN | Guanine nucleotide-binding protein subunit alpha-13 | Q14344 |  | 1.13 | 1.01 |
| 975 | GNAI2_HUMAN | Guanine nucleotide-binding protein G(i) subunit alpha-2 | P04899 | 1.72 |  | 1.06 |
| 976 | GNAI3_HUMAN | Guanine nucleotide-binding protein G(k) subunit alpha | P08754 | 1.16 | 1.06 | 1.15 |
| 977 | GNAO_HUMAN | Guanine nucleotide-binding protein G(o) subunit alpha | P09471 |  | 1.28 | 0.90 |
| 978 | GNAQ_HUMAN | Guanine nucleotide-binding protein G(q) subunit alpha | P50148 |  | 0.44 | 1.26 |
| 979 | GNAS2_HUMAN | Guanine nucleotide-binding protein G(s) subunit alpha isoforms short | P63092 | 0.31 |  | 1.33 |
| 980 | GNPI1_HUMAN | Glucosamine-6-phosphate isomerase 1 | P46926 | 0.79 | 1.67 | 1.01 |
| 981 | GNPI2_HUMAN | Glucosamine-6-phosphate isomerase 2 | Q8TDQ7 |  |  | 0.65 |
| 982 | GOGA3_HUMAN | Golgin subfamily A member 3 | Q08378 | 0.87 |  | 0.92 |
| 983 | GOGA7_HUMAN | Golgin subfamily A member 7 | Q7Z5G4 |  | 0.79 | 0.94 |
| 984 | GORS2_HUMAN | Golgi reassembly-stacking protein 2 | Q9H8Y8 | 1.05 | 1.09 | 0.61 |
| 985 | GPC1_HUMAN | Glypican-1 | P35052 | 0.38 | 1.77 | 1.30 |
| 986 | GPD1L_HUMAN | Glycerol-3-phosphate dehydrogenase 1-like protein | Q8N335 | 1.57 | 0.69 | 1.05 |
| 987 | GPDA_HUMAN | Glycerol-3-phosphate dehydrogenase [NAD(+)], cytoplasmic | P21695 | 0.66 | 1.17 | 1.18 |
| 988 | GPDM_HUMAN | Glycerol-3-phosphate dehydrogenase, mitochondrial | P43304 |  | 0.90 | 1.03 |
| 989 | GPNMB_HUMAN | Transmembrane glycoprotein NMB | Q14956 | 1.03 | 1.20 |  |
| 990 | GPX1_HUMAN | Glutathione peroxidase 1 | P07203 | 0.66 |  | 1.01 |
| 991 | GPX3_HUMAN | Glutathione peroxidase 3 | P22352 | 0.82 | 1.36 | 1.40 |
| 992 | GPX4_HUMAN | Phospholipid hydroperoxide glutathione peroxidase, mitochondrial | P36969 | 0.64 | 3.16 | 0.79 |
| 993 | GRB2_HUMAN | Growth factor receptor-bound protein 2 | P62993 | 1.17 | 0.90 | 0.85 |
| 994 | GRHPR_HUMAN | Glyoxylate reductase/hydroxypyruvate reductase | Q9UBQ7 | 1.07 | 1.32 | 0.84 |
| 995 | GRP75_HUMAN | Stress-70 protein, mitochondrial | P38646 | 1.27 |  | 0.97 |
| 996 | GRP78_HUMAN | 78 kDa glucose-regulated protein | P11021 | 0.83 |  | 0.97 |
| 997 | GRPE1_HUMAN | GrpE protein homolog 1, mitochondrial | Q9HAV7 | 0.99 | 1.20 | 0.92 |
| 998 | GRSF1_HUMAN | G-rich sequence factor 1 | Q12849 | 1.32 | 1.09 | 0.88 |
| 999 | GSH1_HUMAN | Glutamate--cysteine ligase catalytic subunit | P48506 | 1.46 |  | 0.88 |
| 1000 | GSHB_HUMAN | Glutathione synthetase | P48637 | 0.63 | 0.52 | 1.01 |
| 1001 | GSHR_HUMAN | Glutathione reductase, mitochondrial | P00390 | 1.05 | 0.86 | 0.87 |
| 1002 | GSK3A_HUMAN | Glycogen synthase kinase-3 alpha | P49840 |  | 1.03 | 1.18 |
| 1003 | GSK3B_HUMAN | Glycogen synthase kinase-3 beta | P49841 |  | 5.86 | 1.67 |
| 1004 | GSLG1_HUMAN | Golgi apparatus protein 1 | Q92896 | 1.49 | 0.90 | 0.74 |
| 1005 | GSTK1_HUMAN | Glutathione S-transferase kappa 1 | Q9Y2Q3 | 0.52 | 1.11 | 1.21 |
| 1006 | GSTM1_HUMAN | Glutathione S-transferase Mu 1 | P09488 |  | 2.03 | 1.18 |
| 1007 | GSTM2_HUMAN | Glutathione S-transferase Mu 2 | P28161 | 1.22 |  | 0.98 |
| 1008 | GSTM3_HUMAN | Glutathione S-transferase Mu 3 | P21266 | 0.33 | 0.61 | 0.86 |
| 1009 | GSTO1_HUMAN | Glutathione S-transferase omega-1 | P78417 |  | 0.48 | 0.82 |
| 1010 | GSTP1_HUMAN | Glutathione S-transferase P | P09211 | 0.85 | 1.17 |  |
| 1011 | GTF2I_HUMAN | General transcription factor II-I | P78347 | 1.77 | 0.91 | 0.84 |
| 1012 | GTPB1_HUMAN | GTP-binding protein 1 | O00178 |  | 0.78 | 0.87 |
| 1013 | GTR4_HUMAN | Solute carrier family 2, facilitated glucose transporter member 4 | P14672 | 1.15 | 1.13 | 0.91 |
| 1014 | GUF1_HUMAN | Translation factor GUF1, mitochondrial | Q8N442 | 1.28 | 0.97 |  |
| 1015 | GYS1_HUMAN | Glycogen [starch] synthase, muscle | P13807 | 0.97 | 0.92 | 0.91 |
| 1016 | H10_HUMAN | Histone H1.0 | P07305 | 0.95 |  | 0.97 |
| 1017 | H12_HUMAN | Histone H1.2 | P16403 | 1.38 |  | 0.43 |
| 1018 | H14_HUMAN | Histone H1.4 | P10412 | 1.69 | 1.51 | 0.81 |
| 1019 | H15_HUMAN | Histone H1.5 | P16401 |  | 1.14 | 0.64 |
| 1020 | H1BP3_HUMAN | HCLS1-binding protein 3 | Q53T59 | 1.36 |  | 0.93 |
| 1021 | H1X_HUMAN | Histone H1x | Q92522 | 1.21 | 0.99 | 0.57 |
| 1022 | H2A2B_HUMAN | Histone H2A type 2-B | Q8IUE6 |  | 0.36 | 0.75 |
| 1023 | H2AV_HUMAN | Histone H2A.V | Q71UI9 | 3.56 | 0.70 | 1.16 |
| 1024 | H2AW_HUMAN | Core histone macro-H2A.2 | Q9P0M6 | 0.73 | 0.51 | 1.21 |
| 1025 | H2AY_HUMAN | Core histone macro-H2A.1 | O75367 | 1.25 |  | 0.94 |
| 1026 | H2B1C_HUMAN | Histone H2B type 1-C/E/F/G/I | P62807 |  | 1.67 | 0.89 |
| 1027 | H2B2E_HUMAN | Histone H2B type 2-E | Q16778 |  |  | 0.21 |
| 1028 | H32_HUMAN | Histone H3.2 | Q71DI3 | 1.82 |  |  |
| 1029 | H33_HUMAN | Histone H3.3 | P84243 | 1.17 |  | 1.06 |
| 1030 | H4_HUMAN | Histone H4 | P62805 | 0.94 | 0.67 | 1.09 |
| 1031 | HACD2_HUMAN | Very-long-chain (3R)-3-hydroxyacyl-CoA dehydratase 2 | Q6Y1H2 | 1.04 |  | 0.67 |
| 1032 | HACD3_HUMAN | Very-long-chain (3R)-3-hydroxyacyl-CoA dehydratase 3 | Q9P035 | 0.80 |  | 1.03 |
| 1033 | HAP28_HUMAN | 28 kDa heat- and acid-stable phosphoprotein | Q13442 | 0.42 | 0.52 | 0.85 |
| 1034 | HBA_HUMAN | Hemoglobin subunit alpha | P69905 |  |  | 0.95 |
| 1035 | HBB_HUMAN | Hemoglobin subunit beta | P68871 |  |  | 1.44 |
| 1036 | HBD_HUMAN | Hemoglobin subunit delta | P02042 | 1.09 |  | 0.94 |
| 1037 | HBG1_HUMAN | Hemoglobin subunit gamma-1 | P69891 |  | 1.28 | 0.74 |
| 1038 | HBG2_HUMAN | Hemoglobin subunit gamma-2 | P69892 | 1.16 |  | 0.94 |
| 1039 | HCD2_HUMAN | 3-hydroxyacyl-CoA dehydrogenase type-2 | Q99714 | 1.94 |  | 1.70 |
| 1040 | HCDH_HUMAN | Hydroxyacyl-coenzyme A dehydrogenase, mitochondrial | Q16836 | 0.17 |  | 1.02 |
| 1041 | HCFC1_HUMAN | Host cell factor 1 | P51610 |  | 1.07 |  |
| 1042 | HDDC2_HUMAN | HD domain-containing protein 2 | Q7Z4H3 | 1.41 |  | 1.40 |
| 1043 | HDGF_HUMAN | Hepatoma-derived growth factor | P51858 | 0.46 |  | 1.07 |
| 1044 | HDGR3_HUMAN | Hepatoma-derived growth factor-related protein 3 | Q9Y3E1 | 1.00 | 3.77 | 1.09 |
| 1045 | HDHD1_HUMAN | Pseudouridine-5'-monophosphatase | Q08623 | 1.36 | 1.69 | 0.77 |
| 1046 | HDHD2_HUMAN | Haloacid dehalogenase-like hydrolase domain-containing protein 2 | Q9H0R4 |  | 0.72 | 0.99 |
| 1047 | HDHD3_HUMAN | Haloacid dehalogenase-like hydrolase domain-containing protein 3 | Q9BSH5 | 2.83 | 1.04 |  |
| 1048 | HEBP1_HUMAN | Heme-binding protein 1 | Q9NRV9 | 1.85 | 1.13 | 0.99 |
| 1049 | HEBP2_HUMAN | Heme-binding protein 2 | Q9Y5Z4 | 1.13 | 0.93 | 1.03 |
| 1050 | HEM2_HUMAN | Delta-aminolevulinic acid dehydratase | P13716 |  | 0.96 | 1.01 |
| 1051 | HEMH_HUMAN | Ferrochelatase, mitochondrial | P22830 | 0.83 | 1.25 | 0.96 |
| 1052 | HEMO_HUMAN | Hemopexin | P02790 | 0.47 | 1.11 | 0.70 |
| 1053 | HEP2_HUMAN | Heparin cofactor 2 | P05546 | 1.60 | 0.90 | 0.85 |
| 1054 | HERC4_HUMAN | Probable E3 ubiquitin-protein ligase HERC4 | Q5GLZ8 | 1.41 | 0.96 | 0.88 |
| 1055 | HEXA_HUMAN | Beta-hexosaminidase subunit alpha | P06865 | 0.67 | 0.74 | 1.03 |
| 1056 | HEXB_HUMAN | Beta-hexosaminidase subunit beta | P07686 |  | 0.75 | 0.89 |
| 1057 | HGS_HUMAN | Hepatocyte growth factor-regulated tyrosine kinase substrate | O14964 | 0.72 |  | 0.09 |
| 1058 | HHATL_HUMAN | Protein-cysteine N-palmitoyltransferase HHAT-like protein | Q9HCP6 | 0.82 | 0.79 |  |
| 1059 | HIBCH_HUMAN | 3-hydroxyisobutyryl-CoA hydrolase, mitochondrial | Q6NVY1 | 0.49 | 1.06 | 0.99 |
| 1060 | HIG1A_HUMAN | HIG1 domain family member 1A, mitochondrial | Q9Y241 | 1.39 |  | 1.16 |
| 1061 | HIKES_HUMAN | Protein Hikeshi | Q53FT3 | 0.59 |  | 0.93 |
| 1062 | HINT1_HUMAN | Histidine triad nucleotide-binding protein 1 | P49773 | 0.75 |  | 1.13 |
| 1063 | HINT2_HUMAN | Histidine triad nucleotide-binding protein 2, mitochondrial | Q9BX68 | 1.05 | 1.60 | 1.09 |
| 1064 | HINT3_HUMAN | Histidine triad nucleotide-binding protein 3 | Q9NQE9 | 1.41 | 1.53 | 0.58 |
| 1065 | HIP1R_HUMAN | Huntingtin-interacting protein 1-related protein | O75146 | 0.58 | 0.64 | 1.16 |
| 1066 | HMGB1_HUMAN | High mobility group protein B1 | P09429 | 0.86 | 1.07 | 0.95 |
| 1067 | HMGCL_HUMAN | Hydroxymethylglutaryl-CoA lyase, mitochondrial | P35914 | 0.27 | 1.75 | 0.61 |
| 1068 | HMGN2_HUMAN | Non-histone chromosomal protein HMG-17 | P05204 | 0.19 | 1.82 | 0.70 |
| 1069 | HMOX2_HUMAN | Heme oxygenase 2 | P30519 | 0.86 | 0.85 | 1.07 |
| 1070 | HNRDL_HUMAN | Heterogeneous nuclear ribonucleoprotein D-like | O14979 | 0.99 | 0.97 | 0.86 |
| 1071 | HNRH1_HUMAN | Heterogeneous nuclear ribonucleoprotein H | P31943 |  | 0.74 | 0.84 |
| 1072 | HNRH3_HUMAN | Heterogeneous nuclear ribonucleoprotein H3 | P31942 | 1.39 | 1.43 | 0.72 |
| 1073 | HNRL2_HUMAN | Heterogeneous nuclear ribonucleoprotein U-like protein 2 | Q1KMD3 | 1.31 | 1.06 | 1.30 |
| 1074 | HNRPC_HUMAN | Heterogeneous nuclear ribonucleoproteins C1/C2 | P07910 | 0.31 | 1.72 | 1.39 |
| 1075 | HNRPD_HUMAN | Heterogeneous nuclear ribonucleoprotein D0 | Q14103 | 1.63 | 1.11 | 1.05 |
| 1076 | HNRPK_HUMAN | Heterogeneous nuclear ribonucleoprotein K | P61978 | 0.85 | 0.81 | 1.21 |
| 1077 | HNRPL_HUMAN | Heterogeneous nuclear ribonucleoprotein L | P14866 |  | 2.31 | 1.17 |
| 1078 | HNRPM_HUMAN | Heterogeneous nuclear ribonucleoprotein M | P52272 | 0.65 |  | 1.10 |
| 1079 | HNRPQ_HUMAN | Heterogeneous nuclear ribonucleoprotein Q | O60506 | 1.85 | 1.67 | 1.29 |
| 1080 | HNRPR_HUMAN | Heterogeneous nuclear ribonucleoprotein R | O43390 | 2.75 | 1.26 | 1.08 |
| 1081 | HNRPU_HUMAN | Heterogeneous nuclear ribonucleoprotein U | Q00839 | 0.75 | 1.38 | 1.01 |
| 1082 | HOME1_HUMAN | Homer protein homolog 1 | Q86YM7 | 2.96 | 1.49 | 1.03 |
| 1083 | HOT_HUMAN | Hydroxyacid-oxoacid transhydrogenase, mitochondrial | Q8IWW8 | 0.51 | 1.03 | 1.01 |
| 1084 | HP1B3_HUMAN | Heterochromatin protein 1-binding protein 3 | Q5SSJ5 | 0.97 | 0.90 | 0.88 |
| 1085 | HPBP1_HUMAN | Hsp70-binding protein 1 | Q9NZL4 |  | 1.24 | 0.61 |
| 1086 | HPCL1_HUMAN | Hippocalcin-like protein 1 | P37235 | 0.78 |  | 0.89 |
| 1087 | HPLN1_HUMAN | Hyaluronan and proteoglycan link protein 1 | P10915 | 1.25 | 0.95 | 1.07 |
| 1088 | HPRT_HUMAN | Hypoxanthine-guanine phosphoribosyltransferase | P00492 | 1.56 | 1.32 | 0.98 |
| 1089 | HPT_HUMAN | Haptoglobin | P00738 | 1.15 | 1.33 | 1.00 |
| 1090 | HRG_HUMAN | Histidine-rich glycoprotein | P04196 | 1.45 | 1.06 |  |
| 1091 | HS105_HUMAN | Heat shock protein 105 kDa | Q92598 | 2.81 | 0.90 | 1.23 |
| 1092 | HS12A_HUMAN | Heat shock 70 kDa protein 12A | O43301 | 0.82 | 0.82 | 0.93 |
| 1093 | HS12B_HUMAN | Heat shock 70 kDa protein 12B | Q96MM6 | 0.30 | 0.90 | 1.01 |
| 1094 | HS90A_HUMAN | Heat shock protein HSP 90-alpha | P07900 | 0.70 |  | 1.01 |
| 1095 | HS90B_HUMAN | Heat shock protein HSP 90-beta | P08238 | 0.82 | 1.57 | 0.96 |
| 1096 | HSBP1_HUMAN | Heat shock factor-binding protein 1 | O75506 | 1.84 | 0.95 | 1.09 |
| 1097 | HSC20_HUMAN | Iron-sulfur cluster co-chaperone protein HscB, mitochondrial | Q8IWL3 | 0.57 |  | 0.87 |
| 1098 | HSDL1_HUMAN | Inactive hydroxysteroid dehydrogenase-like protein 1 | Q3SXM5 | 0.98 | 0.99 | 1.14 |
| 1099 | HSDL2_HUMAN | Hydroxysteroid dehydrogenase-like protein 2 | Q6YN16 | 0.92 |  | 1.70 |
| 1100 | HSP72_HUMAN | Heat shock-related 70 kDa protein 2 | P54652 | 0.90 | 0.95 | 1.21 |
| 1101 | HSP74_HUMAN | Heat shock 70 kDa protein 4 | P34932 | 0.58 |  | 1.13 |
| 1102 | HSP7C_HUMAN | Heat shock cognate 71 kDa protein | P11142 | 1.89 |  | 1.06 |
| 1103 | HSPB1_HUMAN | Heat shock protein beta-1 | P04792 | 1.31 | 1.38 | 1.14 |
| 1104 | HSPB2_HUMAN | Heat shock protein beta-2 | Q16082 |  | 4.49 | 0.78 |
| 1105 | HSPB3_HUMAN | Heat shock protein beta-3 | Q12988 | 1.25 | 0.18 | 0.89 |
| 1106 | HSPB6_HUMAN | Heat shock protein beta-6 | O14558 | 1.45 |  | 0.75 |
| 1107 | HSPB7_HUMAN | Heat shock protein beta-7 | Q9UBY9 | 0.99 |  | 1.08 |
| 1108 | HSPB8_HUMAN | Heat shock protein beta-8 | Q9UJY1 |  | 1.01 | 1.11 |
| 1109 | HTRA2_HUMAN | Serine protease HTRA2, mitochondrial | O43464 | 1.31 | 0.63 | 1.04 |
| 1110 | HUWE1_HUMAN | E3 ubiquitin-protein ligase HUWE1 | Q7Z6Z7 | 1.89 | 1.26 | 1.06 |
| 1111 | HV103_HUMAN | Ig heavy chain V-I region V35 | P23083 | 0.90 |  | 0.94 |
| 1112 | HV208_HUMAN | Ig heavy chain V-II region SESS | P04438 | 0.95 |  | 0.55 |
| 1113 | HV209_HUMAN | Ig heavy chain V-II region ARH-77 | P06331 | 1.27 | 1.53 | 1.06 |
| 1114 | HV302_HUMAN | Ig heavy chain V-III region WEA | P01763 | 1.08 |  | 1.28 |
| 1115 | HV303_HUMAN | Ig heavy chain V-III region VH26 | P01764 | 0.78 | 0.84 | 0.83 |
| 1116 | HV311_HUMAN | Ig heavy chain V-III region KOL | P01772 | 2.42 | 0.86 | 1.04 |
| 1117 | HV320_HUMAN | Ig heavy chain V-III region GAL | P01781 |  | 0.85 | 1.14 |
| 1118 | HXK1_HUMAN | Hexokinase-1 | P19367 | 2.01 |  | 1.29 |
| 1119 | HYEP_HUMAN | Epoxide hydrolase 1 | P07099 | 0.95 | 0.86 |  |
| 1120 | HYES_HUMAN | Bifunctional epoxide hydrolase 2 | P34913 | 1.17 | 1.12 | 1.21 |
| 1121 | HYI_HUMAN | Putative hydroxypyruvate isomerase | Q5T013 | 1.54 | 2.09 | 0.98 |
| 1122 | HYOU1_HUMAN | Hypoxia up-regulated protein 1 | Q9Y4L1 | 0.91 | 1.15 | 1.13 |
| 1123 | IAH1_HUMAN | Isoamyl acetate-hydrolyzing esterase 1 homolog | Q2TAA2 | 1.46 | 1.18 | 0.97 |
| 1124 | IASPP_HUMAN | RelA-associated inhibitor | Q8WUF5 | 1.03 | 6.67 | 1.01 |
| 1125 | IBP7_HUMAN | Insulin-like growth factor-binding protein 7 | Q16270 | 1.17 | 0.46 | 0.78 |
| 1126 | IC1_HUMAN | Plasma protease C1 inhibitor | P05155 | 2.27 | 0.70 | 1.04 |
| 1127 | ICAL_HUMAN | Calpastatin | P20810 | 0.42 | 0.95 | 1.38 |
| 1128 | ICAM1_HUMAN | Intercellular adhesion molecule 1 | P05362 |  | 0.29 | 1.37 |
| 1129 | ICLN_HUMAN | Methylosome subunit pICln | P54105 | 1.07 |  | 1.20 |
| 1130 | IDE_HUMAN | Insulin-degrading enzyme | P14735 | 0.65 | 1.39 | 1.21 |
| 1131 | IDH3A_HUMAN | Isocitrate dehydrogenase [NAD] subunit alpha, mitochondrial | P50213 | 0.67 | 0.95 |  |
| 1132 | IDH3B_HUMAN | Isocitrate dehydrogenase [NAD] subunit beta, mitochondrial | O43837 |  | 1.24 | 1.11 |
| 1133 | IDH3G_HUMAN | Isocitrate dehydrogenase [NAD] subunit gamma, mitochondrial | P51553 | 0.52 | 1.63 | 0.70 |
| 1134 | IDHC_HUMAN | Isocitrate dehydrogenase [NADP] cytoplasmic | O75874 | 0.67 | 1.15 | 1.00 |
| 1135 | IDHP_HUMAN | Isocitrate dehydrogenase [NADP], mitochondrial | P48735 | 1.38 |  | 1.12 |
| 1136 | IF16_HUMAN | Gamma-interferon-inducible protein 16 | Q16666 |  | 2.88 | 0.66 |
| 1137 | IF1AX_HUMAN | Eukaryotic translation initiation factor 1A, X-chromosomal | P47813 | 1.57 | 0.60 | 1.05 |
| 1138 | IF2A_HUMAN | Eukaryotic translation initiation factor 2 subunit 1 | P05198 | 2.09 | 1.05 | 0.98 |
| 1139 | IF2B_HUMAN | Eukaryotic translation initiation factor 2 subunit 2 | P20042 | 1.25 | 1.17 | 0.93 |
| 1140 | IF2B2_HUMAN | Insulin-like growth factor 2 mRNA-binding protein 2 | Q9Y6M1 |  | 1.34 | 0.94 |
| 1141 | IF2G_HUMAN | Eukaryotic translation initiation factor 2 subunit 3 | P41091 | 0.91 |  | 1.09 |
| 1142 | IF2M_HUMAN | Translation initiation factor IF-2, mitochondrial | P46199 | 1.11 | 1.42 | 0.98 |
| 1143 | IF2P_HUMAN | Eukaryotic translation initiation factor 5B | O60841 | 0.73 |  | 0.60 |
| 1144 | IF3M_HUMAN | Translation initiation factor IF-3, mitochondrial | Q9H2K0 | 1.18 |  | 0.91 |
| 1145 | IF4A1_HUMAN | Eukaryotic initiation factor 4A-I | P60842 | 1.25 | 1.04 | 0.77 |
| 1146 | IF4A2_HUMAN | Eukaryotic initiation factor 4A-II | Q14240 | 0.95 | 0.61 | 1.16 |
| 1147 | IF4A3_HUMAN | Eukaryotic initiation factor 4A-III | P38919 | 0.97 |  | 0.94 |
| 1148 | IF4B_HUMAN | Eukaryotic translation initiation factor 4B | P23588 | 0.60 | 0.65 | 1.16 |
| 1149 | IF4E_HUMAN | Eukaryotic translation initiation factor 4E | P06730 |  | 1.58 | 1.20 |
| 1150 | IF4G1_HUMAN | Eukaryotic translation initiation factor 4 gamma 1 | Q04637 | 0.90 | 4.06 | 0.82 |
| 1151 | IF4G2_HUMAN | Eukaryotic translation initiation factor 4 gamma 2 | P78344 | 1.26 | 0.96 | 0.87 |
| 1152 | IF4H_HUMAN | Eukaryotic translation initiation factor 4H | Q15056 | 2.21 | 0.78 | 1.26 |
| 1153 | IF5_HUMAN | Eukaryotic translation initiation factor 5 | P55010 | 0.46 | 0.87 | 1.13 |
| 1154 | IF5A1_HUMAN | Eukaryotic translation initiation factor 5A-1 | P63241 | 0.93 | 1.10 | 1.05 |
| 1155 | IF6_HUMAN | Eukaryotic translation initiation factor 6 | P56537 | 0.61 | 1.04 | 0.93 |
| 1156 | IFIT1_HUMAN | Interferon-induced protein with tetratricopeptide repeats 1 | P09914 |  | 0.74 | 0.94 |
| 1157 | IGBP1_HUMAN | Immunoglobulin-binding protein 1 | P78318 | 0.75 | 0.88 | 0.84 |
| 1158 | IGHA1_HUMAN | Ig alpha-1 chain C region | P01876 | 0.94 |  | 1.06 |
| 1159 | IGHA2_HUMAN | Ig alpha-2 chain C region | P01877 | 0.87 | 0.90 |  |
| 1160 | IGHG1_HUMAN | Ig gamma-1 chain C region | P01857 | 1.29 |  | 0.93 |
| 1161 | IGHG2_HUMAN | Ig gamma-2 chain C region | P01859 |  | 2.19 | 1.00 |
| 1162 | IGHG4_HUMAN | Ig gamma-4 chain C region | P01861 | 1.24 | 0.64 | 0.84 |
| 1163 | IGHM_HUMAN | Ig mu chain C region | P01871 | 0.62 | 1.12 | 0.90 |
| 1164 | IGJ_HUMAN | Immunoglobulin J chain | P01591 | 0.90 |  | 0.82 |
| 1165 | IGKC_HUMAN | Ig kappa chain C region | P01834 | 0.97 | 0.84 | 0.96 |
| 1166 | IGLL5_HUMAN | Immunoglobulin lambda-like polypeptide 5 | B9A064 | 0.72 | 1.06 | 0.94 |
| 1167 | IGSF8_HUMAN | Immunoglobulin superfamily member 8 | Q969P0 | 0.46 |  | 2.62 |
| 1168 | ILEU_HUMAN | Leukocyte elastase inhibitor | P30740 | 1.07 | 1.43 | 0.62 |
| 1169 | ILF2_HUMAN | Interleukin enhancer-binding factor 2 | Q12905 | 1.06 | 0.62 | 0.92 |
| 1170 | ILF3_HUMAN | Interleukin enhancer-binding factor 3 | Q12906 | 2.68 | 1.17 | 0.97 |
| 1171 | ILK_HUMAN | Integrin-linked protein kinase | Q13418 | 0.90 |  | 0.79 |
| 1172 | ILVBL_HUMAN | Acetolactate synthase-like protein | A1L0T0 | 1.96 | 0.41 | 1.26 |
| 1173 | IMA4_HUMAN | Importin subunit alpha-4 | O00505 | 1.01 | 2.15 | 0.90 |
| 1174 | IMA5_HUMAN | Importin subunit alpha-5 | P52294 | 0.39 | 1.43 |  |
| 1175 | IMA7_HUMAN | Importin subunit alpha-7 | O60684 | 3.60 | 1.00 | 0.83 |
| 1176 | IMB1_HUMAN | Importin subunit beta-1 | Q14974 |  |  | 0.97 |
| 1177 | IMDH2_HUMAN | Inosine-5'-monophosphate dehydrogenase 2 | P12268 | 0.87 | 2.01 | 1.11 |
| 1178 | IMPA1_HUMAN | Inositol monophosphatase 1 | P29218 | 1.14 | 0.73 | 1.06 |
| 1179 | INF2_HUMAN | Inverted formin-2 | Q27J81 |  | 1.08 | 0.91 |
| 1180 | INO1_HUMAN | Inositol-3-phosphate synthase 1 | Q9NPH2 | 1.32 | 0.98 | 0.84 |
| 1181 | INP4B_HUMAN | Type II inositol 3,4-bisphosphate 4-phosphatase | O15327 | 0.69 |  | 0.87 |
| 1182 | INPP_HUMAN | Inositol polyphosphate 1-phosphatase | P49441 | 0.98 | 1.51 | 1.01 |
| 1183 | IPKG_HUMAN | cAMP-dependent protein kinase inhibitor gamma | Q9Y2B9 | 1.02 |  | 1.06 |
| 1184 | IPO5_HUMAN | Importin-5 | O00410 | 0.26 |  | 1.01 |
| 1185 | IPO7_HUMAN | Importin-7 | O95373 | 0.77 | 0.83 | 1.08 |
| 1186 | IPSP_HUMAN | Plasma serine protease inhibitor | P05154 | 1.18 | 0.78 | 0.70 |
| 1187 | IPYR_HUMAN | Inorganic pyrophosphatase | Q15181 | 0.74 | 0.74 | 1.08 |
| 1188 | IPYR2_HUMAN | Inorganic pyrophosphatase 2, mitochondrial | Q9H2U2 |  | 0.90 | 1.52 |
| 1189 | IQGA1_HUMAN | Ras GTPase-activating-like protein IQGAP1 | P46940 | 1.49 | 0.60 | 0.97 |
| 1190 | IR3IP_HUMAN | Immediate early response 3-interacting protein 1 | Q9Y5U9 |  | 0.32 | 0.95 |
| 1191 | ISCA1_HUMAN | Iron-sulfur cluster assembly 1 homolog, mitochondrial | Q9BUE6 | 1.37 | 1.36 | 1.47 |
| 1192 | ISCA2_HUMAN | Iron-sulfur cluster assembly 2 homolog, mitochondrial | Q86U28 | 3.08 | 1.00 | 0.94 |
| 1193 | ISCU_HUMAN | Iron-sulfur cluster assembly enzyme ISCU, mitochondrial | Q9H1K1 |  | 1.12 | 0.60 |
| 1194 | ISOC1_HUMAN | Isochorismatase domain-containing protein 1 | Q96CN7 | 2.00 |  | 0.89 |
| 1195 | ISOC2_HUMAN | Isochorismatase domain-containing protein 2, mitochondrial | Q96AB3 | 1.05 | 1.20 | 0.92 |
| 1196 | IST1_HUMAN | IST1 homolog | P53990 | 0.60 | 0.61 | 0.87 |
| 1197 | ITA1_HUMAN | Integrin alpha-1 | P56199 |  | 1.09 | 1.05 |
| 1198 | ITA5_HUMAN | Integrin alpha-5 | P08648 |  | 1.94 | 0.69 |
| 1199 | ITA6_HUMAN | Integrin alpha-6 | P23229 |  | 3.22 | 1.00 |
| 1200 | ITA7_HUMAN | Integrin alpha-7 | Q13683 | 1.13 | 1.07 | 1.02 |
| 1201 | ITAV_HUMAN | Integrin alpha-V | P06756 | 1.37 | 1.07 | 0.86 |
| 1202 | ITB1_HUMAN | Integrin beta-1 | P05556 |  | 0.95 | 1.04 |
| 1203 | ITBP2_HUMAN | Integrin beta-1-binding protein 2 | Q9UKP3 | 0.77 | 0.73 | 1.60 |
| 1204 | ITIH1_HUMAN | Inter-alpha-trypsin inhibitor heavy chain H1 | P19827 | 0.74 | 0.96 | 0.89 |
| 1205 | ITIH2_HUMAN | Inter-alpha-trypsin inhibitor heavy chain H2 | P19823 |  | 1.53 | 1.02 |
| 1206 | ITIH3_HUMAN | Inter-alpha-trypsin inhibitor heavy chain H3 | Q06033 | 1.24 |  | 0.86 |
| 1207 | ITIH4_HUMAN | Inter-alpha-trypsin inhibitor heavy chain H4 | Q14624 | 0.82 | 1.34 | 1.24 |
| 1208 | ITM2B_HUMAN | Integral membrane protein 2B | Q9Y287 | 0.13 |  | 1.09 |
| 1209 | ITPA_HUMAN | Inosine triphosphate pyrophosphatase | Q9BY32 |  |  | 0.75 |
| 1210 | ITSN1_HUMAN | Intersectin-1 | Q15811 | 0.82 | 1.69 | 0.94 |
| 1211 | IVD_HUMAN | Isovaleryl-CoA dehydrogenase, mitochondrial | P26440 | 0.74 | 0.86 | 0.95 |
| 1212 | JMJD7_HUMAN | JmjC domain-containing protein 7 | P0C870 |  |  | 0.46 |
| 1213 | JPH2_HUMAN | Junctophilin-2 | Q9BR39 | 0.26 |  | 0.94 |
| 1214 | K1C10_HUMAN | Keratin, type I cytoskeletal 10 | P13645 | 1.51 | 1.33 | 1.20 |
| 1215 | K1C14_HUMAN | Keratin, type I cytoskeletal 14 | P02533 | 1.06 |  | 0.98 |
| 1216 | K1C18_HUMAN | Keratin, type I cytoskeletal 18 | P05783 | 1.77 | 0.85 | 0.98 |
| 1217 | K1C9_HUMAN | Keratin, type I cytoskeletal 9 | P35527 | 0.79 | 0.68 | 0.72 |
| 1218 | K22E_HUMAN | Keratin, type II cytoskeletal 2 epidermal | P35908 | 1.02 | 0.83 | 0.90 |
| 1219 | K2C1_HUMAN | Keratin, type II cytoskeletal 1 | P04264 | 1.26 |  | 1.08 |
| 1220 | K2C6A_HUMAN | Keratin, type II cytoskeletal 6A | P02538 | 0.90 |  | 1.15 |
| 1221 | K2C8_HUMAN | Keratin, type II cytoskeletal 8 | P05787 | 0.97 | 1.33 | 0.59 |
| 1222 | KAD1_HUMAN | Adenylate kinase isoenzyme 1 | P00568 | 0.47 |  | 0.76 |
| 1223 | KAD2_HUMAN | Adenylate kinase 2, mitochondrial | P54819 | 1.49 | 0.94 | 1.29 |
| 1224 | KAD3_HUMAN | GTP:AMP phosphotransferase AK3, mitochondrial | Q9UIJ7 | 0.78 | 0.89 | 0.97 |
| 1225 | KAD4_HUMAN | Adenylate kinase 4, mitochondrial | P27144 | 0.64 | 1.37 | 0.67 |
| 1226 | KAIN_HUMAN | Kallistatin | P29622 | 0.14 | 1.32 |  |
| 1227 | KANK1_HUMAN | KN motif and ankyrin repeat domain-containing protein 1 | Q14678 | 0.79 | 0.47 | 0.93 |
| 1228 | KANK2_HUMAN | KN motif and ankyrin repeat domain-containing protein 2 | Q63ZY3 | 1.79 | 1.05 | 1.04 |
| 1229 | KANK3_HUMAN | KN motif and ankyrin repeat domain-containing protein 3 | Q6NY19 |  | 1.00 | 0.82 |
| 1230 | KAP0_HUMAN | cAMP-dependent protein kinase type I-alpha regulatory subunit | P10644 | 1.32 | 1.46 | 0.89 |
| 1231 | KAP2_HUMAN | cAMP-dependent protein kinase type II-alpha regulatory subunit | P13861 | 1.36 | 0.74 | 1.32 |
| 1232 | KAP3_HUMAN | cAMP-dependent protein kinase type II-beta regulatory subunit | P31323 | 0.97 | 0.62 | 0.70 |
| 1233 | KAPCA_HUMAN | cAMP-dependent protein kinase catalytic subunit alpha | P17612 |  | 2.07 | 1.08 |
| 1234 | KAPCB_HUMAN | cAMP-dependent protein kinase catalytic subunit beta | P22694 | 1.16 | 0.29 | 0.57 |
| 1235 | KAT3_HUMAN | Kynurenine--oxoglutarate transaminase 3 | Q6YP21 | 1.03 | 0.96 | 0.97 |
| 1236 | KC1A_HUMAN | Casein kinase I isoform alpha | P48729 | 0.83 | 0.49 | 0.88 |
| 1237 | KC1D_HUMAN | Casein kinase I isoform delta | P48730 |  | 1.04 | 0.76 |
| 1238 | KC1E_HUMAN | Casein kinase I isoform epsilon | P49674 |  | 1.03 | 0.74 |
| 1239 | KCC2B_HUMAN | Calcium/calmodulin-dependent protein kinase type II subunit beta | Q13554 | 1.05 | 1.12 | 0.84 |
| 1240 | KCC2D_HUMAN | Calcium/calmodulin-dependent protein kinase type II subunit delta | Q13557 | 0.40 | 1.08 | 1.02 |
| 1241 | KCC2G_HUMAN | Calcium/calmodulin-dependent protein kinase type II subunit gamma | Q13555 |  | 0.86 | 0.65 |
| 1242 | KCD12_HUMAN | BTB/POZ domain-containing protein KCTD12 | Q96CX2 | 1.04 | 1.34 | 0.90 |
| 1243 | KCRB_HUMAN | Creatine kinase B-type | P12277 |  | 0.67 | 1.07 |
| 1244 | KCRM_HUMAN | Creatine kinase M-type | P06732 | 1.16 | 0.12 | 1.10 |
| 1245 | KCRS_HUMAN | Creatine kinase S-type, mitochondrial | P17540 | 1.13 |  | 0.92 |
| 1246 | KCY_HUMAN | UMP-CMP kinase | P30085 | 0.53 | 0.95 | 1.13 |
| 1247 | KDSR_HUMAN | 3-ketodihydrosphingosine reductase | Q06136 | 1.12 | 1.11 | 1.12 |
| 1248 | KGP1_HUMAN | cGMP-dependent protein kinase 1 | Q13976 |  | 1.15 | 1.14 |
| 1249 | KHDR1_HUMAN | KH domain-containing, RNA-binding, signal transduction-associated protein 1 | Q07666 |  | 1.71 | 0.74 |
| 1250 | KIF1C_HUMAN | Kinesin-like protein KIF1C | O43896 | 1.14 |  | 1.16 |
| 1251 | KINH_HUMAN | Kinesin-1 heavy chain | P33176 | 0.25 | 0.90 | 1.21 |
| 1252 | KLC1_HUMAN | Kinesin light chain 1 | Q07866 | 1.11 | 0.70 | 0.91 |
| 1253 | KLC2_HUMAN | Kinesin light chain 2 | Q9H0B6 | 0.88 |  | 1.80 |
| 1254 | KLC4_HUMAN | Kinesin light chain 4 | Q9NSK0 | 0.82 |  | 0.70 |
| 1255 | KLH31_HUMAN | Kelch-like protein 31 | Q9H511 | 0.58 | 1.06 | 1.44 |
| 1256 | KLH41_HUMAN | Kelch-like protein 41 | O60662 |  | 0.54 | 1.02 |
| 1257 | KNG1_HUMAN | Kininogen-1 | P01042 |  | 1.39 | 1.06 |
| 1258 | KPBB_HUMAN | Phosphorylase b kinase regulatory subunit beta | Q93100 | 1.05 | 2.01 | 0.91 |
| 1259 | KPCA_HUMAN | Protein kinase C alpha type | P17252 |  | 1.46 | 0.79 |
| 1260 | KPRA_HUMAN | Phosphoribosyl pyrophosphate synthase-associated protein 1 | Q14558 | 0.19 |  | 0.98 |
| 1261 | KPRB_HUMAN | Phosphoribosyl pyrophosphate synthase-associated protein 2 | O60256 |  | 0.93 | 1.13 |
| 1262 | KPYM_HUMAN | Pyruvate kinase PKM | P14618 |  |  | 1.07 |
| 1263 | KS6A3_HUMAN | Ribosomal protein S6 kinase alpha-3 | P51812 |  |  | 1.28 |
| 1264 | KT3K_HUMAN | Ketosamine-3-kinase | Q9HA64 | 0.26 |  | 0.72 |
| 1265 | KTHY_HUMAN | Thymidylate kinase | P23919 |  | 1.05 | 0.66 |
| 1266 | KTN1_HUMAN | Kinectin | Q86UP2 | 0.90 |  | 0.87 |
| 1267 | KV109_HUMAN | Ig kappa chain V-I region HK101 (Fragment) | P01601 | 0.97 |  | 1.45 |
| 1268 | KV110_HUMAN | Ig kappa chain V-I region HK102 (Fragment) | P01602 | 0.91 | 1.36 | 0.62 |
| 1269 | KV119_HUMAN | Ig kappa chain V-I region Wes | P01611 | 1.00 | 8.24 |  |
| 1270 | KV121_HUMAN | Ig kappa chain V-I region Ni | P01613 | 1.05 |  | 1.35 |
| 1271 | KV122_HUMAN | Ig kappa chain V-I region BAN | P04430 |  | 0.99 |  |
| 1272 | KV205_HUMAN | Ig kappa chain V-II region GM607 (Fragment) | P06309 |  | 1.05 |  |
| 1273 | KV309_HUMAN | Ig kappa chain V-III region VG (Fragment) | P04433 | 0.58 |  | 1.15 |
| 1274 | KV403_HUMAN | Ig kappa chain V-IV region JI | P06313 | 1.53 |  | 0.93 |
| 1275 | L2HDH_HUMAN | L-2-hydroxyglutarate dehydrogenase, mitochondrial | Q9H9P8 | 1.41 | 1.36 | 0.96 |
| 1276 | LA_HUMAN | Lupus La protein | P05455 | 1.11 | 1.46 | 0.46 |
| 1277 | LACB2_HUMAN | Beta-lactamase-like protein 2 | Q53H82 | 1.16 | 1.29 | 1.05 |
| 1278 | LACTB_HUMAN | Serine beta-lactamase-like protein LACTB, mitochondrial | P83111 | 1.22 | 0.42 | 1.11 |
| 1279 | LAMA2_HUMAN | Laminin subunit alpha-2 | P24043 | 0.91 | 1.13 | 1.10 |
| 1280 | LAMA4_HUMAN | Laminin subunit alpha-4 | Q16363 | 0.57 | 1.17 | 1.00 |
| 1281 | LAMA5_HUMAN | Laminin subunit alpha-5 | O15230 |  |  | 0.90 |
| 1282 | LAMB1_HUMAN | Laminin subunit beta-1 | P07942 | 1.22 |  | 0.84 |
| 1283 | LAMB2_HUMAN | Laminin subunit beta-2 | P55268 | 4.66 |  | 0.89 |
| 1284 | LAMC1_HUMAN | Laminin subunit gamma-1 | P11047 | 0.86 |  | 0.83 |
| 1285 | LAMC2_HUMAN | Laminin subunit gamma-2 | Q13753 | 2.31 |  | 0.71 |
| 1286 | LAMP1_HUMAN | Lysosome-associated membrane glycoprotein 1 | P11279 | 0.51 | 1.57 | 1.03 |
| 1287 | LANC1_HUMAN | LanC-like protein 1 | O43813 | 0.74 | 0.70 | 0.95 |
| 1288 | LANC2_HUMAN | LanC-like protein 2 | Q9NS86 | 1.16 | 1.24 | 1.03 |
| 1289 | LAP2B_HUMAN | Lamina-associated polypeptide 2, isoforms beta/gamma | P42167 |  | 0.73 | 0.99 |
| 1290 | LARP1_HUMAN | La-related protein 1 | Q6PKG0 |  | 1.15 | 1.07 |
| 1291 | LASP1_HUMAN | LIM and SH3 domain protein 1 | Q14847 | 1.45 |  | 0.88 |
| 1292 | LBP_HUMAN | Lipopolysaccharide-binding protein | P18428 |  | 0.94 | 0.96 |
| 1293 | LCAP_HUMAN | Leucyl-cystinyl aminopeptidase | Q9UIQ6 | 0.79 | 1.28 | 0.86 |
| 1294 | LCLT1_HUMAN | Lysocardiolipin acyltransferase 1 | Q6UWP7 | 0.81 | 1.31 | 1.06 |
| 1295 | LCMT1_HUMAN | Leucine carboxyl methyltransferase 1 | Q9UIC8 | 1.09 |  | 0.97 |
| 1296 | LDB3_HUMAN | LIM domain-binding protein 3 | O75112 | 0.95 |  | 1.12 |
| 1297 | LDHA_HUMAN | L-lactate dehydrogenase A chain | P00338 | 1.00 | 0.89 | 1.23 |
| 1298 | LDHB_HUMAN | L-lactate dehydrogenase B chain | P07195 | 1.01 | 0.65 | 1.05 |
| 1299 | LDHD_HUMAN | Probable D-lactate dehydrogenase, mitochondrial | Q86WU2 | 1.79 | 0.56 | 1.17 |
| 1300 | LEG1_HUMAN | Galectin-1 | P09382 | 0.65 |  | 0.94 |
| 1301 | LEG3_HUMAN | Galectin-3 | P17931 | 0.86 | 0.12 | 1.02 |
| 1302 | LEG8_HUMAN | Galectin-8 | O00214 | 0.70 |  | 0.62 |
| 1303 | LEMD2_HUMAN | LEM domain-containing protein 2 | Q8NC56 |  | 1.72 | 0.96 |
| 1304 | LETM1_HUMAN | LETM1 and EF-hand domain-containing protein 1, mitochondrial | O95202 | 0.56 |  | 0.63 |
| 1305 | LG3BP_HUMAN | Galectin-3-binding protein | Q08380 | 0.77 | 1.11 | 1.05 |
| 1306 | LGAT1_HUMAN | Acyl-CoA:lysophosphatidylglycerol acyltransferase 1 | Q92604 |  | 1.04 | 1.13 |
| 1307 | LGUL_HUMAN | Lactoylglutathione lyase | Q04760 |  |  | 0.50 |
| 1308 | LHPP_HUMAN | Phospholysine phosphohistidine inorganic pyrophosphate phosphatase | Q9H008 |  | 0.33 | 0.85 |
| 1309 | LIM3L_HUMAN | LIM and senescent cell antigen-like-containing domain protein 3-like | P0CW20 |  | 2.25 | 1.08 |
| 1310 | LIMC1_HUMAN | LIM and calponin homology domains-containing protein 1 | Q9UPQ0 | 2.27 | 1.82 | 1.00 |
| 1311 | LIMS1_HUMAN | LIM and senescent cell antigen-like-containing domain protein 1 | P48059 |  |  | 1.05 |
| 1312 | LIMS2_HUMAN | LIM and senescent cell antigen-like-containing domain protein 2 | Q7Z4I7 | 1.87 |  | 0.78 |
| 1313 | LIPB1_HUMAN | Liprin-beta-1 | Q86W92 | 1.37 |  | 0.97 |
| 1314 | LIPL_HUMAN | Lipoprotein lipase | P06858 | 1.05 | 1.02 | 0.80 |
| 1315 | LIPS_HUMAN | Hormone-sensitive lipase | Q05469 | 1.41 |  |  |
| 1316 | LIS1_HUMAN | Platelet-activating factor acetylhydrolase IB subunit alpha | P43034 | 0.61 |  | 0.54 |
| 1317 | LKHA4_HUMAN | Leukotriene A-4 hydrolase | P09960 |  | 0.83 | 0.82 |
| 1318 | LMA2L_HUMAN | VIP36-like protein | Q9H0V9 | 1.29 |  | 1.09 |
| 1319 | LMAN1_HUMAN | Protein ERGIC-53 | P49257 | 1.00 | 0.70 | 0.78 |
| 1320 | LMAN2_HUMAN | Vesicular integral-membrane protein VIP36 | Q12907 | 0.80 | 2.81 | 1.24 |
| 1321 | LMCD1_HUMAN | LIM and cysteine-rich domains protein 1 | Q9NZU5 | 0.82 | 1.66 | 1.19 |
| 1322 | LMF2_HUMAN | Lipase maturation factor 2 | Q9BU23 | 0.33 |  | 0.63 |
| 1323 | LMNA_HUMAN | Prelamin-A/C | P02545 |  |  | 1.21 |
| 1324 | LMNB1_HUMAN | Lamin-B1 | P20700 | 1.75 | 0.74 | 0.95 |
| 1325 | LMNB2_HUMAN | Lamin-B2 | Q03252 | 0.73 | 1.57 | 1.00 |
| 1326 | LMO7_HUMAN | LIM domain only protein 7 | Q8WWI1 | 1.31 | 0.67 | 1.05 |
| 1327 | LMOD1_HUMAN | Leiomodin-1 | P29536 | 1.51 |  | 1.06 |
| 1328 | LMOD2_HUMAN | Leiomodin-2 | Q6P5Q4 | 1.16 | 0.44 | 1.17 |
| 1329 | LNP_HUMAN | Protein lunapark | Q9C0E8 | 0.95 |  | 1.06 |
| 1330 | LONM_HUMAN | Lon protease homolog, mitochondrial | P36776 |  | 0.70 | 0.93 |
| 1331 | LPCT4_HUMAN | Lysophospholipid acyltransferase LPCAT4 | Q643R3 |  | 0.88 | 0.94 |
| 1332 | LPP_HUMAN | Lipoma-preferred partner | Q93052 | 0.75 |  | 1.04 |
| 1333 | LPPRC_HUMAN | Leucine-rich PPR motif-containing protein, mitochondrial | P42704 | 1.08 | 1.56 | 1.06 |
| 1334 | LR14B_HUMAN | Leucine-rich repeat-containing protein 14B | A6NHZ5 |  | 0.98 | 1.26 |
| 1335 | LRBA_HUMAN | Lipopolysaccharide-responsive and beige-like anchor protein | P50851 |  | 0.90 | 0.93 |
| 1336 | LRC20_HUMAN | Leucine-rich repeat-containing protein 20 | Q8TCA0 |  | 0.99 | 0.39 |
| 1337 | LRC47_HUMAN | Leucine-rich repeat-containing protein 47 | Q8N1G4 | 1.29 | 1.19 | 1.16 |
| 1338 | LRC59_HUMAN | Leucine-rich repeat-containing protein 59 | Q96AG4 | 0.93 |  | 1.19 |
| 1339 | LRP1_HUMAN | Prolow-density lipoprotein receptor-related protein 1 | Q07954 | 1.12 | 0.95 | 0.75 |
| 1340 | LRRF1_HUMAN | Leucine-rich repeat flightless-interacting protein 1 | Q32MZ4 | 0.52 | 1.08 | 0.95 |
| 1341 | LRRF2_HUMAN | Leucine-rich repeat flightless-interacting protein 2 | Q9Y608 | 1.01 | 0.80 | 1.20 |
| 1342 | LSM2_HUMAN | U6 snRNA-associated Sm-like protein LSm2 | Q9Y333 | 1.49 | 0.89 | 1.08 |
| 1343 | LSM3_HUMAN | U6 snRNA-associated Sm-like protein LSm3 | P62310 |  | 1.01 |  |
| 1344 | LSM4_HUMAN | U6 snRNA-associated Sm-like protein LSm4 | Q9Y4Z0 | 1.03 |  | 0.95 |
| 1345 | LSM5_HUMAN | U6 snRNA-associated Sm-like protein LSm5 | Q9Y4Y9 | 1.15 | 1.13 | 1.69 |
| 1346 | LSM8_HUMAN | U6 snRNA-associated Sm-like protein LSm8 | O95777 | 0.60 | 0.99 | 0.99 |
| 1347 | LTBP1_HUMAN | Latent-transforming growth factor beta-binding protein 1 | Q14766 | 0.93 |  | 1.00 |
| 1348 | LTBP2_HUMAN | Latent-transforming growth factor beta-binding protein 2 | Q14767 |  | 0.60 | 1.36 |
| 1349 | LTBP4_HUMAN | Latent-transforming growth factor beta-binding protein 4 | Q8N2S1 | 0.99 | 0.96 | 0.87 |
| 1350 | LTOR1_HUMAN | Ragulator complex protein LAMTOR1 | Q6IAA8 | 0.55 | 1.72 | 1.58 |
| 1351 | LTOR2_HUMAN | Ragulator complex protein LAMTOR2 | Q9Y2Q5 | 0.95 | 0.81 |  |
| 1352 | LTOR3_HUMAN | Ragulator complex protein LAMTOR3 | Q9UHA4 | 0.44 | 1.66 | 1.12 |
| 1353 | LTOR5_HUMAN | Ragulator complex protein LAMTOR5 | O43504 | 2.63 |  | 1.01 |
| 1354 | LUM_HUMAN | Lumican | P51884 | 0.48 |  | 1.45 |
| 1355 | LV105_HUMAN | Ig lambda chain V-I region NEWM | P01703 | 0.75 | 0.87 |  |
| 1356 | LV106_HUMAN | Ig lambda chain V-I region WAH | P04208 | 1.18 | 1.05 | 1.15 |
| 1357 | LV107_HUMAN | Ig lambda chain V-I region BL2 | P06316 |  | 0.91 | 0.52 |
| 1358 | LV302_HUMAN | Ig lambda chain V-III region LOI | P80748 | 1.87 | 0.80 | 0.66 |
| 1359 | LV403_HUMAN | Ig lambda chain V-IV region Hil | P01717 | 1.80 | 1.10 | 1.49 |
| 1360 | LYAG_HUMAN | Lysosomal alpha-glucosidase | P10253 | 0.95 | 0.74 | 0.88 |
| 1361 | LYPA1_HUMAN | Acyl-protein thioesterase 1 | O75608 | 0.49 | 1.67 | 0.97 |
| 1362 | LYPA2_HUMAN | Acyl-protein thioesterase 2 | O95372 | 0.32 | 0.75 | 0.90 |
| 1363 | LYPL1_HUMAN | Lysophospholipase-like protein 1 | Q5VWZ2 | 0.83 | 1.04 | 0.69 |
| 1364 | LYRIC_HUMAN | Protein LYRIC | Q86UE4 | 1.00 | 2.70 | 0.97 |
| 1365 | LYRM2_HUMAN | LYR motif-containing protein 2 | Q9NU23 | 1.19 |  |  |
| 1366 | LYRM4_HUMAN | LYR motif-containing protein 4 | Q9HD34 | 0.84 | 0.64 | 1.35 |
| 1367 | LYRM5_HUMAN | LYR motif-containing protein 5 | Q6IPR1 | 1.16 | 1.03 | 0.72 |
| 1368 | LYRM7_HUMAN | Complex III assembly factor LYRM7 | Q5U5X0 | 0.61 |  | 0.88 |
| 1369 | LYSC_HUMAN | Lysozyme C | P61626 | 0.96 | 0.95 |  |
| 1370 | LZIC_HUMAN | Protein LZIC | Q8WZA0 |  |  | 0.73 |
| 1371 | M2OM_HUMAN | Mitochondrial 2-oxoglutarate/malate carrier protein | Q02978 |  | 0.90 | 1.02 |
| 1372 | MA2C1_HUMAN | Alpha-mannosidase 2C1 | Q9NTJ4 |  | 1.98 | 1.20 |
| 1373 | MA7D1_HUMAN | MAP7 domain-containing protein 1 | Q3KQU3 | 1.58 | 1.07 | 1.03 |
| 1374 | MAAI_HUMAN | Maleylacetoacetate isomerase | O43708 | 1.74 | 1.64 | 1.05 |
| 1375 | MACD1_HUMAN | O-acetyl-ADP-ribose deacetylase MACROD1 | Q9BQ69 | 0.93 | 1.49 | 0.95 |
| 1376 | MACF1_HUMAN | Microtubule-actin cross-linking factor 1, isoforms 1/2/3/5 | Q9UPN3 |  | 0.57 | 1.09 |
| 1377 | MAGT1_HUMAN | Magnesium transporter protein 1 | Q9H0U3 | 1.64 | 1.11 | 0.91 |
| 1378 | MANF_HUMAN | Mesencephalic astrocyte-derived neurotrophic factor | P55145 |  | 0.81 | 0.84 |
| 1379 | MAOM_HUMAN | NAD-dependent malic enzyme, mitochondrial | P23368 | 0.77 | 1.16 | 1.00 |
| 1380 | MAON_HUMAN | NADP-dependent malic enzyme, mitochondrial | Q16798 | 0.32 | 1.00 | 0.99 |
| 1381 | MAOX_HUMAN | NADP-dependent malic enzyme | P48163 | 0.45 | 0.70 | 1.12 |
| 1382 | MAP1A_HUMAN | Microtubule-associated protein 1A | P78559 | 0.75 |  | 0.75 |
| 1383 | MAP1B_HUMAN | Microtubule-associated protein 1B | P46821 | 0.22 |  | 1.02 |
| 1384 | MAP1S_HUMAN | Microtubule-associated protein 1S | Q66K74 |  | 1.50 | 1.38 |
| 1385 | MAP2_HUMAN | Methionine aminopeptidase 2 | P50579 | 1.18 | 1.22 | 0.75 |
| 1386 | MAP4_HUMAN | Microtubule-associated protein 4 | P27816 | 1.02 | 0.90 | 1.07 |
| 1387 | MAPK2_HUMAN | MAP kinase-activated protein kinase 2 | P49137 | 1.09 |  | 1.15 |
| 1388 | MARC2_HUMAN | Mitochondrial amidoxime reducing component 2 | Q969Z3 | 1.80 | 1.79 | 1.06 |
| 1389 | MARCS_HUMAN | Myristoylated alanine-rich C-kinase substrate | P29966 | 2.73 | 0.86 | 1.00 |
| 1390 | MARE1_HUMAN | Microtubule-associated protein RP/EB family member 1 | Q15691 | 0.76 |  | 1.21 |
| 1391 | MARE2_HUMAN | Microtubule-associated protein RP/EB family member 2 | Q15555 | 0.44 | 0.44 | 1.12 |
| 1392 | MARE3_HUMAN | Microtubule-associated protein RP/EB family member 3 | Q9UPY8 | 1.66 | 0.53 | 1.23 |
| 1393 | MAT2B_HUMAN | Methionine adenosyltransferase 2 subunit beta | Q9NZL9 | 2.09 | 0.39 | 1.00 |
| 1394 | MATN2_HUMAN | Matrilin-2 | O00339 |  | 1.50 | 0.69 |
| 1395 | MATR3_HUMAN | Matrin-3 | P43243 | 1.11 |  | 1.04 |
| 1396 | MAVS_HUMAN | Mitochondrial antiviral-signaling protein | Q7Z434 | 1.19 |  | 1.01 |
| 1397 | MBLC2_HUMAN | Metallo-beta-lactamase domain-containing protein 2 | Q68D91 |  | 1.04 | 0.67 |
| 1398 | MBOA5_HUMAN | Lysophospholipid acyltransferase 5 | Q6P1A2 |  | 1.85 |  |
| 1399 | MBPHL_HUMAN | Myosin-binding protein H-like | A2RUH7 |  |  | 0.80 |
| 1400 | MCA3_HUMAN | Eukaryotic translation elongation factor 1 epsilon-1 | O43324 | 1.02 |  |  |
| 1401 | MCAT_HUMAN | Mitochondrial carnitine/acylcarnitine carrier protein | O43772 | 1.58 | 0.58 | 1.00 |
| 1402 | MCCA_HUMAN | Methylcrotonoyl-CoA carboxylase subunit alpha, mitochondrial | Q96RQ3 | 0.68 |  | 1.97 |
| 1403 | MCCB_HUMAN | Methylcrotonoyl-CoA carboxylase beta chain, mitochondrial | Q9HCC0 |  | 1.29 | 1.17 |
| 1404 | MCEE_HUMAN | Methylmalonyl-CoA epimerase, mitochondrial | Q96PE7 | 0.86 | 1.31 | 1.02 |
| 1405 | MCFD2_HUMAN | Multiple coagulation factor deficiency protein 2 | Q8NI22 | 1.01 |  |  |
| 1406 | MCTS1_HUMAN | Malignant T-cell-amplified sequence 1 | Q9ULC4 | 0.76 | 1.11 | 0.85 |
| 1407 | MDHC_HUMAN | Malate dehydrogenase, cytoplasmic | P40925 |  | 4.61 | 1.17 |
| 1408 | MDHM_HUMAN | Malate dehydrogenase, mitochondrial | P40926 | 1.10 |  | 0.88 |
| 1409 | MECP2_HUMAN | Methyl-CpG-binding protein 2 | P51608 | 1.16 | 0.96 | 0.78 |
| 1410 | MECR_HUMAN | Trans-2-enoyl-CoA reductase, mitochondrial | Q9BV79 | 0.72 | 0.69 | 1.17 |
| 1411 | MEMO1_HUMAN | Protein MEMO1 | Q9Y316 |  | 1.37 | 0.94 |
| 1412 | MEP50_HUMAN | Methylosome protein 50 | Q9BQA1 | 2.38 | 1.28 | 1.12 |
| 1413 | MESD_HUMAN | LDLR chaperone MESD | Q14696 |  | 1.80 |  |
| 1414 | MET7B_HUMAN | Methyltransferase-like protein 7B | Q6UX53 | 0.79 | 1.14 | 1.38 |
| 1415 | METH_HUMAN | Methionine synthase | Q99707 |  | 2.31 | 1.32 |
| 1416 | METK2_HUMAN | S-adenosylmethionine synthase isoform type-2 | P31153 | 0.37 | 0.90 | 0.94 |
| 1417 | MFAP4_HUMAN | Microfibril-associated glycoprotein 4 | P55083 | 0.84 | 1.57 | 0.90 |
| 1418 | MFAP5_HUMAN | Microfibrillar-associated protein 5 | Q13361 | 1.13 | 0.64 | 0.63 |
| 1419 | MFF_HUMAN | Mitochondrial fission factor | Q9GZY8 | 1.25 | 0.81 | 0.96 |
| 1420 | MFGM_HUMAN | Lactadherin | Q08431 | 0.77 | 0.06 | 0.67 |
| 1421 | MFN1_HUMAN | Mitofusin-1 | Q8IWA4 | 1.39 | 1.24 | 0.60 |
| 1422 | MFN2_HUMAN | Mitofusin-2 | O95140 |  | 1.12 | 1.20 |
| 1423 | MFR1L_HUMAN | Mitochondrial fission regulator 1-like | Q9H019 | 1.64 | 0.74 | 0.79 |
| 1424 | MGDP1_HUMAN | Magnesium-dependent phosphatase 1 | Q86V88 | 0.68 |  | 1.54 |
| 1425 | MGLL_HUMAN | Monoglyceride lipase | Q99685 | 0.90 | 0.82 | 1.35 |
| 1426 | MGP_HUMAN | Matrix Gla protein | P08493 | 1.11 |  | 2.82 |
| 1427 | MGST1_HUMAN | Microsomal glutathione S-transferase 1 | P10620 | 1.39 |  | 0.91 |
| 1428 | MGST2_HUMAN | Microsomal glutathione S-transferase 2 | Q99735 | 1.69 |  | 0.82 |
| 1429 | MGST3_HUMAN | Microsomal glutathione S-transferase 3 | O14880 | 0.89 | 1.46 | 0.80 |
| 1430 | MIA40_HUMAN | Mitochondrial intermembrane space import and assembly protein 40 | Q8N4Q1 | 0.57 | 0.76 | 0.84 |
| 1431 | MIC19_HUMAN | MICOS complex subunit MIC19 | Q9NX63 | 0.95 |  | 1.00 |
| 1432 | MIC27_HUMAN | MICOS complex subunit MIC27 | Q6UXV4 |  |  | 0.98 |
| 1433 | MIC60_HUMAN | MICOS complex subunit MIC60 | Q16891 | 0.76 |  | 1.04 |
| 1434 | MID49_HUMAN | Mitochondrial dynamics protein MID49 | Q96C03 | 0.80 |  | 1.14 |
| 1435 | MIME_HUMAN | Mimecan | P20774 | 0.16 |  | 1.02 |
| 1436 | MIMIT_HUMAN | Mimitin, mitochondrial | Q8N183 | 0.10 | 0.90 | 0.96 |
| 1437 | MIPEP_HUMAN | Mitochondrial intermediate peptidase | Q99797 | 0.18 |  | 0.97 |
| 1438 | MIRO1_HUMAN | Mitochondrial Rho GTPase 1 | Q8IXI2 | 1.19 |  | 0.88 |
| 1439 | MIRO2_HUMAN | Mitochondrial Rho GTPase 2 | Q8IXI1 |  | 0.84 | 1.19 |
| 1440 | MK01_HUMAN | Mitogen-activated protein kinase 1 | P28482 | 0.67 | 1.69 | 0.93 |
| 1441 | MK03_HUMAN | Mitogen-activated protein kinase 3 | P27361 |  | 2.17 | 0.89 |
| 1442 | MK14_HUMAN | Mitogen-activated protein kinase 14 | Q16539 | 0.84 | 0.77 | 0.80 |
| 1443 | ML12A_HUMAN | Myosin regulatory light chain 12A | P19105 | 1.12 | 1.92 | 1.45 |
| 1444 | MLEC_HUMAN | Malectin | Q14165 | 0.40 | 1.02 | 1.23 |
| 1445 | MLIP_HUMAN | Muscular LMNA-interacting protein | Q5VWP3 |  |  | 0.84 |
| 1446 | MLP3A_HUMAN | Microtubule-associated proteins 1A/1B light chain 3A | Q9H492 |  | 0.33 | 0.55 |
| 1447 | MLRA_HUMAN | Myosin regulatory light chain 2, atrial isoform | Q01449 | 1.63 | 1.17 | 1.18 |
| 1448 | MLRV_HUMAN | Myosin regulatory light chain 2, ventricular/cardiac muscle isoform | P10916 | 0.96 |  | 0.90 |
| 1449 | MLTK_HUMAN | Mitogen-activated protein kinase kinase kinase MLT | Q9NYL2 |  | 0.87 | 1.15 |
| 1450 | MMAA_HUMAN | Methylmalonic aciduria type A protein, mitochondrial | Q8IVH4 | 1.63 | 0.59 | 1.52 |
| 1451 | MMAB_HUMAN | Cob(I)yrinic acid a,c-diamide adenosyltransferase, mitochondrial | Q96EY8 | 1.24 | 1.26 | 1.16 |
| 1452 | MMRN2_HUMAN | Multimerin-2 | Q9H8L6 | 0.95 | 0.66 | 1.33 |
| 1453 | MMSA_HUMAN | Methylmalonate-semialdehyde dehydrogenase [acylating], mitochondrial | Q02252 | 0.74 | 1.14 | 1.32 |
| 1454 | MOB1A_HUMAN | MOB kinase activator 1A | Q9H8S9 | 1.34 |  | 0.85 |
| 1455 | MOC2A_HUMAN | Molybdopterin synthase sulfur carrier subunit | O96033 | 1.03 | 0.81 | 0.68 |
| 1456 | MOC2B_HUMAN | Molybdopterin synthase catalytic subunit | O96007 |  | 0.85 | 0.91 |
| 1457 | MOCS1_HUMAN | Molybdenum cofactor biosynthesis protein 1 | Q9NZB8 |  | 1.31 | 0.60 |
| 1458 | MOES_HUMAN | Moesin | P26038 | 0.87 |  | 0.98 |
| 1459 | MOGS_HUMAN | Mannosyl-oligosaccharide glucosidase | Q13724 | 0.85 | 1.04 | 1.17 |
| 1460 | MOT1_HUMAN | Monocarboxylate transporter 1 | P53985 | 0.98 | 0.79 | 0.82 |
| 1461 | MOT2_HUMAN | Monocarboxylate transporter 2 | O60669 | 1.09 | 1.10 | 0.66 |
| 1462 | MP2K1_HUMAN | Dual specificity mitogen-activated protein kinase kinase 1 | Q02750 |  | 0.74 |  |
| 1463 | MP2K2_HUMAN | Dual specificity mitogen-activated protein kinase kinase 2 | P36507 | 0.34 | 0.75 | 1.15 |
| 1464 | MP2K4_HUMAN | Dual specificity mitogen-activated protein kinase kinase 4 | P45985 | 0.76 |  | 1.12 |
| 1465 | MPC1_HUMAN | Mitochondrial pyruvate carrier 1 | Q9Y5U8 | 1.05 | 1.45 | 1.01 |
| 1466 | MPC2_HUMAN | Mitochondrial pyruvate carrier 2 | O95563 | 0.99 | 1.01 | 1.04 |
| 1467 | MPCP_HUMAN | Phosphate carrier protein, mitochondrial | Q00325 |  | 1.04 | 1.04 |
| 1468 | MPI_HUMAN | Mannose-6-phosphate isomerase | P34949 | 1.00 | 1.07 | 1.44 |
| 1469 | MPPA_HUMAN | Mitochondrial-processing peptidase subunit alpha | Q10713 | 0.90 |  | 0.70 |
| 1470 | MPPB_HUMAN | Mitochondrial-processing peptidase subunit beta | O75439 | 0.72 | 1.22 | 0.61 |
| 1471 | MPRD_HUMAN | Cation-dependent mannose-6-phosphate receptor | P20645 | 0.65 | 1.14 |  |
| 1472 | MPV17_HUMAN | Protein Mpv17 | P39210 | 0.62 | 1.21 | 0.86 |
| 1473 | MRRP1_HUMAN | Mitochondrial ribonuclease P protein 1 | Q7L0Y3 | 1.82 |  | 0.97 |
| 1474 | MSI2H_HUMAN | RNA-binding protein Musashi homolog 2 | Q96DH6 | 0.97 | 1.14 | 0.67 |
| 1475 | MSRA_HUMAN | Mitochondrial peptide methionine sulfoxide reductase | Q9UJ68 | 2.36 |  | 0.78 |
| 1476 | MSRB2_HUMAN | Methionine-R-sulfoxide reductase B2, mitochondrial | Q9Y3D2 | 0.59 | 1.27 | 0.86 |
| 1477 | MTAP_HUMAN | S-methyl-5'-thioadenosine phosphorylase | Q13126 | 0.85 | 0.86 | 0.88 |
| 1478 | MTCH1_HUMAN | Mitochondrial carrier homolog 1 | Q9NZJ7 | 1.15 |  | 1.27 |
| 1479 | MTCH2_HUMAN | Mitochondrial carrier homolog 2 | Q9Y6C9 | 0.90 | 1.37 | 0.88 |
| 1480 | MTD2L_HUMAN | Probable bifunctional methylenetetrahydrofolate dehydrogenase/cyclohydrolase 2 | Q9H903 |  | 1.02 | 1.36 |
| 1481 | MTFP1_HUMAN | Mitochondrial fission process protein 1 | Q9UDX5 | 0.90 | 1.13 | 0.71 |
| 1482 | MTMR6_HUMAN | Myotubularin-related protein 6 | Q9Y217 | 1.05 |  | 0.43 |
| 1483 | MTMR9_HUMAN | Myotubularin-related protein 9 | Q96QG7 |  | 0.87 | 0.73 |
| 1484 | MTND_HUMAN | 1,2-dihydroxy-3-keto-5-methylthiopentene dioxygenase | Q9BV57 |  | 0.96 | 0.95 |
| 1485 | MTOR_HUMAN | Serine/threonine-protein kinase mTOR | P42345 | 0.72 | 0.89 | 1.38 |
| 1486 | MTPN_HUMAN | Myotrophin | P58546 | 0.82 | 0.69 | 0.78 |
| 1487 | MTU1_HUMAN | Mitochondrial tRNA-specific 2-thiouridylase 1 | O75648 |  | 0.93 | 2.02 |
| 1488 | MTUS2_HUMAN | Microtubule-associated tumor suppressor candidate 2 | Q5JR59 |  | 0.63 |  |
| 1489 | MTX1_HUMAN | Metaxin-1 | Q13505 | 1.15 | 1.25 | 1.03 |
| 1490 | MTX2_HUMAN | Metaxin-2 | O75431 | 1.03 | 0.72 | 1.49 |
| 1491 | MUC18_HUMAN | Cell surface glycoprotein MUC18 | P43121 | 0.54 | 1.02 | 1.17 |
| 1492 | MUL1_HUMAN | Mitochondrial ubiquitin ligase activator of NFKB 1 | Q969V5 |  | 0.52 | 0.76 |
| 1493 | MURC_HUMAN | Muscle-related coiled-coil protein | Q5BKX8 | 1.18 | 0.57 | 0.86 |
| 1494 | MUTA_HUMAN | Methylmalonyl-CoA mutase, mitochondrial | P22033 | 0.59 | 1.32 | 1.04 |
| 1495 | MVP_HUMAN | Major vault protein | Q14764 |  | 0.73 | 0.86 |
| 1496 | MXRA7_HUMAN | Matrix-remodeling-associated protein 7 | P84157 |  | 1.96 | 0.93 |
| 1497 | MY18A_HUMAN | Unconventional myosin-XVIIIa | Q92614 | 1.17 |  | 1.02 |
| 1498 | MY18B_HUMAN | Unconventional myosin-XVIIIb | Q8IUG5 | 1.21 |  | 0.93 |
| 1499 | MYADM_HUMAN | Myeloid-associated differentiation marker | Q96S97 |  | 2.40 | 1.03 |
| 1500 | MYG_HUMAN | Myoglobin | P02144 | 1.15 |  | 1.20 |
| 1501 | MYH1_HUMAN | Myosin-1 | P12882 |  |  | 0.87 |
| 1502 | MYH10_HUMAN | Myosin-10 | P35580 | 0.94 | 1.96 | 0.86 |
| 1503 | MYH11_HUMAN | Myosin-11 | P35749 |  | 1.37 | 0.62 |
| 1504 | MYH13_HUMAN | Myosin-13 | Q9UKX3 | 1.60 |  | 0.95 |
| 1505 | MYH14_HUMAN | Myosin-14 | Q7Z406 | 0.64 | 0.91 | 0.76 |
| 1506 | MYH3_HUMAN | Myosin-3 | P11055 | 0.33 | 0.78 | 0.98 |
| 1507 | MYH4_HUMAN | Myosin-4 | Q9Y623 |  | 1.42 | 1.07 |
| 1508 | MYH6_HUMAN | Myosin-6 | P13533 | 0.56 | 0.99 | 0.88 |
| 1509 | MYH7_HUMAN | Myosin-7 | P12883 |  | 0.96 | 0.88 |
| 1510 | MYH7B_HUMAN | Myosin-7B | A7E2Y1 | 0.86 | 0.52 | 0.73 |
| 1511 | MYH8_HUMAN | Myosin-8 | P13535 | 0.09 | 1.67 | 1.06 |
| 1512 | MYH9_HUMAN | Myosin-9 | P35579 |  | 1.18 | 0.99 |
| 1513 | MYL3_HUMAN | Myosin light chain 3 | P08590 | 4.45 |  | 0.71 |
| 1514 | MYL4_HUMAN | Myosin light chain 4 | P12829 | 1.07 |  | 1.13 |
| 1515 | MYL6_HUMAN | Myosin light polypeptide 6 | P60660 | 0.64 | 1.01 | 0.85 |
| 1516 | MYL9_HUMAN | Myosin regulatory light polypeptide 9 | P24844 | 1.33 |  | 0.63 |
| 1517 | MYLK_HUMAN | Myosin light chain kinase, smooth muscle | Q15746 | 0.65 | 0.74 | 0.87 |
| 1518 | MYLK3_HUMAN | Myosin light chain kinase 3 | Q32MK0 | 1.17 |  | 1.01 |
| 1519 | MYO1B_HUMAN | Unconventional myosin-Ib | O43795 |  | 1.11 | 1.05 |
| 1520 | MYO1C_HUMAN | Unconventional myosin-Ic | O00159 | 1.05 | 1.91 | 1.13 |
| 1521 | MYO1D_HUMAN | Unconventional myosin-Id | O94832 | 0.90 | 1.85 |  |
| 1522 | MYOC_HUMAN | Myocilin | Q99972 |  | 0.67 | 0.97 |
| 1523 | MYOF_HUMAN | Myoferlin | Q9NZM1 |  | 1.15 | 0.39 |
| 1524 | MYOM1_HUMAN | Myomesin-1 | P52179 |  | 0.96 | 0.82 |
| 1525 | MYOM2_HUMAN | Myomesin-2 | P54296 |  | 1.21 | 0.95 |
| 1526 | MYOM3_HUMAN | Myomesin-3 | Q5VTT5 | 0.56 | 1.05 | 1.03 |
| 1527 | MYOTI_HUMAN | Myotilin | Q9UBF9 | 1.04 | 0.85 | 0.93 |
| 1528 | MYOZ1_HUMAN | Myozenin-1 | Q9NP98 |  | 1.07 | 1.15 |
| 1529 | MYOZ2_HUMAN | Myozenin-2 | Q9NPC6 | 1.18 | 1.16 | 0.81 |
| 1530 | MYP0_HUMAN | Myelin protein P0 | P25189 | 1.20 | 1.46 | 0.79 |
| 1531 | MYPC3_HUMAN | Myosin-binding protein C, cardiac-type | Q14896 |  | 0.89 | 0.94 |
| 1532 | MYPN_HUMAN | Myopalladin | Q86TC9 | 0.54 | 0.74 | 0.96 |
| 1533 | MYPT1_HUMAN | Protein phosphatase 1 regulatory subunit 12A | O14974 | 1.91 | 0.77 | 0.96 |
| 1534 | MYPT2_HUMAN | Protein phosphatase 1 regulatory subunit 12B | O60237 | 0.97 |  | 0.95 |
| 1535 | MYZAP_HUMAN | Myocardial zonula adherens protein | P0CAP1 | 1.25 |  | 0.96 |
| 1536 | NAA15_HUMAN | N-alpha-acetyltransferase 15, NatA auxiliary subunit | Q9BXJ9 |  | 1.04 | 0.64 |
| 1537 | NAA25_HUMAN | N-alpha-acetyltransferase 25, NatB auxiliary subunit | Q14CX7 | 0.31 | 1.45 | 0.63 |
| 1538 | NAA35_HUMAN | N-alpha-acetyltransferase 35, NatC auxiliary subunit | Q5VZE5 | 0.74 |  | 1.02 |
| 1539 | NAC1_HUMAN | Sodium/calcium exchanger 1 | P32418 |  | 0.75 | 0.86 |
| 1540 | NACAM_HUMAN | Nascent polypeptide-associated complex subunit alpha, muscle-specific form | E9PAV3 | 1.08 |  | 1.25 |
| 1541 | NADC_HUMAN | Nicotinate-nucleotide pyrophosphorylase [carboxylating] | Q15274 |  | 1.02 | 1.28 |
| 1542 | NAGK_HUMAN | N-acetyl-D-glucosamine kinase | Q9UJ70 | 0.25 | 4.83 |  |
| 1543 | NAKD2_HUMAN | NAD kinase 2, mitochondrial | Q4G0N4 | 2.21 | 1.36 | 0.90 |
| 1544 | NAMPT_HUMAN | Nicotinamide phosphoribosyltransferase | P43490 | 0.84 | 1.34 | 1.09 |
| 1545 | NAR3_HUMAN | Ecto-ADP-ribosyltransferase 3 | Q13508 |  | 1.37 | 0.98 |
| 1546 | NB5R1_HUMAN | NADH-cytochrome b5 reductase 1 | Q9UHQ9 | 1.05 |  | 1.08 |
| 1547 | NB5R3_HUMAN | NADH-cytochrome b5 reductase 3 | P00387 | 0.55 | 1.18 | 1.07 |
| 1548 | NBAS_HUMAN | Neuroblastoma-amplified sequence | A2RRP1 | 0.72 |  | 5.04 |
| 1549 | NCALD_HUMAN | Neurocalcin-delta | P61601 |  | 1.08 | 0.78 |
| 1550 | NCAM1_HUMAN | Neural cell adhesion molecule 1 | P13591 | 0.64 |  | 1.24 |
| 1551 | NCEH1_HUMAN | Neutral cholesterol ester hydrolase 1 | Q6PIU2 | 0.87 | 0.68 | 0.90 |
| 1552 | NCKP1_HUMAN | Nck-associated protein 1 | Q9Y2A7 |  | 1.17 | 0.95 |
| 1553 | NCLN_HUMAN | Nicalin | Q969V3 |  | 2.78 | 0.69 |
| 1554 | NCPR_HUMAN | NADPH--cytochrome P450 reductase | P16435 | 0.79 |  | 0.44 |
| 1555 | NDK3_HUMAN | Nucleoside diphosphate kinase 3 | Q13232 | 1.38 | 1.26 | 1.04 |
| 1556 | NDKA_HUMAN | Nucleoside diphosphate kinase A | P15531 | 1.37 |  | 1.07 |
| 1557 | NDKB_HUMAN | Nucleoside diphosphate kinase B | P22392 |  | 1.50 | 1.10 |
| 1558 | NDRG1_HUMAN | Protein NDRG1 | Q92597 | 1.11 |  | 0.99 |
| 1559 | NDRG2_HUMAN | Protein NDRG2 | Q9UN36 |  | 1.15 | 0.96 |
| 1560 | NDRG4_HUMAN | Protein NDRG4 | Q9ULP0 | 1.10 | 0.85 | 1.02 |
| 1561 | NDUA1_HUMAN | NADH dehydrogenase [ubiquinone] 1 alpha subcomplex subunit 1 | O15239 | 1.49 | 1.03 | 1.32 |
| 1562 | NDUA2_HUMAN | NADH dehydrogenase [ubiquinone] 1 alpha subcomplex subunit 2 | O43678 | 0.59 | 0.81 | 0.98 |
| 1563 | NDUA3_HUMAN | NADH dehydrogenase [ubiquinone] 1 alpha subcomplex subunit 3 | O95167 | 0.82 | 1.46 | 1.08 |
| 1564 | NDUA4_HUMAN | Cytochrome c oxidase subunit NDUFA4 | O00483 |  |  | 1.26 |
| 1565 | NDUA5_HUMAN | NADH dehydrogenase [ubiquinone] 1 alpha subcomplex subunit 5 | Q16718 | 1.71 | 1.50 | 0.80 |
| 1566 | NDUA6_HUMAN | NADH dehydrogenase [ubiquinone] 1 alpha subcomplex subunit 6 | P56556 | 1.18 | 1.19 | 1.04 |
| 1567 | NDUA7_HUMAN | NADH dehydrogenase [ubiquinone] 1 alpha subcomplex subunit 7 | O95182 | 0.51 |  | 1.05 |
| 1568 | NDUA8_HUMAN | NADH dehydrogenase [ubiquinone] 1 alpha subcomplex subunit 8 | P51970 | 1.05 |  | 0.91 |
| 1569 | NDUA9_HUMAN | NADH dehydrogenase [ubiquinone] 1 alpha subcomplex subunit 9, mitochondrial | Q16795 |  | 0.68 | 2.26 |
| 1570 | NDUAA_HUMAN | NADH dehydrogenase [ubiquinone] 1 alpha subcomplex subunit 10, mitochondrial | O95299 | 0.98 | 1.19 | 1.01 |
| 1571 | NDUAB_HUMAN | NADH dehydrogenase [ubiquinone] 1 alpha subcomplex subunit 11 | Q86Y39 | 1.07 | 1.19 | 0.99 |
| 1572 | NDUAC_HUMAN | NADH dehydrogenase [ubiquinone] 1 alpha subcomplex subunit 12 | Q9UI09 | 1.18 | 1.74 | 1.09 |
| 1573 | NDUAD_HUMAN | NADH dehydrogenase [ubiquinone] 1 alpha subcomplex subunit 13 | Q9P0J0 |  | 0.80 | 0.89 |
| 1574 | NDUB1_HUMAN | NADH dehydrogenase [ubiquinone] 1 beta subcomplex subunit 1 | O75438 | 0.87 | 2.23 | 1.49 |
| 1575 | NDUB2_HUMAN | NADH dehydrogenase [ubiquinone] 1 beta subcomplex subunit 2, mitochondrial | O95178 | 1.04 | 0.90 | 0.83 |
| 1576 | NDUB3_HUMAN | NADH dehydrogenase [ubiquinone] 1 beta subcomplex subunit 3 | O43676 | 0.66 | 2.23 | 1.00 |
| 1577 | NDUB4_HUMAN | NADH dehydrogenase [ubiquinone] 1 beta subcomplex subunit 4 | O95168 | 0.35 | 0.98 | 1.14 |
| 1578 | NDUB5_HUMAN | NADH dehydrogenase [ubiquinone] 1 beta subcomplex subunit 5, mitochondrial | O43674 | 0.78 |  | 1.03 |
| 1579 | NDUB6_HUMAN | NADH dehydrogenase [ubiquinone] 1 beta subcomplex subunit 6 | O95139 | 1.74 | 0.69 | 1.03 |
| 1580 | NDUB7_HUMAN | NADH dehydrogenase [ubiquinone] 1 beta subcomplex subunit 7 | P17568 | 3.98 | 1.19 | 0.99 |
| 1581 | NDUB8_HUMAN | NADH dehydrogenase [ubiquinone] 1 beta subcomplex subunit 8, mitochondrial | O95169 | 1.41 | 1.84 | 0.96 |
| 1582 | NDUB9_HUMAN | NADH dehydrogenase [ubiquinone] 1 beta subcomplex subunit 9 | Q9Y6M9 | 0.41 | 1.03 | 0.80 |
| 1583 | NDUBA_HUMAN | NADH dehydrogenase [ubiquinone] 1 beta subcomplex subunit 10 | O96000 | 1.00 | 1.16 | 0.99 |
| 1584 | NDUBB_HUMAN | NADH dehydrogenase [ubiquinone] 1 beta subcomplex subunit 11, mitochondrial | Q9NX14 | 0.95 |  | 0.90 |
| 1585 | NDUC1_HUMAN | NADH dehydrogenase [ubiquinone] 1 subunit C1, mitochondrial | O43677 | 0.39 | 0.90 | 1.19 |
| 1586 | NDUC2_HUMAN | NADH dehydrogenase [ubiquinone] 1 subunit C2 | O95298 | 0.94 | 1.04 | 1.06 |
| 1587 | NDUF3_HUMAN | NADH dehydrogenase [ubiquinone] 1 alpha subcomplex assembly factor 3 | Q9BU61 | 1.18 | 1.04 | 0.88 |
| 1588 | NDUF4_HUMAN | NADH dehydrogenase [ubiquinone] 1 alpha subcomplex assembly factor 4 | Q9P032 | 0.95 |  | 0.68 |
| 1589 | NDUF5_HUMAN | NADH dehydrogenase [ubiquinone] 1 alpha subcomplex assembly factor 5 | Q5TEU4 | 0.93 | 1.16 | 0.90 |
| 1590 | NDUF6_HUMAN | NADH dehydrogenase (ubiquinone) complex I, assembly factor 6 | Q330K2 | 0.32 | 0.47 | 0.93 |
| 1591 | NDUF7_HUMAN | NADH dehydrogenase [ubiquinone] complex I, assembly factor 7 | Q7L592 |  | 1.20 | 1.26 |
| 1592 | NDUS1_HUMAN | NADH-ubiquinone oxidoreductase 75 kDa subunit, mitochondrial | P28331 |  |  | 1.14 |
| 1593 | NDUS2_HUMAN | NADH dehydrogenase [ubiquinone] iron-sulfur protein 2, mitochondrial | O75306 | 1.34 | 1.04 | 1.07 |
| 1594 | NDUS3_HUMAN | NADH dehydrogenase [ubiquinone] iron-sulfur protein 3, mitochondrial | O75489 | 1.33 | 0.82 | 0.98 |
| 1595 | NDUS4_HUMAN | NADH dehydrogenase [ubiquinone] iron-sulfur protein 4, mitochondrial | O43181 |  | 0.54 | 1.05 |
| 1596 | NDUS5_HUMAN | NADH dehydrogenase [ubiquinone] iron-sulfur protein 5 | O43920 | 1.17 | 0.92 | 0.76 |
| 1597 | NDUS6_HUMAN | NADH dehydrogenase [ubiquinone] iron-sulfur protein 6, mitochondrial | O75380 | 1.37 | 0.65 | 0.85 |
| 1598 | NDUS7_HUMAN | NADH dehydrogenase [ubiquinone] iron-sulfur protein 7, mitochondrial | O75251 | 0.28 | 1.15 | 0.95 |
| 1599 | NDUS8_HUMAN | NADH dehydrogenase [ubiquinone] iron-sulfur protein 8, mitochondrial | O00217 | 0.66 | 1.13 | 0.96 |
| 1600 | NDUV1_HUMAN | NADH dehydrogenase [ubiquinone] flavoprotein 1, mitochondrial | P49821 | 0.95 |  | 0.94 |
| 1601 | NDUV2_HUMAN | NADH dehydrogenase [ubiquinone] flavoprotein 2, mitochondrial | P19404 | 1.18 | 0.69 | 0.86 |
| 1602 | NDUV3_HUMAN | NADH dehydrogenase [ubiquinone] flavoprotein 3, mitochondrial | P56181 |  | 3.37 | 0.76 |
| 1603 | NEBL_HUMAN | Nebulette | O76041 |  |  | 0.94 |
| 1604 | NEDD8_HUMAN | NEDD8 | Q15843 | 1.17 | 1.43 | 0.83 |
| 1605 | NEK7_HUMAN | Serine/threonine-protein kinase Nek7 | Q8TDX7 | 1.03 | 0.75 | 0.85 |
| 1606 | NEK9_HUMAN | Serine/threonine-protein kinase Nek9 | Q8TD19 | 0.27 | 1.14 | 1.55 |
| 1607 | NENF_HUMAN | Neudesin | Q9UMX5 | 0.92 | 1.85 | 0.65 |
| 1608 | NEST_HUMAN | Nestin | P48681 | 0.98 |  | 0.99 |
| 1609 | NET1_HUMAN | Netrin-1 | O95631 | 1.45 |  |  |
| 1610 | NEUL_HUMAN | Neurolysin, mitochondrial | Q9BYT8 |  | 0.65 | 0.81 |
| 1611 | NEXN_HUMAN | Nexilin | Q0ZGT2 | 1.27 | 1.10 | 0.91 |
| 1612 | NFKB1_HUMAN | Nuclear factor NF-kappa-B p105 subunit | P19838 | 0.89 |  | 0.85 |
| 1613 | NFS1_HUMAN | Cysteine desulfurase, mitochondrial | Q9Y697 | 0.33 | 0.55 | 0.86 |
| 1614 | NFU1_HUMAN | NFU1 iron-sulfur cluster scaffold homolog, mitochondrial | Q9UMS0 | 0.98 | 1.02 | 1.19 |
| 1615 | NH2L1_HUMAN | NHP2-like protein 1 | P55769 | 0.67 | 0.43 | 1.05 |
| 1616 | NHLC2_HUMAN | NHL repeat-containing protein 2 | Q8NBF2 | 0.93 | 1.37 | 0.93 |
| 1617 | NHRF2_HUMAN | Na(+)/H(+) exchange regulatory cofactor NHE-RF2 | Q15599 |  | 1.91 | 1.12 |
| 1618 | NIBAN_HUMAN | Protein Niban | Q9BZQ8 | 0.44 | 0.40 | 1.03 |
| 1619 | NIBL1_HUMAN | Niban-like protein 1 | Q96TA1 |  |  | 0.69 |
| 1620 | NID1_HUMAN | Nidogen-1 | P14543 | 0.70 |  | 0.96 |
| 1621 | NID2_HUMAN | Nidogen-2 | Q14112 | 0.35 |  | 0.87 |
| 1622 | NIPS1_HUMAN | Protein NipSnap homolog 1 | Q9BPW8 | 1.11 | 1.21 | 0.68 |
| 1623 | NIPS2_HUMAN | Protein NipSnap homolog 2 | O75323 | 0.99 | 1.25 | 0.80 |
| 1624 | NIT1_HUMAN | Nitrilase homolog 1 | Q86X76 | 2.11 | 0.97 | 1.18 |
| 1625 | NIT2_HUMAN | Omega-amidase NIT2 | Q9NQR4 | 1.12 | 0.97 | 1.10 |
| 1626 | NLRX1_HUMAN | NLR family member X1 | Q86UT6 | 1.01 | 0.82 | 1.22 |
| 1627 | NLTP_HUMAN | Non-specific lipid-transfer protein | P22307 | 1.21 | 1.64 | 1.41 |
| 1628 | NMNA3_HUMAN | Nicotinamide mononucleotide adenylyltransferase 3 | Q96T66 | 0.33 | 0.81 | 1.21 |
| 1629 | NMT1_HUMAN | Glycylpeptide N-tetradecanoyltransferase 1 | P30419 | 0.28 | 1.15 | 1.24 |
| 1630 | NNRD_HUMAN | ATP-dependent (S)-NAD(P)H-hydrate dehydratase | Q8IW45 | 0.78 |  | 0.94 |
| 1631 | NNRE_HUMAN | NAD(P)H-hydrate epimerase | Q8NCW5 | 1.54 | 2.31 | 0.78 |
| 1632 | NNTM_HUMAN | NAD(P) transhydrogenase, mitochondrial | Q13423 |  |  | 0.81 |
| 1633 | NOL3_HUMAN | Nucleolar protein 3 | O60936 | 1.14 | 0.94 | 0.80 |
| 1634 | NOLC1_HUMAN | Nucleolar and coiled-body phosphoprotein 1 | Q14978 | 0.78 |  |  |
| 1635 | NONO_HUMAN | Non-POU domain-containing octamer-binding protein | Q15233 | 1.15 | 0.66 | 1.61 |
| 1636 | NOP56_HUMAN | Nucleolar protein 56 | O00567 |  | 1.03 | 0.92 |
| 1637 | NOP58_HUMAN | Nucleolar protein 58 | Q9Y2X3 |  | 1.29 | 0.95 |
| 1638 | NP1L1_HUMAN | Nucleosome assembly protein 1-like 1 | P55209 | 1.18 | 1.02 | 1.10 |
| 1639 | NP1L4_HUMAN | Nucleosome assembly protein 1-like 4 | Q99733 | 3.05 | 1.54 | 1.14 |
| 1640 | NPC2_HUMAN | Epididymal secretory protein E1 | P61916 | 1.10 |  | 0.19 |
| 1641 | NPL4_HUMAN | Nuclear protein localization protein 4 homolog | Q8TAT6 |  | 0.70 | 1.17 |
| 1642 | NPM_HUMAN | Nucleophosmin | P06748 | 0.88 | 0.90 | 1.27 |
| 1643 | NPS3A_HUMAN | Protein NipSnap homolog 3A | Q9UFN0 | 0.68 | 1.21 | 1.31 |
| 1644 | NPS3B_HUMAN | Protein NipSnap homolog 3B | Q9BS92 | 1.49 | 0.72 | 1.23 |
| 1645 | NPTN_HUMAN | Neuroplastin | Q9Y639 | 0.82 | 1.04 | 0.84 |
| 1646 | NQO1_HUMAN | NAD(P)H dehydrogenase [quinone] 1 | P15559 | 0.27 |  | 1.15 |
| 1647 | NQO2_HUMAN | Ribosyldihydronicotinamide dehydrogenase [quinone] | P16083 | 0.85 | 2.70 | 1.08 |
| 1648 | NRAP_HUMAN | Nebulin-related-anchoring protein | Q86VF7 | 0.97 | 1.28 | 1.03 |
| 1649 | NRP1_HUMAN | Neuropilin-1 | O14786 | 0.72 |  | 0.88 |
| 1650 | NSDHL_HUMAN | Sterol-4-alpha-carboxylate 3-dehydrogenase, decarboxylating | Q15738 | 15.28 | 1.05 | 1.03 |
| 1651 | NSF_HUMAN | Vesicle-fusing ATPase | P46459 | 0.83 | 0.96 | 1.02 |
| 1652 | NSF1C_HUMAN | NSFL1 cofactor p47 | Q9UNZ2 | 0.91 | 0.36 | 1.03 |
| 1653 | NSUN2_HUMAN | tRNA (cytosine(34)-C(5))-methyltransferase | Q08J23 |  | 0.94 | 0.84 |
| 1654 | NT5C_HUMAN | 5'(3')-deoxyribonucleotidase, cytosolic type | Q8TCD5 | 0.92 | 1.25 | 1.27 |
| 1655 | NTF2_HUMAN | Nuclear transport factor 2 | P61970 | 0.57 |  | 0.89 |
| 1656 | NTM1A_HUMAN | N-terminal Xaa-Pro-Lys N-methyltransferase 1 | Q9BV86 | 1.89 | 0.82 | 1.35 |
| 1657 | NTPCR_HUMAN | Cancer-related nucleoside-triphosphatase | Q9BSD7 | 1.07 | 1.07 | 0.89 |
| 1658 | NU107_HUMAN | Nuclear pore complex protein Nup107 | P57740 | 1.34 |  | 0.78 |
| 1659 | NU155_HUMAN | Nuclear pore complex protein Nup155 | O75694 |  | 1.34 | 0.85 |
| 1660 | NU1M_HUMAN | NADH-ubiquinone oxidoreductase chain 1 | P03886 | 2.29 | 0.82 | 1.16 |
| 1661 | NU2M_HUMAN | NADH-ubiquinone oxidoreductase chain 2 | P03891 | 1.54 | 1.06 | 1.16 |
| 1662 | NU4M_HUMAN | NADH-ubiquinone oxidoreductase chain 4 | P03905 | 0.90 | 1.10 | 1.07 |
| 1663 | NU5M_HUMAN | NADH-ubiquinone oxidoreductase chain 5 | P03915 | 1.60 | 0.94 | 0.89 |
| 1664 | NU6M_HUMAN | NADH-ubiquinone oxidoreductase chain 6 | P03923 |  | 1.12 | 0.74 |
| 1665 | NUBP2_HUMAN | Cytosolic Fe-S cluster assembly factor NUBP2 | Q9Y5Y2 | 1.10 | 0.99 | 0.82 |
| 1666 | NUBPL_HUMAN | Iron-sulfur protein NUBPL | Q8TB37 | 1.09 | 1.63 | 0.94 |
| 1667 | NUCB1_HUMAN | Nucleobindin-1 | Q02818 | 1.91 | 0.77 | 1.27 |
| 1668 | NUCB2_HUMAN | Nucleobindin-2 | P80303 | 1.21 | 0.76 | 1.09 |
| 1669 | NUCKS_HUMAN | Nuclear ubiquitous casein and cyclin-dependent kinase substrate 1 | Q9H1E3 | 0.45 | 1.25 | 0.93 |
| 1670 | NUCL_HUMAN | Nucleolin | P19338 | 1.12 | 0.67 | 0.98 |
| 1671 | NUD16_HUMAN | U8 snoRNA-decapping enzyme | Q96DE0 | 1.33 |  |  |
| 1672 | NUD19_HUMAN | Nucleoside diphosphate-linked moiety X motif 19, mitochondrial | A8MXV4 | 0.82 | 0.96 | 0.80 |
| 1673 | NUDC_HUMAN | Nuclear migration protein nudC | Q9Y266 | 0.55 | 2.81 | 0.73 |
| 1674 | NUDT4_HUMAN | Diphosphoinositol polyphosphate phosphohydrolase 2 | Q9NZJ9 | 0.90 | 0.87 | 1.04 |
| 1675 | NUDT5_HUMAN | ADP-sugar pyrophosphatase | Q9UKK9 |  | 1.05 | 0.99 |
| 1676 | NUDT6_HUMAN | Nucleoside diphosphate-linked moiety X motif 6 | P53370 |  | 1.11 | 0.89 |
| 1677 | NUDT8_HUMAN | Nucleoside diphosphate-linked moiety X motif 8, mitochondrial | Q8WV74 |  | 1.18 | 0.80 |
| 1678 | NUMA1_HUMAN | Nuclear mitotic apparatus protein 1 | Q14980 | 1.00 | 0.74 | 1.04 |
| 1679 | NUP85_HUMAN | Nuclear pore complex protein Nup85 | Q9BW27 |  | 0.85 | 1.16 |
| 1680 | NUP93_HUMAN | Nuclear pore complex protein Nup93 | Q8N1F7 |  | 0.95 | 0.76 |
| 1681 | NXN_HUMAN | Nucleoredoxin | Q6DKJ4 |  |  | 0.95 |
| 1682 | OAT_HUMAN | Ornithine aminotransferase, mitochondrial | P04181 |  | 2.33 | 1.26 |
| 1683 | OBSCN_HUMAN | Obscurin | Q5VST9 | 1.31 |  | 1.09 |
| 1684 | OBSL1_HUMAN | Obscurin-like protein 1 | O75147 | 1.13 | 0.42 | 0.86 |
| 1685 | OCAD1_HUMAN | OCIA domain-containing protein 1 | Q9NX40 | 1.01 |  | 0.91 |
| 1686 | ODB2_HUMAN | Lipoamide acyltransferase component of branched-chain alpha-keto acid dehydrogenase complex, mitochondrial | P11182 | 3.08 |  | 1.14 |
| 1687 | ODBA_HUMAN | 2-oxoisovalerate dehydrogenase subunit alpha, mitochondrial | P12694 | 0.23 | 1.25 | 0.83 |
| 1688 | ODBB_HUMAN | 2-oxoisovalerate dehydrogenase subunit beta, mitochondrial | P21953 | 1.94 | 0.82 | 1.99 |
| 1689 | ODO1_HUMAN | 2-oxoglutarate dehydrogenase, mitochondrial | Q02218 | 2.19 |  | 0.97 |
| 1690 | ODO2_HUMAN | Dihydrolipoyllysine-residue succinyltransferase component of 2-oxoglutarate dehydrogenase complex, mitochondrial | P36957 | 0.91 | 0.92 | 1.34 |
| 1691 | ODP2_HUMAN | Dihydrolipoyllysine-residue acetyltransferase component of pyruvate dehydrogenase complex, mitochondrial | P10515 | 0.35 | 0.87 | 0.83 |
| 1692 | ODPA_HUMAN | Pyruvate dehydrogenase E1 component subunit alpha, somatic form, mitochondrial | P08559 |  | 1.00 | 0.95 |
| 1693 | ODPB_HUMAN | Pyruvate dehydrogenase E1 component subunit beta, mitochondrial | P11177 | 0.85 |  | 0.95 |
| 1694 | ODPX_HUMAN | Pyruvate dehydrogenase protein X component, mitochondrial | O00330 | 0.90 |  | 0.89 |
| 1695 | OFUT1_HUMAN | GDP-fucose protein O-fucosyltransferase 1 | Q9H488 |  | 0.60 | 0.98 |
| 1696 | OFUT2_HUMAN | GDP-fucose protein O-fucosyltransferase 2 | Q9Y2G5 | 1.02 |  | 0.68 |
| 1697 | OGA_HUMAN | Protein O-GlcNAcase | O60502 | 0.59 |  | 0.72 |
| 1698 | OGDHL_HUMAN | 2-oxoglutarate dehydrogenase-like, mitochondrial | Q9ULD0 | 0.59 |  | 1.08 |
| 1699 | OGT1_HUMAN | UDP-N-acetylglucosamine--peptide N-acetylglucosaminyltransferase 110 kDa subunit | O15294 | 1.10 | 0.44 | 0.96 |
| 1700 | OLA1_HUMAN | Obg-like ATPase 1 | Q9NTK5 |  | 1.04 | 0.89 |
| 1701 | OLFL1_HUMAN | Olfactomedin-like protein 1 | Q6UWY5 | 1.08 | 1.16 | 1.10 |
| 1702 | OLFL3_HUMAN | Olfactomedin-like protein 3 | Q9NRN5 |  | 1.11 | 0.71 |
| 1703 | OMA1_HUMAN | Metalloendopeptidase OMA1, mitochondrial | Q96E52 |  | 0.46 | 0.65 |
| 1704 | OPA1_HUMAN | Dynamin-like 120 kDa protein, mitochondrial | O60313 | 1.47 |  | 1.52 |
| 1705 | OPA3_HUMAN | Optic atrophy 3 protein | Q9H6K4 | 1.00 | 1.20 | 0.85 |
| 1706 | OPLA_HUMAN | 5-oxoprolinase | O14841 | 0.69 |  | 0.98 |
| 1707 | ORN_HUMAN | Oligoribonuclease, mitochondrial | Q9Y3B8 | 1.11 | 0.74 | 1.02 |
| 1708 | OSBL1_HUMAN | Oxysterol-binding protein-related protein 1 | Q9BXW6 | 1.36 | 1.89 | 0.80 |
| 1709 | OSBP1_HUMAN | Oxysterol-binding protein 1 | P22059 | 1.06 | 1.31 | 0.88 |
| 1710 | OST48_HUMAN | Dolichyl-diphosphooligosaccharide--protein glycosyltransferase 48 kDa subunit | P39656 | 1.43 | 0.72 | 1.01 |
| 1711 | OTUB1_HUMAN | Ubiquitin thioesterase OTUB1 | Q96FW1 | 1.13 | 1.09 | 0.88 |
| 1712 | OXSM_HUMAN | 3-oxoacyl-[acyl-carrier-protein] synthase, mitochondrial | Q9NWU1 | 0.33 | 1.09 | 0.92 |
| 1713 | OXSR1_HUMAN | Serine/threonine-protein kinaseR1 | O95747 | 1.54 | 0.44 | 1.04 |
| 1714 | PA1B2_HUMAN | Platelet-activating factor acetylhydrolase IB subunit beta | P68402 | 1.05 | 1.24 | 1.02 |
| 1715 | PA2G4_HUMAN | Proliferation-associated protein 2G4 | Q9UQ80 |  | 0.95 | 1.24 |
| 1716 | PABP1_HUMAN | Polyadenylate-binding protein 1 | P11940 | 1.34 | 1.01 | 1.08 |
| 1717 | PABP2_HUMAN | Polyadenylate-binding protein 2 | Q86U42 | 0.53 | 0.64 | 1.09 |
| 1718 | PABP4_HUMAN | Polyadenylate-binding protein 4 | Q13310 |  | 0.92 | 1.06 |
| 1719 | PACN2_HUMAN | Protein kinase C and casein kinase substrate in neurons protein 2 | Q9UNF0 | 1.03 | 1.18 | 1.07 |
| 1720 | PACN3_HUMAN | Protein kinase C and casein kinase substrate in neurons protein 3 | Q9UKS6 | 1.15 | 0.40 | 1.73 |
| 1721 | PACS1_HUMAN | Phosphofurin acidic cluster sorting protein 1 | Q6VY07 |  | 0.55 | 1.11 |
| 1722 | PAIP1_HUMAN | Polyadenylate-binding protein-interacting protein 1 | Q9H074 |  | 2.81 | 1.14 |
| 1723 | PAIRB_HUMAN | Plasminogen activator inhibitor 1 RNA-binding protein | Q8NC51 | 0.72 | 0.79 | 1.04 |
| 1724 | PAK2_HUMAN | Serine/threonine-protein kinase PAK 2 | Q13177 | 1.72 | 1.10 | 0.99 |
| 1725 | PAK4_HUMAN | Serine/threonine-protein kinase PAK 4 | O96013 | 0.15 | 0.64 | 0.59 |
| 1726 | PALLD_HUMAN | Palladin | Q8WX93 | 0.68 |  | 1.12 |
| 1727 | PALM2_HUMAN | Paralemmin-2 | Q8IXS6 | 0.94 | 1.22 | 1.15 |
| 1728 | PALMD_HUMAN | Palmdelphin | Q9NP74 | 2.19 | 0.89 | 0.81 |
| 1729 | PANK4_HUMAN | Pantothenate kinase 4 | Q9NVE7 | 1.53 | 0.90 | 1.13 |
| 1730 | PARK7_HUMAN | Protein DJ-1 | Q99497 | 1.19 | 1.19 | 1.20 |
| 1731 | PARP1_HUMAN | Poly [ADP-ribose] polymerase 1 | P09874 | 1.10 | 1.11 | 0.95 |
| 1732 | PARVA_HUMAN | Alpha-parvin | Q9NVD7 | 0.79 | 0.12 | 1.10 |
| 1733 | PARVB_HUMAN | Beta-parvin | Q9HBI1 | 0.90 | 0.36 | 0.86 |
| 1734 | PAWR_HUMAN | PRKC apoptosis WT1 regulator protein | Q96IZ0 |  | 2.11 | 0.86 |
| 1735 | PBDC1_HUMAN | Protein PBDC1 | Q9BVG4 | 1.33 |  | 1.19 |
| 1736 | PBIP1_HUMAN | Pre-B-cell leukemia transcription factor-interacting protein 1 | Q96AQ6 | 1.20 | 1.19 | 1.12 |
| 1737 | PCBP1_HUMAN | Poly(rC)-binding protein 1 | Q15365 | 1.85 |  | 1.22 |
| 1738 | PCBP2_HUMAN | Poly(rC)-binding protein 2 | Q15366 | 1.16 | 1.06 | 0.76 |
| 1739 | PCCA_HUMAN | Propionyl-CoA carboxylase alpha chain, mitochondrial | P05165 | 1.27 | 1.28 | 0.96 |
| 1740 | PCCB_HUMAN | Propionyl-CoA carboxylase beta chain, mitochondrial | P05166 | 0.96 |  | 1.06 |
| 1741 | PCNP_HUMAN | PEST proteolytic signal-containing nuclear protein | Q8WW12 |  | 1.57 | 1.29 |
| 1742 | PCOC1_HUMAN | Procollagen C-endopeptidase enhancer 1 | Q15113 |  | 1.18 | 1.24 |
| 1743 | PCTL_HUMAN | PCTP-like protein | Q9Y365 | 0.85 |  |  |
| 1744 | PCY1A_HUMAN | Choline-phosphate cytidylyltransferase A | P49585 | 0.22 | 0.72 | 0.81 |
| 1745 | PCY2_HUMAN | Ethanolamine-phosphate cytidylyltransferase | Q99447 | 0.87 | 0.89 | 0.95 |
| 1746 | PCYOX_HUMAN | Prenylcysteine oxidase 1 | Q9UHG3 |  | 1.49 | 1.07 |
| 1747 | PDC10_HUMAN | Programmed cell death protein 10 | Q9BUL8 | 1.05 | 0.37 | 1.19 |
| 1748 | PDC6I_HUMAN | Programmed cell death 6-interacting protein | Q8WUM4 | 0.55 | 1.22 | 1.90 |
| 1749 | PDCD5_HUMAN | Programmed cell death protein 5 | O14737 | 0.78 | 0.95 | 0.83 |
| 1750 | PDCD6_HUMAN | Programmed cell death protein 6 | O75340 | 0.69 | 1.45 | 0.72 |
| 1751 | PDE12_HUMAN | 2',5'-phosphodiesterase 12 | Q6L8Q7 | 1.24 | 0.90 | 0.93 |
| 1752 | PDE1C_HUMAN | Calcium/calmodulin-dependent 3',5'-cyclic nucleotide phosphodiesterase 1C | Q14123 | 1.64 |  | 0.94 |
| 1753 | PDE3A_HUMAN | cGMP-inhibited 3',5'-cyclic phosphodiesterase A | Q14432 | 0.82 | 1.10 | 0.78 |
| 1754 | PDE6D_HUMAN | Retinal rod rhodopsin-sensitive cGMP 3',5'-cyclic phosphodiesterase subunit delta | O43924 |  | 1.39 |  |
| 1755 | PDIA1_HUMAN | Protein disulfide-isomerase | P07237 | 0.73 |  | 1.13 |
| 1756 | PDIA3_HUMAN | Protein disulfide-isomerase A3 | P30101 | 0.94 | 1.06 | 0.88 |
| 1757 | PDIA4_HUMAN | Protein disulfide-isomerase A4 | P13667 | 1.24 | 1.21 | 1.17 |
| 1758 | PDIA6_HUMAN | Protein disulfide-isomerase A6 | Q15084 |  | 0.74 | 0.99 |
| 1759 | PDIP2_HUMAN | Polymerase delta-interacting protein 2 | Q9Y2S7 | 2.63 | 0.70 | 0.83 |
| 1760 | PDK1_HUMAN | [Pyruvate dehydrogenase (acetyl-transferring)] kinase isozyme 1, mitochondrial | Q15118 | 0.31 | 1.22 | 0.83 |
| 1761 | PDK2_HUMAN | [Pyruvate dehydrogenase (acetyl-transferring)] kinase isozyme 2, mitochondrial | Q15119 | 0.79 | 1.01 | 0.99 |
| 1762 | PDK3_HUMAN | [Pyruvate dehydrogenase (acetyl-transferring)] kinase isozyme 3, mitochondrial | Q15120 | 0.74 | 1.06 | 0.87 |
| 1763 | PDLI1_HUMAN | PDZ and LIM domain protein 1 | O00151 | 0.04 | 0.64 | 1.06 |
| 1764 | PDLI3_HUMAN | PDZ and LIM domain protein 3 | Q53GG5 | 3.47 |  | 1.04 |
| 1765 | PDLI4_HUMAN | PDZ and LIM domain protein 4 | P50479 | 0.48 | 1.38 | 0.86 |
| 1766 | PDLI5_HUMAN | PDZ and LIM domain protein 5 | Q96HC4 | 0.94 | 0.61 | 1.05 |
| 1767 | PDLI7_HUMAN | PDZ and LIM domain protein 7 | Q9NR12 |  | 1.25 | 0.96 |
| 1768 | PDP1_HUMAN | [Pyruvate dehydrogenase [acetyl-transferring]]-phosphatase 1, mitochondrial | Q9P0J1 | 0.85 | 1.13 | 0.98 |
| 1769 | PDPR_HUMAN | Pyruvate dehydrogenase phosphatase regulatory subunit, mitochondrial | Q8NCN5 | 0.50 | 0.82 | 1.27 |
| 1770 | PDXK_HUMAN | Pyridoxal kinase | O00764 | 1.05 | 0.85 | 0.95 |
| 1771 | PDZ11_HUMAN | PDZ domain-containing protein 11 | Q5EBL8 | 0.74 | 1.15 | 1.91 |
| 1772 | PEA15_HUMAN | Astrocytic phosphoprotein PEA-15 | Q15121 | 0.36 | 0.91 | 1.08 |
| 1773 | PEBP1_HUMAN | Phosphatidylethanolamine-binding protein 1 | P30086 |  | 1.54 | 1.07 |
| 1774 | PECA1_HUMAN | Platelet endothelial cell adhesion molecule | P16284 |  |  | 1.34 |
| 1775 | PEDF_HUMAN | Pigment epithelium-derived factor | P36955 | 0.63 |  | 0.94 |
| 1776 | PEF1_HUMAN | Peflin | Q9UBV8 | 0.79 | 0.95 | 0.90 |
| 1777 | PEPD_HUMAN | Xaa-Pro dipeptidase | P12955 | 0.95 | 0.64 | 0.97 |
| 1778 | PEPL_HUMAN | Periplakin | O60437 | 2.65 | 1.96 | 0.52 |
| 1779 | PEPL1_HUMAN | Probable aminopeptidase NPEPL1 | Q8NDH3 | 1.24 |  | 0.99 |
| 1780 | PERI_HUMAN | Peripherin | P41219 | 0.43 | 2.01 | 1.20 |
| 1781 | PERM1_HUMAN | PGC-1 and ERR-induced regulator in muscle protein 1 | Q5SV97 | 0.82 | 0.42 | 1.04 |
| 1782 | PEX19_HUMAN | Peroxisomal biogenesis factor 19 | P40855 | 1.89 |  | 0.91 |
| 1783 | PFD1_HUMAN | Prefoldin subunit 1 | O60925 | 0.91 | 0.82 | 1.17 |
| 1784 | PFD2_HUMAN | Prefoldin subunit 2 | Q9UHV9 | 0.19 | 0.91 | 0.94 |
| 1785 | PFD3_HUMAN | Prefoldin subunit 3 | P61758 | 0.60 | 0.90 | 0.84 |
| 1786 | PFD4_HUMAN | Prefoldin subunit 4 | Q9NQP4 | 1.71 | 0.79 | 0.54 |
| 1787 | PFD5_HUMAN | Prefoldin subunit 5 | Q99471 | 0.92 | 0.65 | 0.53 |
| 1788 | PFD6_HUMAN | Prefoldin subunit 6 | O15212 | 0.83 | 0.92 | 0.73 |
| 1789 | PFKAL_HUMAN | ATP-dependent 6-phosphofructokinase, liver type | P17858 | 1.17 | 0.99 | 1.01 |
| 1790 | PFKAM_HUMAN | ATP-dependent 6-phosphofructokinase, muscle type | P08237 |  |  | 1.05 |
| 1791 | PFKAP_HUMAN | ATP-dependent 6-phosphofructokinase, platelet type | Q01813 | 0.52 | 1.11 | 0.89 |
| 1792 | PGAM1_HUMAN | Phosphoglycerate mutase 1 | P18669 | 0.43 |  | 1.16 |
| 1793 | PGAM2_HUMAN | Phosphoglycerate mutase 2 | P15259 | 0.92 | 1.09 | 1.04 |
| 1794 | PGAM5_HUMAN | Serine/threonine-protein phosphatase PGAM5, mitochondrial | Q96HS1 | 0.80 |  | 0.93 |
| 1795 | PGBM_HUMAN | Basement membrane-specific heparan sulfate proteoglycan core protein | P98160 | 0.79 | 1.26 | 1.09 |
| 1796 | PGES2_HUMAN | Prostaglandin E synthase 2 | Q9H7Z7 | 0.91 |  | 1.04 |
| 1797 | PGK1_HUMAN | Phosphoglycerate kinase 1 | P00558 | 1.53 |  | 1.80 |
| 1798 | PGM1_HUMAN | Phosphoglucomutase-1 | P36871 | 0.90 | 0.96 | 1.09 |
| 1799 | PGM2_HUMAN | Phosphoglucomutase-2 | Q96G03 |  | 0.49 | 1.12 |
| 1800 | PGM5_HUMAN | Phosphoglucomutase-like protein 5 | Q15124 | 1.37 |  | 0.98 |
| 1801 | PGP_HUMAN | Phosphoglycolate phosphatase | A6NDG6 | 0.88 | 1.28 | 0.79 |
| 1802 | PGRC1_HUMAN | Membrane-associated progesterone receptor component 1 | O00264 | 0.96 | 1.39 | 0.75 |
| 1803 | PGRC2_HUMAN | Membrane-associated progesterone receptor component 2 | O15173 |  | 0.93 | 1.25 |
| 1804 | PGRP2_HUMAN | N-acetylmuramoyl-L-alanine amidase | Q96PD5 | 0.67 | 0.75 | 0.67 |
| 1805 | PGS1_HUMAN | Biglycan | P21810 |  | 0.97 | 1.04 |
| 1806 | PGS2_HUMAN | Decorin | P07585 | 1.10 | 0.78 | 1.02 |
| 1807 | PGTA_HUMAN | Geranylgeranyl transferase type-2 subunit alpha | Q92696 | 0.87 |  | 0.95 |
| 1808 | PHB_HUMAN | Prohibitin | P35232 | 0.86 | 1.01 | 0.89 |
| 1809 | PHB2_HUMAN | Prohibitin-2 | Q99623 | 1.16 | 0.30 | 0.98 |
| 1810 | PHKG1_HUMAN | Phosphorylase b kinase gamma catalytic chain, skeletal muscle/heart isoform | Q16816 | 1.21 | 1.21 | 0.35 |
| 1811 | PHLB1_HUMAN | Pleckstrin homology-like domain family B member 1 | Q86UU1 |  | 0.42 | 1.49 |
| 1812 | PHOCN_HUMAN | MOB-like protein phocein | Q9Y3A3 | 1.06 | 0.95 | 1.34 |
| 1813 | PHP14_HUMAN | 14 kDa phosphohistidine phosphatase | Q9NRX4 | 0.92 | 0.89 | 0.79 |
| 1814 | PHS_HUMAN | Pterin-4-alpha-carbinolamine dehydratase | P61457 | 1.27 | 1.84 | 0.80 |
| 1815 | PHS2_HUMAN | Pterin-4-alpha-carbinolamine dehydratase 2 | Q9H0N5 | 1.09 |  | 1.00 |
| 1816 | PI16_HUMAN | Peptidase inhibitor 16 | Q6UXB8 |  | 1.56 | 1.00 |
| 1817 | PICAL_HUMAN | Phosphatidylinositol-binding clathrin assembly protein | Q13492 |  | 0.86 | 0.47 |
| 1818 | PICK1_HUMAN | PRKCA-binding protein | Q9NRD5 | 0.70 |  |  |
| 1819 | PIGS_HUMAN | GPI transamidase component PIG-S | Q96S52 |  | 0.44 | 1.26 |
| 1820 | PIGT_HUMAN | GPI transamidase component PIG-T | Q969N2 | 0.77 |  | 0.77 |
| 1821 | PIMT_HUMAN | Protein-L-isoaspartate(D-aspartate) O-methyltransferase | P22061 | 0.68 | 1.09 | 1.21 |
| 1822 | PIN1_HUMAN | Peptidyl-prolyl cis-trans isomerase NIMA-interacting 1 | Q13526 | 0.78 | 1.29 | 0.90 |
| 1823 | PIPNA_HUMAN | Phosphatidylinositol transfer protein alpha isoform | Q00169 | 1.06 | 0.77 | 1.17 |
| 1824 | PIPNB_HUMAN | Phosphatidylinositol transfer protein beta isoform | P48739 | 1.33 | 0.42 | 1.37 |
| 1825 | PITH1_HUMAN | PITH domain-containing protein 1 | Q9GZP4 |  | 1.27 | 1.21 |
| 1826 | PKHA5_HUMAN | Pleckstrin homology domain-containing family A member 5 | Q9HAU0 | 1.72 |  | 1.04 |
| 1827 | PKHA6_HUMAN | Pleckstrin homology domain-containing family A member 6 | Q9Y2H5 |  | 0.26 | 1.04 |
| 1828 | PKP2_HUMAN | Plakophilin-2 | Q99959 | 1.32 |  | 0.92 |
| 1829 | PLAK_HUMAN | Junction plakoglobin | P14923 | 1.17 |  | 0.94 |
| 1830 | PLAP_HUMAN | Phospholipase A-2-activating protein | Q9Y263 |  | 1.05 | 1.07 |
| 1831 | PLCA_HUMAN | 1-acyl-sn-glycerol-3-phosphate acyltransferase alpha | Q99943 |  | 1.89 | 1.10 |
| 1832 | PLCD3_HUMAN | 1-phosphatidylinositol 4,5-bisphosphate phosphodiesterase delta-3 | Q8N3E9 | 0.29 | 0.54 | 1.40 |
| 1833 | PLCL1_HUMAN | Inactive phospholipase C-like protein 1 | Q15111 | 1.04 | 0.79 | 1.42 |
| 1834 | PLCX3_HUMAN | PI-PLC X domain-containing protein 3 | Q63HM9 | 2.68 | 1.15 | 1.11 |
| 1835 | PLEC_HUMAN | Plectin | Q15149 |  | 0.69 | 1.96 |
| 1836 | PLIN1_HUMAN | Perilipin-1 | O60240 | 3.56 | 1.37 | 1.22 |
| 1837 | PLIN3_HUMAN | Perilipin-3 | O60664 | 0.93 |  | 0.73 |
| 1838 | PLIN4_HUMAN | Perilipin-4 | Q96Q06 | 1.26 |  | 1.25 |
| 1839 | PLM_HUMAN | Phospholemman | O00168 | 1.11 | 1.47 | 0.91 |
| 1840 | PLMN_HUMAN | Plasminogen | P00747 | 1.06 | 0.99 | 1.02 |
| 1841 | PLP2_HUMAN | Proteolipid protein 2 | Q04941 | 1.92 | 0.95 | 1.45 |
| 1842 | PLPP_HUMAN | Pyridoxal phosphate phosphatase | Q96GD0 | 0.97 | 1.06 | 1.02 |
| 1843 | PLRKT_HUMAN | Plasminogen receptor (KT) | Q9HBL7 | 0.56 | 0.98 |  |
| 1844 | PLSL_HUMAN | Plastin-2 | P13796 | 1.28 | 1.19 | 1.78 |
| 1845 | PLST_HUMAN | Plastin-3 | P13797 | 1.28 | 1.33 | 1.29 |
| 1846 | PLTP_HUMAN | Phospholipid transfer protein | P55058 |  | 2.83 | 1.38 |
| 1847 | PLVAP_HUMAN | Plasmalemma vesicle-associated protein | Q9BX97 |  |  | 0.74 |
| 1848 | PNCB_HUMAN | Nicotinate phosphoribosyltransferase | Q6XQN6 | 2.51 | 0.88 | 0.80 |
| 1849 | PNPH_HUMAN | Purine nucleoside phosphorylase | P00491 | 0.16 | 1.37 | 1.15 |
| 1850 | PNPO_HUMAN | Pyridoxine-5'-phosphate oxidase | Q9NVS9 |  | 1.64 | 0.99 |
| 1851 | PNPT1_HUMAN | Polyribonucleotide nucleotidyltransferase 1, mitochondrial | Q8TCS8 | 0.99 | 0.46 | 0.91 |
| 1852 | PODN_HUMAN | Podocan | Q7Z5L7 |  | 0.86 | 1.02 |
| 1853 | PODXL_HUMAN | Podocalyxin | O00592 | 1.15 | 0.80 | 0.95 |
| 1854 | PON1_HUMAN | Serum paraoxonase/arylesterase 1 | P27169 |  | 0.64 | 0.92 |
| 1855 | PON2_HUMAN | Serum paraoxonase/arylesterase 2 | Q15165 | 3.25 | 0.93 | 1.10 |
| 1856 | POPD1_HUMAN | Blood vessel epicardial substance | Q8NE79 |  | 1.27 | 1.17 |
| 1857 | POPD2_HUMAN | Popeye domain-containing protein 2 | Q9HBU9 |  | 1.13 |  |
| 1858 | POSTN_HUMAN | Periostin | Q15063 | 1.26 |  | 1.04 |
| 1859 | POTEI_HUMAN | POTE ankyrin domain family member I | P0CG38 |  | 0.82 | 0.82 |
| 1860 | POTEJ_HUMAN | POTE ankyrin domain family member J | P0CG39 | 1.14 |  |  |
| 1861 | PP12C_HUMAN | Protein phosphatase 1 regulatory subunit 12C | Q9BZL4 | 1.11 | 0.45 | 0.93 |
| 1862 | PP13G_HUMAN | Protein phosphatase 1 regulatory subunit 3G | B7ZBB8 | 0.49 |  | 0.74 |
| 1863 | PP14B_HUMAN | Protein phosphatase 1 regulatory subunit 14B | Q96C90 | 1.39 |  | 1.32 |
| 1864 | PP14C_HUMAN | Protein phosphatase 1 regulatory subunit 14C | Q8TAE6 | 0.54 | 1.28 | 0.84 |
| 1865 | PP1A_HUMAN | Serine/threonine-protein phosphatase PP1-alpha catalytic subunit | P62136 |  | 0.77 | 0.89 |
| 1866 | PP1B_HUMAN | Serine/threonine-protein phosphatase PP1-beta catalytic subunit | P62140 | 0.76 | 1.53 | 1.47 |
| 1867 | PP1R7_HUMAN | Protein phosphatase 1 regulatory subunit 7 | Q15435 | 0.97 | 1.18 | 1.17 |
| 1868 | PP2AA_HUMAN | Serine/threonine-protein phosphatase 2A catalytic subunit alpha isoform | P67775 | 1.13 | 1.08 | 0.63 |
| 1869 | PP2BB_HUMAN | Serine/threonine-protein phosphatase 2B catalytic subunit beta isoform | P16298 | 0.44 |  | 0.44 |
| 1870 | PPAC_HUMAN | Low molecular weight phosphotyrosine protein phosphatase | P24666 |  | 1.37 | 0.79 |
| 1871 | PPAL_HUMAN | Lysosomal acid phosphatase | P11117 | 1.17 |  | 0.74 |
| 1872 | PPCE_HUMAN | Prolyl endopeptidase | P48147 |  | 0.71 | 1.05 |
| 1873 | PPGB_HUMAN | Lysosomal protective protein | P10619 |  | 1.20 | 0.89 |
| 1874 | PPIA_HUMAN | Peptidyl-prolyl cis-trans isomerase A | P62937 | 0.69 |  | 0.86 |
| 1875 | PPIB_HUMAN | Peptidyl-prolyl cis-trans isomerase B | P23284 | 1.50 |  | 0.89 |
| 1876 | PPIC_HUMAN | Peptidyl-prolyl cis-trans isomerase C | P45877 |  | 0.95 |  |
| 1877 | PPIF_HUMAN | Peptidyl-prolyl cis-trans isomerase F, mitochondrial | P30405 | 0.53 | 1.34 | 0.96 |
| 1878 | PPIH_HUMAN | Peptidyl-prolyl cis-trans isomerase H | O43447 | 1.85 |  | 0.82 |
| 1879 | PPIL1_HUMAN | Peptidyl-prolyl cis-trans isomerase-like 1 | Q9Y3C6 | 4.70 |  | 1.04 |
| 1880 | PPIL3_HUMAN | Peptidyl-prolyl cis-trans isomerase-like 3 | Q9H2H8 | 1.42 | 1.11 | 0.68 |
| 1881 | PPLA_HUMAN | Cardiac phospholamban | P26678 | 0.63 | 3.70 | 0.69 |
| 1882 | PPM1A_HUMAN | Protein phosphatase 1A | P35813 |  |  | 0.27 |
| 1883 | PPM1F_HUMAN | Protein phosphatase 1F | P49593 |  |  | 0.85 |
| 1884 | PPM1K_HUMAN | Protein phosphatase 1K, mitochondrial | Q8N3J5 | 0.78 | 0.49 | 1.66 |
| 1885 | PPME1_HUMAN | Protein phosphatase methylesterase 1 | Q9Y570 | 1.07 | 2.42 | 1.10 |
| 1886 | PPN_HUMAN | Papilin | O95428 |  | 0.97 | 1.32 |
| 1887 | PPOX_HUMAN | Protoporphyrinogen oxidase | P50336 | 1.84 | 3.25 | 1.06 |
| 1888 | PPP5_HUMAN | Serine/threonine-protein phosphatase 5 | P53041 |  | 0.88 | 1.27 |
| 1889 | PPR3A_HUMAN | Protein phosphatase 1 regulatory subunit 3A | Q16821 | 1.37 | 1.02 | 0.99 |
| 1890 | PPT1_HUMAN | Palmitoyl-protein thioesterase 1 | P50897 | 0.44 | 1.17 | 1.04 |
| 1891 | PPTC7_HUMAN | Protein phosphatase PTC7 homolog | Q8NI37 | 0.86 | 0.27 | 1.17 |
| 1892 | PRAF3_HUMAN | PRA1 family protein 3 | O75915 | 1.28 |  | 1.11 |
| 1893 | PRDBP_HUMAN | Protein kinase C delta-binding protein | Q969G5 | 0.84 | 1.67 | 0.89 |
| 1894 | PRDX1_HUMAN | Peroxiredoxin-1 | Q06830 | 0.95 | 1.17 | 0.95 |
| 1895 | PRDX2_HUMAN | Peroxiredoxin-2 | P32119 |  | 1.43 | 1.09 |
| 1896 | PRDX3_HUMAN | Thioredoxin-dependent peroxide reductase, mitochondrial | P30048 |  | 1.87 | 0.95 |
| 1897 | PRDX4_HUMAN | Peroxiredoxin-4 | Q13162 | 2.38 | 0.27 | 0.88 |
| 1898 | PRDX5_HUMAN | Peroxiredoxin-5, mitochondrial | P30044 | 0.66 | 1.16 | 1.28 |
| 1899 | PRDX6_HUMAN | Peroxiredoxin-6 | P30041 | 1.69 |  | 1.01 |
| 1900 | PRELP_HUMAN | Prolargin | P51888 | 0.96 |  | 1.09 |
| 1901 | PREP_HUMAN | Presequence protease, mitochondrial | Q5JRX3 | 0.86 |  | 1.11 |
| 1902 | PRKDC_HUMAN | DNA-dependent protein kinase catalytic subunit | P78527 | 1.41 | 1.18 | 1.01 |
| 1903 | PRKRA_HUMAN | Interferon-inducible double-stranded RNA-dependent protein kinase activator A | O75569 | 1.31 | 0.86 | 1.03 |
| 1904 | PROB1_HUMAN | Proline-rich basic protein 1 | E7EW31 | 0.91 | 1.03 | 0.75 |
| 1905 | PROF1_HUMAN | Profilin-1 | P07737 | 1.46 |  | 1.05 |
| 1906 | PROF2_HUMAN | Profilin-2 | P35080 | 1.04 |  | 0.80 |
| 1907 | PROS_HUMAN | Vitamin K-dependent protein S | P07225 | 0.86 |  | 0.77 |
| 1908 | PROSC_HUMAN | Proline synthase co-transcribed bacterial homolog protein | O94903 | 1.53 | 2.29 | 1.02 |
| 1909 | PRP19_HUMAN | Pre-mRNA-processing factor 19 | Q9UMS4 | 0.83 |  | 1.11 |
| 1910 | PRP8_HUMAN | Pre-mRNA-processing-splicing factor 8 | Q6P2Q9 | 3.34 | 2.23 | 1.05 |
| 1911 | PRPS1_HUMAN | Ribose-phosphate pyrophosphokinase 1 | P60891 |  | 2.19 | 0.66 |
| 1912 | PRRC1_HUMAN | Protein PRRC1 | Q96M27 |  | 1.00 | 1.41 |
| 1913 | PRS10_HUMAN | 26S protease regulatory subunit 10B | P62333 | 0.95 | 0.47 | 1.02 |
| 1914 | PRS4_HUMAN | 26S protease regulatory subunit 4 | P62191 | 1.63 | 0.95 | 1.24 |
| 1915 | PRS6A_HUMAN | 26S protease regulatory subunit 6A | P17980 | 1.28 | 0.84 | 1.26 |
| 1916 | PRS6B_HUMAN | 26S protease regulatory subunit 6B | P43686 | 0.79 |  | 1.03 |
| 1917 | PRS7_HUMAN | 26S protease regulatory subunit 7 | P35998 | 1.05 | 1.74 | 1.03 |
| 1918 | PRS8_HUMAN | 26S protease regulatory subunit 8 | P62195 | 0.86 |  | 0.95 |
| 1919 | PRUNE_HUMAN | Protein prune homolog | Q86TP1 | 0.74 | 1.25 | 0.92 |
| 1920 | PSA_HUMAN | Puromycin-sensitive aminopeptidase | P55786 | 0.18 |  | 1.04 |
| 1921 | PSA1_HUMAN | Proteasome subunit alpha type-1 | P25786 | 1.38 | 1.27 | 0.93 |
| 1922 | PSA2_HUMAN | Proteasome subunit alpha type-2 | P25787 |  | 0.64 | 0.96 |
| 1923 | PSA3_HUMAN | Proteasome subunit alpha type-3 | P25788 | 1.31 | 0.29 | 1.00 |
| 1924 | PSA4_HUMAN | Proteasome subunit alpha type-4 | P25789 | 0.79 | 1.10 | 0.90 |
| 1925 | PSA5_HUMAN | Proteasome subunit alpha type-5 | P28066 | 0.04 | 0.64 | 0.88 |
| 1926 | PSA6_HUMAN | Proteasome subunit alpha type-6 | P60900 | 1.75 | 0.43 | 1.18 |
| 1927 | PSA7_HUMAN | Proteasome subunit alpha type-7 | O14818 | 0.96 | 0.69 | 1.10 |
| 1928 | PSB1_HUMAN | Proteasome subunit beta type-1 | P20618 | 1.75 |  | 1.08 |
| 1929 | PSB2_HUMAN | Proteasome subunit beta type-2 | P49721 | 0.58 | 0.95 | 0.81 |
| 1930 | PSB3_HUMAN | Proteasome subunit beta type-3 | P49720 | 0.68 | 0.75 | 1.05 |
| 1931 | PSB4_HUMAN | Proteasome subunit beta type-4 | P28070 | 1.16 | 0.90 | 0.88 |
| 1932 | PSB5_HUMAN | Proteasome subunit beta type-5 | P28074 | 0.94 | 1.51 | 1.10 |
| 1933 | PSB6_HUMAN | Proteasome subunit beta type-6 | P28072 | 0.98 | 0.98 | 0.62 |
| 1934 | PSB7_HUMAN | Proteasome subunit beta type-7 | Q99436 | 1.22 | 2.65 | 0.95 |
| 1935 | PSD11_HUMAN | 26S proteasome non-ATPase regulatory subunit 11 | O00231 | 0.86 | 2.17 | 0.91 |
| 1936 | PSD12_HUMAN | 26S proteasome non-ATPase regulatory subunit 12 | O00232 | 4.06 | 1.34 | 0.59 |
| 1937 | PSD13_HUMAN | 26S proteasome non-ATPase regulatory subunit 13 | Q9UNM6 | 0.72 | 0.72 | 0.91 |
| 1938 | PSDE_HUMAN | 26S proteasome non-ATPase regulatory subunit 14 | O00487 | 0.69 | 0.80 | 1.05 |
| 1939 | PSMD1_HUMAN | 26S proteasome non-ATPase regulatory subunit 1 | Q99460 | 1.61 | 0.67 | 0.85 |
| 1940 | PSMD2_HUMAN | 26S proteasome non-ATPase regulatory subunit 2 | Q13200 | 1.41 | 0.83 | 1.12 |
| 1941 | PSMD3_HUMAN | 26S proteasome non-ATPase regulatory subunit 3 | O43242 | 0.63 | 1.03 | 1.46 |
| 1942 | PSMD4_HUMAN | 26S proteasome non-ATPase regulatory subunit 4 | P55036 |  |  | 0.97 |
| 1943 | PSMD5_HUMAN | 26S proteasome non-ATPase regulatory subunit 5 | Q16401 | 1.58 | 1.05 | 1.34 |
| 1944 | PSMD6_HUMAN | 26S proteasome non-ATPase regulatory subunit 6 | Q15008 | 1.28 | 2.21 | 0.88 |
| 1945 | PSMD7_HUMAN | 26S proteasome non-ATPase regulatory subunit 7 | P51665 | 1.07 | 1.45 | 0.58 |
| 1946 | PSMD8_HUMAN | 26S proteasome non-ATPase regulatory subunit 8 | P48556 | 0.96 | 0.74 | 1.00 |
| 1947 | PSMD9_HUMAN | 26S proteasome non-ATPase regulatory subunit 9 | O00233 |  | 0.92 | 1.15 |
| 1948 | PSME1_HUMAN | Proteasome activator complex subunit 1 | Q06323 | 0.54 | 0.97 | 0.90 |
| 1949 | PSME2_HUMAN | Proteasome activator complex subunit 2 | Q9UL46 | 1.07 | 0.76 | 0.82 |
| 1950 | PSMF1_HUMAN | Proteasome inhibitor PI31 subunit | Q92530 |  | 1.20 | 1.29 |
| 1951 | PSPC1_HUMAN | Paraspeckle component 1 | Q8WXF1 | 1.19 |  |  |
| 1952 | PT100_HUMAN | Protein PET100 homolog, mitochondrial | P0DJ07 | 0.59 |  |  |
| 1953 | PTBP1_HUMAN | Polypyrimidine tract-binding protein 1 | P26599 | 1.22 | 1.04 | 1.17 |
| 1954 | PTCD3_HUMAN | Pentatricopeptide repeat domain-containing protein 3, mitochondrial | Q96EY7 |  | 1.17 | 1.05 |
| 1955 | PTER_HUMAN | Phosphotriesterase-related protein | Q96BW5 | 1.09 | 1.00 | 1.12 |
| 1956 | PTGDS_HUMAN | Prostaglandin-H2 D-isomerase | P41222 | 1.10 | 1.05 | 1.14 |
| 1957 | PTGIS_HUMAN | Prostacyclin synthase | Q16647 | 1.24 | 0.78 | 0.84 |
| 1958 | PTGR1_HUMAN | Prostaglandin reductase 1 | Q14914 | 0.50 | 1.03 | 0.83 |
| 1959 | PTGR2_HUMAN | Prostaglandin reductase 2 | Q8N8N7 | 1.13 | 1.16 | 0.88 |
| 1960 | PTH2_HUMAN | Peptidyl-tRNA hydrolase 2, mitochondrial | Q9Y3E5 | 0.46 | 3.56 | 1.27 |
| 1961 | PTMA_HUMAN | Prothymosin alpha | P06454 | 1.13 |  | 1.13 |
| 1962 | PTMS_HUMAN | Parathymosin | P20962 | 1.21 | 1.02 | 0.85 |
| 1963 | PTN1_HUMAN | Tyrosine-protein phosphatase non-receptor type 1 | P18031 | 0.43 | 0.67 | 0.96 |
| 1964 | PTN11_HUMAN | Tyrosine-protein phosphatase non-receptor type 11 | Q06124 | 1.06 | 0.87 | 1.00 |
| 1965 | PTN23_HUMAN | Tyrosine-protein phosphatase non-receptor type 23 | Q9H3S7 |  | 0.78 | 0.72 |
| 1966 | PTPA_HUMAN | Serine/threonine-protein phosphatase 2A activator | Q15257 | 1.20 | 1.15 | 0.83 |
| 1967 | PTRD1_HUMAN | Putative peptidyl-tRNA hydrolase PTRHD1 | Q6GMV3 | 0.26 | 0.53 | 0.68 |
| 1968 | PTRF_HUMAN | Polymerase I and transcript release factor | Q6NZI2 | 1.37 |  | 1.14 |
| 1969 | PUR2_HUMAN | Trifunctional purine biosynthetic protein adenosine-3 | P22102 | 0.82 | 1.09 | 1.19 |
| 1970 | PUR6_HUMAN | Multifunctional protein ADE2 | P22234 | 1.26 |  | 0.97 |
| 1971 | PUR8_HUMAN | Adenylosuccinate lyase | P30566 |  |  | 0.91 |
| 1972 | PUR9_HUMAN | Bifunctional purine biosynthesis protein PURH | P31939 | 1.27 | 1.20 | 0.74 |
| 1973 | PURA_HUMAN | Transcriptional activator protein Pur-alpha | Q00577 | 0.95 | 0.99 | 0.86 |
| 1974 | PURA1_HUMAN | Adenylosuccinate synthetase isozyme 1 | Q8N142 | 0.97 | 0.78 | 0.97 |
| 1975 | PURB_HUMAN | Transcriptional activator protein Pur-beta | Q96QR8 | 0.94 |  |  |
| 1976 | PXDC2_HUMAN | Plexin domain-containing protein 2 | Q6UX71 | 0.59 | 0.86 |  |
| 1977 | PXDN_HUMAN | Peroxidasin homolog | Q92626 |  | 0.91 | 0.63 |
| 1978 | PYC_HUMAN | Pyruvate carboxylase, mitochondrial | P11498 | 0.70 |  |  |
| 1979 | PYGB_HUMAN | Glycogen phosphorylase, brain form | P11216 | 1.20 |  | 0.97 |
| 1980 | PYGL_HUMAN | Glycogen phosphorylase, liver form | P06737 | 1.29 |  | 0.89 |
| 1981 | PYGM_HUMAN | Glycogen phosphorylase, muscle form | P11217 |  |  | 1.06 |
| 1982 | PYRD_HUMAN | Dihydroorotate dehydrogenase (quinone), mitochondrial | Q02127 |  | 0.80 | 0.84 |
| 1983 | QCR1_HUMAN | Cytochrome b-c1 complex subunit 1, mitochondrial | P31930 | 1.09 |  | 0.68 |
| 1984 | QCR10_HUMAN | Cytochrome b-c1 complex subunit 10 | O14957 | 0.91 | 0.89 | 0.92 |
| 1985 | QCR2_HUMAN | Cytochrome b-c1 complex subunit 2, mitochondrial | P22695 |  |  | 1.06 |
| 1986 | QCR6_HUMAN | Cytochrome b-c1 complex subunit 6, mitochondrial | P07919 | 1.17 | 0.92 | 0.92 |
| 1987 | QCR7_HUMAN | Cytochrome b-c1 complex subunit 7 | P14927 | 1.26 | 1.02 | 0.95 |
| 1988 | QCR8_HUMAN | Cytochrome b-c1 complex subunit 8 | O14949 | 1.41 |  | 1.16 |
| 1989 | QCR9_HUMAN | Cytochrome b-c1 complex subunit 9 | Q9UDW1 | 0.74 | 2.11 | 1.16 |
| 1990 | QKI_HUMAN | Protein quaking | Q96PU8 | 0.89 | 1.10 | 1.04 |
| 1991 | QOR_HUMAN | Quinone oxidoreductase | Q08257 | 0.74 | 0.97 | 0.82 |
| 1992 | RAB10_HUMAN | Ras-related protein Rab-10 | P61026 |  | 0.89 | 0.65 |
| 1993 | RAB12_HUMAN | Ras-related protein Rab-12 | Q6IQ22 |  | 0.72 | 1.15 |
| 1994 | RAB14_HUMAN | Ras-related protein Rab-14 | P61106 | 0.96 | 1.04 | 0.86 |
| 1995 | RAB18_HUMAN | Ras-related protein Rab-18 | Q9NP72 | 0.88 |  | 0.91 |
| 1996 | RAB1A_HUMAN | Ras-related protein Rab-1A | P62820 | 0.75 | 0.96 | 1.03 |
| 1997 | RAB21_HUMAN | Ras-related protein Rab-21 | Q9UL25 | 0.85 | 0.92 | 0.85 |
| 1998 | RAB2A_HUMAN | Ras-related protein Rab-2A | P61019 | 1.09 | 0.69 | 0.96 |
| 1999 | RAB35_HUMAN | Ras-related protein Rab-35 | Q15286 | 2.99 | 0.69 | 1.22 |
| 2000 | RAB4A_HUMAN | Ras-related protein Rab-4A | P20338 | 2.51 | 0.44 | 0.55 |
| 2001 | RAB5A_HUMAN | Ras-related protein Rab-5A | P20339 | 1.07 | 1.56 | 1.07 |
| 2002 | RAB5B_HUMAN | Ras-related protein Rab-5B | P61020 |  | 0.58 | 0.95 |
| 2003 | RAB5C_HUMAN | Ras-related protein Rab-5C | P51148 | 1.29 | 1.28 | 0.94 |
| 2004 | RAB6A_HUMAN | Ras-related protein Rab-6A | P20340 | 0.95 | 1.41 | 0.78 |
| 2005 | RAB7A_HUMAN | Ras-related protein Rab-7a | P51149 | 0.72 |  | 1.02 |
| 2006 | RAB8B_HUMAN | Ras-related protein Rab-8B | Q92930 |  | 0.96 |  |
| 2007 | RAB9A_HUMAN | Ras-related protein Rab-9A | P51151 | 2.63 |  | 0.97 |
| 2008 | RABL3_HUMAN | Rab-like protein 3 | Q5HYI8 |  | 1.07 | 1.22 |
| 2009 | RAC1_HUMAN | Ras-related C3 botulinum toxin substrate 1 | P63000 | 1.34 | 1.39 | 1.09 |
| 2010 | RAD_HUMAN | GTP-binding protein RAD | P55042 |  | 1.09 | 1.06 |
| 2011 | RADI_HUMAN | Radixin | P35241 | 0.54 | 1.02 | 1.00 |
| 2012 | RAIN_HUMAN | Ras-interacting protein 1 | Q5U651 |  | 0.83 | 0.89 |
| 2013 | RALA_HUMAN | Ras-related protein Ral-A | P11233 | 1.67 |  | 0.92 |
| 2014 | RALB_HUMAN | Ras-related protein Ral-B | P11234 | 1.11 | 1.14 | 1.49 |
| 2015 | RALY_HUMAN | RNA-binding protein Raly | Q9UKM9 |  | 0.97 | 0.86 |
| 2016 | RAN_HUMAN | GTP-binding nuclear protein Ran | P62826 | 1.09 | 0.75 | 0.89 |
| 2017 | RANG_HUMAN | Ran-specific GTPase-activating protein | P43487 | 0.92 | 1.29 | 0.72 |
| 2018 | RAP1A_HUMAN | Ras-related protein Rap-1A | P62834 | 0.82 | 0.94 | 1.59 |
| 2019 | RAP1B_HUMAN | Ras-related protein Rap-1b | P61224 | 1.08 | 0.53 |  |
| 2020 | RAP2C_HUMAN | Ras-related protein Rap-2c | Q9Y3L5 |  | 0.96 | 1.47 |
| 2021 | RB11B_HUMAN | Ras-related protein Rab-11B | Q15907 | 1.54 |  | 1.06 |
| 2022 | RB6I2_HUMAN | ELKS/Rab6-interacting/CAST family member 1 | Q8IUD2 | 1.75 | 1.02 | 0.92 |
| 2023 | RBBP4_HUMAN | Histone-binding protein RBBP4 | Q09028 | 1.12 |  | 0.98 |
| 2024 | RBG1L_HUMAN | Rab GTPase-activating protein 1-like | Q5R372 | 1.10 | 1.06 | 1.03 |
| 2025 | RBGPR_HUMAN | Rab3 GTPase-activating protein non-catalytic subunit | Q9H2M9 | 1.49 | 0.76 | 1.32 |
| 2026 | RBM20_HUMAN | RNA-binding protein 20 | Q5T481 | 0.28 |  |  |
| 2027 | RBM3_HUMAN | RNA-binding protein 3 | P98179 | 0.03 |  | 0.83 |
| 2028 | RBM39_HUMAN | RNA-binding protein 39 | Q14498 |  | 2.03 | 1.18 |
| 2029 | RBM8A_HUMAN | RNA-binding protein 8A | Q9Y5S9 | 1.91 |  | 0.88 |
| 2030 | RBMX_HUMAN | RNA-binding motif protein, X chromosome | P38159 | 0.98 | 1.15 | 1.08 |
| 2031 | RBP2_HUMAN | E3 SUMO-protein ligase RanBP2 | P49792 |  | 0.90 | 1.35 |
| 2032 | RBP56_HUMAN | TATA-binding protein-associated factor 2N | Q92804 |  | 2.09 | 0.57 |
| 2033 | RBPMS_HUMAN | RNA-binding protein with multiple splicing | Q93062 |  | 1.14 | 1.18 |
| 2034 | RBSK_HUMAN | Ribokinase | Q9H477 | 0.94 |  | 0.40 |
| 2035 | RCN1_HUMAN | Reticulocalbin-1 | Q15293 | 0.49 | 0.62 | 0.81 |
| 2036 | RCN2_HUMAN | Reticulocalbin-2 | Q14257 | 2.15 | 1.22 | 0.80 |
| 2037 | RCN3_HUMAN | Reticulocalbin-3 | Q96D15 | 0.95 | 0.66 | 0.98 |
| 2038 | RD23A_HUMAN | UV excision repair protein RAD23 homolog A | P54725 | 2.31 | 0.94 | 1.03 |
| 2039 | RD23B_HUMAN | UV excision repair protein RAD23 homolog B | P54727 | 2.07 | 0.72 | 1.04 |
| 2040 | RDH13_HUMAN | Retinol dehydrogenase 13 | Q8NBN7 | 0.98 | 0.74 | 1.01 |
| 2041 | RDH14_HUMAN | Retinol dehydrogenase 14 | Q9HBH5 | 1.24 |  | 1.04 |
| 2042 | RECQ1_HUMAN | ATP-dependent DNA helicase Q1 | P46063 |  |  | 1.09 |
| 2043 | REEP5_HUMAN | Receptor expression-enhancing protein 5 | Q00765 | 1.31 | 0.11 | 0.85 |
| 2044 | RENT1_HUMAN | Regulator of nonsense transcripts 1 | Q92900 | 0.93 | 1.14 | 1.42 |
| 2045 | RET1_HUMAN | Retinol-binding protein 1 | P09455 |  | 0.59 | 1.08 |
| 2046 | RET4_HUMAN | Retinol-binding protein 4 | P02753 | 0.94 | 1.02 | 1.08 |
| 2047 | RET7_HUMAN | Retinoid-binding protein 7 | Q96R05 |  | 1.29 | 1.63 |
| 2048 | RETN_HUMAN | Resistin | Q9HD89 |  | 1.18 | 0.77 |
| 2049 | RFA1_HUMAN | Replication protein A 70 kDa DNA-binding subunit | P27694 | 0.70 | 1.12 | 1.02 |
| 2050 | RFA3_HUMAN | Replication protein A 14 kDa subunit | P35244 | 1.42 | 1.33 | 0.92 |
| 2051 | RFIP5_HUMAN | Rab11 family-interacting protein 5 | Q9BXF6 |  | 1.10 | 0.69 |
| 2052 | RFTN1_HUMAN | Raftlin | Q14699 |  | 1.69 | 1.02 |
| 2053 | RHG01_HUMAN | Rho GTPase-activating protein 1 | Q07960 |  | 0.79 | 1.09 |
| 2054 | RHG17_HUMAN | Rho GTPase-activating protein 17 | Q68EM7 | 1.36 | 1.37 | 0.94 |
| 2055 | RHOA_HUMAN | Transforming protein RhoA | P61586 | 1.02 | 1.53 | 4.72 |
| 2056 | RHOC_HUMAN | Rho-related GTP-binding protein RhoC | P08134 | 0.99 | 1.74 | 0.67 |
| 2057 | RHOG_HUMAN | Rho-related GTP-binding protein RhoG | P84095 | 1.69 | 1.06 | 0.90 |
| 2058 | RIC8A_HUMAN | Synembryn-A | Q9NPQ8 | 0.48 | 1.85 | 0.60 |
| 2059 | RILP_HUMAN | Rab-interacting lysosomal protein | Q96NA2 |  | 0.90 | 1.18 |
| 2060 | RINI_HUMAN | Ribonuclease inhibitor | P13489 | 0.69 |  | 0.98 |
| 2061 | RL10_HUMAN | 60S ribosomal protein L10 | P27635 | 1.28 | 2.07 | 0.53 |
| 2062 | RL10A_HUMAN | 60S ribosomal protein L10a | P62906 | 1.05 |  | 0.93 |
| 2063 | RL11_HUMAN | 60S ribosomal protein L11 | P62913 | 0.90 | 0.95 | 0.74 |
| 2064 | RL12_HUMAN | 60S ribosomal protein L12 | P30050 | 1.31 | 0.95 | 1.53 |
| 2065 | RL13_HUMAN | 60S ribosomal protein L13 | P26373 | 1.17 | 1.72 | 0.98 |
| 2066 | RL13A_HUMAN | 60S ribosomal protein L13a | P40429 | 1.51 | 0.87 | 1.14 |
| 2067 | RL14_HUMAN | 60S ribosomal protein L14 | P50914 | 0.54 | 0.82 |  |
| 2068 | RL15_HUMAN | 60S ribosomal protein L15 | P61313 | 1.21 | 0.91 | 0.97 |
| 2069 | RL17_HUMAN | 60S ribosomal protein L17 | P18621 | 0.59 |  | 1.15 |
| 2070 | RL18_HUMAN | 60S ribosomal protein L18 | Q07020 | 1.21 | 0.90 | 0.80 |
| 2071 | RL18A_HUMAN | 60S ribosomal protein L18a | Q02543 | 1.67 |  | 1.12 |
| 2072 | RL19_HUMAN | 60S ribosomal protein L19 | P84098 | 1.09 | 0.82 |  |
| 2073 | RL21_HUMAN | 60S ribosomal protein L21 | P46778 | 0.61 | 1.22 | 1.37 |
| 2074 | RL22_HUMAN | 60S ribosomal protein L22 | P35268 | 1.41 | 0.48 | 0.73 |
| 2075 | RL23_HUMAN | 60S ribosomal protein L23 | P62829 | 1.58 | 1.46 | 1.25 |
| 2076 | RL23A_HUMAN | 60S ribosomal protein L23a | P62750 | 0.88 | 0.52 |  |
| 2077 | RL24_HUMAN | 60S ribosomal protein L24 | P83731 | 0.94 | 1.07 | 0.97 |
| 2078 | RL26_HUMAN | 60S ribosomal protein L26 | P61254 | 1.03 | 0.81 | 1.03 |
| 2079 | RL27_HUMAN | 60S ribosomal protein L27 | P61353 |  | 0.89 | 0.93 |
| 2080 | RL27A_HUMAN | 60S ribosomal protein L27a | P46776 | 0.64 | 0.65 | 0.63 |
| 2081 | RL28_HUMAN | 60S ribosomal protein L28 | P46779 | 1.02 | 1.07 | 1.08 |
| 2082 | RL29_HUMAN | 60S ribosomal protein L29 | P47914 | 1.77 | 2.54 | 0.73 |
| 2083 | RL3_HUMAN | 60S ribosomal protein L3 | P39023 | 1.14 |  | 1.08 |
| 2084 | RL30_HUMAN | 60S ribosomal protein L30 | P62888 | 0.96 | 1.77 | 1.26 |
| 2085 | RL31_HUMAN | 60S ribosomal protein L31 | P62899 | 1.96 | 0.89 | 0.79 |
| 2086 | RL32_HUMAN | 60S ribosomal protein L32 | P62910 |  | 1.11 | 1.29 |
| 2087 | RL34_HUMAN | 60S ribosomal protein L34 | P49207 | 1.13 |  | 1.29 |
| 2088 | RL35_HUMAN | 60S ribosomal protein L35 | P42766 | 0.93 |  | 0.66 |
| 2089 | RL35A_HUMAN | 60S ribosomal protein L35a | P18077 | 0.68 | 0.79 | 0.62 |
| 2090 | RL36_HUMAN | 60S ribosomal protein L36 | Q9Y3U8 | 1.02 | 2.05 | 0.74 |
| 2091 | RL36L_HUMAN | 60S ribosomal protein L36a-like | Q969Q0 | 2.17 | 0.40 |  |
| 2092 | RL37A_HUMAN | 60S ribosomal protein L37a | P61513 | 3.34 | 1.46 | 1.24 |
| 2093 | RL38_HUMAN | 60S ribosomal protein L38 | P63173 | 1.96 | 1.42 | 1.08 |
| 2094 | RL39_HUMAN | 60S ribosomal protein L39 | P62891 | 0.85 |  | 1.85 |
| 2095 | RL3L_HUMAN | 60S ribosomal protein L3-like | Q92901 | 0.95 | 0.80 | 0.92 |
| 2096 | RL4_HUMAN | 60S ribosomal protein L4 | P36578 | 0.86 | 1.56 | 0.81 |
| 2097 | RL5_HUMAN | 60S ribosomal protein L5 | P46777 | 1.09 | 1.38 | 1.37 |
| 2098 | RL6_HUMAN | 60S ribosomal protein L6 | Q02878 | 0.99 |  | 0.91 |
| 2099 | RL7_HUMAN | 60S ribosomal protein L7 | P18124 |  | 0.41 | 0.91 |
| 2100 | RL7A_HUMAN | 60S ribosomal protein L7a | P62424 | 0.44 | 0.71 | 1.37 |
| 2101 | RL8_HUMAN | 60S ribosomal protein L8 | P62917 | 0.77 | 1.28 | 1.13 |
| 2102 | RL9_HUMAN | 60S ribosomal protein L9 | P32969 | 1.05 |  | 1.00 |
| 2103 | RLA0_HUMAN | 60S acidic ribosomal protein P0 | P05388 |  | 1.32 | 0.96 |
| 2104 | RLA1_HUMAN | 60S acidic ribosomal protein P1 | P05386 | 1.27 | 0.94 | 1.09 |
| 2105 | RLA2_HUMAN | 60S acidic ribosomal protein P2 | P05387 | 1.16 | 0.82 | 0.98 |
| 2106 | RM01_HUMAN | 39S ribosomal protein L1, mitochondrial | Q9BYD6 |  |  | 0.23 |
| 2107 | RM03_HUMAN | 39S ribosomal protein L3, mitochondrial | P09001 |  | 0.49 | 0.94 |
| 2108 | RM04_HUMAN | 39S ribosomal protein L4, mitochondrial | Q9BYD3 | 1.79 | 1.05 | 0.90 |
| 2109 | RM09_HUMAN | 39S ribosomal protein L9, mitochondrial | Q9BYD2 |  | 1.24 | 0.92 |
| 2110 | RM11_HUMAN | 39S ribosomal protein L11, mitochondrial | Q9Y3B7 | 1.11 | 1.03 | 0.54 |
| 2111 | RM12_HUMAN | 39S ribosomal protein L12, mitochondrial | P52815 | 0.93 |  | 1.31 |
| 2112 | RM13_HUMAN | 39S ribosomal protein L13, mitochondrial | Q9BYD1 |  | 0.99 | 0.84 |
| 2113 | RM14_HUMAN | 39S ribosomal protein L14, mitochondrial | Q6P1L8 | 0.92 |  | 0.82 |
| 2114 | RM15_HUMAN | 39S ribosomal protein L15, mitochondrial | Q9P015 | 1.64 | 1.17 | 0.99 |
| 2115 | RM19_HUMAN | 39S ribosomal protein L19, mitochondrial | P49406 |  |  | 0.80 |
| 2116 | RM21_HUMAN | 39S ribosomal protein L21, mitochondrial | Q7Z2W9 | 1.11 | 0.50 |  |
| 2117 | RM22_HUMAN | 39S ribosomal protein L22, mitochondrial | Q9NWU5 | 2.13 |  | 0.90 |
| 2118 | RM23_HUMAN | 39S ribosomal protein L23, mitochondrial | Q16540 | 1.00 | 1.66 | 2.05 |
| 2119 | RM24_HUMAN | 39S ribosomal protein L24, mitochondrial | Q96A35 | 1.16 |  | 1.19 |
| 2120 | RM28_HUMAN | 39S ribosomal protein L28, mitochondrial | Q13084 | 1.89 | 1.61 | 1.04 |
| 2121 | RM37_HUMAN | 39S ribosomal protein L37, mitochondrial | Q9BZE1 | 1.57 | 1.28 | 0.98 |
| 2122 | RM38_HUMAN | 39S ribosomal protein L38, mitochondrial | Q96DV4 |  | 1.38 | 0.84 |
| 2123 | RM39_HUMAN | 39S ribosomal protein L39, mitochondrial | Q9NYK5 | 0.86 | 0.41 | 1.32 |
| 2124 | RM40_HUMAN | 39S ribosomal protein L40, mitochondrial | Q9NQ50 | 1.19 | 1.28 | 0.83 |
| 2125 | RM41_HUMAN | 39S ribosomal protein L41, mitochondrial | Q8IXM3 |  | 1.71 | 0.66 |
| 2126 | RM43_HUMAN | 39S ribosomal protein L43, mitochondrial | Q8N983 |  | 0.70 | 0.69 |
| 2127 | RM44_HUMAN | 39S ribosomal protein L44, mitochondrial | Q9H9J2 | 0.63 | 0.82 | 0.95 |
| 2128 | RM46_HUMAN | 39S ribosomal protein L46, mitochondrial | Q9H2W6 | 0.28 | 1.04 | 1.08 |
| 2129 | RM49_HUMAN | 39S ribosomal protein L49, mitochondrial | Q13405 | 0.15 | 1.31 | 0.80 |
| 2130 | RM50_HUMAN | 39S ribosomal protein L50, mitochondrial | Q8N5N7 | 0.90 | 1.10 | 0.70 |
| 2131 | RM54_HUMAN | 39S ribosomal protein L54, mitochondrial | Q6P161 | 2.11 |  | 0.50 |
| 2132 | RM55_HUMAN | 39S ribosomal protein L55, mitochondrial | Q7Z7F7 | 1.50 | 1.91 | 0.44 |
| 2133 | RMD1_HUMAN | Regulator of microtubule dynamics protein 1 | Q96DB5 | 1.11 | 0.63 | 0.94 |
| 2134 | RMD3_HUMAN | Regulator of microtubule dynamics protein 3 | Q96TC7 | 0.91 | 0.69 | 0.75 |
| 2135 | RMND1_HUMAN | Required for meiotic nuclear division protein 1 homolog | Q9NWS8 |  | 0.44 | 1.11 |
| 2136 | RO60_HUMAN | 60 kDa SS-A/Ro ribonucleoprotein | P10155 | 1.24 | 1.77 | 0.91 |
| 2137 | ROA0_HUMAN | Heterogeneous nuclear ribonucleoprotein A0 | Q13151 | 0.96 | 1.11 | 1.18 |
| 2138 | ROA1_HUMAN | Heterogeneous nuclear ribonucleoprotein A1 | P09651 | 1.56 | 1.06 | 1.31 |
| 2139 | ROA2_HUMAN | Heterogeneous nuclear ribonucleoproteins A2/B1 | P22626 | 1.54 | 0.97 | 0.89 |
| 2140 | ROA3_HUMAN | Heterogeneous nuclear ribonucleoprotein A3 | P51991 | 0.68 | 1.36 | 1.19 |
| 2141 | ROAA_HUMAN | Heterogeneous nuclear ribonucleoprotein A/B | Q99729 | 1.94 | 1.31 | 1.15 |
| 2142 | ROCK1_HUMAN | Rho-associated protein kinase 1 | Q13464 | 2.31 | 0.61 | 0.84 |
| 2143 | ROCK2_HUMAN | Rho-associated protein kinase 2 | O75116 | 3.19 | 0.20 | 1.09 |
| 2144 | ROMO1_HUMAN | Reactive oxygen species modulator 1 | P60602 |  | 1.08 | 1.54 |
| 2145 | RPB2_HUMAN | DNA-directed RNA polymerase II subunit RPB2 | P30876 |  | 2.17 | 1.73 |
| 2146 | RPIA_HUMAN | Ribose-5-phosphate isomerase | P49247 |  | 0.39 | 0.79 |
| 2147 | RPN1_HUMAN | Dolichyl-diphosphooligosaccharide--protein glycosyltransferase subunit 1 | P04843 | 1.06 | 1.09 | 0.88 |
| 2148 | RPN2_HUMAN | Dolichyl-diphosphooligosaccharide--protein glycosyltransferase subunit 2 | P04844 | 2.01 |  | 1.09 |
| 2149 | RRAGC_HUMAN | Ras-related GTP-binding protein C | Q9HB90 |  | 1.71 | 0.98 |
| 2150 | RRAS_HUMAN | Ras-related protein R-Ras | P10301 | 0.91 | 1.67 | 1.24 |
| 2151 | RRAS2_HUMAN | Ras-related protein R-Ras2 | P62070 | 0.74 | 0.62 | 0.92 |
| 2152 | RRBP1_HUMAN | Ribosome-binding protein 1 | Q9P2E9 | 3.94 | 0.71 | 1.00 |
| 2153 | RRF2M_HUMAN | Ribosome-releasing factor 2, mitochondrial | Q969S9 | 1.17 | 1.16 | 0.97 |
| 2154 | RRFM_HUMAN | Ribosome-recycling factor, mitochondrial | Q96E11 | 1.09 | 1.05 | 1.10 |
| 2155 | RS10_HUMAN | 40S ribosomal protein S10 | P46783 | 1.01 | 0.76 | 1.07 |
| 2156 | RS11_HUMAN | 40S ribosomal protein S11 | P62280 | 1.18 | 0.70 | 0.92 |
| 2157 | RS12_HUMAN | 40S ribosomal protein S12 | P25398 |  | 0.68 | 1.04 |
| 2158 | RS13_HUMAN | 40S ribosomal protein S13 | P62277 | 1.13 | 0.93 | 1.11 |
| 2159 | RS14_HUMAN | 40S ribosomal protein S14 | P62263 | 1.79 | 5.35 | 1.12 |
| 2160 | RS15_HUMAN | 40S ribosomal protein S15 | P62841 | 1.25 | 1.08 | 0.97 |
| 2161 | RS15A_HUMAN | 40S ribosomal protein S15a | P62244 | 0.86 | 0.69 | 1.10 |
| 2162 | RS16_HUMAN | 40S ribosomal protein S16 | P62249 | 1.25 | 1.46 | 1.02 |
| 2163 | RS18_HUMAN | 40S ribosomal protein S18 | P62269 | 5.11 | 1.42 | 0.87 |
| 2164 | RS19_HUMAN | 40S ribosomal protein S19 | P39019 | 0.85 | 1.10 | 0.71 |
| 2165 | RS2_HUMAN | 40S ribosomal protein S2 | P15880 | 0.98 | 0.45 | 1.17 |
| 2166 | RS20_HUMAN | 40S ribosomal protein S20 | P60866 | 1.51 | 0.60 | 0.76 |
| 2167 | RS21_HUMAN | 40S ribosomal protein S21 | P63220 | 0.73 | 0.96 |  |
| 2168 | RS23_HUMAN | 40S ribosomal protein S23 | P62266 | 0.54 | 0.74 | 0.80 |
| 2169 | RS24_HUMAN | 40S ribosomal protein S24 | P62847 | 0.22 |  | 0.94 |
| 2170 | RS25_HUMAN | 40S ribosomal protein S25 | P62851 | 0.96 | 0.70 | 0.93 |
| 2171 | RS26_HUMAN | 40S ribosomal protein S26 | P62854 | 0.21 |  | 0.92 |
| 2172 | RS28_HUMAN | 40S ribosomal protein S28 | P62857 | 0.57 | 1.31 |  |
| 2173 | RS3_HUMAN | 40S ribosomal protein S3 | P23396 | 1.10 | 0.64 | 0.94 |
| 2174 | RS30_HUMAN | 40S ribosomal protein S30 | P62861 |  | 1.85 | 1.14 |
| 2175 | RS3A_HUMAN | 40S ribosomal protein S3a | P61247 | 1.42 |  | 0.96 |
| 2176 | RS4X_HUMAN | 40S ribosomal protein S4, X isoform | P62701 | 0.69 | 1.50 | 0.99 |
| 2177 | RS5_HUMAN | 40S ribosomal protein S5 | P46782 |  | 0.84 | 0.85 |
| 2178 | RS6_HUMAN | 40S ribosomal protein S6 | P62753 | 0.50 | 1.29 | 0.64 |
| 2179 | RS7_HUMAN | 40S ribosomal protein S7 | P62081 | 0.78 | 1.15 | 0.78 |
| 2180 | RS8_HUMAN | 40S ribosomal protein S8 | P62241 | 0.48 | 1.04 | 0.50 |
| 2181 | RS9_HUMAN | 40S ribosomal protein S9 | P46781 | 1.01 | 0.99 | 0.91 |
| 2182 | RSSA_HUMAN | 40S ribosomal protein SA | P08865 |  |  | 0.88 |
| 2183 | RSU1_HUMAN | Ras suppressor protein 1 | Q15404 | 0.74 | 1.12 | 0.97 |
| 2184 | RT05_HUMAN | 28S ribosomal protein S5, mitochondrial | P82675 |  | 1.31 | 0.82 |
| 2185 | RT07_HUMAN | 28S ribosomal protein S7, mitochondrial | Q9Y2R9 | 0.98 | 0.98 | 0.96 |
| 2186 | RT09_HUMAN | 28S ribosomal protein S9, mitochondrial | P82933 |  |  | 1.03 |
| 2187 | RT10_HUMAN | 28S ribosomal protein S10, mitochondrial | P82664 |  | 1.03 | 1.12 |
| 2188 | RT11_HUMAN | 28S ribosomal protein S11, mitochondrial | P82912 |  |  |  |
| 2189 | RT14_HUMAN | 28S ribosomal protein S14, mitochondrial | O60783 | 1.79 | 1.18 | 0.93 |
| 2190 | RT15_HUMAN | 28S ribosomal protein S15, mitochondrial | P82914 | 0.93 |  |  |
| 2191 | RT16_HUMAN | 28S ribosomal protein S16, mitochondrial | Q9Y3D3 | 0.64 |  | 0.78 |
| 2192 | RT17_HUMAN | 28S ribosomal protein S17, mitochondrial | Q9Y2R5 | 0.94 | 1.12 | 0.98 |
| 2193 | RT18B_HUMAN | 28S ribosomal protein S18b, mitochondrial | Q9Y676 | 0.66 | 0.96 | 0.82 |
| 2194 | RT18C_HUMAN | 28S ribosomal protein S18c, mitochondrial | Q9Y3D5 |  | 1.24 | 0.78 |
| 2195 | RT21_HUMAN | 28S ribosomal protein S21, mitochondrial | P82921 | 1.22 | 1.12 | 0.93 |
| 2196 | RT22_HUMAN | 28S ribosomal protein S22, mitochondrial | P82650 | 0.94 | 0.49 | 1.00 |
| 2197 | RT23_HUMAN | 28S ribosomal protein S23, mitochondrial | Q9Y3D9 | 1.38 | 1.05 | 1.03 |
| 2198 | RT24_HUMAN | 28S ribosomal protein S24, mitochondrial | Q96EL2 | 1.89 |  | 1.02 |
| 2199 | RT25_HUMAN | 28S ribosomal protein S25, mitochondrial | P82663 | 0.24 | 0.57 | 1.08 |
| 2200 | RT26_HUMAN | 28S ribosomal protein S26, mitochondrial | Q9BYN8 | 3.66 | 2.56 | 0.76 |
| 2201 | RT27_HUMAN | 28S ribosomal protein S27, mitochondrial | Q92552 |  |  | 1.21 |
| 2202 | RT28_HUMAN | 28S ribosomal protein S28, mitochondrial | Q9Y2Q9 | 1.11 | 0.86 | 0.73 |
| 2203 | RT29_HUMAN | 28S ribosomal protein S29, mitochondrial | P51398 | 0.49 |  | 1.08 |
| 2204 | RT30_HUMAN | 28S ribosomal protein S30, mitochondrial | Q9NP92 | 0.70 | 0.55 |  |
| 2205 | RT31_HUMAN | 28S ribosomal protein S31, mitochondrial | Q92665 | 0.77 |  | 1.13 |
| 2206 | RT34_HUMAN | 28S ribosomal protein S34, mitochondrial | P82930 | 1.54 | 1.53 | 1.10 |
| 2207 | RT35_HUMAN | 28S ribosomal protein S35, mitochondrial | P82673 | 1.05 | 2.42 | 2.24 |
| 2208 | RT36_HUMAN | 28S ribosomal protein S36, mitochondrial | P82909 | 1.19 | 0.69 | 1.17 |
| 2209 | RT4I1_HUMAN | Reticulon-4-interacting protein 1, mitochondrial | Q8WWV3 | 0.78 | 2.15 | 1.41 |
| 2210 | RTCA_HUMAN | RNA 3'-terminal phosphate cyclase | O00442 | 1.07 | 0.52 | 0.53 |
| 2211 | RTCB_HUMAN | tRNA-splicing ligase RtcB homolog | Q9Y3I0 | 0.98 | 0.52 | 1.12 |
| 2212 | RTN2_HUMAN | Reticulon-2 | O75298 |  | 0.63 | 1.27 |
| 2213 | RTN3_HUMAN | Reticulon-3 | O95197 | 0.86 | 0.92 | 0.82 |
| 2214 | RTN4_HUMAN | Reticulon-4 | Q9NQC3 | 0.77 | 3.05 | 1.09 |
| 2215 | RU17_HUMAN | U1 small nuclear ribonucleoprotein 70 kDa | P08621 | 0.36 |  | 1.00 |
| 2216 | RUVB1_HUMAN | RuvB-like 1 | Q9Y265 | 1.29 | 1.25 | 0.87 |
| 2217 | RUVB2_HUMAN | RuvB-like 2 | Q9Y230 | 0.21 | 1.19 |  |
| 2218 | RUXE_HUMAN | Small nuclear ribonucleoprotein E | P62304 |  | 0.90 | 0.65 |
| 2219 | RUXG_HUMAN | Small nuclear ribonucleoprotein G | P62308 |  |  | 0.68 |
| 2220 | RYR2_HUMAN | Ryanodine receptor 2 | Q92736 | 0.47 | 0.69 | 1.14 |
| 2221 | S100B_HUMAN | Protein S100-B | P04271 | 0.69 | 0.93 | 1.04 |
| 2222 | S10A1_HUMAN | Protein S100-A1 | P23297 | 0.86 | 0.78 | 0.70 |
| 2223 | S10A4_HUMAN | Protein S100-A4 | P26447 | 0.69 |  | 1.33 |
| 2224 | S10A6_HUMAN | Protein S100-A6 | P06703 | 0.95 | 0.66 | 1.32 |
| 2225 | S10A8_HUMAN | Protein S100-A8 | P05109 |  | 2.15 | 0.77 |
| 2226 | S10A9_HUMAN | Protein S100-A9 | P06702 | 0.22 | 0.79 | 1.46 |
| 2227 | S10AA_HUMAN | Protein S100-A10 | P60903 | 1.21 | 1.12 | 0.90 |
| 2228 | S10AB_HUMAN | Protein S100-A11 | P31949 | 0.59 | 0.63 | 1.01 |
| 2229 | S10AD_HUMAN | Protein S100-A13 | Q99584 | 1.05 | 0.46 | 1.09 |
| 2230 | S10AG_HUMAN | Protein S100-A16 | Q96FQ6 | 0.44 | 1.12 | 0.90 |
| 2231 | S2542_HUMAN | Mitochondrial coenzyme A transporter SLC25A42 | Q86VD7 | 1.39 | 0.52 | 1.93 |
| 2232 | S2546_HUMAN | Solute carrier family 25 member 46 | Q96AG3 | 1.03 | 0.15 | 0.60 |
| 2233 | S27A1_HUMAN | Long-chain fatty acid transport protein 1 | Q6PCB7 | 1.11 | 1.53 | 1.42 |
| 2234 | S27A6_HUMAN | Long-chain fatty acid transport protein 6 | Q9Y2P4 | 1.02 |  | 1.09 |
| 2235 | S29A1_HUMAN | Equilibrative nucleoside transporter 1 | Q99808 |  | 0.90 | 0.82 |
| 2236 | S39AE_HUMAN | Zinc transporter ZIP14 | Q15043 | 1.15 | 0.93 | 1.12 |
| 2237 | S4A7_HUMAN | Sodium bicarbonate cotransporter 3 | Q9Y6M7 | 0.48 |  | 1.06 |
| 2238 | SAA4_HUMAN | Serum amyloid A-4 protein | P35542 | 1.27 |  | 0.96 |
| 2239 | SAC1_HUMAN | Phosphatidylinositide phosphatase SAC1 | Q9NTJ5 | 1.05 | 1.22 | 1.13 |
| 2240 | SAE1_HUMAN | SUMO-activating enzyme subunit 1 | Q9UBE0 | 1.77 | 1.14 | 1.26 |
| 2241 | SAE2_HUMAN | SUMO-activating enzyme subunit 2 | Q9UBT2 | 0.60 | 1.03 | 1.09 |
| 2242 | SAHH_HUMAN | Adenosylhomocysteinase | P23526 | 1.47 | 0.92 | 1.00 |
| 2243 | SAHH2_HUMAN | Putative adenosylhomocysteinase 2 | O43865 | 2.05 |  | 0.91 |
| 2244 | SAM50_HUMAN | Sorting and assembly machinery component 50 homolog | Q9Y512 | 1.20 |  | 0.87 |
| 2245 | SAMH1_HUMAN | Deoxynucleoside triphosphate triphosphohydrolase SAMHD1 | Q9Y3Z3 |  | 1.84 | 0.90 |
| 2246 | SAMP_HUMAN | Serum amyloid P-component | P02743 | 0.49 | 1.54 | 1.06 |
| 2247 | SAP_HUMAN | Prosaposin | P07602 | 0.82 | 0.40 | 1.16 |
| 2248 | SAP18_HUMAN | Histone deacetylase complex subunit SAP18 | O00422 | 0.27 |  | 0.65 |
| 2249 | SAP3_HUMAN | Ganglioside GM2 activator | P17900 | 0.63 | 3.13 | 1.06 |
| 2250 | SAR1A_HUMAN | GTP-binding protein SAR1a | Q9NR31 | 1.16 | 0.71 | 0.98 |
| 2251 | SAR1B_HUMAN | GTP-binding protein SAR1b | Q9Y6B6 | 1.21 | 1.14 | 1.34 |
| 2252 | SARNP_HUMAN | SAP domain-containing ribonucleoprotein | P82979 |  | 0.86 | 1.09 |
| 2253 | SBDS_HUMAN | Ribosome maturation protein SBDS | Q9Y3A5 | 0.88 | 1.17 | 0.97 |
| 2254 | SBP1_HUMAN | Selenium-binding protein 1 | Q13228 | 1.27 | 1.10 | 0.94 |
| 2255 | SC11A_HUMAN | Signal peptidase complex catalytic subunit SEC11A | P67812 |  | 0.58 | 1.27 |
| 2256 | SC22B_HUMAN | Vesicle-trafficking protein SEC22b | O75396 | 0.75 |  | 1.03 |
| 2257 | SC23A_HUMAN | Protein transport protein Sec23A | Q15436 |  | 0.64 | 0.95 |
| 2258 | SC24B_HUMAN | Protein transport protein Sec24B | O95487 | 0.82 | 1.34 | 0.92 |
| 2259 | SC24C_HUMAN | Protein transport protein Sec24C | P53992 |  | 1.69 | 0.86 |
| 2260 | SC31A_HUMAN | Protein transport protein Sec31A | O94979 | 0.95 |  | 0.97 |
| 2261 | SCAM1_HUMAN | Secretory carrier-associated membrane protein 1 | O15126 |  | 1.53 | 0.84 |
| 2262 | SCAM2_HUMAN | Secretory carrier-associated membrane protein 2 | O15127 | 1.18 | 1.01 | 0.67 |
| 2263 | SCAM3_HUMAN | Secretory carrier-associated membrane protein 3 | O14828 |  | 0.69 | 0.66 |
| 2264 | SCFD1_HUMAN | Sec1 family domain-containing protein 1 | Q8WVM8 | 0.94 | 0.90 | 1.34 |
| 2265 | SCMC1_HUMAN | Calcium-binding mitochondrial carrier protein SCaMC-1 | Q6NUK1 | 1.36 | 1.20 | 0.76 |
| 2266 | SCN7A_HUMAN | Sodium channel protein type 7 subunit alpha | Q01118 |  | 1.51 | 0.53 |
| 2267 | SCO1_HUMAN | Protein SCO1 homolog, mitochondrial | O75880 | 1.79 | 0.48 | 1.02 |
| 2268 | SCOC_HUMAN | Short coiled-coil protein | Q9UIL1 | 0.67 |  | 0.49 |
| 2269 | SCOT1_HUMAN | Succinyl-CoA:3-ketoacid coenzyme A transferase 1, mitochondrial | P55809 | 0.80 |  | 1.03 |
| 2270 | SCPDL_HUMAN | Saccharopine dehydrogenase-like oxidoreductase | Q8NBX0 | 1.50 |  | 1.14 |
| 2271 | SCRB2_HUMAN | Lysosome membrane protein 2 | Q14108 | 1.07 | 0.83 | 1.06 |
| 2272 | SCRN1_HUMAN | Secernin-1 | Q12765 |  | 1.03 | 0.94 |
| 2273 | SCRN2_HUMAN | Secernin-2 | Q96FV2 | 0.40 | 0.82 | 0.89 |
| 2274 | SCRN3_HUMAN | Secernin-3 | Q0VDG4 | 0.44 | 1.05 | 1.43 |
| 2275 | SDCB1_HUMAN | Syntenin-1 | O00560 | 0.79 | 1.21 | 0.95 |
| 2276 | SDHA_HUMAN | Succinate dehydrogenase [ubiquinone] flavoprotein subunit, mitochondrial | P31040 | 0.61 |  | 1.00 |
| 2277 | SDHB_HUMAN | Succinate dehydrogenase [ubiquinone] iron-sulfur subunit, mitochondrial | P21912 | 0.95 | 2.11 | 0.96 |
| 2278 | SDPR_HUMAN | Serum deprivation-response protein | O95810 | 1.45 | 0.77 | 0.95 |
| 2279 | SE1L1_HUMAN | Protein sel-1 homolog 1 | Q9UBV2 |  | 0.96 | 0.73 |
| 2280 | SEC13_HUMAN | Protein SEC13 homolog | P55735 | 0.98 | 1.07 | 0.86 |
| 2281 | SEC20_HUMAN | Vesicle transport protein SEC20 | Q12981 |  | 0.62 | 1.28 |
| 2282 | SEH1_HUMAN | Nucleoporin SEH1 | Q96EE3 |  | 1.32 | 1.21 |
| 2283 | SEP10_HUMAN | Septin-10 | Q9P0V9 | 1.07 |  | 0.88 |
| 2284 | SEP11_HUMAN | Septin-11 | Q9NVA2 | 1.00 | 0.95 | 1.13 |
| 2285 | SEPT2_HUMAN | Septin-2 | Q15019 | 0.76 | 1.28 | 1.07 |
| 2286 | SEPT7_HUMAN | Septin-7 | Q16181 | 0.86 |  | 0.82 |
| 2287 | SEPT8_HUMAN | Septin-8 | Q92599 | 2.15 |  | 0.70 |
| 2288 | SEPT9_HUMAN | Septin-9 | Q9UHD8 |  | 1.06 | 1.15 |
| 2289 | SERA_HUMAN | D-3-phosphoglycerate dehydrogenase | O43175 | 3.02 | 1.77 | 0.76 |
| 2290 | SERC1_HUMAN | Serine incorporator 1 | Q9NRX5 | 1.39 | 1.18 | 0.97 |
| 2291 | SERPH_HUMAN | Serpin H1 | P50454 |  | 0.93 | 0.64 |
| 2292 | SET_HUMAN | Protein SET | Q01105 | 1.42 |  | 1.33 |
| 2293 | SF01_HUMAN | Splicing factor 1 | Q15637 | 0.58 |  | 1.22 |
| 2294 | SF3B1_HUMAN | Splicing factor 3B subunit 1 | O75533 | 0.55 | 1.09 | 0.88 |
| 2295 | SF3B3_HUMAN | Splicing factor 3B subunit 3 | Q15393 | 1.47 | 1.34 | 0.67 |
| 2296 | SFPQ_HUMAN | Splicing factor, proline- and glutamine-rich | P23246 | 1.27 | 0.79 |  |
| 2297 | SFRP1_HUMAN | Secreted frizzled-related protein 1 | Q8N474 | 1.28 | 1.79 |  |
| 2298 | SFXN1_HUMAN | Sideroflexin-1 | Q9H9B4 | 1.39 | 0.84 | 1.00 |
| 2299 | SFXN3_HUMAN | Sideroflexin-3 | Q9BWM7 | 1.13 | 0.97 | 0.85 |
| 2300 | SFXN4_HUMAN | Sideroflexin-4 | Q6P4A7 | 1.58 | 0.75 | 0.63 |
| 2301 | SGCA_HUMAN | Alpha-sarcoglycan | Q16586 | 1.05 | 0.65 | 0.87 |
| 2302 | SGCB_HUMAN | Beta-sarcoglycan | Q16585 |  | 1.07 | 1.04 |
| 2303 | SGCD_HUMAN | Delta-sarcoglycan | Q92629 | 0.79 | 1.10 | 1.17 |
| 2304 | SGCE_HUMAN | Epsilon-sarcoglycan | O43556 | 1.75 | 0.54 | 1.34 |
| 2305 | SGCG_HUMAN | Gamma-sarcoglycan | Q13326 | 1.00 | 0.69 | 0.97 |
| 2306 | SGTA_HUMAN | Small glutamine-rich tetratricopeptide repeat-containing protein alpha | O43765 | 0.87 | 0.79 | 1.27 |
| 2307 | SH3BG_HUMAN | SH3 domain-binding glutamic acid-rich protein | P55822 | 1.15 |  | 0.81 |
| 2308 | SH3G1_HUMAN | Endophilin-A2 | Q99961 |  | 0.68 | 0.71 |
| 2309 | SH3L1_HUMAN | SH3 domain-binding glutamic acid-rich-like protein | O75368 | 1.64 | 0.81 | 0.96 |
| 2310 | SH3L3_HUMAN | SH3 domain-binding glutamic acid-rich-like protein 3 | Q9H299 | 1.06 | 2.05 | 1.25 |
| 2311 | SHLB1_HUMAN | Endophilin-B1 | Q9Y371 | 0.70 | 1.94 | 0.85 |
| 2312 | SHLB2_HUMAN | Endophilin-B2 | Q9NR46 | 0.77 | 0.95 | 0.85 |
| 2313 | SIAE_HUMAN | Sialate O-acetylesterase | Q9HAT2 | 1.16 | 0.90 | 1.34 |
| 2314 | SIAS_HUMAN | Sialic acid synthase | Q9NR45 | 1.34 |  | 1.59 |
| 2315 | SIAT9_HUMAN | Lactosylceramide alpha-2,3-sialyltransferase | Q9UNP4 |  |  | 0.83 |
| 2316 | SIR3_HUMAN | NAD-dependent protein deacetylase sirtuin-3, mitochondrial | Q9NTG7 | 5.81 | 1.24 | 1.06 |
| 2317 | SIR5_HUMAN | NAD-dependent protein deacylase sirtuin-5, mitochondrial | Q9NXA8 | 0.78 | 1.53 | 1.12 |
| 2318 | SKP1_HUMAN | S-phase kinase-associated protein 1 | P63208 | 0.85 | 1.09 | 0.93 |
| 2319 | SLIRP_HUMAN | SRA stem-loop-interacting RNA-binding protein, mitochondrial | Q9GZT3 | 0.88 | 1.87 | 0.73 |
| 2320 | SLK_HUMAN | STE20-like serine/threonine-protein kinase | Q9H2G2 | 0.44 | 0.67 | 0.87 |
| 2321 | SLMAP_HUMAN | Sarcolemmal membrane-associated protein | Q14BN4 | 2.07 | 1.05 | 1.01 |
| 2322 | SMC3_HUMAN | Structural maintenance of chromosomes protein 3 | Q9UQE7 | 2.81 | 1.00 | 0.95 |
| 2323 | SMCA4_HUMAN | Transcription activator BRG1 | P51532 |  | 6.14 | 0.98 |
| 2324 | SMD1_HUMAN | Small nuclear ribonucleoprotein Sm D1 | P62314 | 2.19 |  | 0.87 |
| 2325 | SMD2_HUMAN | Small nuclear ribonucleoprotein Sm D2 | P62316 |  | 1.02 | 1.07 |
| 2326 | SMD3_HUMAN | Small nuclear ribonucleoprotein Sm D3 | P62318 | 0.90 |  |  |
| 2327 | SMI20_HUMAN | Small integral membrane protein 20 | Q8N5G0 | 2.05 |  | 1.10 |
| 2328 | SMPX_HUMAN | Small muscular protein | Q9UHP9 | 1.04 | 0.71 | 1.10 |
| 2329 | SMTN_HUMAN | Smoothelin | P53814 | 1.12 | 0.85 | 1.20 |
| 2330 | SMYD1_HUMAN | Histone-lysine N-methyltransferase SMYD1 | Q8NB12 | 1.29 | 0.53 | 1.08 |
| 2331 | SNAA_HUMAN | Alpha-soluble NSF attachment protein | P54920 |  | 2.65 | 1.10 |
| 2332 | SNAG_HUMAN | Gamma-soluble NSF attachment protein | Q99747 | 1.14 |  | 0.72 |
| 2333 | SND1_HUMAN | Staphylococcal nuclease domain-containing protein 1 | Q7KZF4 | 1.39 | 0.68 | 1.05 |
| 2334 | SNF8_HUMAN | Vacuolar-sorting protein SNF8 | Q96H20 |  | 1.75 | 1.05 |
| 2335 | SNG2_HUMAN | Synaptogyrin-2 | O43760 |  | 0.65 | 0.92 |
| 2336 | SNP23_HUMAN | Synaptosomal-associated protein 23 | O00161 | 0.20 |  | 1.07 |
| 2337 | SNP29_HUMAN | Synaptosomal-associated protein 29 | O95721 | 0.64 |  | 0.93 |
| 2338 | SNR40_HUMAN | U5 small nuclear ribonucleoprotein 40 kDa protein | Q96DI7 | 0.68 |  | 0.99 |
| 2339 | SNRPA_HUMAN | U1 small nuclear ribonucleoprotein A | P09012 | 1.41 |  | 1.13 |
| 2340 | SNTA1_HUMAN | Alpha-1-syntrophin | Q13424 |  | 0.98 | 0.99 |
| 2341 | SNTB2_HUMAN | Beta-2-syntrophin | Q13425 |  | 1.11 | 0.88 |
| 2342 | SNX1_HUMAN | Sorting nexin-1 | Q13596 | 0.96 |  | 0.65 |
| 2343 | SNX12_HUMAN | Sorting nexin-12 | Q9UMY4 | 0.61 |  | 1.25 |
| 2344 | SNX17_HUMAN | Sorting nexin-17 | Q15036 | 0.97 |  | 0.81 |
| 2345 | SNX18_HUMAN | Sorting nexin-18 | Q96RF0 |  | 0.95 | 1.16 |
| 2346 | SNX2_HUMAN | Sorting nexin-2 | O60749 | 1.21 | 6.31 | 0.92 |
| 2347 | SNX3_HUMAN | Sorting nexin-3 | O60493 | 0.81 | 0.75 | 0.79 |
| 2348 | SNX6_HUMAN | Sorting nexin-6 | Q9UNH7 | 0.41 |  | 1.24 |
| 2349 | SNX9_HUMAN | Sorting nexin-9 | Q9Y5X1 | 1.05 | 0.77 | 0.76 |
| 2350 | SODC_HUMAN | Superoxide dismutase [Cu-Zn] | P00441 |  | 1.38 | 1.76 |
| 2351 | SODE_HUMAN | Extracellular superoxide dismutase [Cu-Zn] | P08294 |  | 1.06 | 1.08 |
| 2352 | SODM_HUMAN | Superoxide dismutase [Mn], mitochondrial | P04179 | 0.52 | 2.81 | 0.75 |
| 2353 | SORCN_HUMAN | Sorcin | P30626 | 1.07 | 0.52 | 1.09 |
| 2354 | SPB6_HUMAN | Serpin B6 | P35237 | 0.90 | 1.19 | 0.97 |
| 2355 | SPB9_HUMAN | Serpin B9 | P50453 | 0.92 |  | 0.92 |
| 2356 | SPEE_HUMAN | Spermidine synthase | P19623 | 1.22 |  | 0.95 |
| 2357 | SPEG_HUMAN | Striated muscle preferentially expressed protein kinase | Q15772 |  | 0.86 | 0.94 |
| 2358 | SPG7_HUMAN | Paraplegin | Q9UQ90 | 0.66 |  | 1.00 |
| 2359 | SPKAP_HUMAN | A-kinase anchor protein SPHKAP | Q2M3C7 | 0.80 | 0.94 | 0.90 |
| 2360 | SPN90_HUMAN | NCK-interacting protein with SH3 domain | Q9NZQ3 |  | 5.25 |  |
| 2361 | SPON1_HUMAN | Spondin-1 | Q9HCB6 | 0.75 | 1.85 | 1.10 |
| 2362 | SPRE_HUMAN | Sepiapterin reductase | P35270 | 1.27 |  | 0.91 |
| 2363 | SPRL1_HUMAN | SPARC-like protein 1 | Q14515 |  | 1.07 | 0.81 |
| 2364 | SPRY4_HUMAN | SPRY domain-containing protein 4 | Q8WW59 | 1.20 | 1.31 | 0.90 |
| 2365 | SPSY_HUMAN | Spermine synthase | P52788 | 1.12 | 0.81 | 1.07 |
| 2366 | SPT20_HUMAN | Spermatogenesis-associated protein 20 | Q8TB22 | 0.90 | 1.07 | 0.86 |
| 2367 | SPTA1_HUMAN | Spectrin alpha chain, erythrocytic 1 | P02549 | 0.85 | 0.81 | 0.93 |
| 2368 | SPTB1_HUMAN | Spectrin beta chain, erythrocytic | P11277 | 0.30 |  | 0.84 |
| 2369 | SPTB2_HUMAN | Spectrin beta chain, non-erythrocytic 1 | Q01082 | 0.98 |  | 1.04 |
| 2370 | SPTN1_HUMAN | Spectrin alpha chain, non-erythrocytic 1 | Q13813 | 0.49 |  | 1.20 |
| 2371 | SQRD_HUMAN | Sulfide:quinone oxidoreductase, mitochondrial | Q9Y6N5 | 1.60 | 1.25 | 0.67 |
| 2372 | SRBS1_HUMAN | Sorbin and SH3 domain-containing protein 1 | Q9BX66 | 0.95 |  | 0.97 |
| 2373 | SRBS2_HUMAN | Sorbin and SH3 domain-containing protein 2 | O94875 | 1.16 |  | 0.83 |
| 2374 | SRC8_HUMAN | Src substrate cortactin | Q14247 | 0.87 | 0.69 | 0.91 |
| 2375 | SRCA_HUMAN | Sarcalumenin | Q86TD4 | 0.85 | 0.76 | 1.49 |
| 2376 | SRCH_HUMAN | Sarcoplasmic reticulum histidine-rich calcium-binding protein | P23327 | 0.97 | 2.58 | 1.03 |
| 2377 | SRP14_HUMAN | Signal recognition particle 14 kDa protein | P37108 |  | 0.84 | 0.95 |
| 2378 | SRP54_HUMAN | Signal recognition particle 54 kDa protein | P61011 | 0.79 |  | 0.85 |
| 2379 | SRP68_HUMAN | Signal recognition particle subunit SRP68 | Q9UHB9 | 0.86 | 1.33 | 1.30 |
| 2380 | SRPR_HUMAN | Signal recognition particle receptor subunit alpha | P08240 | 1.20 | 0.95 | 1.02 |
| 2381 | SRPRB_HUMAN | Signal recognition particle receptor subunit beta | Q9Y5M8 | 0.46 | 0.92 | 0.99 |
| 2382 | SRPX_HUMAN | Sushi repeat-containing protein SRPX | P78539 | 1.10 |  | 1.12 |
| 2383 | SRRT_HUMAN | Serrate RNA effector molecule homolog | Q9BXP5 |  |  | 0.89 |
| 2384 | SRS10_HUMAN | Serine/arginine-rich splicing factor 10 | O75494 | 0.55 |  | 0.63 |
| 2385 | SRSF1_HUMAN | Serine/arginine-rich splicing factor 1 | Q07955 |  | 0.43 | 1.01 |
| 2386 | SRSF2_HUMAN | Serine/arginine-rich splicing factor 2 | Q01130 | 1.02 | 0.90 | 1.01 |
| 2387 | SRSF3_HUMAN | Serine/arginine-rich splicing factor 3 | P84103 | 1.22 | 4.13 | 0.99 |
| 2388 | SRSF7_HUMAN | Serine/arginine-rich splicing factor 7 | Q16629 | 2.86 | 1.12 |  |
| 2389 | SSBP_HUMAN | Single-stranded DNA-binding protein, mitochondrial | Q04837 | 0.75 | 1.82 | 0.93 |
| 2390 | SSDH_HUMAN | Succinate-semialdehyde dehydrogenase, mitochondrial | P51649 |  | 0.99 | 0.97 |
| 2391 | SSRA_HUMAN | Translocon-associated protein subunit alpha | P43307 |  | 1.13 | 0.83 |
| 2392 | SSRD_HUMAN | Translocon-associated protein subunit delta | P51571 | 1.22 | 1.80 | 0.99 |
| 2393 | SSRP1_HUMAN | FACT complex subunit SSRP1 | Q08945 |  | 1.07 | 1.49 |
| 2394 | ST1A4_HUMAN | Sulfotransferase 1A4 | P0DMN0 | 1.64 |  | 1.50 |
| 2395 | STA5B_HUMAN | Signal transducer and activator of transcription 5B | P51692 |  | 1.14 |  |
| 2396 | STAT1_HUMAN | Signal transducer and activator of transcription 1-alpha/beta | P42224 | 0.92 | 1.17 | 0.83 |
| 2397 | STAT3_HUMAN | Signal transducer and activator of transcription 3 | P40763 | 1.02 | 0.91 | 0.95 |
| 2398 | STAU2_HUMAN | Double-stranded RNA-binding protein Staufen homolog 2 | Q9NUL3 |  | 1.92 | 1.06 |
| 2399 | STBD1_HUMAN | Starch-binding domain-containing protein 1 | O95210 | 0.85 | 0.94 | 0.83 |
| 2400 | STEA4_HUMAN | Metalloreductase STEAP4 | Q687X5 |  | 1.12 | 0.76 |
| 2401 | STING_HUMAN | Stimulator of interferon genes protein | Q86WV6 | 1.04 | 1.02 | 0.66 |
| 2402 | STIP1_HUMAN | Stress-induced-phosphoprotein 1 | P31948 | 1.42 | 0.92 | 1.10 |
| 2403 | STK24_HUMAN | Serine/threonine-protein kinase 24 | Q9Y6E0 | 1.27 | 0.68 | 1.35 |
| 2404 | STML2_HUMAN | Stomatin-like protein 2, mitochondrial | Q9UJZ1 | 1.16 | 1.53 | 0.93 |
| 2405 | STMN1_HUMAN | Stathmin | P16949 | 0.94 | 1.08 | 0.89 |
| 2406 | STOM_HUMAN | Erythrocyte band 7 integral membrane protein | P27105 | 1.66 | 1.28 | 0.94 |
| 2407 | STRAP_HUMAN | Serine-threonine kinase receptor-associated protein | Q9Y3F4 |  | 2.38 | 0.78 |
| 2408 | STRN_HUMAN | Striatin | O43815 | 1.51 |  | 0.98 |
| 2409 | STRN3_HUMAN | Striatin-3 | Q13033 | 0.55 | 1.41 | 0.99 |
| 2410 | STRUM_HUMAN | WASH complex subunit strumpellin | Q12768 |  | 0.83 | 1.34 |
| 2411 | STT3A_HUMAN | Dolichyl-diphosphooligosaccharide--protein glycosyltransferase subunit STT3A | P46977 | 0.95 |  | 0.61 |
| 2412 | STT3B_HUMAN | Dolichyl-diphosphooligosaccharide--protein glycosyltransferase subunit STT3B | Q8TCJ2 | 0.95 | 0.95 | 0.72 |
| 2413 | STX12_HUMAN | Syntaxin-12 | Q86Y82 | 1.22 | 0.60 | 1.15 |
| 2414 | STX4_HUMAN | Syntaxin-4 | Q12846 |  | 1.66 | 0.77 |
| 2415 | STX7_HUMAN | Syntaxin-7 | O15400 | 1.27 | 1.25 | 1.05 |
| 2416 | SUCA_HUMAN | Succinyl-CoA ligase [ADP/GDP-forming] subunit alpha, mitochondrial | P53597 | 0.77 | 2.11 | 1.04 |
| 2417 | SUCB1_HUMAN | Succinyl-CoA ligase [ADP-forming] subunit beta, mitochondrial | Q9P2R7 | 1.11 | 0.23 | 0.92 |
| 2418 | SUCB2_HUMAN | Succinyl-CoA ligase [GDP-forming] subunit beta, mitochondrial | Q96I99 | 1.80 | 1.38 | 0.98 |
| 2419 | SUMO2_HUMAN | Small ubiquitin-related modifier 2 | P61956 | 0.61 |  | 0.95 |
| 2420 | SUN1_HUMAN | SUN domain-containing protein 1 | O94901 | 1.25 | 1.41 | 0.83 |
| 2421 | SUN2_HUMAN | SUN domain-containing protein 2 | Q9UH99 | 0.80 | 1.27 | 0.91 |
| 2422 | SUOX_HUMAN | Sulfite oxidase, mitochondrial | P51687 | 1.01 | 0.90 | 1.22 |
| 2423 | SURF1_HUMAN | Surfeit locus protein 1 | Q15526 | 0.53 | 1.19 | 1.13 |
| 2424 | SURF4_HUMAN | Surfeit locus protein 4 | O15260 |  | 0.51 | 1.18 |
| 2425 | SUV3_HUMAN | ATP-dependent RNA helicase SUPV3L1, mitochondrial | Q8IYB8 | 0.86 | 0.20 | 1.03 |
| 2426 | SVIL_HUMAN | Supervillin | O95425 | 0.55 | 1.28 | 0.86 |
| 2427 | SYAC_HUMAN | Alanine--tRNA ligase, cytoplasmic | P49588 | 0.86 | 1.19 | 0.76 |
| 2428 | SYCC_HUMAN | Cysteine--tRNA ligase, cytoplasmic | P49589 | 0.46 | 0.27 | 1.26 |
| 2429 | SYCM_HUMAN | Probable cysteine--tRNA ligase, mitochondrial | Q9HA77 |  | 1.20 | 1.33 |
| 2430 | SYDC_HUMAN | Aspartate--tRNA ligase, cytoplasmic | P14868 | 0.93 | 0.67 |  |
| 2431 | SYDM_HUMAN | Aspartate--tRNA ligase, mitochondrial | Q6PI48 | 1.03 |  | 1.06 |
| 2432 | SYEM_HUMAN | Probable glutamate--tRNA ligase, mitochondrial | Q5JPH6 | 1.96 | 1.47 | 0.97 |
| 2433 | SYEP_HUMAN | Bifunctional glutamate/proline--tRNA ligase | P07814 | 0.55 | 1.04 | 0.95 |
| 2434 | SYFA_HUMAN | Phenylalanine--tRNA ligase alpha subunit | Q9Y285 | 0.72 | 0.97 | 0.99 |
| 2435 | SYFB_HUMAN | Phenylalanine--tRNA ligase beta subunit | Q9NSD9 |  | 0.80 | 1.40 |
| 2436 | SYFM_HUMAN | Phenylalanine--tRNA ligase, mitochondrial | O95363 | 1.02 |  | 0.30 |
| 2437 | SYG_HUMAN | Glycine--tRNA ligase | P41250 | 0.85 | 1.49 |  |
| 2438 | SYHC_HUMAN | Histidine--tRNA ligase, cytoplasmic | P12081 | 1.02 | 0.90 | 0.86 |
| 2439 | SYHM_HUMAN | Probable histidine--tRNA ligase, mitochondrial | P49590 | 1.25 | 0.84 | 1.16 |
| 2440 | SYIC_HUMAN | Isoleucine--tRNA ligase, cytoplasmic | P41252 | 1.10 | 0.53 | 0.92 |
| 2441 | SYIM_HUMAN | Isoleucine--tRNA ligase, mitochondrial | Q9NSE4 | 0.65 | 6.85 | 0.57 |
| 2442 | SYJ2B_HUMAN | Synaptojanin-2-binding protein | P57105 | 1.49 |  | 0.97 |
| 2443 | SYK_HUMAN | Lysine--tRNA ligase | Q15046 | 0.81 | 0.49 | 0.92 |
| 2444 | SYLC_HUMAN | Leucine--tRNA ligase, cytoplasmic | Q9P2J5 | 0.95 |  | 1.18 |
| 2445 | SYLM_HUMAN | Probable leucine--tRNA ligase, mitochondrial | Q15031 |  | 2.15 | 0.77 |
| 2446 | SYMC_HUMAN | Methionine--tRNA ligase, cytoplasmic | P56192 | 1.34 | 1.01 | 0.62 |
| 2447 | SYNC_HUMAN | Asparagine--tRNA ligase, cytoplasmic | O43776 |  |  | 0.88 |
| 2448 | SYNE1_HUMAN | Nesprin-1 | Q8NF91 |  | 2.31 | 0.92 |
| 2449 | SYNE2_HUMAN | Nesprin-2 | Q8WXH0 | 2.03 | 2.05 | 0.75 |
| 2450 | SYNEM_HUMAN | Synemin | O15061 | 0.81 |  | 0.93 |
| 2451 | SYNP2_HUMAN | Synaptopodin-2 | Q9UMS6 | 0.77 | 0.67 | 1.04 |
| 2452 | SYNPO_HUMAN | Synaptopodin | Q8N3V7 | 1.54 |  | 1.08 |
| 2453 | SYP2L_HUMAN | Synaptopodin 2-like protein | Q9H987 |  |  | 1.08 |
| 2454 | SYPL2_HUMAN | Synaptophysin-like protein 2 | Q5VXT5 |  | 1.00 | 1.09 |
| 2455 | SYPM_HUMAN | Probable proline--tRNA ligase, mitochondrial | Q7L3T8 | 0.64 | 0.52 | 1.00 |
| 2456 | SYQ_HUMAN | Glutamine--tRNA ligase | P47897 | 1.67 | 0.61 | 1.00 |
| 2457 | SYRC_HUMAN | Arginine--tRNA ligase, cytoplasmic | P54136 | 1.32 | 1.87 | 0.92 |
| 2458 | SYSC_HUMAN | Serine--tRNA ligase, cytoplasmic | P49591 | 0.31 | 1.03 | 1.17 |
| 2459 | SYSM_HUMAN | Serine--tRNA ligase, mitochondrial | Q9NP81 | 1.07 |  | 0.94 |
| 2460 | SYTC_HUMAN | Threonine--tRNA ligase, cytoplasmic | P26639 | 1.61 | 0.90 | 1.03 |
| 2461 | SYTM_HUMAN | Threonine--tRNA ligase, mitochondrial | Q9BW92 | 0.62 | 1.20 |  |
| 2462 | SYUA_HUMAN | Alpha-synuclein | P37840 | 2.03 |  | 0.82 |
| 2463 | SYVC_HUMAN | Valine--tRNA ligase | P26640 | 0.55 |  | 1.01 |
| 2464 | SYWC_HUMAN | Tryptophan--tRNA ligase, cytoplasmic | P23381 | 0.84 | 0.72 | 1.02 |
| 2465 | SYYC_HUMAN | Tyrosine--tRNA ligase, cytoplasmic | P54577 |  | 1.49 | 0.88 |
| 2466 | SYYM_HUMAN | Tyrosine--tRNA ligase, mitochondrial | Q9Y2Z4 |  | 2.99 | 0.65 |
| 2467 | T126A_HUMAN | Transmembrane protein 126A | Q9H061 | 2.03 | 1.11 |  |
| 2468 | T3HPD_HUMAN | Trans-L-3-hydroxyproline dehydratase | Q96EM0 | 0.88 | 0.99 | 0.73 |
| 2469 | TACC2_HUMAN | Transforming acidic coiled-coil-containing protein 2 | O95359 | 0.89 |  | 0.89 |
| 2470 | TACO1_HUMAN | Translational activator of cytochrome c oxidase 1 | Q9BSH4 | 1.01 | 2.11 | 1.11 |
| 2471 | TADBP_HUMAN | TAR DNA-binding protein 43 | Q13148 | 1.34 | 16.29 | 0.71 |
| 2472 | TAGL_HUMAN | Transgelin | Q01995 | 0.93 |  | 1.01 |
| 2473 | TAGL2_HUMAN | Transgelin-2 | P37802 | 0.72 | 1.92 | 1.06 |
| 2474 | TALDO_HUMAN | Transaldolase | P37837 |  | 0.81 | 0.73 |
| 2475 | TAM41_HUMAN | Mitochondrial translocator assembly and maintenance protein 41 homolog | Q96BW9 |  | 1.19 | 1.47 |
| 2476 | TARA_HUMAN | TRIO and F-actin-binding protein | Q9H2D6 |  | 1.33 | 1.51 |
| 2477 | TARSH_HUMAN | Target of Nesh-SH3 | Q7Z7G0 |  |  | 1.01 |
| 2478 | TATD1_HUMAN | Putative deoxyribonuclease TATDN1 | Q6P1N9 |  | 0.87 | 1.06 |
| 2479 | TAU_HUMAN | Microtubule-associated protein tau | P10636 | 1.16 | 0.85 | 0.83 |
| 2480 | TBA1A_HUMAN | Tubulin alpha-1A chain | Q71U36 | 1.33 |  |  |
| 2481 | TBA1B_HUMAN | Tubulin alpha-1B chain | P68363 | 0.65 |  | 1.49 |
| 2482 | TBA4A_HUMAN | Tubulin alpha-4A chain | P68366 | 1.66 | 1.63 | 0.63 |
| 2483 | TBA8_HUMAN | Tubulin alpha-8 chain | Q9NY65 | 0.63 | 1.02 | 0.79 |
| 2484 | TBB2A_HUMAN | Tubulin beta-2A chain | Q13885 | 0.96 |  | 1.14 |
| 2485 | TBB3_HUMAN | Tubulin beta-3 chain | Q13509 |  | 0.60 | 2.74 |
| 2486 | TBB4B_HUMAN | Tubulin beta-4B chain | P68371 | 0.79 |  | 0.74 |
| 2487 | TBB5_HUMAN | Tubulin beta chain | P07437 | 1.45 | 1.22 | 0.99 |
| 2488 | TBB6_HUMAN | Tubulin beta-6 chain | Q9BUF5 |  | 1.42 | 1.02 |
| 2489 | TBC17_HUMAN | TBC1 domain family member 17 | Q9HA65 | 1.09 |  | 1.17 |
| 2490 | TBC24_HUMAN | TBC1 domain family member 24 | Q9ULP9 |  | 0.42 |  |
| 2491 | TBCA_HUMAN | Tubulin-specific chaperone A | O75347 | 1.10 | 0.52 | 0.88 |
| 2492 | TBCB_HUMAN | Tubulin-folding cofactor B | Q99426 | 1.19 | 0.89 | 1.01 |
| 2493 | TBCD1_HUMAN | TBC1 domain family member 1 | Q86TI0 | 0.73 |  |  |
| 2494 | TBCD4_HUMAN | TBC1 domain family member 4 | O60343 | 0.82 | 2.25 | 0.99 |
| 2495 | TBCE_HUMAN | Tubulin-specific chaperone E | Q15813 | 0.99 |  | 0.80 |
| 2496 | TBCEL_HUMAN | Tubulin-specific chaperone cofactor E-like protein | Q5QJ74 |  | 0.77 | 1.40 |
| 2497 | TBG1_HUMAN | Tubulin gamma-1 chain | P23258 |  | 3.40 | 0.88 |
| 2498 | TBRG4_HUMAN | Protein TBRG4 | Q969Z0 | 1.67 | 0.89 | 1.35 |
| 2499 | TCEA1_HUMAN | Transcription elongation factor A protein 1 | P23193 | 2.86 | 1.66 | 1.39 |
| 2500 | TCHP_HUMAN | Trichoplein keratin filament-binding protein | Q9BT92 | 0.82 |  | 0.84 |
| 2501 | TCP4_HUMAN | Activated RNA polymerase II transcriptional coactivator p15 | P53999 |  | 1.01 | 0.98 |
| 2502 | TCPA_HUMAN | T-complex protein 1 subunit alpha | P17987 | 1.64 |  | 0.90 |
| 2503 | TCPB_HUMAN | T-complex protein 1 subunit beta | P78371 | 1.04 | 1.15 | 1.21 |
| 2504 | TCPD_HUMAN | T-complex protein 1 subunit delta | P50991 | 0.89 |  | 0.90 |
| 2505 | TCPE_HUMAN | T-complex protein 1 subunit epsilon | P48643 |  | 1.15 |  |
| 2506 | TCPG_HUMAN | T-complex protein 1 subunit gamma | P49368 | 1.91 |  | 1.26 |
| 2507 | TCPH_HUMAN | T-complex protein 1 subunit eta | Q99832 | 0.76 | 0.69 |  |
| 2508 | TCPQ_HUMAN | T-complex protein 1 subunit theta | P50990 | 1.71 | 0.55 | 1.06 |
| 2509 | TCPZ_HUMAN | T-complex protein 1 subunit zeta | P40227 | 0.86 | 0.75 |  |
| 2510 | TCTP_HUMAN | Translationally-controlled tumor protein | P13693 | 1.14 | 0.90 | 1.09 |
| 2511 | TEBP_HUMAN | Prostaglandin E synthase 3 | Q15185 | 0.52 | 0.93 | 1.04 |
| 2512 | TECR_HUMAN | Very-long-chain enoyl-CoA reductase | Q9NZ01 |  | 1.57 | 0.72 |
| 2513 | TECRL_HUMAN | Trans-2,3-enoyl-CoA reductase-like | Q5HYJ1 | 0.78 | 1.11 | 1.21 |
| 2514 | TELT_HUMAN | Telethonin | O15273 | 1.27 |  | 1.03 |
| 2515 | TENS1_HUMAN | Tensin-1 | Q9HBL0 | 1.02 | 1.47 | 0.96 |
| 2516 | TENX_HUMAN | Tenascin-X | P22105 |  |  | 0.98 |
| 2517 | TERA_HUMAN | Transitional endoplasmic reticulum ATPase | P55072 | 0.95 |  | 1.04 |
| 2518 | TES_HUMAN | Testin | Q9UGI8 |  |  | 0.35 |
| 2519 | TETN_HUMAN | Tetranectin | P05452 | 1.54 | 1.04 | 0.53 |
| 2520 | TEX10_HUMAN | Testis-expressed sequence 10 protein | Q9NXF1 | 1.00 |  |  |
| 2521 | TFAM_HUMAN | Transcription factor A, mitochondrial | Q00059 |  | 0.87 | 1.03 |
| 2522 | TFG_HUMAN | Protein TFG | Q92734 | 1.45 |  | 1.07 |
| 2523 | TFR1_HUMAN | Transferrin receptor protein 1 | P02786 | 1.13 |  | 0.52 |
| 2524 | TGFI1_HUMAN | Transforming growth factor beta-1-induced transcript 1 protein | O43294 |  | 0.68 | 1.12 |
| 2525 | TGM2_HUMAN | Protein-glutamine gamma-glutamyltransferase 2 | P21980 | 1.27 |  | 1.13 |
| 2526 | THAP4_HUMAN | THAP domain-containing protein 4 | Q8WY91 | 0.52 | 0.57 | 1.79 |
| 2527 | THEM6_HUMAN | Protein THEM6 | Q8WUY1 | 0.99 | 0.52 | 1.01 |
| 2528 | THIK_HUMAN | 3-ketoacyl-CoA thiolase, peroxisomal | P09110 | 0.89 | 1.22 | 2.13 |
| 2529 | THIL_HUMAN | Acetyl-CoA acetyltransferase, mitochondrial | P24752 | 1.34 |  | 1.02 |
| 2530 | THIM_HUMAN | 3-ketoacyl-CoA thiolase, mitochondrial | P42765 | 0.97 |  | 0.89 |
| 2531 | THIO_HUMAN | Thioredoxin | P10599 | 2.38 | 0.44 | 1.09 |
| 2532 | THIOM_HUMAN | Thioredoxin, mitochondrial | Q99757 |  | 2.25 | 1.16 |
| 2533 | THOC4_HUMAN | THO complex subunit 4 | Q86V81 | 1.28 | 0.56 | 0.77 |
| 2534 | THOP1_HUMAN | Thimet oligopeptidase | P52888 | 1.08 | 0.95 | 1.07 |
| 2535 | THRB_HUMAN | Prothrombin | P00734 |  | 0.57 | 0.86 |
| 2536 | THSD4_HUMAN | Thrombospondin type-1 domain-containing protein 4 | Q6ZMP0 |  | 0.52 | 1.20 |
| 2537 | THTM_HUMAN | 3-mercaptopyruvate sulfurtransferase | P25325 | 1.46 |  | 0.84 |
| 2538 | THTR_HUMAN | Thiosulfate sulfurtransferase | Q16762 | 0.81 |  | 0.56 |
| 2539 | THY1_HUMAN | Thy-1 membrane glycoprotein | P04216 | 0.96 | 2.15 | 0.79 |
| 2540 | THYN1_HUMAN | Thymocyte nuclear protein 1 | Q9P016 | 0.96 |  |  |
| 2541 | TIAR_HUMAN | Nucleolysin TIAR | Q01085 |  | 0.26 | 1.31 |
| 2542 | TIDC1_HUMAN | Complex I assembly factor TIMMDC1, mitochondrial | Q9NPL8 | 1.01 | 1.63 | 1.36 |
| 2543 | TIF1B_HUMAN | Transcription intermediary factor 1-beta | Q13263 | 0.47 | 1.39 | 1.26 |
| 2544 | TIGAR_HUMAN | Fructose-2,6-bisphosphatase TIGAR | Q9NQ88 | 1.69 | 0.93 | 1.40 |
| 2545 | TIM10_HUMAN | Mitochondrial import inner membrane translocase subunit Tim10 | P62072 | 1.67 | 0.92 | 0.99 |
| 2546 | TIM13_HUMAN | Mitochondrial import inner membrane translocase subunit Tim13 | Q9Y5L4 | 1.64 | 0.97 | 1.30 |
| 2547 | TIM14_HUMAN | Mitochondrial import inner membrane translocase subunit TIM14 | Q96DA6 | 0.75 | 0.59 | 0.53 |
| 2548 | TIM16_HUMAN | Mitochondrial import inner membrane translocase subunit TIM16 | Q9Y3D7 | 0.12 |  | 0.38 |
| 2549 | TIM21_HUMAN | Mitochondrial import inner membrane translocase subunit Tim21 | Q9BVV7 | 1.06 | 0.95 | 0.58 |
| 2550 | TIM44_HUMAN | Mitochondrial import inner membrane translocase subunit TIM44 | O43615 | 0.92 | 0.93 | 0.94 |
| 2551 | TIM50_HUMAN | Mitochondrial import inner membrane translocase subunit TIM50 | Q3ZCQ8 |  | 2.54 | 0.70 |
| 2552 | TIM8A_HUMAN | Mitochondrial import inner membrane translocase subunit Tim8 A | O60220 | 0.41 | 0.83 | 0.67 |
| 2553 | TIM8B_HUMAN | Mitochondrial import inner membrane translocase subunit Tim8 B | Q9Y5J9 | 1.26 | 0.94 | 0.58 |
| 2554 | TIM9_HUMAN | Mitochondrial import inner membrane translocase subunit Tim9 | Q9Y5J7 |  |  | 0.51 |
| 2555 | TINAL_HUMAN | Tubulointerstitial nephritis antigen-like | Q9GZM7 | 0.68 |  | 0.96 |
| 2556 | TIPRL_HUMAN | TIP41-like protein | O75663 |  | 2.03 | 0.99 |
| 2557 | TITIN_HUMAN | Titin | Q8WZ42 | 1.06 | 1.03 | 0.92 |
| 2558 | TKT_HUMAN | Transketolase | P29401 | 0.76 | 1.14 | 1.13 |
| 2559 | TLN1_HUMAN | Talin-1 | Q9Y490 |  |  | 0.95 |
| 2560 | TLN2_HUMAN | Talin-2 | Q9Y4G6 | 0.73 |  | 0.97 |
| 2561 | TM109_HUMAN | Transmembrane protein 109 | Q9BVC6 | 1.00 | 1.22 | 1.08 |
| 2562 | TM141_HUMAN | Transmembrane protein 141 | Q96I45 |  | 0.35 | 1.74 |
| 2563 | TM143_HUMAN | Transmembrane protein 143 | Q96AN5 | 2.65 | 17.38 | 1.00 |
| 2564 | TM14C_HUMAN | Transmembrane protein 14C | Q9P0S9 | 1.14 | 1.00 | 0.93 |
| 2565 | TM159_HUMAN | Promethin | Q96B96 | 0.99 | 0.97 |  |
| 2566 | TM182_HUMAN | Transmembrane protein 182 | Q6ZP80 | 0.45 |  | 1.17 |
| 2567 | TM1L2_HUMAN | TOM1-like protein 2 | Q6ZVM7 | 0.72 | 1.00 | 1.14 |
| 2568 | TM205_HUMAN | Transmembrane protein 205 | Q6UW68 | 1.33 | 0.69 | 1.08 |
| 2569 | TM245_HUMAN | Transmembrane protein 245 | Q9H330 | 1.14 | 1.80 | 1.06 |
| 2570 | TM256_HUMAN | Transmembrane protein 256 | Q8N2U0 | 2.33 | 1.15 | 0.66 |
| 2571 | TM263_HUMAN | Transmembrane protein 263 | Q8WUH6 | 1.80 | 1.75 | 1.16 |
| 2572 | TM40L_HUMAN | Mitochondrial import receptor subunit TOM40B | Q969M1 |  | 4.37 | 1.02 |
| 2573 | TM9S2_HUMAN | Transmembrane 9 superfamily member 2 | Q99805 |  | 0.80 | 1.87 |
| 2574 | TMED2_HUMAN | Transmembrane emp24 domain-containing protein 2 | Q15363 | 1.11 | 0.43 | 1.30 |
| 2575 | TMED4_HUMAN | Transmembrane emp24 domain-containing protein 4 | Q7Z7H5 |  | 0.88 | 1.15 |
| 2576 | TMED7_HUMAN | Transmembrane emp24 domain-containing protein 7 | Q9Y3B3 | 0.89 | 1.19 | 0.91 |
| 2577 | TMED9_HUMAN | Transmembrane emp24 domain-containing protein 9 | Q9BVK6 | 0.31 | 0.99 | 1.22 |
| 2578 | TMEDA_HUMAN | Transmembrane emp24 domain-containing protein 10 | P49755 |  | 0.54 | 1.06 |
| 2579 | TMLH_HUMAN | Trimethyllysine dioxygenase, mitochondrial | Q9NVH6 |  |  | 0.98 |
| 2580 | TMM11_HUMAN | Transmembrane protein 11, mitochondrial | P17152 |  | 0.64 | 1.23 |
| 2581 | TMM33_HUMAN | Transmembrane protein 33 | P57088 |  | 1.74 |  |
| 2582 | TMM43_HUMAN | Transmembrane protein 43 | Q9BTV4 | 0.89 | 2.31 | 1.15 |
| 2583 | TMM65_HUMAN | Transmembrane protein 65 | Q6PI78 | 1.53 | 4.21 | 0.97 |
| 2584 | TMOD1_HUMAN | Tropomodulin-1 | P28289 | 0.17 | 0.49 | 0.87 |
| 2585 | TMOD2_HUMAN | Tropomodulin-2 | Q9NZR1 | 1.27 |  | 0.81 |
| 2586 | TMOD3_HUMAN | Tropomodulin-3 | Q9NYL9 | 0.22 | 0.82 |  |
| 2587 | TMX2_HUMAN | Thioredoxin-related transmembrane protein 2 | Q9Y320 | 0.90 | 0.74 | 1.52 |
| 2588 | TMX3_HUMAN | Protein disulfide-isomerase TMX3 | Q96JJ7 | 0.26 | 0.82 | 0.91 |
| 2589 | TMX4_HUMAN | Thioredoxin-related transmembrane protein 4 | Q9H1E5 | 0.41 |  | 0.89 |
| 2590 | TNAP2_HUMAN | Tumor necrosis factor alpha-induced protein 2 | Q03169 | 0.94 |  | 0.81 |
| 2591 | TNG2_HUMAN | Transport and Golgi organization protein 2 homolog | Q6ICL3 | 1.27 | 1.32 | 0.48 |
| 2592 | TNNC1_HUMAN | Troponin C, slow skeletal and cardiac muscles | P63316 | 0.36 | 0.95 | 1.14 |
| 2593 | TNNI1_HUMAN | Troponin I, slow skeletal muscle | P19237 | 0.80 | 2.36 | 1.03 |
| 2594 | TNNI3_HUMAN | Troponin I, cardiac muscle | P19429 |  | 0.67 | 1.07 |
| 2595 | TNNT2_HUMAN | Troponin T, cardiac muscle | P45379 | 0.54 | 0.78 | 1.20 |
| 2596 | TNPO1_HUMAN | Transportin-1 | Q92973 | 1.47 | 1.15 | 1.00 |
| 2597 | TOIP1_HUMAN | Torsin-1A-interacting protein 1 | Q5JTV8 | 0.46 | 1.36 | 1.12 |
| 2598 | TOLIP_HUMAN | Toll-interacting protein | Q9H0E2 | 2.58 | 0.92 | 0.81 |
| 2599 | TOM1_HUMAN | Target of Myb protein 1 | O60784 | 1.21 | 0.85 | 1.11 |
| 2600 | TOM20_HUMAN | Mitochondrial import receptor subunit TOM20 homolog | Q15388 | 2.13 | 0.12 | 0.89 |
| 2601 | TOM22_HUMAN | Mitochondrial import receptor subunit TOM22 homolog | Q9NS69 | 0.47 | 0.86 |  |
| 2602 | TOM40_HUMAN | Mitochondrial import receptor subunit TOM40 homolog | O96008 | 1.02 | 0.94 | 0.98 |
| 2603 | TOM6_HUMAN | Mitochondrial import receptor subunit TOM6 homolog | Q96B49 |  | 1.13 | 0.98 |
| 2604 | TOM70_HUMAN | Mitochondrial import receptor subunit TOM70 | O94826 |  | 0.86 | 1.03 |
| 2605 | TOP2B_HUMAN | DNA topoisomerase 2-beta | Q02880 | 2.21 |  | 1.11 |
| 2606 | TPC1_HUMAN | Two pore calcium channel protein 1 | Q9ULQ1 | 0.73 |  | 0.72 |
| 2607 | TPC6B_HUMAN | Trafficking protein particle complex subunit 6B | Q86SZ2 |  | 1.04 | 1.22 |
| 2608 | TPD52_HUMAN | Tumor protein D52 | P55327 | 1.09 | 1.04 | 0.94 |
| 2609 | TPD53_HUMAN | Tumor protein D53 | Q16890 | 0.87 | 1.12 | 1.28 |
| 2610 | TPD54_HUMAN | Tumor protein D54 | O43399 | 1.08 | 0.63 | 0.90 |
| 2611 | TPIS_HUMAN | Triosephosphate isomerase | P60174 | 1.20 | 7.24 | 0.91 |
| 2612 | TPM1_HUMAN | Tropomyosin alpha-1 chain | P09493 | 1.12 |  | 1.32 |
| 2613 | TPM2_HUMAN | Tropomyosin beta chain | P07951 |  | 0.89 | 1.12 |
| 2614 | TPM3_HUMAN | Tropomyosin alpha-3 chain | P06753 | 0.87 | 1.25 | 1.60 |
| 2615 | TPM4_HUMAN | Tropomyosin alpha-4 chain | P67936 |  | 0.80 | 0.96 |
| 2616 | TPP1_HUMAN | Tripeptidyl-peptidase 1 | O14773 | 0.76 | 1.15 | 1.29 |
| 2617 | TPP2_HUMAN | Tripeptidyl-peptidase 2 | P29144 | 0.88 | 0.42 | 0.70 |
| 2618 | TPPC4_HUMAN | Trafficking protein particle complex subunit 4 | Q9Y296 |  | 1.20 | 0.99 |
| 2619 | TPPP_HUMAN | Tubulin polymerization-promoting protein | O94811 | 1.74 | 0.98 | 0.73 |
| 2620 | TPPP3_HUMAN | Tubulin polymerization-promoting protein family member 3 | Q9BW30 | 1.13 | 1.06 | 0.80 |
| 2621 | TPR_HUMAN | Nucleoprotein TPR | P12270 |  | 1.02 | 0.97 |
| 2622 | TPRKB_HUMAN | EKC/KEOPS complex subunit TPRKB | Q9Y3C4 |  | 0.87 | 2.47 |
| 2623 | TR112_HUMAN | Multifunctional methyltransferase subunit TRM112-like protein | Q9UI30 | 3.02 |  | 0.25 |
| 2624 | TR150_HUMAN | Thyroid hormone receptor-associated protein 3 | Q9Y2W1 | 0.41 |  | 0.54 |
| 2625 | TRA2B_HUMAN | Transformer-2 protein homolog beta | P62995 | 0.29 | 1.11 | 0.96 |
| 2626 | TRAP1_HUMAN | Heat shock protein 75 kDa, mitochondrial | Q12931 | 0.64 | 0.93 | 1.12 |
| 2627 | TRDN_HUMAN | Triadin | Q13061 |  | 0.26 | 1.03 |
| 2628 | TRFE_HUMAN | Serotransferrin | P02787 |  |  | 1.01 |
| 2629 | TRI25_HUMAN | E3 ubiquitin/ISG15 ligase TRIM25 | Q14258 |  | 0.58 | 0.97 |
| 2630 | TRI54_HUMAN | Tripartite motif-containing protein 54 | Q9BYV2 | 1.16 | 1.36 | 0.25 |
| 2631 | TRI55_HUMAN | Tripartite motif-containing protein 55 | Q9BYV6 | 0.94 | 1.85 | 1.35 |
| 2632 | TRI63_HUMAN | E3 ubiquitin-protein ligase TRIM63 | Q969Q1 |  | 0.94 |  |
| 2633 | TRXR1_HUMAN | Thioredoxin reductase 1, cytoplasmic | Q16881 | 0.99 | 0.88 | 1.37 |
| 2634 | TRXR2_HUMAN | Thioredoxin reductase 2, mitochondrial | Q9NNW7 |  | 1.11 | 0.89 |
| 2635 | TRY1_HUMAN | Trypsin-1 | P07477 |  | 1.32 | 1.12 |
| 2636 | TRYB1_HUMAN | Tryptase alpha/beta-1 | Q15661 | 1.17 | 1.06 |  |
| 2637 | TS101_HUMAN | Tumor susceptibility gene 101 protein | Q99816 |  | 1.74 | 1.00 |
| 2638 | TSN_HUMAN | Translin | Q15631 | 0.11 | 1.08 | 1.22 |
| 2639 | TSN9_HUMAN | Tetraspanin-9 | O75954 | 1.39 |  | 0.45 |
| 2640 | TSP1_HUMAN | Thrombospondin-1 | P07996 |  | 1.57 | 0.55 |
| 2641 | TSP4_HUMAN | Thrombospondin-4 | P35443 | 0.92 | 0.44 | 1.08 |
| 2642 | TSPOA_HUMAN | Translocator protein | P30536 | 2.15 | 1.03 |  |
| 2643 | TSTD1_HUMAN | Thiosulfate sulfurtransferase/rhodanese-like domain-containing protein 1 | Q8NFU3 |  | 0.92 | 0.77 |
| 2644 | TTC19_HUMAN | Tetratricopeptide repeat protein 19, mitochondrial | Q6DKK2 | 0.87 |  | 0.90 |
| 2645 | TTC37_HUMAN | Tetratricopeptide repeat protein 37 | Q6PGP7 | 1.05 | 0.21 | 0.72 |
| 2646 | TTC38_HUMAN | Tetratricopeptide repeat protein 38 | Q5R3I4 |  | 0.09 |  |
| 2647 | TTHY_HUMAN | Transthyretin | P02766 | 1.00 | 1.98 | 1.20 |
| 2648 | TWF2_HUMAN | Twinfilin-2 | Q6IBS0 | 0.12 | 0.81 | 0.82 |
| 2649 | TX1B3_HUMAN | Tax1-binding protein 3 | O14907 | 0.70 | 3.10 | 0.69 |
| 2650 | TXD12_HUMAN | Thioredoxin domain-containing protein 12 | O95881 | 0.95 | 1.77 | 1.09 |
| 2651 | TXD17_HUMAN | Thioredoxin domain-containing protein 17 | Q9BRA2 | 0.95 | 0.88 | 1.01 |
| 2652 | TXLNB_HUMAN | Beta-taxilin | Q8N3L3 | 0.40 | 0.65 | 1.14 |
| 2653 | TXND5_HUMAN | Thioredoxin domain-containing protein 5 | Q8NBS9 | 0.79 | 0.74 | 1.06 |
| 2654 | TXNL1_HUMAN | Thioredoxin-like protein 1 | O43396 | 0.88 | 1.03 | 0.80 |
| 2655 | TXTP_HUMAN | Tricarboxylate transport protein, mitochondrial | P53007 | 1.10 | 0.70 | 1.14 |
| 2656 | TYPH_HUMAN | Thymidine phosphorylase | P19971 |  | 1.37 | 0.77 |
| 2657 | U2AF1_HUMAN | Splicing factor U2AF 35 kDa subunit | Q01081 |  | 0.97 | 0.99 |
| 2658 | U2AF2_HUMAN | Splicing factor U2AF 65 kDa subunit | P26368 | 1.11 |  | 1.77 |
| 2659 | U520_HUMAN | U5 small nuclear ribonucleoprotein 200 kDa helicase | O75643 | 0.90 | 0.94 | 0.87 |
| 2660 | U5S1_HUMAN | 116 kDa U5 small nuclear ribonucleoprotein component | Q15029 | 1.13 |  | 0.80 |
| 2661 | UB2D1_HUMAN | Ubiquitin-conjugating enzyme E2 D1 | P51668 |  | 0.35 | 1.19 |
| 2662 | UB2D2_HUMAN | Ubiquitin-conjugating enzyme E2 D2 | P62837 | 1.53 |  | 0.89 |
| 2663 | UB2D4_HUMAN | Ubiquitin-conjugating enzyme E2 D4 | Q9Y2X8 | 2.15 |  | 2.26 |
| 2664 | UB2L3_HUMAN | Ubiquitin-conjugating enzyme E2 L3 | P68036 | 0.69 | 0.86 |  |
| 2665 | UB2V1_HUMAN | Ubiquitin-conjugating enzyme E2 variant 1 | Q13404 | 0.46 | 2.83 | 1.11 |
| 2666 | UBA1_HUMAN | Ubiquitin-like modifier-activating enzyme 1 | P22314 | 1.01 | 0.77 | 0.97 |
| 2667 | UBA3_HUMAN | NEDD8-activating enzyme E1 catalytic subunit | Q8TBC4 | 0.95 | 3.73 | 1.09 |
| 2668 | UBA6_HUMAN | Ubiquitin-like modifier-activating enzyme 6 | A0AVT1 |  | 0.86 | 1.27 |
| 2669 | UBAC1_HUMAN | Ubiquitin-associated domain-containing protein 1 | Q9BSL1 | 1.21 | 1.03 | 0.69 |
| 2670 | UBC12_HUMAN | NEDD8-conjugating enzyme Ubc12 | P61081 | 1.56 | 4.70 | 1.22 |
| 2671 | UBC9_HUMAN | SUMO-conjugating enzyme UBC9 | P63279 | 1.20 | 0.82 | 0.82 |
| 2672 | UBE2K_HUMAN | Ubiquitin-conjugating enzyme E2 K | P61086 | 0.58 | 0.93 | 0.84 |
| 2673 | UBE2N_HUMAN | Ubiquitin-conjugating enzyme E2 N | P61088 | 0.80 |  | 0.97 |
| 2674 | UBE3A_HUMAN | Ubiquitin-protein ligase E3A | Q05086 | 0.95 | 0.86 | 2.33 |
| 2675 | UBE3C_HUMAN | Ubiquitin-protein ligase E3C | Q15386 | 0.78 | 1.84 | 0.89 |
| 2676 | UBE4B_HUMAN | Ubiquitin conjugation factor E4 B | O95155 |  | 1.61 | 0.96 |
| 2677 | UBFD1_HUMAN | Ubiquitin domain-containing protein UBFD1 | O14562 |  | 0.62 | 1.06 |
| 2678 | UBP10_HUMAN | Ubiquitin carboxyl-terminal hydrolase 10 | Q14694 | 0.49 |  |  |
| 2679 | UBP13_HUMAN | Ubiquitin carboxyl-terminal hydrolase 13 | Q92995 |  | 0.87 | 1.23 |
| 2680 | UBP14_HUMAN | Ubiquitin carboxyl-terminal hydrolase 14 | P54578 | 0.94 | 0.93 | 0.88 |
| 2681 | UBP15_HUMAN | Ubiquitin carboxyl-terminal hydrolase 15 | Q9Y4E8 | 0.90 | 0.98 | 1.50 |
| 2682 | UBP24_HUMAN | Ubiquitin carboxyl-terminal hydrolase 24 | Q9UPU5 | 1.01 |  | 0.72 |
| 2683 | UBP28_HUMAN | Ubiquitin carboxyl-terminal hydrolase 28 | Q96RU2 | 0.55 |  | 0.84 |
| 2684 | UBP2L_HUMAN | Ubiquitin-associated protein 2-like | Q14157 | 1.09 | 0.64 | 0.81 |
| 2685 | UBP47_HUMAN | Ubiquitin carboxyl-terminal hydrolase 47 | Q96K76 |  | 0.59 | 0.87 |
| 2686 | UBP5_HUMAN | Ubiquitin carboxyl-terminal hydrolase 5 | P45974 | 1.45 |  | 1.31 |
| 2687 | UBP7_HUMAN | Ubiquitin carboxyl-terminal hydrolase 7 | Q93009 | 0.60 |  | 1.01 |
| 2688 | UBQL1_HUMAN | Ubiquilin-1 | Q9UMX0 | 1.43 |  | 0.56 |
| 2689 | UBQL2_HUMAN | Ubiquilin-2 | Q9UHD9 |  | 0.65 | 0.85 |
| 2690 | UBR4_HUMAN | E3 ubiquitin-protein ligase UBR4 | Q5T4S7 | 2.75 | 1.20 | 0.79 |
| 2691 | UBXN1_HUMAN | UBX domain-containing protein 1 | Q04323 | 0.41 | 0.65 | 1.31 |
| 2692 | UBXN4_HUMAN | UBX domain-containing protein 4 | Q92575 | 0.92 | 0.93 |  |
| 2693 | UBXN6_HUMAN | UBX domain-containing protein 6 | Q9BZV1 | 0.55 | 0.87 | 0.81 |
| 2694 | UCHL1_HUMAN | Ubiquitin carboxyl-terminal hydrolase isozyme L1 | P09936 | 0.95 | 0.88 | 0.86 |
| 2695 | UCHL3_HUMAN | Ubiquitin carboxyl-terminal hydrolase isozyme L3 | P15374 | 1.58 | 1.27 | 1.35 |
| 2696 | UCHL5_HUMAN | Ubiquitin carboxyl-terminal hydrolase isozyme L5 | Q9Y5K5 | 1.45 | 0.83 | 0.96 |
| 2697 | UCRI_HUMAN | Cytochrome b-c1 complex subunit Rieske, mitochondrial | P47985 |  | 0.68 | 3.09 |
| 2698 | UFD1_HUMAN | Ubiquitin fusion degradation protein 1 homolog | Q92890 | 0.69 | 0.86 | 0.71 |
| 2699 | UFL1_HUMAN | E3 UFM1-protein ligase 1 | O94874 |  | 1.29 | 1.02 |
| 2700 | UFM1_HUMAN | Ubiquitin-fold modifier 1 | P61960 | 1.05 |  | 0.64 |
| 2701 | UFSP2_HUMAN | Ufm1-specific protease 2 | Q9NUQ7 | 1.49 |  | 0.80 |
| 2702 | UGGG1_HUMAN | UDP-glucose:glycoprotein glucosyltransferase 1 | Q9NYU2 | 0.53 | 1.16 | 1.11 |
| 2703 | UGPA_HUMAN | UTP--glucose-1-phosphate uridylyltransferase | Q16851 | 0.74 | 0.86 | 1.23 |
| 2704 | UK114_HUMAN | Ribonuclease UK114 | P52758 | 1.16 | 0.46 | 1.24 |
| 2705 | ULA1_HUMAN | NEDD8-activating enzyme E1 regulatory subunit | Q13564 | 0.54 | 1.18 | 1.08 |
| 2706 | UN45B_HUMAN | Protein unc-45 homolog B | Q8IWX7 | 0.72 | 0.86 | 1.06 |
| 2707 | UQCC1_HUMAN | Ubiquinol-cytochrome-c reductase complex assembly factor 1 | Q9NVA1 | 1.72 | 1.19 | 0.89 |
| 2708 | UQCC2_HUMAN | Ubiquinol-cytochrome-c reductase complex assembly factor 2 | Q9BRT2 | 1.18 | 0.81 | 1.35 |
| 2709 | URP2_HUMAN | Fermitin family homolog 3 | Q86UX7 |  | 0.87 | 0.67 |
| 2710 | USMG5_HUMAN | Up-regulated during skeletal muscle growth protein 5 | Q96IX5 | 0.63 | 0.77 | 0.98 |
| 2711 | USO1_HUMAN | General vesicular transport factor p115 | O60763 | 1.07 | 0.77 | 0.84 |
| 2712 | USP9X_HUMAN | Probable ubiquitin carboxyl-terminal hydrolase FAF-X | Q93008 | 1.05 |  |  |
| 2713 | UTRO_HUMAN | Utrophin | P46939 | 0.85 | 0.90 | 1.07 |
| 2714 | VA0D1_HUMAN | V-type proton ATPase subunit d 1 | P61421 | 1.60 | 1.16 | 1.03 |
| 2715 | VAMP2_HUMAN | Vesicle-associated membrane protein 2 | P63027 | 0.89 | 2.05 | 0.73 |
| 2716 | VAMP3_HUMAN | Vesicle-associated membrane protein 3 | Q15836 | 0.92 | 0.47 | 1.40 |
| 2717 | VAMP5_HUMAN | Vesicle-associated membrane protein 5 | O95183 |  | 0.65 |  |
| 2718 | VAPA_HUMAN | Vesicle-associated membrane protein-associated protein A | Q9P0L0 | 1.11 | 1.21 | 1.01 |
| 2719 | VAPB_HUMAN | Vesicle-associated membrane protein-associated protein B/C | O95292 | 0.64 | 1.25 | 1.05 |
| 2720 | VASP_HUMAN | Vasodilator-stimulated phosphoprotein | P50552 | 0.87 | 1.24 | 0.92 |
| 2721 | VAT1_HUMAN | Synaptic vesicle membrane protein VAT-1 homolog | Q99536 | 0.90 | 1.32 | 1.08 |
| 2722 | VATA_HUMAN | V-type proton ATPase catalytic subunit A | P38606 | 1.06 |  | 1.02 |
| 2723 | VATB2_HUMAN | V-type proton ATPase subunit B, brain isoform | P21281 |  | 1.03 | 0.96 |
| 2724 | VATE1_HUMAN | V-type proton ATPase subunit E 1 | P36543 |  | 1.14 | 1.23 |
| 2725 | VATF_HUMAN | V-type proton ATPase subunit F | Q16864 | 0.91 | 0.70 | 0.14 |
| 2726 | VATG1_HUMAN | V-type proton ATPase subunit G 1 | O75348 | 0.71 | 0.42 | 1.02 |
| 2727 | VATH_HUMAN | V-type proton ATPase subunit H | Q9UI12 | 0.82 |  | 1.19 |
| 2728 | VDAC1_HUMAN | Voltage-dependent anion-selective channel protein 1 | P21796 | 1.14 |  | 1.01 |
| 2729 | VDAC2_HUMAN | Voltage-dependent anion-selective channel protein 2 | P45880 | 1.14 | 1.12 | 1.00 |
| 2730 | VDAC3_HUMAN | Voltage-dependent anion-selective channel protein 3 | Q9Y277 | 1.24 | 0.90 | 0.96 |
| 2731 | VIGLN_HUMAN | Vigilin | Q00341 | 0.61 | 0.90 | 0.66 |
| 2732 | VIME_HUMAN | Vimentin | P08670 | 1.12 |  | 1.18 |
| 2733 | VINC_HUMAN | Vinculin | P18206 |  |  | 0.93 |
| 2734 | VINEX_HUMAN | Vinexin | O60504 |  | 0.26 | 1.60 |
| 2735 | VISL1_HUMAN | Visinin-like protein 1 | P62760 |  | 1.08 | 1.29 |
| 2736 | VKORL_HUMAN | Vitamin K epoxide reductase complex subunit 1-like protein 1 | Q8N0U8 |  | 0.63 | 0.03 |
| 2737 | VLDLR_HUMAN | Very low-density lipoprotein receptor | P98155 | 1.28 | 0.45 | 1.11 |
| 2738 | VP13A_HUMAN | Vacuolar protein sorting-associated protein 13A | Q96RL7 | 0.67 |  | 0.87 |
| 2739 | VP13C_HUMAN | Vacuolar protein sorting-associated protein 13C | Q709C8 |  | 0.91 | 0.75 |
| 2740 | VP26A_HUMAN | Vacuolar protein sorting-associated protein 26A | O75436 | 0.64 | 0.75 | 1.20 |
| 2741 | VP26B_HUMAN | Vacuolar protein sorting-associated protein 26B | Q4G0F5 | 1.34 | 0.80 | 1.01 |
| 2742 | VP33A_HUMAN | Vacuolar protein sorting-associated protein 33A | Q96AX1 |  | 1.77 | 2.39 |
| 2743 | VP37A_HUMAN | Vacuolar protein sorting-associated protein 37A | Q8NEZ2 | 0.79 |  | 1.79 |
| 2744 | VPP1_HUMAN | V-type proton ATPase 116 kDa subunit a isoform 1 | Q93050 | 0.90 | 1.57 | 0.95 |
| 2745 | VPS25_HUMAN | Vacuolar protein-sorting-associated protein 25 | Q9BRG1 | 0.97 | 0.88 | 1.10 |
| 2746 | VPS28_HUMAN | Vacuolar protein sorting-associated protein 28 homolog | Q9UK41 | 1.28 | 1.03 | 1.27 |
| 2747 | VPS29_HUMAN | Vacuolar protein sorting-associated protein 29 | Q9UBQ0 | 0.35 | 0.63 | 0.87 |
| 2748 | VPS35_HUMAN | Vacuolar protein sorting-associated protein 35 | Q96QK1 |  | 1.42 | 0.98 |
| 2749 | VPS36_HUMAN | Vacuolar protein-sorting-associated protein 36 | Q86VN1 | 0.85 |  | 0.84 |
| 2750 | VPS45_HUMAN | Vacuolar protein sorting-associated protein 45 | Q9NRW7 |  | 0.82 | 1.06 |
| 2751 | VPS4A_HUMAN | Vacuolar protein sorting-associated protein 4A | Q9UN37 |  | 0.86 | 0.94 |
| 2752 | VTA1_HUMAN | Vacuolar protein sorting-associated protein VTA1 homolog | Q9NP79 |  | 0.69 | 0.91 |
| 2753 | VTDB_HUMAN | Vitamin D-binding protein | P02774 | 0.95 | 0.79 | 0.85 |
| 2754 | VTNC_HUMAN | Vitronectin | P04004 | 0.84 | 0.59 | 1.51 |
| 2755 | VWA1_HUMAN | von Willebrand factor A domain-containing protein 1 | Q6PCB0 | 1.03 | 0.52 | 1.18 |
| 2756 | VWA8_HUMAN | von Willebrand factor A domain-containing protein 8 | A3KMH1 |  |  | 1.08 |
| 2757 | VWF_HUMAN | von Willebrand factor | P04275 | 0.55 | 0.82 | 0.89 |
| 2758 | WASF2_HUMAN | Wiskott-Aldrich syndrome protein family member 2 | Q9Y6W5 |  | 2.99 | 0.98 |
| 2759 | WASH7_HUMAN | WASH complex subunit 7 | Q2M389 | 0.90 |  |  |
| 2760 | WBP2_HUMAN | WW domain-binding protein 2 | Q969T9 | 0.95 | 1.77 | 0.71 |
| 2761 | WDFY1_HUMAN | WD repeat and FYVE domain-containing protein 1 | Q8IWB7 | 2.23 | 2.13 | 0.55 |
| 2762 | WDR1_HUMAN | WD repeat-containing protein 1 | O75083 | 1.94 | 0.54 | 1.21 |
| 2763 | WDR13_HUMAN | WD repeat-containing protein 13 | Q9H1Z4 |  | 0.90 | 0.70 |
| 2764 | WDR37_HUMAN | WD repeat-containing protein 37 | Q9Y2I8 | 0.37 | 1.22 | 1.16 |
| 2765 | WDR44_HUMAN | WD repeat-containing protein 44 | Q5JSH3 |  | 1.71 |  |
| 2766 | WDR61_HUMAN | WD repeat-containing protein 61 | Q9GZS3 | 0.75 | 1.32 | 1.07 |
| 2767 | WFS1_HUMAN | Wolframin | O76024 | 0.30 | 1.31 |  |
| 2768 | WIBG_HUMAN | Partner of Y14 and mago | Q9BRP8 |  | 2.15 | 1.14 |
| 2769 | WNK1_HUMAN | Serine/threonine-protein kinase WNK1 | Q9H4A3 |  |  |  |
| 2770 | XIRP1_HUMAN | Xin actin-binding repeat-containing protein 1 | Q702N8 | 0.72 |  | 0.79 |
| 2771 | XIRP2_HUMAN | Xin actin-binding repeat-containing protein 2 | A4UGR9 |  | 2.23 | 0.84 |
| 2772 | XPO1_HUMAN | Exportin-1 | O14980 | 1.07 | 1.24 | 0.84 |
| 2773 | XPO2_HUMAN | Exportin-2 | P55060 | 1.06 | 1.04 | 0.90 |
| 2774 | XPO4_HUMAN | Exportin-4 | Q9C0E2 |  | 0.60 |  |
| 2775 | XPO7_HUMAN | Exportin-7 | Q9UIA9 | 2.36 | 0.28 | 0.77 |
| 2776 | XPP1_HUMAN | Xaa-Pro aminopeptidase 1 | Q9NQW7 | 1.07 |  | 0.55 |
| 2777 | XPP3_HUMAN | Probable Xaa-Pro aminopeptidase 3 | Q9NQH7 | 1.98 | 0.90 | 0.87 |
| 2778 | XRCC5_HUMAN | X-ray repair cross-complementing protein 5 | P13010 | 0.58 | 0.91 | 1.09 |
| 2779 | XRCC6_HUMAN | X-ray repair cross-complementing protein 6 | P12956 | 1.21 |  | 1.08 |
| 2780 | YAP1_HUMAN | Transcriptional coactivator YAP1 | P46937 | 1.21 |  | 0.92 |
| 2781 | YBOX1_HUMAN | Nuclease-sensitive element-binding protein 1 | P67809 | 0.54 | 0.68 | 0.96 |
| 2782 | YBOX3_HUMAN | Y-box-binding protein 3 | P16989 | 0.79 | 0.86 | 0.79 |
| 2783 | YES_HUMAN | Tyrosine-protein kinase Yes | P07947 | 0.56 |  | 0.92 |
| 2784 | YKT6_HUMAN | Synaptobrevin homolog YKT6 | O15498 | 0.62 | 1.45 | 1.64 |
| 2785 | ZA2G_HUMAN | Zinc-alpha-2-glycoprotein | P25311 | 1.56 |  | 1.10 |
| 2786 | ZADH2_HUMAN | Zinc-binding alcohol dehydrogenase domain-containing protein 2 | Q8N4Q0 | 7.59 | 0.64 | 1.07 |
| 2787 | ZNT9_HUMAN | Zinc transporter 9 | Q6PML9 |  | 1.01 | 0.99 |
| 2788 | ZO1_HUMAN | Tight junction protein ZO-1 | Q07157 | 1.45 | 0.16 | 1.15 |
| 2789 | ZO2_HUMAN | Tight junction protein ZO-2 | Q9UDY2 | 1.34 |  |  |
| 2790 | ZYX_HUMAN | Zyxin | Q15942 | 1.41 | 1.64 |  |
| 2791 | ADPPT_HUMAN | L-aminoadipate-semialdehyde dehydrogenase-phosphopantetheinyl transferase | Q9NRN7 | 1.07 | 0.99 |  |
| 2792 | AK1C3_HUMAN | Aldo-keto reductase family 1 member C3 | P42330 | 1.92 | 0.69 |  |
| 2793 | AL5AP_HUMAN | Arachidonate 5-lipoxygenase-activating protein | P20292 | 0.30 | 0.36 |  |
| 2794 | APOO_HUMAN | Apolipoprotein O | Q9BUR5 | 0.96 | 1.00 |  |
| 2795 | ARHG7_HUMAN | Rho guanine nucleotide exchange factor 7 | Q14155 | 1.29 | 0.86 |  |
| 2796 | ARM10_HUMAN | Armadillo repeat-containing protein 10 | Q8N2F6 | 0.90 | 1.32 |  |
| 2797 | CF203_HUMAN | Uncharacterized protein C6orf203 | Q9P0P8 | 0.67 | 0.72 |  |
| 2798 | CF211_HUMAN | UPF0364 protein C6orf211 | Q9H993 | 0.83 | 0.90 |  |
| 2799 | CHCH2_HUMAN | Coiled-coil-helix-coiled-coil-helix domain-containing protein 2, mitochondrial | Q9Y6H1 | 0.98 | 0.82 |  |
| 2800 | CHKB_HUMAN | Choline/ethanolamine kinase | Q9Y259 | 1.39 | 2.31 |  |
| 2801 | CP7B1_HUMAN | 25-hydroxycholesterol 7-alpha-hydroxylase | O75881 | 3.94 | 0.33 |  |
| 2802 | CRIP1_HUMAN | Cysteine-rich protein 1 | P50238 | 0.51 | 1.18 |  |
| 2803 | DAZP1_HUMAN | DAZ-associated protein 1 | Q96EP5 | 1.46 | 0.92 |  |
| 2804 | ESAM_HUMAN | Endothelial cell-selective adhesion molecule | Q96AP7 | 0.63 | 0.51 |  |
| 2805 | FAD1_HUMAN | FAD synthase | Q8NFF5 | 1.13 | 5.92 |  |
| 2806 | FARP1_HUMAN | FERM, RhoGEF and pleckstrin domain-containing protein 1 | Q9Y4F1 | 1.36 | 1.42 |  |
| 2807 | FBLN4_HUMAN | EGF-containing fibulin-like extracellular matrix protein 2 | O95967 | 1.09 | 0.91 |  |
| 2808 | FITM2_HUMAN | Fat storage-inducing transmembrane protein 2 | Q8N6M3 | 0.91 | 0.80 |  |
| 2809 | GBB2_HUMAN | Guanine nucleotide-binding protein G(I)/G(S)/G(T) subunit beta-2 | P62879 | 0.74 | 1.15 |  |
| 2810 | GCSH_HUMAN | Glycine cleavage system H protein, mitochondrial | P23434 | 1.71 | 1.43 |  |
| 2811 | GET4_HUMAN | Golgi to ER traffic protein 4 homolog | Q7L5D6 | 1.60 | 0.46 |  |
| 2812 | GPAT3_HUMAN | Glycerol-3-phosphate acyltransferase 3 | Q53EU6 | 1.25 | 1.02 |  |
| 2813 | H2A2A_HUMAN | Histone H2A type 2-A | Q6FI13 | 0.93 | 1.21 |  |
| 2814 | H2AX_HUMAN | Histone H2AX | P16104 | 1.71 | 0.95 |  |
| 2815 | HECD1_HUMAN | E3 ubiquitin-protein ligase HECTD1 | Q9ULT8 | 0.99 | 1.09 |  |
| 2816 | HECD3_HUMAN | E3 ubiquitin-protein ligase HECTD3 | Q5T447 | 0.84 | 2.27 |  |
| 2817 | HNRH2_HUMAN | Heterogeneous nuclear ribonucleoprotein H2 | P55795 | 0.90 | 1.08 |  |
| 2818 | HNRPF_HUMAN | Heterogeneous nuclear ribonucleoprotein F | P52597 | 0.73 | 0.89 |  |
| 2819 | HSP71_HUMAN | Heat shock 70 kDa protein 1A/1B | P08107 | 1.36 |  |  |
| 2820 | HV305_HUMAN | Ig heavy chain V-III region BRO | P01766 | 0.13 | 0.88 |  |
| 2821 | HV309_HUMAN | Ig heavy chain V-III region NIE | P01770 | 0.86 | 0.97 |  |
| 2822 | IMA3_HUMAN | Importin subunit alpha-3 | O00629 | 0.80 | 1.02 |  |
| 2823 | IPO9_HUMAN | Importin-9 | Q96P70 | 0.89 | 1.16 |  |
| 2824 | K2C3_HUMAN | Keratin, type II cytoskeletal 3 | P12035 | 0.30 | 1.15 |  |
| 2825 | KV117_HUMAN | Ig kappa chain V-I region Scw | P01609 | 2.42 | 0.53 |  |
| 2826 | KV308_HUMAN | Ig kappa chain V-III region CLL | P04207 | 0.91 | 1.80 |  |
| 2827 | KV311_HUMAN | Ig kappa chain V-III region IARC/BL41 | P06311 |  | 1.31 |  |
| 2828 | KV313_HUMAN | Ig kappa chain V-III region HIC | P18136 | 1.33 | 0.35 |  |
| 2829 | LAC2_HUMAN | Ig lambda-2 chain C regions | P0CG05 |  | 0.77 |  |
| 2830 | LGMN_HUMAN | Legumain | Q99538 | 0.62 | 2.36 |  |
| 2831 | LPP60_HUMAN | 60 kDa lysophospholipase | Q86U10 | 1.43 | 0.78 |  |
| 2832 | LV301_HUMAN | Ig lambda chain V-III region SH | P01714 | 1.42 | 1.07 |  |
| 2833 | MET7A_HUMAN | Methyltransferase-like protein 7A | Q9H8H3 | 0.95 | 0.89 |  |
| 2834 | MIF_HUMAN | Macrophage migration inhibitory factor | P14174 | 5.97 | 1.15 |  |
| 2835 | MP2K3_HUMAN | Dual specificity mitogen-activated protein kinase kinase 3 | P46734 | 1.03 | 0.69 |  |
| 2836 | NUBP1_HUMAN | Cytosolic Fe-S cluster assembly factor NUBP1 | P53384 | 0.99 |  |  |
| 2837 | NUCG_HUMAN | Endonuclease G, mitochondrial | Q14249 | 2.00 |  |  |
| 2838 | PDK4_HUMAN | [Pyruvate dehydrogenase (acetyl-transferring)] kinase isozyme 4, mitochondrial | Q16654 | 1.51 | 1.34 |  |
| 2839 | PEX5_HUMAN | Peroxisomal targeting signal 1 receptor | P50542 | 7.45 | 0.81 |  |
| 2840 | PI42C_HUMAN | Phosphatidylinositol 5-phosphate 4-kinase type-2 gamma | Q8TBX8 | 2.15 | 1.27 |  |
| 2841 | PTX3_HUMAN | Pentraxin-related protein PTX3 | P26022 | 0.94 | 0.79 |  |
| 2842 | QIL1_HUMAN | Protein QIL1 | Q5XKP0 | 3.47 | 1.49 |  |
| 2843 | RAB1B_HUMAN | Ras-related protein Rab-1B | Q9H0U4 |  | 1.27 |  |
| 2844 | RBPS2_HUMAN | RNA-binding protein with multiple splicing 2 | Q6ZRY4 | 0.86 | 1.49 |  |
| 2845 | RM45_HUMAN | 39S ribosomal protein L45, mitochondrial | Q9BRJ2 | 4.83 | 0.91 |  |
| 2846 | RM52_HUMAN | 39S ribosomal protein L52, mitochondrial | Q86TS9 | 2.17 | 1.67 |  |
| 2847 | RS17L_HUMAN | 40S ribosomal protein S17-like | P0CW22 |  | 1.05 |  |
| 2848 | RS27A_HUMAN | Ubiquitin-40S ribosomal protein S27a | P62979 | 2.27 | 0.91 |  |
| 2849 | S27A4_HUMAN | Long-chain fatty acid transport protein 4 | Q6P1M0 | 2.56 | 0.85 |  |
| 2850 | SCN5A_HUMAN | Sodium channel protein type 5 subunit alpha | Q14524 | 0.68 | 0.99 |  |
| 2851 | SKIV2_HUMAN | Helicase SKI2W | Q15477 | 2.01 | 1.56 |  |
| 2852 | SPCS2_HUMAN | Signal peptidase complex subunit 2 | Q15005 | 0.95 | 0.90 |  |
| 2853 | SRRM2_HUMAN | Serine/arginine repetitive matrix protein 2 | Q9UQ35 | 1.27 | 0.72 |  |
| 2854 | SSRG_HUMAN | Translocon-associated protein subunit gamma | Q9UNL2 | 0.70 | 0.90 |  |
| 2855 | SUGP2_HUMAN | SURP and G-patch domain-containing protein 2 | Q8IX01 | 1.04 | 1.36 |  |
| 2856 | SUGT1_HUMAN | Suppressor of G2 allele of SKP1 homolog | Q9Y2Z0 | 1.51 | 0.46 |  |
| 2857 | SYPL1_HUMAN | Synaptophysin-like protein 1 | Q16563 | 1.80 | 0.88 |  |
| 2858 | SYWM_HUMAN | Tryptophan--tRNA ligase, mitochondrial | Q9UGM6 | 1.11 | 1.79 |  |
| 2859 | TSNAX_HUMAN | Translin-associated protein X | Q99598 | 0.85 | 1.15 |  |
| 2860 | VATC1_HUMAN | V-type proton ATPase subunit C 1 | P21283 | 1.32 | 0.62 |  |
| 2861 | CB39L_HUMAN | Calcium-binding protein 39-like | Q9H9S4 | 2.21 |  |  |
| 2862 | SAMD3_HUMAN | Sterile alpha motif domain-containing protein 3 | Q8N6K7 | 0.26 |  |  |
| 2863 | DR4L2_HUMAN | Dehydrogenase/reductase SDR family member 4-like 2 | Q6PKH6 | 2.58 |  |  |
| 2864 | 1B35_HUMAN | HLA class I histocompatibility antigen, B-35 alpha chain | P30685 | 1.77 |  |  |
| 2865 | 1B78_HUMAN | HLA class I histocompatibility antigen, B-78 alpha chain | P30498 | 1.45 |  |  |
| 2866 | 1B56_HUMAN | HLA class I histocompatibility antigen, B-56 alpha chain | P30495 | 3.40 |  |  |
| 2867 | 1B53_HUMAN | HLA class I histocompatibility antigen, B-53 alpha chain | P30491 | 0.08 |  |  |
| 2868 | 1B52_HUMAN | HLA class I histocompatibility antigen, B-52 alpha chain | P30490 | 2.15 |  |  |
| 2869 | 1B50_HUMAN | HLA class I histocompatibility antigen, B-50 alpha chain | P30488 | 1.77 |  |  |
| 2870 | 1B49_HUMAN | HLA class I histocompatibility antigen, B-49 alpha chain | P30487 | 4.83 |  |  |
| 2871 | 1B46_HUMAN | HLA class I histocompatibility antigen, B-46 alpha chain | P30484 | 3.16 |  |  |
| 2872 | 1B15_HUMAN | HLA class I histocompatibility antigen, B-15 alpha chain | P30464 | 2.11 |  |  |
| 2873 | 1B57_HUMAN | HLA class I histocompatibility antigen, B-57 alpha chain | P18465 | 5.50 |  |  |
| 2874 | 1B51_HUMAN | HLA class I histocompatibility antigen, B-51 alpha chain | P18464 | 2.65 |  |  |
| 2875 | 1B58_HUMAN | HLA class I histocompatibility antigen, B-58 alpha chain | P10319 | 1.01 |  |  |
| 2876 | HS74L_HUMAN | Heat shock 70 kDa protein 4L | O95757 | 4.25 |  |  |
| 2877 | RMXL2_HUMAN | RNA-binding motif protein, X-linked-like-2 | O75526 | 0.51 |  |  |
| 2878 | LZTS1_HUMAN | Leucine zipper putative tumor suppressor 1 | Q9Y250 | 0.45 |  |  |
| 2879 | RSRP1_HUMAN | Arginine/serine-rich protein 1 | Q9BUV0 | 2.27 |  |  |
| 2880 | KV102_HUMAN | Ig kappa chain V-I region AU | P01594 | 1.57 |  |  |
| 2881 | PROD_HUMAN | Proline dehydrogenase 1, mitochondrial | O43272 | 3.40 |  |  |
| 2882 | CAH7_HUMAN | Carbonic anhydrase 7 | P43166 | 0.63 |  |  |
| 2883 | BTBDI_HUMAN | BTB/POZ domain-containing protein 18 | B2RXH4 | 1.25 |  |  |
| 2884 | OR2K2_HUMAN | Olfactory receptor 2K2 | Q8NGT1 | 2.27 |  |  |
| 2885 | LAR4B_HUMAN | La-related protein 4B | Q92615 |  |  |  |
| 2886 | TGFB2_HUMAN | Transforming growth factor beta-2 | P61812 | 1.85 |  |  |
| 2887 | SETD9_HUMAN | SET domain-containing protein 9 | Q8NE22 | 3.10 |  |  |
| 2888 | OSTC_HUMAN | Oligosaccharyltransferase complex subunitTC | Q9NRP0 | 1.53 |  |  |
| 2889 | SIKE1_HUMAN | Suppressor of IKBKE 1 | Q9BRV8 | 0.52 |  |  |
| 2890 | MACOI_HUMAN | Macoilin | Q8N5G2 | 1.61 |  |  |
| 2891 | HPLN4_HUMAN | Hyaluronan and proteoglycan link protein 4 | Q86UW8 | 1.08 |  |  |
| 2892 | STAT2_HUMAN | Signal transducer and activator of transcription 2 | P52630 | 0.24 |  |  |
| 2893 | RASK_HUMAN | GTPase KRas | P01116 | 1.00 |  |  |
| 2894 | EVI5_HUMAN | Ecotropic viral integration site 5 protein homolog | O60447 | 3.16 |  |  |
| 2895 | RBM4B_HUMAN | RNA-binding protein 4B | Q9BQ04 | 0.95 |  |  |
| 2896 | COE2_HUMAN | Transcription factor COE2 | Q9HAK2 | 1.01 |  |  |
| 2897 | CS068_HUMAN | Uncharacterized protein C19orf68 | Q86XI8 | 0.76 |  |  |
| 2898 | PP6R3_HUMAN | Serine/threonine-protein phosphatase 6 regulatory subunit 3 | Q5H9R7 | 2.99 |  |  |
| 2899 | DDX53_HUMAN | Probable ATP-dependent RNA helicase DDX53 | Q86TM3 | 0.73 |  |  |
| 2900 | KHDR2_HUMAN | KH domain-containing, RNA-binding, signal transduction-associated protein 2 | Q5VWX1 | 1.02 |  |  |
| 2901 | TARB1_HUMAN | Probable methyltransferase TARBP1 | Q13395 | 4.02 |  |  |
| 2902 | ROP1L_HUMAN | Ropporin-1-like protein | Q96C74 | 1.66 |  |  |
| 2903 | SNP25_HUMAN | Synaptosomal-associated protein 25 | P60880 | 5.92 |  |  |
| 2904 | TTL13_HUMAN | Tubulin polyglutamylase TTLL13 | A6NNM8 | 6.19 |  |  |
| 2905 | GDAP2_HUMAN | Ganglioside-induced differentiation-associated protein 2 | Q9NXN4 | 2.13 |  |  |
| 2906 | AVEN_HUMAN | Cell death regulator Aven | Q9NQS1 | 2.31 |  |  |
| 2907 | ADCY6_HUMAN | Adenylate cyclase type 6 | O43306 | 3.10 |  |  |
| 2908 | RRP44_HUMAN | Exosome complex exonuclease RRP44 | Q9Y2L1 | 1.87 |  |  |
| 2909 | TMX1_HUMAN | Thioredoxin-related transmembrane protein 1 | Q9H3N1 | 1.16 |  |  |
| 2910 | AK1C2_HUMAN | Aldo-keto reductase family 1 member C2 | P52895 | 1.04 |  |  |
| 2911 | ERR2_HUMAN | Steroid hormone receptor ERR2 | O95718 | 0.50 |  |  |
| 2912 | PCNA_HUMAN | Proliferating cell nuclear antigen | P12004 | 1.66 |  |  |
| 2913 | ENKUR_HUMAN | Enkurin | Q8TC29 | 1.07 |  |  |
| 2914 | C102A_HUMAN | Coiled-coil domain-containing protein 102A | Q96A19 | 1.16 |  |  |
| 2915 | DAP1_HUMAN | Death-associated protein 1 | P51397 | 20.89 |  |  |
| 2916 | KV101_HUMAN | Ig kappa chain V-I region AG | P01593 | 0.33 |  |  |
| 2917 | MB12A_HUMAN | Multivesicular body subunit 12A | Q96EY5 | 2.33 |  |  |
| 2918 | KIF7_HUMAN | Kinesin-like protein KIF7 | Q2M1P5 | 0.78 |  |  |
| 2919 | JAGN1_HUMAN | Protein jagunal homolog 1 | Q8N5M9 | 0.86 |  |  |
| 2920 | SORT_HUMAN | Sortilin | Q99523 | 0.23 |  |  |
| 2921 | SHIP2_HUMAN | Phosphatidylinositol 3,4,5-trisphosphate 5-phosphatase 2 | O15357 | 0.90 |  |  |
| 2922 | TA2R3_HUMAN | Taste receptor type 2 member 3 | Q9NYW6 | 0.37 |  |  |
| 2923 | PLCC_HUMAN | 1-acyl-sn-glycerol-3-phosphate acyltransferase gamma | Q9NRZ7 | 2.27 |  |  |
| 2924 | SCFD2_HUMAN | Sec1 family domain-containing protein 2 | Q8WU76 | 0.95 |  |  |
| 2925 | FUND1_HUMAN | FUN14 domain-containing protein 1 | Q8IVP5 | 0.39 |  |  |
| 2926 | TBC8B_HUMAN | TBC1 domain family member 8B | Q0IIM8 | 1.71 |  |  |
| 2927 | IFIT2_HUMAN | Interferon-induced protein with tetratricopeptide repeats 2 | P09913 | 0.18 |  |  |
| 2928 | WAC_HUMAN | WW domain-containing adapter protein with coiled-coil | Q9BTA9 | 0.94 |  |  |
| 2929 | COG2_HUMAN | Conserved oligomeric Golgi complex subunit 2 | Q14746 | 0.59 |  |  |
| 2930 | WFDC1_HUMAN | WAP four-disulfide core domain protein 1 | Q9HC57 | 1.42 |  |  |
| 2931 | DOPP1_HUMAN | Dolichyldiphosphatase 1 | Q86YN1 | 3.16 |  |  |
| 2932 | APOM_HUMAN | Apolipoprotein M | O95445 | 1.46 |  |  |
| 2933 | PI42A_HUMAN | Phosphatidylinositol 5-phosphate 4-kinase type-2 alpha | P48426 | 1.24 |  |  |
| 2934 | ATG13_HUMAN | Autophagy-related protein 13 | O75143 | 1.02 |  |  |
| 2935 | SNX8_HUMAN | Sorting nexin-8 | Q9Y5X2 | 0.31 |  |  |
| 2936 | RN114_HUMAN | E3 ubiquitin-protein ligase RNF114 | Q9Y508 | 0.18 |  |  |
| 2937 | FA21C_HUMAN | WASH complex subunit FAM21C | Q9Y4E1 | 0.62 |  |  |
| 2938 | AR2BP_HUMAN | ADP-ribosylation factor-like protein 2-binding protein | Q9Y2Y0 | 2.44 |  |  |
| 2939 | PPM1H_HUMAN | Protein phosphatase 1H | Q9ULR3 | 2.29 |  |  |
| 2940 | FBX3_HUMAN | F-box only protein 3 | Q9UK99 | 0.09 |  |  |
| 2941 | UBIP1_HUMAN | Upstream-binding protein 1 | Q9NZI7 | 0.64 |  |  |
| 2942 | TREM2_HUMAN | Triggering receptor expressed on myeloid cells 2 | Q9NZC2 | 0.73 |  |  |
| 2943 | SDHF3_HUMAN | Succinate dehydrogenase assembly factor 3, mitochondrial | Q9NRP4 | 0.24 |  |  |
| 2944 | PXMP2_HUMAN | Peroxisomal membrane protein 2 | Q9NR77 | 0.89 |  |  |
| 2945 | STAR7_HUMAN | StAR-related lipid transfer protein 7, mitochondrial | Q9NQZ5 | 0.62 |  |  |
| 2946 | RAB17_HUMAN | Ras-related protein Rab-17 | Q9H0T7 | 0.71 |  |  |
| 2947 | SRCN1_HUMAN | SRC kinase signaling inhibitor 1 | Q9C0H9 | 0.50 |  |  |
| 2948 | ZY11B_HUMAN | Protein zyg-11 homolog B | Q9C0D3 | 2.33 |  |  |
| 2949 | RPAP1_HUMAN | RNA polymerase II-associated protein 1 | Q9BWH6 | 0.64 |  |  |
| 2950 | DIRC2_HUMAN | Disrupted in renal carcinoma protein 2 | Q96SL1 | 0.66 |  |  |
| 2951 | B3GT6_HUMAN | Beta-1,3-galactosyltransferase 6 | Q96L58 | 0.76 |  |  |
| 2952 | CNDP1_HUMAN | Beta-Ala-His dipeptidase | Q96KN2 | 0.89 |  |  |
| 2953 | CLP1L_HUMAN | Cleft lip and palate transmembrane protein 1-like protein | Q96KA5 | 0.12 |  |  |
| 2954 | AN32B_HUMAN | Acidic leucine-rich nuclear phosphoprotein 32 family member B | Q92688 | 0.41 |  |  |
| 2955 | IL17D_HUMAN | Interleukin-17D | Q8TAD2 | 1.18 |  |  |
| 2956 | PLBL2_HUMAN | Putative phospholipase B-like 2 | Q8NHP8 | 0.90 |  |  |
| 2957 | NBEA_HUMAN | Neurobeachin | Q8NFP9 | 2.03 |  |  |
| 2958 | PCYXL_HUMAN | Prenylcysteine oxidase-like | Q8NBM8 | 5.25 |  |  |
| 2959 | PELP1_HUMAN | Proline-, glutamic acid- and leucine-rich protein 1 | Q8IZL8 | 2.01 |  |  |
| 2960 | K2013_HUMAN | Uncharacterized protein KIAA2013 | Q8IYS2 | 1.80 |  |  |
| 2961 | CPNE8_HUMAN | Copine-8 | Q86YQ8 | 1.41 |  |  |
| 2962 | COMD6_HUMAN | COMM domain-containing protein 6 | Q7Z4G1 | 1.09 |  |  |
| 2963 | PGAP1_HUMAN | GPI inositol-deacylase | Q75T13 | 1.60 |  |  |
| 2964 | UQCC3_HUMAN | Ubiquinol-cytochrome-c reductase complex assembly factor 3 | Q6UW78 | 2.78 |  |  |
| 2965 | IPP2M_HUMAN | Protein phosphatase inhibitor 2-like protein 3 | Q6NXS1 | 1.05 |  |  |
| 2966 | FOCAD_HUMAN | Focadhesin | Q5VW36 | 1.96 |  |  |
| 2967 | ZFP69_HUMAN | Zinc finger protein ZFP69 | Q49AA0 | 1.37 |  |  |
| 2968 | SCN9A_HUMAN | Sodium channel protein type 9 subunit alpha | Q15858 | 2.75 |  |  |
| 2969 | TOM34_HUMAN | Mitochondrial import receptor subunit TOM34 | Q15785 | 1.85 |  |  |
| 2970 | IFIT5_HUMAN | Interferon-induced protein with tetratricopeptide repeats 5 | Q13325 | 0.18 |  |  |
| 2971 | AT2C1_HUMAN | Calcium-transporting ATPase type 2C member 1 | P98194 | 0.14 |  |  |
| 2972 | CLD10_HUMAN | Claudin-10 | P78369 | 0.25 |  |  |
| 2973 | H31_HUMAN | Histone H3.1 | P68431 | 9.64 |  |  |
| 2974 | HDAC4_HUMAN | Histone deacetylase 4 | P56524 | 2.86 |  |  |
| 2975 | NUP98_HUMAN | Nuclear pore complex protein Nup98-Nup96 | P52948 | 0.64 |  |  |
| 2976 | IGLL1_HUMAN | Immunoglobulin lambda-like polypeptide 1 | P15814 |  |  |  |
| 2977 | VAV_HUMAN | Proto-oncogene vav | P15498 | 0.14 |  |  |
| 2978 | MMP9_HUMAN | Matrix metalloproteinase-9 | P14780 | 1.71 |  |  |
| 2979 | KV111_HUMAN | Ig kappa chain V-I region Ka | P01603 | 0.88 |  |  |
| 2980 | FRYL_HUMAN | Protein furry homolog-like | O94915 | 1.03 |  |  |
| 2981 | LTN1_HUMAN | E3 ubiquitin-protein ligase listerin | O94822 | 1.11 |  |  |
| 2982 | RNH2A_HUMAN | Ribonuclease H2 subunit A | O75792 | 0.90 |  |  |
| 2983 | KBL_HUMAN | 2-amino-3-ketobutyrate coenzyme A ligase, mitochondrial | O75600 | 0.68 |  |  |
| 2984 | TRIM3_HUMAN | Tripartite motif-containing protein 3 | O75382 | 2.11 |  |  |
| 2985 | TI17B_HUMAN | Mitochondrial import inner membrane translocase subunit Tim17-B | O60830 | 0.95 |  |  |
| 2986 | LY75_HUMAN | Lymphocyte antigen 75 | O60449 | 0.72 |  |  |
| 2987 | VIP2_HUMAN | Inositol hexakisphosphate and diphosphoinositol-pentakisphosphate kinase 2 | O43314 | 0.40 |  |  |
| 2988 | MCES_HUMAN | mRNA cap guanine-N7 methyltransferase | O43148 |  |  |  |
| 2989 | GOSR2_HUMAN | Golgi SNAP receptor complex member 2 | O14653 | 2.03 |  |  |
| 2990 | AN36A_HUMAN | Ankyrin repeat domain-containing protein 36A | A6QL64 | 0.22 |  |  |
| 2991 | BIG2_HUMAN | Brefeldin A-inhibited guanine nucleotide-exchange protein 2 | Q9Y6D5 | 1.72 |  |  |
| 2992 | WRP73_HUMAN | WD repeat-containing protein WRAP73 | Q9P2S5 | 0.83 |  |  |
| 2993 | ANKZ1_HUMAN | Ankyrin repeat and zinc finger domain-containing protein 1 | Q9H8Y5 | 1.43 |  |  |
| 2994 | DDX24_HUMAN | ATP-dependent RNA helicase DDX24 | Q9GZR7 | 0.54 |  |  |
| 2995 | SELO_HUMAN | Selenoprotein O | Q9BVL4 | 0.64 |  |  |
| 2996 | PKHO2_HUMAN | Pleckstrin homology domain-containing family O member 2 | Q8TD55 | 0.42 |  |  |
| 2997 | WDR48_HUMAN | WD repeat-containing protein 48 | Q8TAF3 | 0.41 |  |  |
| 2998 | MROH1_HUMAN | Maestro heat-like repeat-containing protein family member 1 | Q8NDA8 | 0.95 |  |  |
| 2999 | GHDC_HUMAN | GH3 domain-containing protein | Q8N2G8 | 1.33 |  |  |
| 3000 | H2B3B_HUMAN | Histone H2B type 3-B | Q8N257 | 0.79 |  |  |
| 3001 | KLH34_HUMAN | Kelch-like protein 34 | Q8N239 | 1.21 |  |  |
| 3002 | RAVR1_HUMAN | Ribonucleoprotein PTB-binding 1 | Q8IY67 | 0.83 |  |  |
| 3003 | TMC8_HUMAN | Transmembrane channel-like protein 8 | Q8IU68 | 0.89 |  |  |
| 3004 | TPC11_HUMAN | Trafficking protein particle complex subunit 11 | Q7Z392 | 0.90 |  |  |
| 3005 | MICA3_HUMAN | Protein-methionine sulfoxide oxidase MICAL3 | Q7RTP6 | 0.17 |  |  |
| 3006 | DDX46_HUMAN | Probable ATP-dependent RNA helicase DDX46 | Q7L014 |  |  |  |
| 3007 | CDC73_HUMAN | Parafibromin | Q6P1J9 | 0.15 |  |  |
| 3008 | IQEC1_HUMAN | IQ motif and SEC7 domain-containing protein 1 | Q6DN90 | 0.42 |  |  |
| 3009 | SMAG2_HUMAN | Protein Smaug homolog 2 | Q5PRF9 | 0.70 |  |  |
| 3010 | BMS1_HUMAN | Ribosome biogenesis protein BMS1 homolog | Q14692 | 0.92 |  |  |
| 3011 | 2A5G_HUMAN | Serine/threonine-protein phosphatase 2A 56 kDa regulatory subunit gamma isoform | Q13362 | 0.82 |  |  |
| 3012 | SOS1_HUMAN | Son of sevenless homolog 1 | Q07889 | 0.52 |  |  |
| 3013 | GFPT1_HUMAN | Glutamine--fructose-6-phosphate aminotransferase [isomerizing] 1 | Q06210 | 1.03 |  |  |
| 3014 | LAP2A_HUMAN | Lamina-associated polypeptide 2, isoform alpha | P42166 | 0.92 |  |  |
| 3015 | PSB10_HUMAN | Proteasome subunit beta type-10 | P40306 | 1.36 |  |  |
| 3016 | SMAD9_HUMAN | Mothers against decapentaplegic homolog 9 | O15198 | 0.56 |  |  |
| 3017 | PUR4_HUMAN | Phosphoribosylformylglycinamidine synthase | O15067 | 0.95 |  |  |
| 3018 | HNRC2_HUMAN | Heterogeneous nuclear ribonucleoprotein C-like 2 | B2RXH8 | 0.74 |  |  |
| 3019 | BIG1_HUMAN | Brefeldin A-inhibited guanine nucleotide-exchange protein 1 | Q9Y6D6 | 0.37 |  |  |
| 3020 | RBM15_HUMAN | Putative RNA-binding protein 15 | Q96T37 | 1.77 |  |  |
| 3021 | RBFA_HUMAN | Putative ribosome-binding factor A, mitochondrial | Q8N0V3 | 0.99 |  |  |
| 3022 | EMAL6_HUMAN | Echinoderm microtubule-associated protein-like 6 | Q6ZMW3 | 0.49 |  |  |
| 3023 | ULK3_HUMAN | Serine/threonine-protein kinase ULK3 | Q6PHR2 | 0.54 |  |  |
| 3024 | LAR1B_HUMAN | La-related protein 1B | Q659C4 | 1.14 |  |  |
| 3025 | CLN3_HUMAN | Battenin | Q13286 | 0.77 |  |  |
| 3026 | KIF1A_HUMAN | Kinesin-like protein KIF1A | Q12756 | 0.14 |  |  |
| 3027 | MCM7_HUMAN | DNA replication licensing factor MCM7 | P33993 | 2.63 |  |  |
| 3028 | SMCA1_HUMAN | Probable global transcription activator SNF2L1 | P28370 | 0.21 |  |  |
| 3029 | PAX3_HUMAN | Paired box protein Pax-3 | P23760 | 1.46 |  |  |
| 3030 | KCRU_HUMAN | Creatine kinase U-type, mitochondrial | P12532 | 1.24 |  |  |
| 3031 | CP1A1_HUMAN | Cytochrome P450 1A1 | P04798 | 0.86 |  |  |
| 3032 | LARGE_HUMAN | Glycosyltransferase-like protein LARGE1 | O95461 | 1.91 |  |  |
| 3033 | STRP2_HUMAN | Striatin-interacting protein 2 | Q9ULQ0 | 0.99 |  |  |
| 3034 | GBRL1_HUMAN | Gamma-aminobutyric acid receptor-associated protein-like 1 | Q9H0R8 | 2.83 |  |  |
| 3035 | FTO_HUMAN | Alpha-ketoglutarate-dependent dioxygenase FTO | Q9C0B1 | 0.63 |  |  |
| 3036 | DEPD7_HUMAN | DEP domain-containing protein 7 | Q96QD5 | 0.83 |  |  |
| 3037 | PLIN5_HUMAN | Perilipin-5 | Q00G26 | 1.01 |  |  |
| 3038 | CXXC1_HUMAN | CXXC-type zinc finger protein 1 | Q9P0U4 | 0.90 |  |  |
| 3039 | EPN3_HUMAN | Epsin-3 | Q9H201 | 1.20 |  |  |
| 3040 | MIO_HUMAN | WD repeat-containing protein mio | Q9NXC5 | 1.56 |  |  |
| 3041 | GRDN_HUMAN | Girdin | Q3V6T2 | 1.06 |  |  |
| 3042 | DIAP1_HUMAN | Protein diaphanous homolog 1 | O60610 | 1.60 |  |  |
| 3043 | GTPC1_HUMAN | Putative GTP cyclohydrolase 1 type 2 NIF3L1 | Q9GZT8 | 0.59 |  |  |
| 3044 | OXND1_HUMAN | Oxidoreductase NAD-binding domain-containing protein 1 | Q96HP4 | 0.90 |  |  |
| 3045 | PLCB_HUMAN | 1-acyl-sn-glycerol-3-phosphate acyltransferase beta | O15120 | 2.05 |  |  |
| 3046 | KHNYN_HUMAN | Protein KHNYN | O15037 | 0.52 |  |  |
| 3047 | AHI1_HUMAN | Jouberin | Q8N157 | 0.76 |  |  |
| 3048 | RUXGL_HUMAN | Putative small nuclear ribonucleoprotein G-like protein 15 | A8MWD9 | 1.63 |  |  |
| 3049 | GDN_HUMAN | Glia-derived nexin | P07093 | 0.90 |  |  |
| 3050 | CNOT1_HUMAN | CCR4-NOT transcription complex subunit 1 | A5YKK6 | 0.72 |  |  |
| 3051 | TENA_HUMAN | Tenascin | P24821 | 0.56 |  |  |
| 3052 | NUPL1_HUMAN | Nucleoporin p58/p45 | Q9BVL2 | 1.60 |  |  |
| 3053 | PREY_HUMAN | Protein preY, mitochondrial | Q96I23 | 0.76 |  |  |
| 3054 | KCMF1_HUMAN | E3 ubiquitin-protein ligase KCMF1 | Q9P0J7 | 0.95 |  |  |
| 3055 | WDR11_HUMAN | WD repeat-containing protein 11 | Q9BZH6 | 2.31 |  |  |
| 3056 | GOGA2_HUMAN | Golgin subfamily A member 2 | Q08379 | 0.90 |  |  |
| 3057 | DPOG1_HUMAN | DNA polymerase subunit gamma-1 | P54098 | 1.34 |  |  |
| 3058 | RRAGD_HUMAN | Ras-related GTP-binding protein D | Q9NQL2 | 1.06 |  |  |
| 3059 | KV204_HUMAN | Ig kappa chain V-II region TEW | P01617 | 1.54 |  |  |
| 3060 | UBR3_HUMAN | E3 ubiquitin-protein ligase UBR3 | Q6ZT12 | 0.42 |  |  |
| 3061 | RL26L_HUMAN | 60S ribosomal protein L26-like 1 | Q9UNX3 |  |  |  |
| 3062 | FRY_HUMAN | Protein furry homolog | Q5TBA9 |  |  |  |
| 3063 | PRPS2_HUMAN | Ribose-phosphate pyrophosphokinase 2 | P11908 | 0.86 |  |  |
| 3064 | FAH2B_HUMAN | Fumarylacetoacetate hydrolase domain-containing protein 2B | Q6P2I3 | 1.16 |  |  |
| 3065 | NOMO1_HUMAN | Nodal modulator 1 | Q15155 | 0.89 |  |  |
| 3066 | 1A33_HUMAN | HLA class I histocompatibility antigen, A-33 alpha chain | P16190 |  |  |  |
| 3067 | H2B1N_HUMAN | Histone H2B type 1-N | Q99877 |  |  |  |
| 3068 | WDR62_HUMAN | WD repeat-containing protein 62 | O43379 |  | 1.46 |  |
| 3069 | 2B1F_HUMAN | HLA class II histocompatibility antigen, DRB1-15 beta chain | P01911 |  | 0.86 |  |
| 3070 | KV123_HUMAN | Ig kappa chain V-I region Walker | P04431 |  | 1.61 |  |
| 3071 | KV124_HUMAN | Ig kappa chain V-I region Daudi | P04432 |  | 0.84 |  |
| 3072 | GFAP_HUMAN | Glial fibrillary acidic protein | P14136 |  | 0.76 |  |
| 3073 | KRT81_HUMAN | Keratin, type II cuticular Hb1 | Q14533 |  | 0.93 |  |
| 3074 | RHG40_HUMAN | Rho GTPase-activating protein 40 | Q5TG30 |  | 1.51 |  |
| 3075 | K2C78_HUMAN | Keratin, type II cytoskeletal 78 | Q8N1N4 |  | 1.03 |  |
| 3076 | KLH32_HUMAN | Kelch-like protein 32 | Q96NJ5 |  | 1.57 |  |
| 3077 | RPR1A_HUMAN | Regulation of nuclear pre-mRNA domain-containing protein 1A | Q96P16 |  | 0.93 |  |
| 3078 | FHR5_HUMAN | Complement factor H-related protein 5 | Q9BXR6 |  | 0.23 |  |
| 3079 | K2C75_HUMAN | Keratin, type II cytoskeletal 75 | O95678 |  | 0.59 |  |
| 3080 | KV118_HUMAN | Ig kappa chain V-I region WEA | P01610 |  | 1.50 |  |
| 3081 | K2C79_HUMAN | Keratin, type II cytoskeletal 79 | Q5XKE5 |  | 0.82 |  |
| 3082 | ARL8A_HUMAN | ADP-ribosylation factor-like protein 8A | Q96BM9 |  | 2.75 |  |
| 3083 | RIC8B_HUMAN | Synembryn-B | Q9NVN3 |  | 1.07 |  |
| 3084 | K2C7_HUMAN | Keratin, type II cytoskeletal 7 | P08729 |  |  |  |
| 3085 | ARI1A_HUMAN | AT-rich interactive domain-containing protein 1A | O14497 |  | 0.59 |  |
| 3086 | KRT85_HUMAN | Keratin, type II cuticular Hb5 | P78386 |  | 2.17 |  |
| 3087 | VPS18_HUMAN | Vacuolar protein sorting-associated protein 18 homolog | Q9P253 |  | 0.37 |  |
| 3088 | CAH3_HUMAN | Carbonic anhydrase 3 | P07451 |  | 0.68 |  |
| 3089 | R39L5_HUMAN | Putative 60S ribosomal protein L39-like 5 | Q59GN2 |  | 0.69 |  |
| 3090 | KBTB3_HUMAN | Kelch repeat and BTB domain-containing protein 3 | Q8NAB2 |  | 1.05 |  |
| 3091 | CA198_HUMAN | Uncharacterized protein C1orf198 | Q9H425 |  | 2.00 |  |
| 3092 | HV306_HUMAN | Ig heavy chain V-III region BUT | P01767 |  | 0.58 |  |
| 3093 | KI3X1_HUMAN | Putative killer cell immunoglobulin-like receptor-like protein KIR3DX1 | Q9H7L2 |  | 1.43 |  |
| 3094 | NOX5_HUMAN | NADPH oxidase 5 | Q96PH1 |  | 7.38 |  |
| 3095 | DCNL5_HUMAN | DCN1-like protein 5 | Q9BTE7 |  | 1.08 |  |
| 3096 | ERIC5_HUMAN | Glutamate-rich protein 5 | Q6P6B1 |  | 1.54 |  |
| 3097 | PHLB2_HUMAN | Pleckstrin homology-like domain family B member 2 | Q86SQ0 |  | 1.17 |  |
| 3098 | ANR40_HUMAN | Ankyrin repeat domain-containing protein 40 | Q6AI12 |  | 1.12 |  |
| 3099 | PCM1_HUMAN | Pericentriolar material 1 protein | Q15154 |  | 0.64 |  |
| 3100 | PARM1_HUMAN | Prostate androgen-regulated mucin-like protein 1 | Q6UWI2 |  | 0.80 |  |
| 3101 | U17L1_HUMAN | Putative ubiquitin carboxyl-terminal hydrolase 17-like protein 1 | Q7RTZ2 |  | 0.95 |  |
| 3102 | K1C19_HUMAN | Keratin, type I cytoskeletal 19 | P08727 |  | 1.01 |  |
| 3103 | HS3S1_HUMAN | Heparan sulfate glucosamine 3-O-sulfotransferase 1 | O14792 |  | 0.90 |  |
| 3104 | RBBP9_HUMAN | Putative hydrolase RBBP9 | O75884 |  | 1.02 |  |
| 3105 | K2C80_HUMAN | Keratin, type II cytoskeletal 80 | Q6KB66 |  | 0.37 |  |
| 3106 | INADL_HUMAN | InaD-like protein | Q8NI35 |  | 1.91 |  |
| 3107 | FA96A_HUMAN | MIP18 family protein FAM96A | Q9H5X1 |  | 0.64 |  |
| 3108 | DUS4L_HUMAN | tRNA-dihydrouridine(20a/20b) synthase [NAD(P)+]-like | O95620 |  | 1.75 |  |
| 3109 | DAF_HUMAN | Complement decay-accelerating factor | P08174 |  | 1.50 |  |
| 3110 | LPP1_HUMAN | Lipid phosphate phosphohydrolase 1 | O14494 |  | 0.57 |  |
| 3111 | SENP8_HUMAN | Sentrin-specific protease 8 | Q96LD8 |  | 1.79 |  |
| 3112 | INP4A_HUMAN | Type I inositol 3,4-bisphosphate 4-phosphatase | Q96PE3 |  | 2.70 |  |
| 3113 | ZCHC4_HUMAN | Zinc finger CCHC domain-containing protein 4 | Q9H5U6 |  | 0.91 |  |
| 3114 | MRS2_HUMAN | Magnesium transporter MRS2 homolog, mitochondrial | Q9HD23 |  | 1.10 |  |
| 3115 | HECW2_HUMAN | E3 ubiquitin-protein ligase HECW2 | Q9P2P5 |  | 1.41 |  |
| 3116 | STX2_HUMAN | Syntaxin-2 | P32856 |  | 4.33 |  |
| 3117 | PGCA_HUMAN | Aggrecan core protein | P16112 |  | 1.67 |  |
| 3118 | DVL3_HUMAN | Segment polarity protein dishevelled homolog DVL-3 | Q92997 |  | 0.92 |  |
| 3119 | NR1D2_HUMAN | Nuclear receptor subfamily 1 group D member 2 | Q14995 |  | 2.09 |  |
| 3120 | ABCD2_HUMAN | ATP-binding cassette sub-family D member 2 | Q9UBJ2 |  | 1.80 |  |
| 3121 | WDR66_HUMAN | WD repeat-containing protein 66 | Q8TBY9 |  | 1.21 |  |
| 3122 | RAB3B_HUMAN | Ras-related protein Rab-3B | P20337 |  | 0.70 |  |
| 3123 | RM53_HUMAN | 39S ribosomal protein L53, mitochondrial | Q96EL3 |  | 0.96 |  |
| 3124 | MGAT1_HUMAN | Alpha-1,3-mannosyl-glycoprotein 2-beta-N-acetylglucosaminyltransferase | P26572 |  | 0.53 |  |
| 3125 | RHG10_HUMAN | Rho GTPase-activating protein 10 | A1A4S6 |  | 6.98 |  |
| 3126 | PP2AB_HUMAN | Serine/threonine-protein phosphatase 2A catalytic subunit beta isoform | P62714 |  | 0.70 |  |
| 3127 | S61A2_HUMAN | Protein transport protein Sec61 subunit alpha isoform 2 | Q9H9S3 |  | 1.08 |  |
| 3128 | MOCS3_HUMAN | Adenylyltransferase and sulfurtransferase MOCS3 | O95396 |  | 0.31 |  |
| 3129 | BROX_HUMAN | BRO1 domain-containing protein BROX | Q5VW32 |  | 2.54 |  |
| 3130 | CR025_HUMAN | Uncharacterized protein C18orf25 | Q96B23 |  | 0.56 |  |
| 3131 | DGKA_HUMAN | Diacylglycerol kinase alpha | P23743 |  | 1.41 |  |
| 3132 | MLP3B_HUMAN | Microtubule-associated proteins 1A/1B light chain 3B | Q9GZQ8 |  | 3.73 |  |
| 3133 | MOS1_HUMAN | Mitochondrial inner membrane organizing system protein 1 | Q5TGZ0 |  | 0.87 |  |
| 3134 | CCNY_HUMAN | Cyclin-Y | Q8ND76 |  | 0.37 |  |
| 3135 | GFOD1_HUMAN | Glucose-fructose oxidoreductase domain-containing protein 1 | Q9NXC2 |  | 0.82 |  |
| 3136 | MNDA_HUMAN | Myeloid cell nuclear differentiation antigen | P41218 |  | 3.63 |  |
| 3137 | VPS51_HUMAN | Vacuolar protein sorting-associated protein 51 homolog | Q9UID3 |  | 0.82 |  |
| 3138 | MFAP2_HUMAN | Microfibrillar-associated protein 2 | P55001 |  | 0.37 |  |
| 3139 | ERGI3_HUMAN | Endoplasmic reticulum-Golgi intermediate compartment protein 3 | Q9Y282 |  | 1.39 |  |
| 3140 | SMAD1_HUMAN | Mothers against decapentaplegic homolog 1 | Q15797 |  | 1.18 |  |
| 3141 | CY24B_HUMAN | Cytochrome b-245 heavy chain | P04839 |  | 1.25 |  |
| 3142 | ITB5_HUMAN | Integrin beta-5 | P18084 |  | 1.16 |  |
| 3143 | 3HAO_HUMAN | 3-hydroxyanthranilate 3,4-dioxygenase | P46952 |  | 1.12 |  |
| 3144 | FBX7_HUMAN | F-box only protein 7 | Q9Y3I1 |  | 1.14 |  |
| 3145 | CAPS2_HUMAN | Calcium-dependent secretion activator 2 | Q86UW7 |  | 2.61 |  |
| 3146 | UBL7_HUMAN | Ubiquitin-like protein 7 | Q96S82 |  | 0.95 |  |
| 3147 | PP4C_HUMAN | Serine/threonine-protein phosphatase 4 catalytic subunit | P60510 |  | 0.95 |  |
| 3148 | DPOE3_HUMAN | DNA polymerase epsilon subunit 3 | Q9NRF9 |  | 0.54 |  |
| 3149 | ZHX2_HUMAN | Zinc fingers and homeoboxes protein 2 | Q9Y6X8 |  | 0.86 |  |
| 3150 | ZN207_HUMAN | BUB3-interacting and GLEBS motif-containing protein ZNF207 | O43670 |  | 1.37 |  |
| 3151 | A16A1_HUMAN | Aldehyde dehydrogenase family 16 member A1 | Q8IZ83 |  | 0.83 |  |
| 3152 | MRP1_HUMAN | Multidrug resistance-associated protein 1 | P33527 |  | 1.34 |  |
| 3153 | ADAM9_HUMAN | Disintegrin and metalloproteinase domain-containing protein 9 | Q13443 |  | 0.84 |  |
| 3154 | CHRD1_HUMAN | Cysteine and histidine-rich domain-containing protein 1 | Q9UHD1 |  | 1.42 |  |
| 3155 | CRNS1_HUMAN | Carnosine synthase 1 | A5YM72 |  | 0.55 |  |
| 3156 | DCTN6_HUMAN | Dynactin subunit 6 | O00399 |  | 1.07 |  |
| 3157 | PPP6_HUMAN | Serine/threonine-protein phosphatase 6 catalytic subunit | O00743 |  | 1.18 |  |
| 3158 | DVL1_HUMAN | Segment polarity protein dishevelled homolog DVL-1 | O14640 |  | 0.58 |  |
| 3159 | STX16_HUMAN | Syntaxin-16 | O14662 |  | 0.94 |  |
| 3160 | SC16A_HUMAN | Protein transport protein Sec16A | O15027 |  | 1.02 |  |
| 3161 | TIF1A_HUMAN | Transcription intermediary factor 1-alpha | O15164 |  | 0.27 |  |
| 3162 | RENR_HUMAN | Renin receptor | O75787 |  | 2.65 |  |
| 3163 | KV120_HUMAN | Ig kappa chain V-I region Mev | P01612 |  | 0.95 |  |
| 3164 | TRBM_HUMAN | Thrombomodulin | P07204 |  | 1.38 |  |
| 3165 | BGLR_HUMAN | Beta-glucuronidase | P08236 |  |  |  |
| 3166 | ELNE_HUMAN | Neutrophil elastase | P08246 |  |  |  |
| 3167 | ITB4_HUMAN | Integrin beta-4 | P16144 |  |  |  |
| 3168 | PTN2_HUMAN | Tyrosine-protein phosphatase non-receptor type 2 | P17706 |  | 5.70 |  |
| 3169 | CBL_HUMAN | E3 ubiquitin-protein ligase CBL | P22681 |  | 1.24 |  |
| 3170 | 1A29_HUMAN | HLA class I histocompatibility antigen, A-29 alpha chain | P30512 |  | 1.00 |  |
| 3171 | PLPL4_HUMAN | Patatin-like phospholipase domain-containing protein 4 | P41247 |  | 0.86 |  |
| 3172 | PCP_HUMAN | Lysosomal Pro-X carboxypeptidase | P42785 |  | 0.41 |  |
| 3173 | NU153_HUMAN | Nuclear pore complex protein Nup153 | P49790 |  | 1.09 |  |
| 3174 | PSME3_HUMAN | Proteasome activator complex subunit 3 | P61289 |  | 1.06 |  |
| 3175 | ACTA_HUMAN | Actin, aortic smooth muscle | P62736 |  | 1.09 |  |
| 3176 | NOP14_HUMAN | Nucleolar protein 14 | P78316 |  | 0.53 |  |
| 3177 | IFM3_HUMAN | Interferon-induced transmembrane protein 3 | Q01628 |  | 0.99 |  |
| 3178 | SRS11_HUMAN | Serine/arginine-rich splicing factor 11 | Q05519 |  | 1.10 |  |
| 3179 | ATR_HUMAN | Serine/threonine-protein kinase ATR | Q13535 |  | 1.26 |  |
| 3180 | DIP2A_HUMAN | Disco-interacting protein 2 homolog A | Q14689 |  | 1.61 |  |
| 3181 | MFS10_HUMAN | Major facilitator superfamily domain-containing protein 10 | Q14728 |  | 0.90 |  |
| 3182 | LRC14_HUMAN | Leucine-rich repeat-containing protein 14 | Q15048 |  | 0.80 |  |
| 3183 | SNUT2_HUMAN | U4/U6.U5 tri-snRNP-associated protein 2 | Q53GS9 |  | 0.49 |  |
| 3184 | LBH_HUMAN | Protein LBH | Q53QV2 |  | 1.09 |  |
| 3185 | PIGG_HUMAN | GPI ethanolamine phosphate transferase 2 | Q5H8A4 |  | 1.41 |  |
| 3186 | K1614_HUMAN | Uncharacterized protein KIAA1614 | Q5VZ46 |  | 1.80 |  |
| 3187 | EGFLA_HUMAN | Pikachurin | Q63HQ2 |  | 0.72 |  |
| 3188 | H2B2C_HUMAN | Putative histone H2B type 2-C | Q6DN03 |  | 1.13 |  |
| 3189 | OTU7B_HUMAN | OTU domain-containing protein 7B | Q6GQQ9 |  | 0.58 |  |
| 3190 | WDR87_HUMAN | WD repeat-containing protein 87 | Q6ZQQ6 |  | 9.38 |  |
| 3191 | ACD11_HUMAN | Acyl-CoA dehydrogenase family member 11 | Q709F0 |  | 1.07 |  |
| 3192 | SAMC_HUMAN | S-adenosylmethionine mitochondrial carrier protein | Q70HW3 |  | 1.00 |  |
| 3193 | ADCK1_HUMAN | Uncharacterized aarF domain-containing protein kinase 1 | Q86TW2 |  | 0.79 |  |
| 3194 | DTD1_HUMAN | D-tyrosyl-tRNA(Tyr) deacylase 1 | Q8TEA8 |  | 0.46 |  |
| 3195 | SETD7_HUMAN | Histone-lysine N-methyltransferase SETD7 | Q8WTS6 |  | 1.12 |  |
| 3196 | PTPM1_HUMAN | Phosphatidylglycerophosphatase and protein-tyrosine phosphatase 1 | Q8WUK0 |  | 0.90 |  |
| 3197 | PPR1C_HUMAN | Protein phosphatase 1 regulatory subunit 1C | Q8WVI7 |  | 0.49 |  |
| 3198 | CHC10_HUMAN | Coiled-coil-helix-coiled-coil-helix domain-containing protein 10, mitochondrial | Q8WYQ3 |  | 0.95 |  |
| 3199 | RPE_HUMAN | Ribulose-phosphate 3-epimerase | Q96AT9 |  | 1.17 |  |
| 3200 | MIB2_HUMAN | E3 ubiquitin-protein ligase MIB2 | Q96AX9 |  | 0.65 |  |
| 3201 | CP058_HUMAN | UPF0420 protein C16orf58 | Q96GQ5 |  | 0.98 |  |
| 3202 | FXL18_HUMAN | F-box/LRR-repeat protein 18 | Q96ME1 |  | 0.72 |  |
| 3203 | CNT3B_HUMAN | Contactin-associated protein-like 3B | Q96NU0 |  | 1.15 |  |
| 3204 | NED4L_HUMAN | E3 ubiquitin-protein ligase NEDD4-like | Q96PU5 |  | 1.11 |  |
| 3205 | MMS19_HUMAN | MMS19 nucleotide excision repair protein homolog | Q96T76 |  | 0.97 |  |
| 3206 | MICU1_HUMAN | Calcium uptake protein 1, mitochondrial | Q9BPX6 |  | 1.32 |  |
| 3207 | GORS1_HUMAN | Golgi reassembly-stacking protein 1 | Q9BQQ3 |  | 1.25 |  |
| 3208 | URM1_HUMAN | Ubiquitin-related modifier 1 | Q9BTM9 |  | 0.97 |  |
| 3209 | CA050_HUMAN | Uncharacterized protein C1orf50 | Q9BV19 |  | 2.44 |  |
| 3210 | LS14B_HUMAN | Protein LSM14 homolog B | Q9BX40 |  | 1.69 |  |
| 3211 | RWDD1_HUMAN | RWD domain-containing protein 1 | Q9H446 |  | 0.19 |  |
| 3212 | PKHA2_HUMAN | Pleckstrin homology domain-containing family A member 2 | Q9HB19 |  | 1.08 |  |
| 3213 | BRE_HUMAN | BRCA1-A complex subunit BRE | Q9NXR7 |  | 0.97 |  |
| 3214 | DHCR7_HUMAN | 7-dehydrocholesterol reductase | Q9UBM7 |  | 0.89 |  |
| 3215 | DRG1_HUMAN | Developmentally-regulated GTP-binding protein 1 | Q9Y295 |  | 1.34 |  |
| 3216 | TPPC1_HUMAN | Trafficking protein particle complex subunit 1 | Q9Y5R8 |  | 1.11 |  |
| 3217 | CP080_HUMAN | UPF0468 protein C16orf80 | Q9Y6A4 |  | 1.69 |  |
| 3218 | SPCS1_HUMAN | Signal peptidase complex subunit 1 | Q9Y6A9 |  | 0.48 |  |
| 3219 | VPS4B_HUMAN | Vacuolar protein sorting-associated protein 4B | O75351 |  | 1.25 |  |
| 3220 | ATG7_HUMAN | Ubiquitin-like modifier-activating enzyme ATG7 | O95352 |  | 0.81 |  |
| 3221 | RU2B_HUMAN | U2 small nuclear ribonucleoprotein B'' | P08579 |  | 0.94 |  |
| 3222 | IBP2_HUMAN | Insulin-like growth factor-binding protein 2 | P18065 |  | 0.85 |  |
| 3223 | CD82_HUMAN | CD82 antigen | P27701 |  | 0.95 |  |
| 3224 | UBP4_HUMAN | Ubiquitin carboxyl-terminal hydrolase 4 | Q13107 |  | 1.94 |  |
| 3225 | RRS1_HUMAN | Ribosome biogenesis regulatory protein homolog | Q15050 |  |  |  |
| 3226 | AL1L2_HUMAN | Mitochondrial 10-formyltetrahydrofolate dehydrogenase | Q3SY69 |  | 1.08 |  |
| 3227 | CK083_HUMAN | UPF0723 protein C11orf83 | Q6UW78 |  | 0.96 |  |
| 3228 | KCTD9_HUMAN | BTB/POZ domain-containing protein KCTD9 | Q7L273 |  | 6.73 |  |
| 3229 | RM10_HUMAN | 39S ribosomal protein L10, mitochondrial | Q7Z7H8 |  | 0.98 |  |
| 3230 | PNKD_HUMAN | Probable hydrolase PNKD | Q8N490 |  | 1.32 |  |
| 3231 | CHMP7_HUMAN | Charged multivesicular body protein 7 | Q8WUX9 |  | 1.57 |  |
| 3232 | NR4A3_HUMAN | Nuclear receptor subfamily 4 group A member 3 | Q92570 |  | 1.02 |  |
| 3233 | SYN2_HUMAN | Synapsin-2 | Q92777 |  | 0.86 |  |
| 3234 | OTUL_HUMAN | Ubiquitin thioesterase otulin | Q96BN8 |  | 0.66 |  |
| 3235 | DPYL5_HUMAN | Dihydropyrimidinase-related protein 5 | Q9BPU6 |  | 1.14 |  |
| 3236 | NRIP2_HUMAN | Nuclear receptor-interacting protein 2 | Q9BQI9 |  | 0.98 |  |
| 3237 | ALKB7_HUMAN | Alpha-ketoglutarate-dependent dioxygenase alkB homolog 7, mitochondrial | Q9BT30 |  | 2.05 |  |
| 3238 | GOLP3_HUMAN | Golgi phosphoprotein 3 | Q9H4A6 |  | 0.95 |  |
| 3239 | TM38A_HUMAN | Trimeric intracellular cation channel type A | Q9H6F2 |  | 0.66 |  |
| 3240 | ABCF3_HUMAN | ATP-binding cassette sub-family F member 3 | Q9NUQ8 |  | 0.76 |  |
| 3241 | TOM7_HUMAN | Mitochondrial import receptor subunit TOM7 homolog | Q9P0U1 |  | 0.63 |  |
| 3242 | PPIE_HUMAN | Peptidyl-prolyl cis-trans isomerase E | Q9UNP9 |  | 2.01 |  |
| 3243 | RPKL1_HUMAN | Ribosomal protein S6 kinase-like 1 | Q9Y6S9 |  | 1.07 |  |
| 3244 | CD151_HUMAN | CD151 antigen | P48509 |  | 0.27 |  |
| 3245 | AIF1_HUMAN | Allograft inflammatory factor 1 | P55008 |  | 0.60 |  |
| 3246 | LIPA1_HUMAN | Liprin-alpha-1 | Q13136 |  | 1.00 |  |
| 3247 | TSR1_HUMAN | Pre-rRNA-processing protein TSR1 homolog | Q2NL82 |  | 0.91 |  |
| 3248 | HKDC1_HUMAN | Putative hexokinase HKDC1 | Q2TB90 |  | 1.00 |  |
| 3249 | S11IP_HUMAN | Serine/threonine-protein kinase 11-interacting protein | Q8N1F8 |  | 2.73 |  |
| 3250 | NUP37_HUMAN | Nucleoporin Nup37 | Q8NFH4 |  | 1.50 |  |
| 3251 | NECP2_HUMAN | Adaptin ear-binding coat-associated protein 2 | Q9NVZ3 |  | 0.67 |  |
| 3252 | REV1_HUMAN | DNA repair protein REV1 | Q9UBZ9 |  | 2.36 |  |
| 3253 | BT3A3_HUMAN | Butyrophilin subfamily 3 member A3 | O00478 |  | 0.95 |  |
| 3254 | NU3M_HUMAN | NADH-ubiquinone oxidoreductase chain 3 | P03897 |  | 0.91 |  |
| 3255 | MMP2_HUMAN | 72 kDa type IV collagenase | P08253 |  | 1.54 |  |
| 3256 | L1CAM_HUMAN | Neural cell adhesion molecule L1 | P32004 |  | 0.48 |  |
| 3257 | 8ODP_HUMAN | 7,8-dihydro-8-oxoguanine triphosphatase | P36639 |  | 1.21 |  |
| 3258 | KPCD_HUMAN | Protein kinase C delta type | Q05655 |  | 0.46 |  |
| 3259 | ZN846_HUMAN | Zinc finger protein 846 | Q147U1 |  | 2.11 |  |
| 3260 | COG1_HUMAN | Conserved oligomeric Golgi complex subunit 1 | Q8WTW3 |  | 0.67 |  |
| 3261 | THIC_HUMAN | Acetyl-CoA acetyltransferase, cytosolic | Q9BWD1 |  | 0.86 |  |
| 3262 | LSM7_HUMAN | U6 snRNA-associated Sm-like protein LSm7 | Q9UK45 |  | 0.47 |  |
| 3263 | TBL2_HUMAN | Transducin beta-like protein 2 | Q9Y4P3 |  | 0.88 |  |
| 3264 | PDDC1_HUMAN | Parkinson disease 7 domain-containing protein 1 | Q8NB37 |  | 0.41 |  |
| 3265 | FA65A_HUMAN | Protein FAM65A | Q6ZS17 |  | 0.98 |  |
| 3266 | NUDC2_HUMAN | NudC domain-containing protein 2 | Q8WVJ2 |  | 1.08 |  |
| 3267 | EDC4_HUMAN | Enhancer of mRNA-decapping protein 4 | Q6P2E9 |  | 1.39 |  |
| 3268 | LR75A_HUMAN | Leucine-rich repeat-containing protein 75A | Q8NAA5 |  | 0.54 |  |
| 3269 | RPR1B_HUMAN | Regulation of nuclear pre-mRNA domain-containing protein 1B | Q9NQG5 |  | 0.96 |  |
| 3270 | PSB9_HUMAN | Proteasome subunit beta type-9 | P28065 |  | 0.97 |  |
| 3271 | RHOB_HUMAN | Rho-related GTP-binding protein RhoB | P62745 |  | 0.85 |  |
| 3272 | PPIL4_HUMAN | Peptidyl-prolyl cis-trans isomerase-like 4 | Q8WUA2 |  | 1.41 |  |
| 3273 | 2B17_HUMAN | HLA class II histocompatibility antigen, DRB1-7 beta chain | P13761 |  | 0.81 |  |
| 3274 | RM42_HUMAN | 39S ribosomal protein L42, mitochondrial | Q9Y6G3 |  | 0.81 |  |
| 3275 | ACTN3_HUMAN | Alpha-actinin-3 | Q08043 |  | 0.99 |  |
| 3276 | RT12_HUMAN | 28S ribosomal protein S12, mitochondrial | O15235 |  | 2.65 |  |
| 3277 | GPI8_HUMAN | GPI-anchor transamidase | Q92643 |  | 0.77 |  |
| 3278 | LIAS_HUMAN | Lipoyl synthase, mitochondrial | O43766 |  | 1.15 |  |
| 3279 | C2AIL_HUMAN | CDKN2AIP N-terminal-like protein | Q96HQ2 |  | 0.29 |  |
| 3280 | COG3_HUMAN | Conserved oligomeric Golgi complex subunit 3 | Q96JB2 |  | 0.91 |  |
| 3281 | SUMO3_HUMAN | Small ubiquitin-related modifier 3 | P55854 |  | 1.10 |  |
| 3282 | RB3GP_HUMAN | Rab3 GTPase-activating protein catalytic subunit | Q15042 |  | 1.24 |  |
| 3283 | NCDN_HUMAN | Neurochondrin | Q9UBB6 |  | 1.74 |  |
| 3284 | CELF1_HUMAN | CUGBP Elav-like family member 1 | Q92879 |  | 0.47 |  |
| 3285 | MTHFS_HUMAN | 5-formyltetrahydrofolate cyclo-ligase | P49914 |  | 1.69 |  |
| 3286 | ATOX1_HUMAN | Copper transport protein ATOX1 | O00244 |  | 0.86 |  |
| 3287 | TI23B_HUMAN | Putative mitochondrial import inner membrane translocase subunit Tim23B | Q5SRD1 |  | 1.14 |  |
| 3288 | TPPC8_HUMAN | Trafficking protein particle complex subunit 8 | Q9Y2L5 |  | 0.96 |  |
| 3289 | UB2D3_HUMAN | Ubiquitin-conjugating enzyme E2 D3 | P61077 |  | 3.91 |  |
| 3290 | FA21B_HUMAN | WASH complex subunit FAM21B | Q5SNT6 |  | 0.87 |  |
| 3291 | HIG2A_HUMAN | HIG1 domain family member 2A, mitochondrial | Q9BW72 |  | 0.99 |  |
| 3292 | FXRD1_HUMAN | FAD-dependent oxidoreductase domain-containing protein 1 | Q96CU9 |  | 0.79 |  |
| 3293 | CNBP_HUMAN | Cellular nucleic acid-binding protein | P62633 |  | 1.25 |  |
| 3294 | PLOD1_HUMAN | Procollagen-lysine,2-oxoglutarate 5-dioxygenase 1 | Q02809 |  | 0.75 |  |
| 3295 | KV203_HUMAN | Ig kappa chain V-II region MIL | P01616 |  | 1.04 |  |
| 3296 | RS26L_HUMAN | Putative 40S ribosomal protein S26-like 1 | Q5JNZ5 |  |  |  |
| 3297 | TENC1_HUMAN | Tensin-like C1 domain-containing phosphatase | Q63HR2 |  | 1.09 |  |
| 3298 | SF3B4_HUMAN | Splicing factor 3B subunit 4 | Q15427 |  | 0.59 |  |
| 3299 | VKGC_HUMAN | Vitamin K-dependent gamma-carboxylase | P38435 |  | 0.88 |  |
| 3300 | RASH_HUMAN | GTPase HRas | P01112 |  | 0.84 |  |
| 3301 | ATP8_HUMAN | ATP synthase protein 8 | P03928 |  | 1.04 |  |
| 3302 | MPRI_HUMAN | Cation-independent mannose-6-phosphate receptor | P11717 |  | 0.69 |  |
| 3303 | EIF1_HUMAN | Eukaryotic translation initiation factor 1 | P41567 |  |  |  |
| 3304 | PDS5A_HUMAN | Sister chromatid cohesion protein PDS5 homolog A | Q29RF7 |  | 0.95 |  |
| 3305 | DHAK_HUMAN | Bifunctional ATP-dependent dihydroxyacetone kinase/FAD-AMP lyase (cyclizing) | Q3LXA3 |  | 1.09 |  |
| 3306 | DNLZ_HUMAN | DNL-type zinc finger protein | Q5SXM8 |  | 0.93 |  |
| 3307 | T126B_HUMAN | Complex I assembly factor TMEM126B, mitochondrial | Q8IUX1 |  | 1.22 |  |
| 3308 | PREB_HUMAN | Prolactin regulatory element-binding protein | Q9HCU5 |  | 1.32 |  |
| 3309 | ITIH5_HUMAN | Inter-alpha-trypsin inhibitor heavy chain H5 | Q86UX2 |  | 1.87 |  |
| 3310 | STK38_HUMAN | Serine/threonine-protein kinase 38 | Q15208 |  | 1.04 |  |
| 3311 | BRK1_HUMAN | Protein BRICK1 | Q8WUW1 |  | 0.99 |  |
| 3312 | 5NTC_HUMAN | Cytosolic purine 5'-nucleotidase | P49902 |  | 0.32 |  |
| 3313 | POTEF_HUMAN | POTE ankyrin domain family member F | A5A3E0 |  | 1.12 |  |
| 3314 | HV102_HUMAN | Ig heavy chain V-I region HG3 | P01743 |  | 1.19 |  |
| 3315 | C4BPA_HUMAN | C4b-binding protein alpha chain | P04003 |  | 0.87 |  |
| 3316 | ST1A2_HUMAN | Sulfotransferase 1A2 | P50226 |  | 0.82 |  |
| 3317 | GSTM4_HUMAN | Glutathione S-transferase Mu 4 | Q03013 |  | 1.09 |  |
| 3318 | KV404_HUMAN | Ig kappa chain V-IV region B17 | P06314 |  | 1.57 |  |
| 3319 | NCOAT_HUMAN | Bifunctional protein NCOAT | O60502 |  | 0.93 |  |
| 3320 | IF2GL_HUMAN | Putative eukaryotic translation initiation factor 2 subunit 3-like protein | Q2VIR3 |  | 1.22 |  |
| 3321 | AT5G2_HUMAN | ATP synthase F(0) complex subunit C2, mitochondrial | Q06055 |  | 0.97 |  |
| 3322 | NOMO2_HUMAN | Nodal modulator 2 | Q5JPE7 |  | 1.41 |  |
| 3323 | 1B07_HUMAN | HLA class I histocompatibility antigen, B-7 alpha chain | P01889 |  | 7.66 |  |
| 3324 | EIFCL_HUMAN | Eukaryotic translation initiation factor 3 subunit C-like protein | B5ME19 |  | 0.94 |  |
| 3325 | TBA1C_HUMAN | Tubulin alpha-1C chain | Q9BQE3 |  | 0.57 |  |
| 3326 | IGHG3_HUMAN | Ig gamma-3 chain C region | P01860 |  | 0.54 |  |
| 3327 | 1A74_HUMAN | HLA class I histocompatibility antigen, A-74 alpha chain | P30459 |  |  |  |
| 3328 | ARF3_HUMAN | ADP-ribosylation factor 3 | P61204 |  |  |  |
| 3329 | APOOL_HUMAN | Apolipoprotein O-like | Q6UXV4 |  |  |  |
| 3330 | CHCH3_HUMAN | Coiled-coil-helix-coiled-coil-helix domain-containing protein 3, mitochondrial | Q9NX63 |  |  |  |
| 3331 | IMMT_HUMAN | Mitochondrial inner membrane protein | Q16891 |  |  |  |
| 3332 | HS71B_HUMAN | Heat shock 70 kDa protein 1B | P0DMV9 |  |  | 1.10 |
| 3333 | H2A2C_HUMAN | Histone H2A type 2-C | Q16777 |  |  | 1.01 |
| 3334 | CO5A2_HUMAN | Collagen alpha-2(V) chain | P05997 |  |  | 1.15 |
| 3335 | NOMO3_HUMAN | Nodal modulator 3 | P69849 |  |  | 0.83 |
| 3336 | 1A24_HUMAN | HLA class I histocompatibility antigen, A-24 alpha chain | P05534 |  |  | 1.02 |
| 3337 | STXB3_HUMAN | Syntaxin-binding protein 3 | O00186 |  |  | 0.95 |
| 3338 | SNX5_HUMAN | Sorting nexin-5 | Q9Y5X3 |  |  | 0.80 |
| 3339 | SGT1_HUMAN | Protein SGT1 homolog | Q9Y2Z0 |  |  | 1.07 |
| 3340 | SAFB1_HUMAN | Scaffold attachment factor B1 | Q15424 |  |  | 0.83 |
| 3341 | SF3A1_HUMAN | Splicing factor 3A subunit 1 | Q15459 |  |  | 0.92 |
| 3342 | MIC26_HUMAN | MICOS complex subunit MIC26 | Q9BUR5 |  |  | 1.14 |
| 3343 | STXB1_HUMAN | Syntaxin-binding protein 1 | P61764 |  |  | 0.94 |
| 3344 | COR1B_HUMAN | Coronin-1B | Q9BR76 |  |  | 1.12 |
| 3345 | TNS2_HUMAN | Tensin-2 | Q63HR2 |  |  | 1.18 |
| 3346 | FA21A_HUMAN | WASH complex subunit FAM21A | Q641Q2 |  |  | 0.72 |
| 3347 | KV312_HUMAN | Ig kappa chain V-III region HAH | P18135 |  |  | 0.79 |
| 3348 | EXOC3_HUMAN | Exocyst complex component 3 | O60645 |  |  | 0.94 |
| 3349 | RL40_HUMAN | Ubiquitin-60S ribosomal protein L40 | P62987 |  |  | 0.99 |
| 3350 | NASP_HUMAN | Nuclear autoantigenic sperm protein | P49321 |  |  | 0.99 |
| 3351 | ARMT1_HUMAN | Protein-glutamate O-methyltransferase | Q9H993 |  |  | 1.00 |
| 3352 | PML_HUMAN | Protein PML | P29590 |  |  | 0.87 |
| 3353 | MIC13_HUMAN | MICOS complex subunit MIC13 | Q5XKP0 |  |  | 0.92 |
| 3354 | MIA3_HUMAN | Melanoma inhibitory activity protein 3 | Q5JRA6 |  |  | 1.03 |
| 3355 | SART3_HUMAN | Squamous cell carcinoma antigen recognized by T-cells 3 | Q15020 |  |  | 0.84 |
| 3356 | DNJB9_HUMAN | DnaJ homolog subfamily B member 9 | Q9UBS3 |  |  | 0.85 |
| 3357 | SWP70_HUMAN | Switch-associated protein 70 | Q9UH65 |  |  | 0.91 |
| 3358 | GOGB1_HUMAN | Golgin subfamily B member 1 | Q14789 |  |  | 1.36 |
| 3359 | SUSD2_HUMAN | Sushi domain-containing protein 2 | Q9UGT4 |  |  | 0.89 |
| 3360 | PHYD1_HUMAN | Phytanoyl-CoA dioxygenase domain-containing protein 1 | Q5SRE7 |  |  | 1.06 |
| 3361 | PYRD2_HUMAN | Pyridine nucleotide-disulfide oxidoreductase domain-containing protein 2 | Q8N2H3 |  |  | 0.91 |
| 3362 | GSTT1_HUMAN | Glutathione S-transferase theta-1 | P30711 |  |  | 0.99 |
| 3363 | RABE1_HUMAN | Rab GTPase-binding effector protein 1 | Q15276 |  |  | 0.93 |
| 3364 | THBG_HUMAN | Thyroxine-binding globulin | P05543 |  |  | 0.82 |
| 3365 | IRGQ_HUMAN | Immunity-related GTPase family Q protein | Q8WZA9 |  |  | 0.69 |
| 3366 | ABHEB_HUMAN | Alpha/beta hydrolase domain-containing protein 14B | Q96IU4 |  |  | 0.86 |
| 3367 | LC7L2_HUMAN | Putative RNA-binding protein Luc7-like 2 | Q9Y383 |  |  | 1.02 |
| 3368 | RBBP7_HUMAN | Histone-binding protein RBBP7 | Q16576 |  |  | 0.96 |
| 3369 | CX6A2_HUMAN | Cytochrome c oxidase subunit 6A2, mitochondrial | Q02221 |  |  | 0.67 |
| 3370 | UACA_HUMAN | Uveal autoantigen with coiled-coil domains and ankyrin repeats | Q9BZF9 |  |  | 0.89 |
| 3371 | NXP20_HUMAN | Protein NOXP20 | Q8IWE2 |  |  | 1.03 |
| 3372 | POF1B_HUMAN | Protein POF1B | Q8WVV4 |  |  | 1.18 |
| 3373 | P5CS_HUMAN | Delta-1-pyrroline-5-carboxylate synthase | P54886 |  |  | 0.40 |
| 3374 | EXC6B_HUMAN | Exocyst complex component 6B | Q9Y2D4 |  |  | 0.92 |
| 3375 | KPB1_HUMAN | Phosphorylase b kinase regulatory subunit alpha, skeletal muscle isoform | P46020 |  |  | 0.70 |
| 3376 | CISD2_HUMAN | CDGSH iron-sulfur domain-containing protein 2 | Q8N5K1 |  |  | 0.97 |
| 3377 | HTSF1_HUMAN | HIV Tat-specific factor 1 | O43719 |  |  | 0.86 |
| 3378 | CCD51_HUMAN | Coiled-coil domain-containing protein 51 | Q96ER9 |  |  | 0.79 |
| 3379 | PPCS_HUMAN | Phosphopantothenate--cysteine ligase | Q9HAB8 |  |  | 0.97 |
| 3380 | KCC1D_HUMAN | Calcium/calmodulin-dependent protein kinase type 1D | Q8IU85 |  |  | 0.68 |
| 3381 | EWS_HUMAN | RNA-binding protein EWS | Q01844 |  |  | 0.81 |
| 3382 | PDCD4_HUMAN | Programmed cell death protein 4 | Q53EL6 |  |  | 1.02 |
| 3383 | SRSF4_HUMAN | Serine/arginine-rich splicing factor 4 | Q08170 |  |  | 1.10 |
| 3384 | SEC63_HUMAN | Translocation protein SEC63 homolog | Q9UGP8 |  |  | 0.96 |
| 3385 | RS17_HUMAN | 40S ribosomal protein S17 | P08708 |  |  | 0.72 |
| 3386 | OPTN_HUMAN | Optineurin | Q96CV9 |  |  | 1.21 |
| 3387 | JIP4_HUMAN | C-Jun-amino-terminal kinase-interacting protein 4 | O60271 |  |  | 1.36 |
| 3388 | COMDA_HUMAN | COMM domain-containing protein 10 | Q9Y6G5 |  |  | 1.41 |
| 3389 | APOL1_HUMAN | Apolipoprotein L1 | O14791 |  |  | 0.74 |
| 3390 | PKHA7_HUMAN | Pleckstrin homology domain-containing family A member 7 | Q6IQ23 |  |  | 1.19 |
| 3391 | CCD93_HUMAN | Coiled-coil domain-containing protein 93 | Q567U6 |  |  | 0.80 |
| 3392 | ABR_HUMAN | Active breakpoint cluster region-related protein | Q12979 |  |  | 0.63 |
| 3393 | ECM1_HUMAN | Extracellular matrix protein 1 | Q16610 |  |  | 0.85 |
| 3394 | VATD_HUMAN | V-type proton ATPase subunit D | Q9Y5K8 |  |  | 0.58 |
| 3395 | ACINU_HUMAN | Apoptotic chromatin condensation inducer in the nucleus | Q9UKV3 |  |  | 1.21 |
| 3396 | NEUA_HUMAN | N-acylneuraminate cytidylyltransferase | Q8NFW8 |  |  | 0.94 |
| 3397 | RU2A_HUMAN | U2 small nuclear ribonucleoprotein A' | P09661 |  |  | 0.73 |
| 3398 | SDHF2_HUMAN | Succinate dehydrogenase assembly factor 2, mitochondrial | Q9NX18 |  |  | 1.03 |
| 3399 | UBE2O_HUMAN | (E3-independent) E2 ubiquitin-conjugating enzyme | Q9C0C9 |  |  | 0.91 |
| 3400 | ST38L_HUMAN | Serine/threonine-protein kinase 38-like | Q9Y2H1 |  |  | 0.93 |
| 3401 | A4_HUMAN | Amyloid beta A4 protein | P05067 |  |  | 1.16 |
| 3402 | SYUG_HUMAN | Gamma-synuclein | O76070 |  |  | 0.93 |
| 3403 | ASML_HUMAN | N-acetylserotonin O-methyltransferase-like protein | O95671 |  |  | 0.75 |
| 3404 | SF3B2_HUMAN | Splicing factor 3B subunit 2 | Q13435 |  |  |  |
| 3405 | SEPT5_HUMAN | Septin-5 | Q99719 |  |  | 0.92 |
| 3406 | CK068_HUMAN | UPF0696 protein C11orf68 | Q9H3H3 |  |  | 0.59 |
| 3407 | HAX1_HUMAN | HCLS1-associated protein X-1 | O00165 |  |  | 0.42 |
| 3408 | RHEB_HUMAN | GTP-binding protein Rheb | Q15382 |  |  | 0.95 |
| 3409 | ITLN1_HUMAN | Intelectin-1 | Q8WWA0 |  |  | 0.82 |
| 3410 | DENR_HUMAN | Density-regulated protein | O43583 |  |  | 1.23 |
| 3411 | MCUR1_HUMAN | Mitochondrial calcium uniporter regulator 1 | Q96AQ8 |  |  |  |
| 3412 | THTPA_HUMAN | Thiamine-triphosphatase | Q9BU02 |  |  | 1.46 |
| 3413 | PMVK_HUMAN | Phosphomevalonate kinase | Q15126 |  |  | 0.84 |
| 3414 | RABL6_HUMAN | Rab-like protein 6 | Q3YEC7 |  |  | 0.53 |
| 3415 | CLCA_HUMAN | Clathrin light chain A | P09496 |  |  | 0.89 |
| 3416 | COMD9_HUMAN | COMM domain-containing protein 9 | Q9P000 |  |  | 0.82 |
| 3417 | PPCEL_HUMAN | Prolyl endopeptidase-like | Q4J6C6 |  |  | 1.02 |
| 3418 | TKFC_HUMAN | Triokinase/FMN cyclase | Q3LXA3 |  |  | 0.53 |
| 3419 | GBP1_HUMAN | Guanylate-binding protein 1 | P32455 |  |  | 0.80 |
| 3420 | HNRL1_HUMAN | Heterogeneous nuclear ribonucleoprotein U-like protein 1 | Q9BUJ2 |  |  | 0.75 |
| 3421 | NAA10_HUMAN | N-alpha-acetyltransferase 10 | P41227 |  |  | 1.16 |
| 3422 | MAGD2_HUMAN | Melanoma-associated antigen D2 | Q9UNF1 |  |  | 1.31 |
| 3423 | PP2BA_HUMAN | Serine/threonine-protein phosphatase 2B catalytic subunit alpha isoform | Q08209 |  |  |  |
| 3424 | UBF1_HUMAN | Nucleolar transcription factor 1 | P17480 |  |  | 1.17 |
| 3425 | MYH15_HUMAN | Myosin-15 | Q9Y2K3 |  |  | 0.87 |
| 3426 | FUS_HUMAN | RNA-binding protein FUS | P35637 |  |  |  |
| 3427 | SMRC2_HUMAN | SWI/SNF complex subunit SMARCC2 | Q8TAQ2 |  |  | 1.02 |
| 3428 | CD34_HUMAN | Hematopoietic progenitor cell antigen CD34 | P28906 |  |  | 0.66 |
| 3429 | RT06_HUMAN | 28S ribosomal protein S6, mitochondrial | P82932 |  |  | 0.83 |
| 3430 | CHID1_HUMAN | Chitinase domain-containing protein 1 | Q9BWS9 |  |  | 0.95 |
| 3431 | RAD21_HUMAN | Double-strand-break repair protein rad21 homolog | O60216 |  |  | 0.73 |
| 3432 | LPP3_HUMAN | Lipid phosphate phosphohydrolase 3 | O14495 |  |  | 0.99 |
| 3433 | CCD43_HUMAN | Coiled-coil domain-containing protein 43 | Q96MW1 |  |  |  |
| 3434 | CACO1_HUMAN | Calcium-binding and coiled-coil domain-containing protein 1 | Q9P1Z2 |  |  |  |
| 3435 | P4HA1_HUMAN | Prolyl 4-hydroxylase subunit alpha-1 | P13674 |  |  | 0.81 |
| 3436 | PI42B_HUMAN | Phosphatidylinositol 5-phosphate 4-kinase type-2 beta | P78356 |  |  | 0.79 |
| 3437 | HMCN2_HUMAN | Hemicentin-2 | Q8NDA2 |  |  | 1.39 |
| 3438 | CO052_HUMAN | Uncharacterized protein C15orf52 | Q6ZUT6 |  |  | 0.73 |
| 3439 | HMGN1_HUMAN | Non-histone chromosomal protein HMG-14 | P05114 |  |  | 1.50 |
| 3440 | DDX3Y_HUMAN | ATP-dependent RNA helicase DDX3Y | O15523 |  |  | 1.08 |
| 3441 | RAB1C_HUMAN | Putative Ras-related protein Rab-1C | Q92928 |  |  | 0.68 |
| 3442 | DNJC5_HUMAN | DnaJ homolog subfamily C member 5 | Q9H3Z4 |  |  | 1.11 |
| 3443 | RM48_HUMAN | 39S ribosomal protein L48, mitochondrial | Q96GC5 |  |  | 0.90 |
| 3444 | ARMX3_HUMAN | Armadillo repeat-containing X-linked protein 3 | Q9UH62 |  |  | 0.64 |
| 3445 | LYRM1_HUMAN | LYR motif-containing protein 1 | O43325 |  |  | 0.83 |
| 3446 | MCU_HUMAN | Calcium uniporter protein, mitochondrial | Q8NE86 |  |  | 0.76 |
| 3447 | SF3A3_HUMAN | Splicing factor 3A subunit 3 | Q12874 |  |  | 0.86 |
| 3448 | EMC3_HUMAN | ER membrane protein complex subunit 3 | Q9P0I2 |  |  | 1.10 |
| 3449 | RUS1_HUMAN | RUS1 family protein C16orf58 | Q96GQ5 |  |  | 0.49 |
| 3450 | GMPPA_HUMAN | Mannose-1-phosphate guanyltransferase alpha | Q96IJ6 |  |  | 0.85 |
| 3451 | EFHD2_HUMAN | EF-hand domain-containing protein D2 | Q96C19 |  |  | 1.08 |
| 3452 | IPKA_HUMAN | cAMP-dependent protein kinase inhibitor alpha | P61925 |  |  | 1.37 |
| 3453 | SYAP1_HUMAN | Synapse-associated protein 1 | Q96A49 |  |  | 1.09 |
| 3454 | EI2BA_HUMAN | Translation initiation factor eIF-2B subunit alpha | Q14232 |  |  | 0.72 |
| 3455 | SH3G2_HUMAN | Endophilin-A1 | Q99962 |  |  | 0.84 |
| 3456 | APOD_HUMAN | Apolipoprotein D | P05090 |  |  | 1.43 |
| 3457 | GSTT2_HUMAN | Glutathione S-transferase theta-2B | P0CG30 |  |  | 1.31 |
| 3458 | T22D4_HUMAN | TSC22 domain family protein 4 | Q9Y3Q8 |  |  | 0.88 |
| 3459 | S61A1_HUMAN | Protein transport protein Sec61 subunit alpha isoform 1 | P61619 |  |  | 1.24 |
| 3460 | RAB6B_HUMAN | Ras-related protein Rab-6B | Q9NRW1 |  |  |  |
| 3461 | E41L3_HUMAN | Band 4.1-like protein 3 | Q9Y2J2 |  |  | 1.11 |
| 3462 | RAB13_HUMAN | Ras-related protein Rab-13 | P51153 |  |  | 0.84 |
| 3463 | TRFL_HUMAN | Lactotransferrin | P02788 |  |  | 0.88 |
| 3464 | STX8_HUMAN | Syntaxin-8 | Q9UNK0 |  |  | 1.13 |
| 3465 | MRC2_HUMAN | C-type mannose receptor 2 | Q9UBG0 |  |  | 0.67 |
| 3466 | PLD3_HUMAN | Phospholipase D3 | Q8IV08 |  |  | 0.74 |
| 3467 | BUB3_HUMAN | Mitotic checkpoint protein BUB3 | O43684 |  |  | 1.11 |
| 3468 | NDRG3_HUMAN | Protein NDRG3 | Q9UGV2 |  |  | 1.41 |
| 3469 | 1A01_HUMAN | HLA class I histocompatibility antigen, A-1 alpha chain | P30443 |  |  | 1.07 |
| 3470 | 1A36_HUMAN | HLA class I histocompatibility antigen, A-36 alpha chain | P30455 |  |  | 1.15 |
| 3471 | SIR2_HUMAN | NAD-dependent protein deacetylase sirtuin-2 | Q8IXJ6 |  |  | 1.14 |
| 3472 | RBM24_HUMAN | RNA-binding protein 24 | Q9BX46 |  |  | 1.04 |
| 3473 | SEPP1_HUMAN | Selenoprotein P | P49908 |  |  | 0.97 |
| 3474 | RAD50_HUMAN | DNA repair protein RAD50 | Q92878 |  |  | 0.67 |
| 3475 | NEMO_HUMAN | NF-kappa-B essential modulator | Q9Y6K9 |  |  | 0.61 |
| 3476 | NUDT3_HUMAN | Diphosphoinositol polyphosphate phosphohydrolase 1 | O95989 |  |  | 1.13 |
| 3477 | RM02_HUMAN | 39S ribosomal protein L2, mitochondrial | Q5T653 |  |  | 1.77 |
| 3478 | NEUM_HUMAN | Neuromodulin | P17677 |  |  | 0.75 |
| 3479 | SH3K1_HUMAN | SH3 domain-containing kinase-binding protein 1 | Q96B97 |  |  | 0.96 |
| 3480 | PKP4_HUMAN | Plakophilin-4 | Q99569 |  |  | 0.89 |
| 3481 | RSMB_HUMAN | Small nuclear ribonucleoprotein-associated proteins B and B' | P14678 |  |  | 1.21 |
| 3482 | ARC1B_HUMAN | Actin-related protein 2/3 complex subunit 1B | O15143 |  |  | 0.84 |
| 3483 | ANFY1_HUMAN | Rabankyrin-5 | Q9P2R3 |  |  | 1.26 |
| 3484 | RASN_HUMAN | GTPase NRas | P01111 |  |  | 0.73 |
| 3485 | IFIT3_HUMAN | Interferon-induced protein with tetratricopeptide repeats 3 | O14879 |  |  |  |
| 3486 | NUDC3_HUMAN | NudC domain-containing protein 3 | Q8IVD9 |  |  | 0.79 |
| 3487 | NUDT9_HUMAN | ADP-ribose pyrophosphatase, mitochondrial | Q9BW91 |  |  | 0.74 |
| 3488 | DCTN3_HUMAN | Dynactin subunit 3 | O75935 |  |  | 0.54 |
| 3489 | RAGP1_HUMAN | Ran GTPase-activating protein 1 | P46060 |  |  | 0.97 |
| 3490 | RAB3A_HUMAN | Ras-related protein Rab-3A | P20336 |  |  | 1.31 |
| 3491 | MAPK3_HUMAN | MAP kinase-activated protein kinase 3 | Q16644 |  |  | 0.78 |
| 3492 | LIN7C_HUMAN | Protein lin-7 homolog C | Q9NUP9 |  |  | 1.01 |
| 3493 | SON_HUMAN | Protein SON | P18583 |  |  | 1.31 |
| 3494 | ABCF2_HUMAN | ATP-binding cassette sub-family F member 2 | Q9UG63 |  |  | 0.93 |
| 3495 | ARHGH_HUMAN | Rho guanine nucleotide exchange factor 17 | Q96PE2 |  |  | 0.89 |
| 3496 | RM47_HUMAN | 39S ribosomal protein L47, mitochondrial | Q9HD33 |  |  | 1.07 |
| 3497 | PAXI_HUMAN | Paxillin | P49023 |  |  | 1.28 |
| 3498 | DPYL4_HUMAN | Dihydropyrimidinase-related protein 4 | O14531 |  |  | 1.34 |
| 3499 | RAP2A_HUMAN | Ras-related protein Rap-2a | P10114 |  |  | 1.28 |
| 3500 | CO5A1_HUMAN | Collagen alpha-1(V) chain | P20908 |  |  | 0.99 |
| 3501 | RGN_HUMAN | Regucalcin | Q15493 |  |  | 0.49 |
| 3502 | JIP3_HUMAN | C-Jun-amino-terminal kinase-interacting protein 3 | Q9UPT6 |  |  | 1.09 |
| 3503 | NIF3L_HUMAN | NIF3-like protein 1 | Q9GZT8 |  |  | 0.75 |
| 3504 | CHP3_HUMAN | Calcineurin B homologous protein 3 | Q96BS2 |  |  | 1.17 |
| 3505 | ZC3HF_HUMAN | Zinc finger CCCH domain-containing protein 15 | Q8WU90 |  |  | 0.50 |
| 3506 | ADAS_HUMAN | Alkyldihydroxyacetonephosphate synthase, peroxisomal | O00116 |  |  | 1.01 |
| 3507 | RUFY1_HUMAN | RUN and FYVE domain-containing protein 1 | Q96T51 |  |  |  |
| 3508 | CNPY2_HUMAN | Protein canopy homolog 2 | Q9Y2B0 |  |  | 1.20 |
| 3509 | GT251_HUMAN | Procollagen galactosyltransferase 1 | Q8NBJ5 |  |  | 1.29 |
| 3510 | ADA_HUMAN | Adenosine deaminase | P00813 |  |  | 0.92 |
| 3511 | F177A_HUMAN | Protein FAM177A1 | Q8N128 |  |  | 1.43 |
| 3512 | ACL6A_HUMAN | Actin-like protein 6A | O96019 |  |  | 1.38 |
| 3513 | EBP_HUMAN | 3-beta-hydroxysteroid-Delta(8),Delta(7)-isomerase | Q15125 |  |  | 1.14 |
| 3514 | SMCA5_HUMAN | SWI/SNF-related matrix-associated actin-dependent regulator of chromatin subfamily A member 5 | O60264 |  |  | 0.96 |
| 3515 | S20A2_HUMAN | Sodium-dependent phosphate transporter 2 | Q08357 |  |  | 0.80 |
| 3516 | STIM1_HUMAN | Stromal interaction molecule 1 | Q13586 |  |  | 0.80 |
| 3517 | MTNB_HUMAN | Methylthioribulose-1-phosphate dehydratase | Q96GX9 |  |  | 0.97 |
| 3518 | AS3MT_HUMAN | Arsenite methyltransferase | Q9HBK9 |  |  | 0.63 |
| 3519 | PUF60_HUMAN | Poly(U)-binding-splicing factor PUF60 | Q9UHX1 |  |  | 0.64 |
| 3520 | PLCD1_HUMAN | 1-phosphatidylinositol 4,5-bisphosphate phosphodiesterase delta-1 | P51178 |  |  | 0.83 |
| 3521 | PININ_HUMAN | Pinin | Q9H307 |  |  | 1.10 |
| 3522 | ACTBM_HUMAN | Putative beta-actin-like protein 3 | Q9BYX7 |  |  | 0.50 |
| 3523 | ELMO2_HUMAN | Engulfment and cell motility protein 2 | Q96JJ3 |  |  | 1.90 |
| 3524 | IPP2_HUMAN | Protein phosphatase inhibitor 2 | P41236 |  |  | 0.56 |
| 3525 | COPZ1_HUMAN | Coatomer subunit zeta-1 | P61923 |  |  | 0.71 |
| 3526 | UFC1_HUMAN | Ubiquitin-fold modifier-conjugating enzyme 1 | Q9Y3C8 |  |  | 0.87 |
| 3527 | HIP1_HUMAN | Huntingtin-interacting protein 1 | O00291 |  |  | 1.07 |
| 3528 | LRC40_HUMAN | Leucine-rich repeat-containing protein 40 | Q9H9A6 |  |  | 0.77 |
| 3529 | FACE1_HUMAN | CAAX prenyl protease 1 homolog | O75844 |  |  | 0.83 |
| 3530 | BASP1_HUMAN | Brain acid soluble protein 1 | P80723 |  |  | 0.93 |
| 3531 | GATC_HUMAN | Glutamyl-tRNA(Gln) amidotransferase subunit C, mitochondrial | O43716 |  |  | 0.99 |
| 3532 | TTC1_HUMAN | Tetratricopeptide repeat protein 1 | Q99614 |  |  | 1.00 |
| 3533 | PA24C_HUMAN | Cytosolic phospholipase A2 gamma | Q9UP65 |  |  | 1.33 |
| 3534 | HG2A_HUMAN | HLA class II histocompatibility antigen gamma chain | P04233 |  |  | 1.15 |
| 3535 | RAB23_HUMAN | Ras-related protein Rab-23 | Q9ULC3 |  |  | 0.92 |
| 3536 | MK09_HUMAN | Mitogen-activated protein kinase 9 | P45984 |  |  | 0.89 |
| 3537 | ABCF1_HUMAN | ATP-binding cassette sub-family F member 1 | Q8NE71 |  |  | 1.13 |
| 3538 | KBP_HUMAN | KIF1-binding protein | Q96EK5 |  |  | 0.80 |
| 3539 | GBF1_HUMAN | Golgi-specific brefeldin A-resistance guanine nucleotide exchange factor 1 | Q92538 |  |  | 1.10 |
| 3540 | ODR4_HUMAN | Protein odr-4 homolog | Q5SWX8 |  |  | 1.10 |
| 3541 | ACSL4_HUMAN | Long-chain-fatty-acid--CoA ligase 4 | O60488 |  |  | 0.72 |
| 3542 | SNUT1_HUMAN | U4/U6.U5 tri-snRNP-associated protein 1 | O43290 |  |  |  |
| 3543 | GLTP_HUMAN | Glycolipid transfer protein | Q9NZD2 |  |  | 0.67 |
| 3544 | CD166_HUMAN | CD166 antigen | Q13740 |  |  | 1.23 |
| 3545 | GOGA5_HUMAN | Golgin subfamily A member 5 | Q8TBA6 |  |  | 0.99 |
| 3546 | NFL_HUMAN | Neurofilament light polypeptide | P07196 |  |  | 0.90 |
| 3547 | SRP72_HUMAN | Signal recognition particle subunit SRP72 | O76094 |  |  |  |
| 3548 | GSDMD_HUMAN | Gasdermin-D | P57764 |  |  | 1.34 |
| 3549 | PCSK1_HUMAN | ProSAAS | Q9UHG2 |  |  | 0.99 |
| 3550 | ISLR_HUMAN | Immunoglobulin superfamily containing leucine-rich repeat protein | O14498 |  |  | 0.96 |
| 3551 | NPY_HUMAN | Pro-neuropeptide Y | P01303 |  |  | 0.95 |
| 3552 | ARFP1_HUMAN | Arfaptin-1 | P53367 |  |  | 1.16 |
| 3553 | RT33_HUMAN | 28S ribosomal protein S33, mitochondrial | Q9Y291 |  |  |  |
| 3554 | DCD_HUMAN | Dermcidin | P81605 |  |  | 0.96 |
| 3555 | DDX23_HUMAN | Probable ATP-dependent RNA helicase DDX23 | Q9BUQ8 |  |  | 0.72 |
| 3556 | KDEL2_HUMAN | KDEL motif-containing protein 2 | Q7Z4H8 |  |  | 0.84 |
| 3557 | FSTL1_HUMAN | Follistatin-related protein 1 | Q12841 |  |  | 0.75 |
| 3558 | TCAL3_HUMAN | Transcription elongation factor A protein-like 3 | Q969E4 |  |  | 1.35 |
| 3559 | AKTS1_HUMAN | Proline-rich AKT1 substrate 1 | Q96B36 |  |  | 1.16 |
| 3560 | P4K2A_HUMAN | Phosphatidylinositol 4-kinase type 2-alpha | Q9BTU6 |  |  | 1.24 |
| 3561 | PKN2_HUMAN | Serine/threonine-protein kinase N2 | Q16513 |  |  | 1.36 |
| 3562 | OSGEP_HUMAN | Probable tRNA N6-adenosine threonylcarbamoyltransferase | Q9NPF4 |  |  | 0.65 |
| 3563 | IMPCT_HUMAN | Protein IMPACT | Q9P2X3 |  |  | 1.00 |
| 3564 | NUP54_HUMAN | Nucleoporin p54 | Q7Z3B4 |  |  |  |
| 3565 | EPS8_HUMAN | Epidermal growth factor receptor kinase substrate 8 | Q12929 |  |  | 1.11 |
| 3566 | BAG1_HUMAN | BAG family molecular chaperone regulator 1 | Q99933 |  |  | 1.04 |
| 3567 | PGH1_HUMAN | Prostaglandin G/H synthase 1 | P23219 |  |  | 0.51 |
| 3568 | GOLI4_HUMAN | Golgi integral membrane protein 4 | O00461 |  |  | 0.71 |
| 3569 | CSN7B_HUMAN | COP9 signalosome complex subunit 7b | Q9H9Q2 |  |  | 1.48 |
| 3570 | NHRF1_HUMAN | Na(+)/H(+) exchange regulatory cofactor NHE-RF1 | O14745 |  |  | 0.86 |
| 3571 | TPSNR_HUMAN | Tapasin-related protein | Q9BX59 |  |  | 0.75 |
| 3572 | AP3M1_HUMAN | AP-3 complex subunit mu-1 | Q9Y2T2 |  |  | 0.95 |
| 3573 | NTKL_HUMAN | N-terminal kinase-like protein | Q96KG9 |  |  | 0.91 |
| 3574 | SRXN1_HUMAN | Sulfiredoxin-1 | Q9BYN0 |  |  | 1.07 |
| 3575 | DIRA1_HUMAN | GTP-binding protein Di-Ras1 | O95057 |  |  | 1.05 |
| 3576 | TRI32_HUMAN | E3 ubiquitin-protein ligase TRIM32 | Q13049 |  |  | 0.72 |
| 3577 | TRIP6_HUMAN | Thyroid receptor-interacting protein 6 | Q15654 |  |  | 1.02 |
| 3578 | PCID2_HUMAN | PCI domain-containing protein 2 | Q5JVF3 |  |  | 0.76 |
| 3579 | NTRI_HUMAN | Neurotrimin | Q9P121 |  |  | 1.13 |
| 3580 | ENTP1_HUMAN | Ectonucleoside triphosphate diphosphohydrolase 1 | P49961 |  |  | 0.94 |
| 3581 | SMAP_HUMAN | Small acidic protein | O00193 |  |  | 0.96 |
| 3582 | COMD8_HUMAN | COMM domain-containing protein 8 | Q9NX08 |  |  | 0.90 |
| 3583 | PPA6_HUMAN | Lysophosphatidic acid phosphatase type 6 | Q9NPH0 |  |  | 0.87 |
| 3584 | LAC3_HUMAN | Ig lambda-3 chain C regions | P0CG06 |  |  | 1.04 |
| 3585 | RNF14_HUMAN | E3 ubiquitin-protein ligase RNF14 | Q9UBS8 |  |  | 0.84 |
| 3586 | OXDD_HUMAN | D-aspartate oxidase | Q99489 |  |  | 0.61 |
| 3587 | TM223_HUMAN | Transmembrane protein 223 | A0PJW6 |  |  | 0.93 |
| 3588 | HV318_HUMAN | Ig heavy chain V-III region TUR | P01779 |  |  | 1.27 |
| 3589 | SPP24_HUMAN | Secreted phosphoprotein 24 | Q13103 |  |  | 1.21 |
| 3590 | SDC4_HUMAN | Syndecan-4 | P31431 |  |  | 1.30 |
| 3591 | HTRA1_HUMAN | Serine protease HTRA1 | Q92743 |  |  | 1.01 |
| 3592 | F134C_HUMAN | Protein FAM134C | Q86VR2 |  |  | 0.79 |
| 3593 | SPCS3_HUMAN | Signal peptidase complex subunit 3 | P61009 |  |  | 0.85 |
| 3594 | RN181_HUMAN | E3 ubiquitin-protein ligase RNF181 | Q9P0P0 |  |  | 1.13 |
| 3595 | RU1C_HUMAN | U1 small nuclear ribonucleoprotein C | P09234 |  |  | 1.18 |
| 3596 | MYDGF_HUMAN | Myeloid-derived growth factor | Q969H8 |  |  | 1.17 |
| 3597 | COA4_HUMAN | Cytochrome c oxidase assembly factor 4 homolog, mitochondrial | Q9NYJ1 |  |  | 0.73 |
| 3598 | ASPC1_HUMAN | Tether containing UBX domain for GLUT4 | Q9BZE9 |  |  | 0.73 |
| 3599 | CAPG_HUMAN | Macrophage-capping protein | P40121 |  |  | 1.15 |
| 3600 | PLPL8_HUMAN | Calcium-independent phospholipase A2-gamma | Q9NP80 |  |  | 1.23 |
| 3601 | SHC2_HUMAN | SHC-transforming protein 2 | P98077 |  |  | 0.77 |
| 3602 | SHC1_HUMAN | SHC-transforming protein 1 | P29353 |  |  | 1.26 |
| 3603 | HEM6_HUMAN | Oxygen-dependent coproporphyrinogen-III oxidase, mitochondrial | P36551 |  |  | 1.62 |
| 3604 | RAB24_HUMAN | Ras-related protein Rab-24 | Q969Q5 |  |  | 1.05 |
| 3605 | CCD53_HUMAN | WASH complex subunit CCDC53 | Q9Y3C0 |  |  | 1.09 |
| 3606 | DYHC2_HUMAN | Cytoplasmic dynein 2 heavy chain 1 | Q8NCM8 |  |  | 0.86 |
| 3607 | SPS1_HUMAN | Selenide, water dikinase 1 | P49903 |  |  | 0.73 |
| 3608 | ACYP1_HUMAN | Acylphosphatase-1 | P07311 |  |  |  |
| 3609 | GPAT4_HUMAN | Glycerol-3-phosphate acyltransferase 4 | Q86UL3 |  |  | 1.01 |
| 3610 | RT02_HUMAN | 28S ribosomal protein S2, mitochondrial | Q9Y399 |  |  | 0.41 |
| 3611 | TMM47_HUMAN | Transmembrane protein 47 | Q9BQJ4 |  |  | 0.95 |
| 3612 | PLXB2_HUMAN | Plexin-B2 | O15031 |  |  | 1.30 |
| 3613 | LXN_HUMAN | Latexin | Q9BS40 |  |  | 1.02 |
| 3614 | GDS1_HUMAN | Rap1 GTPase-GDP dissociation stimulator 1 | P52306 |  |  | 0.76 |
| 3615 | ENPP4_HUMAN | Bis(5'-adenosyl)-triphosphatase ENPP4 | Q9Y6X5 |  |  | 0.66 |
| 3616 | PP1G_HUMAN | Serine/threonine-protein phosphatase PP1-gamma catalytic subunit | P36873 |  |  | 1.05 |
| 3617 | CPSF6_HUMAN | Cleavage and polyadenylation specificity factor subunit 6 | Q16630 |  |  | 1.08 |
| 3618 | NEGR1_HUMAN | Neuronal growth regulator 1 | Q7Z3B1 |  |  |  |
| 3619 | ABI1_HUMAN | Abl interactor 1 | Q8IZP0 |  |  | 0.74 |
| 3620 | FUBP3_HUMAN | Far upstream element-binding protein 3 | Q96I24 |  |  | 1.10 |
| 3621 | RRAGA_HUMAN | Ras-related GTP-binding protein A | Q7L523 |  |  |  |
| 3622 | SH3R2_HUMAN | Putative E3 ubiquitin-protein ligase SH3RF2 | Q8TEC5 |  |  | 0.68 |
| 3623 | CCAR1_HUMAN | Cell division cycle and apoptosis regulator protein 1 | Q8IX12 |  |  | 0.97 |
| 3624 | LEGL_HUMAN | Galectin-related protein | Q3ZCW2 |  |  | 1.04 |
| 3625 | RM20_HUMAN | 39S ribosomal protein L20, mitochondrial | Q9BYC9 |  |  | 0.73 |
| 3626 | THNS1_HUMAN | Threonine synthase-like 1 | Q8IYQ7 |  |  | 1.02 |
| 3627 | SYN1_HUMAN | Synapsin-1 | P17600 |  |  | 0.68 |
| 3628 | GIMA8_HUMAN | GTPase IMAP family member 8 | Q8ND71 |  |  |  |
| 3629 | HOOK3_HUMAN | Protein Hook homolog 3 | Q86VS8 |  |  | 1.31 |
| 3630 | PSD10_HUMAN | 26S proteasome non-ATPase regulatory subunit 10 | O75832 |  |  | 1.15 |
| 3631 | COMD3_HUMAN | COMM domain-containing protein 3 | Q9UBI1 |  |  | 1.00 |
| 3632 | VAT1L_HUMAN | Synaptic vesicle membrane protein VAT-1 homolog-like | Q9HCJ6 |  |  | 0.87 |
| 3633 | PMM2_HUMAN | Phosphomannomutase 2 | O15305 |  |  | 0.73 |
| 3634 | TBCD_HUMAN | Tubulin-specific chaperone D | Q9BTW9 |  |  | 1.05 |
| 3635 | DEMA_HUMAN | Dematin | Q08495 |  |  | 0.93 |
| 3636 | KAT1_HUMAN | Kynurenine--oxoglutarate transaminase 1 | Q16773 |  |  | 1.13 |
| 3637 | EPCR_HUMAN | Endothelial protein C receptor | Q9UNN8 |  |  |  |
| 3638 | ANGL2_HUMAN | Angiopoietin-related protein 2 | Q9UKU9 |  |  |  |
| 3639 | TIMP3_HUMAN | Metalloproteinase inhibitor 3 | P35625 |  |  | 0.76 |
| 3640 | CBX5_HUMAN | Chromobox protein homolog 5 | P45973 |  |  |  |
| 3641 | CHM1B_HUMAN | Charged multivesicular body protein 1b | Q7LBR1 |  |  | 0.61 |
| 3642 | ICT1_HUMAN | Peptidyl-tRNA hydrolase ICT1, mitochondrial | Q14197 |  |  | 0.74 |
| 3643 | ALG11_HUMAN | GDP-Man:Man(3)GlcNAc(2)-PP-Dol alpha-1,2-mannosyltransferase | Q2TAA5 |  |  | 0.70 |
| 3644 | MAP11_HUMAN | Methionine aminopeptidase 1 | P53582 |  |  | 0.94 |
| 3645 | SKT_HUMAN | Sickle tail protein homolog | Q5T5P2 |  |  | 1.10 |
| 3646 | GOGA4_HUMAN | Golgin subfamily A member 4 | Q13439 |  |  | 0.53 |
| 3647 | TMA7_HUMAN | Translation machinery-associated protein 7 | Q9Y2S6 |  |  | 1.05 |
| 3648 | KCC2A_HUMAN | Calcium/calmodulin-dependent protein kinase type II subunit alpha | Q9UQM7 |  |  |  |
| 3649 | EOGT_HUMAN | EGF domain-specific O-linked N-acetylglucosamine transferase | Q5NDL2 |  |  | 1.33 |
| 3650 | DNJB1_HUMAN | DnaJ homolog subfamily B member 1 | P25685 |  |  | 1.09 |
| 3651 | CD302_HUMAN | CD302 antigen | Q8IX05 |  |  | 0.71 |
| 3652 | SMYD2_HUMAN | N-lysine methyltransferase SMYD2 | Q9NRG4 |  |  | 2.74 |
| 3653 | PPM1B_HUMAN | Protein phosphatase 1B | O75688 |  |  | 0.80 |
| 3654 | LTMD1_HUMAN | LETM1 domain-containing protein 1 | Q6P1Q0 |  |  |  |
| 3655 | RIR2B_HUMAN | Ribonucleoside-diphosphate reductase subunit M2 B | Q7LG56 |  |  | 0.68 |
| 3656 | PTPRM_HUMAN | Receptor-type tyrosine-protein phosphatase mu | P28827 |  |  | 0.98 |
| 3657 | RM17_HUMAN | 39S ribosomal protein L17, mitochondrial | Q9NRX2 |  |  | 0.97 |
| 3658 | TPMT_HUMAN | Thiopurine S-methyltransferase | P51580 |  |  | 0.83 |
| 3659 | 5NT3A_HUMAN | Cytosolic 5'-nucleotidase 3A | Q9H0P0 |  |  | 0.75 |
| 3660 | SC61B_HUMAN | Protein transport protein Sec61 subunit beta | P60468 |  |  | 1.09 |
| 3661 | KPB2_HUMAN | Phosphorylase b kinase regulatory subunit alpha, liver isoform | P46019 |  |  | 1.20 |
| 3662 | SEP15_HUMAN | 15 kDa selenoprotein | O60613 |  |  | 1.33 |
| 3663 | SP16H_HUMAN | FACT complex subunit SPT16 | Q9Y5B9 |  |  | 1.64 |
| 3664 | RAE1L_HUMAN | mRNA export factor | P78406 |  |  | 1.24 |
| 3665 | SGMR1_HUMAN | Sigma non-opioid intracellular receptor 1 | Q99720 |  |  | 1.06 |
| 3666 | SDOS_HUMAN | Protein syndesmos | Q9BRJ7 |  |  | 0.97 |
| 3667 | TMUB1_HUMAN | Transmembrane and ubiquitin-like domain-containing protein 1 | Q9BVT8 |  |  | 1.59 |
| 3668 | FAF1_HUMAN | FAS-associated factor 1 | Q9UNN5 |  |  | 0.97 |
| 3669 | KV306_HUMAN | Ig kappa chain V-III region POM | P01624 |  |  | 1.20 |
| 3670 | CDK5_HUMAN | Cyclin-dependent-like kinase 5 | Q00535 |  |  | 1.25 |
| 3671 | MYO6_HUMAN | Unconventional myosin-VI | Q9UM54 |  |  | 1.17 |
| 3672 | PDS5B_HUMAN | Sister chromatid cohesion protein PDS5 homolog B | Q9NTI5 |  |  | 1.12 |
| 3673 | PPR1A_HUMAN | Protein phosphatase 1 regulatory subunit 1A | Q13522 |  |  | 1.00 |
| 3674 | NT5D1_HUMAN | 5'-nucleotidase domain-containing protein 1 | Q5TFE4 |  |  | 0.86 |
| 3675 | ABLM2_HUMAN | Actin-binding LIM protein 2 | Q6H8Q1 |  |  | 0.90 |
| 3676 | IFIX_HUMAN | Pyrin and HIN domain-containing protein 1 | Q6K0P9 |  |  | 1.15 |
| 3677 | TMM70_HUMAN | Transmembrane protein 70, mitochondrial | Q9BUB7 |  |  | 0.45 |
| 3678 | LUZP1_HUMAN | Leucine zipper protein 1 | Q86V48 |  |  | 0.84 |
| 3679 | NICA_HUMAN | Nicastrin | Q92542 |  |  | 1.38 |
| 3680 | OTU6B_HUMAN | OTU domain-containing protein 6B | Q8N6M0 |  |  | 0.63 |
| 3681 | PIR_HUMAN | Pirin | O00625 |  |  |  |
| 3682 | TB182_HUMAN | 182 kDa tankyrase-1-binding protein | Q9C0C2 |  |  |  |
| 3683 | NCHL1_HUMAN | Neural cell adhesion molecule L1-like protein | O00533 |  |  |  |
| 3684 | DEN4C_HUMAN | DENN domain-containing protein 4C | Q5VZ89 |  |  |  |
| 3685 | EDF1_HUMAN | Endothelial differentiation-related factor 1 | O60869 |  |  | 0.10 |
| 3686 | FA12_HUMAN | Coagulation factor XII | P00748 |  |  | 0.82 |
| 3687 | MBP_HUMAN | Myelin basic protein | P02686 |  |  | 1.53 |
| 3688 | RBGP1_HUMAN | Rab GTPase-activating protein 1 | Q9Y3P9 |  |  | 1.81 |
| 3689 | TR61B_HUMAN | tRNA (adenine(58)-N(1))-methyltransferase, mitochondrial | Q9BVS5 |  |  | 0.76 |
| 3690 | SMAD3_HUMAN | Mothers against decapentaplegic homolog 3 | P84022 |  |  |  |
| 3691 | SMAD2_HUMAN | Mothers against decapentaplegic homolog 2 | Q15796 |  |  |  |
| 3692 | DLG2_HUMAN | Disks large homolog 2 | Q15700 |  |  | 1.39 |
| 3693 | DLG1_HUMAN | Disks large homolog 1 | Q12959 |  |  | 0.94 |
| 3694 | GATA_HUMAN | Glutamyl-tRNA(Gln) amidotransferase subunit A, mitochondrial | Q9H0R6 |  |  | 0.88 |
| 3695 | K1C16_HUMAN | Keratin, type I cytoskeletal 16 | P08779 |  |  | 1.10 |
| 3696 | ARCH_HUMAN | Protein archease | Q8IWT0 |  |  | 0.68 |
| 3697 | PTSS1_HUMAN | Phosphatidylserine synthase 1 | P48651 |  |  | 0.93 |
| 3698 | BCLF1_HUMAN | Bcl-2-associated transcription factor 1 | Q9NYF8 |  |  | 1.28 |
| 3699 | CXAR_HUMAN | Coxsackievirus and adenovirus receptor | P78310 |  |  | 0.57 |
| 3700 | NU133_HUMAN | Nuclear pore complex protein Nup133 | Q8WUM0 |  |  | 0.67 |
| 3701 | SNTB1_HUMAN | Beta-1-syntrophin | Q13884 |  |  | 0.56 |
| 3702 | TXN4A_HUMAN | Thioredoxin-like protein 4A | P83876 |  |  | 2.76 |
| 3703 | TMCO1_HUMAN | Transmembrane and coiled-coil domain-containing protein 1 | Q9UM00 |  |  | 2.03 |
| 3704 | VAC14_HUMAN | Protein VAC14 homolog | Q08AM6 |  |  | 1.04 |
| 3705 | RASM_HUMAN | Ras-related protein M-Ras | O14807 |  |  | 0.97 |
| 3706 | SPT5H_HUMAN | Transcription elongation factor SPT5 | O00267 |  |  | 0.92 |
| 3707 | PSIP1_HUMAN | PC4 and SFRS1-interacting protein | O75475 |  |  | 0.89 |
| 3708 | SPS2L_HUMAN | SPATS2-like protein | Q9NUQ6 |  |  | 1.79 |
| 3709 | KGUA_HUMAN | Guanylate kinase | Q16774 |  |  | 0.89 |
| 3710 | PALM_HUMAN | Paralemmin-1 | O75781 |  |  | 1.11 |
| 3711 | DCAF6_HUMAN | DDB1- and CUL4-associated factor 6 | Q58WW2 |  |  | 1.57 |
| 3712 | OXA1L_HUMAN | Mitochondrial inner membrane protein OXA1L | Q15070 |  |  | 0.83 |
| 3713 | TYB4_HUMAN | Thymosin beta-4 | P62328 |  |  | 1.03 |
| 3714 | TPC12_HUMAN | Trafficking protein particle complex subunit 12 | Q8WVT3 |  |  |  |
| 3715 | COX19_HUMAN | Cytochrome c oxidase assembly protein COX19 | Q49B96 |  |  | 0.80 |
| 3716 | SSPN_HUMAN | Sarcospan | Q14714 |  |  | 1.18 |
| 3717 | RL1D1_HUMAN | Ribosomal L1 domain-containing protein 1 | O76021 |  |  | 1.14 |
| 3718 | RM27_HUMAN | 39S ribosomal protein L27, mitochondrial | Q9P0M9 |  |  | 1.23 |
| 3719 | MSRB3_HUMAN | Methionine-R-sulfoxide reductase B3 | Q8IXL7 |  |  | 0.58 |
| 3720 | UBL5_HUMAN | Ubiquitin-like protein 5 | Q9BZL1 |  |  | 0.82 |
| 3721 | TAF7_HUMAN | Transcription initiation factor TFIID subunit 7 | Q15545 |  |  | 1.46 |
| 3722 | GTR1_HUMAN | Solute carrier family 2, facilitated glucose transporter member 1 | P11166 |  |  | 1.15 |
| 3723 | ARI1_HUMAN | E3 ubiquitin-protein ligase ARIH1 | Q9Y4X5 |  |  | 0.87 |
| 3724 | BOLA3_HUMAN | BolA-like protein 3 | Q53S33 |  |  | 0.77 |
| 3725 | KV201_HUMAN | Ig kappa chain V-II region Cum | P01614 |  |  | 0.76 |
| 3726 | CYTSA_HUMAN | Cytospin-A | Q69YQ0 |  |  | 0.84 |
| 3727 | CYTSB_HUMAN | Cytospin-B | Q5M775 |  |  | 1.09 |
| 3728 | FKBP7_HUMAN | Peptidyl-prolyl cis-trans isomerase FKBP7 | Q9Y680 |  |  | 0.63 |
| 3729 | SL9A1_HUMAN | Sodium/hydrogen exchanger 1 | P19634 |  |  | 0.55 |
| 3730 | FNTB_HUMAN | Protein farnesyltransferase subunit beta | P49356 |  |  | 0.74 |
| 3731 | RS29_HUMAN | 40S ribosomal protein S29 | P62273 |  |  | 0.89 |
| 3732 | SELB_HUMAN | Selenocysteine-specific elongation factor | P57772 |  |  |  |
| 3733 | GOPC_HUMAN | Golgi-associated PDZ and coiled-coil motif-containing protein | Q9HD26 |  |  | 1.26 |
| 3734 | PRPK_HUMAN | TP53-regulating kinase | Q96S44 |  |  |  |
| 3735 | LRC39_HUMAN | Leucine-rich repeat-containing protein 39 | Q96DD0 |  |  | 1.04 |
| 3736 | SRPK1_HUMAN | SRSF protein kinase 1 | Q96SB4 |  |  | 1.00 |
| 3737 | SRPK2_HUMAN | SRSF protein kinase 2 | P78362 |  |  | 0.75 |
| 3738 | FHL3_HUMAN | Four and a half LIM domains protein 3 | Q13643 |  |  | 1.06 |
| 3739 | CARM1_HUMAN | Histone-arginine methyltransferase CARM1 | Q86X55 |  |  | 0.86 |
| 3740 | FGL2_HUMAN | Fibroleukin | Q14314 |  |  | 1.20 |
| 3741 | SPG20_HUMAN | Spartin | Q8N0X7 |  |  | 0.88 |
| 3742 | E41L1_HUMAN | Band 4.1-like protein 1 | Q9H4G0 |  |  | 0.78 |
| 3743 | RN123_HUMAN | E3 ubiquitin-protein ligase RNF123 | Q5XPI4 |  |  | 1.83 |
| 3744 | SSA27_HUMAN | Sjoegren syndrome/scleroderma autoantigen 1 | O60232 |  |  | 1.03 |
| 3745 | CPNE5_HUMAN | Copine-5 | Q9HCH3 |  |  | 0.88 |
| 3746 | SSH3_HUMAN | Protein phosphatase Slingshot homolog 3 | Q8TE77 |  |  | 1.00 |
| 3747 | IFT27_HUMAN | Intraflagellar transport protein 27 homolog | Q9BW83 |  |  | 0.95 |
| 3748 | USE1_HUMAN | Vesicle transport protein USE1 | Q9NZ43 |  |  | 1.02 |
| 3749 | ACE_HUMAN | Angiotensin-converting enzyme | P12821 |  |  | 0.94 |
| 3750 | BICD2_HUMAN | Protein bicaudal D homolog 2 | Q8TD16 |  |  | 1.65 |
| 3751 | UBL4A_HUMAN | Ubiquitin-like protein 4A | P11441 |  |  | 0.97 |
| 3752 | ASC_HUMAN | Apoptosis-associated speck-like protein containing a CARD | Q9ULZ3 |  |  | 0.70 |
| 3753 | MYCBP_HUMAN | C-Myc-binding protein | Q99417 |  |  | 1.11 |
| 3754 | STAM2_HUMAN | Signal transducing adapter molecule 2 | O75886 |  |  | 1.02 |
| 3755 | KLKB1_HUMAN | Plasma kallikrein | P03952 |  |  | 0.74 |
| 3756 | CPZIP_HUMAN | CapZ-interacting protein | Q6JBY9 |  |  | 0.46 |
| 3757 | ARFG2_HUMAN | ADP-ribosylation factor GTPase-activating protein 2 | Q8N6H7 |  |  | 1.49 |
| 3758 | XRN2_HUMAN | 5'-3' exoribonuclease 2 | Q9H0D6 |  |  | 0.80 |
| 3759 | MOB2_HUMAN | MOB kinase activator 2 | Q70IA6 |  |  | 1.01 |
| 3760 | BAK_HUMAN | Bcl-2 homologous antagonist/killer | Q16611 |  |  | 0.75 |
| 3761 | AP3S1_HUMAN | AP-3 complex subunit sigma-1 | Q92572 |  |  | 0.89 |
| 3762 | CHM2B_HUMAN | Charged multivesicular body protein 2b | Q9UQN3 |  |  | 0.62 |
| 3763 | DCAF7_HUMAN | DDB1- and CUL4-associated factor 7 | P61962 |  |  | 1.26 |
| 3764 | HN1L_HUMAN | Hematological and neurological expressed 1-like protein | Q9H910 |  |  | 0.88 |
| 3765 | HMGA1_HUMAN | High mobility group protein HMG-I/HMG-Y | P17096 |  |  | 1.11 |
| 3766 | B2CL1_HUMAN | Bcl-2-like protein 1 | Q07817 |  |  | 1.14 |
| 3767 | GBA2_HUMAN | Non-lysosomal glucosylceramidase | Q9HCG7 |  |  | 1.57 |
| 3768 | CQ062_HUMAN | Uncharacterized protein C17orf62 | Q9BQA9 |  |  | 0.59 |
| 3769 | BABA1_HUMAN | BRISC and BRCA1-A complex member 1 | Q9NWV8 |  |  |  |
| 3770 | HPTR_HUMAN | Haptoglobin-related protein | P00739 |  |  |  |
| 3771 | PTSS2_HUMAN | Phosphatidylserine synthase 2 | Q9BVG9 |  |  |  |
| 3772 | LACE1_HUMAN | Lactation elevated protein 1 | Q8WV93 |  |  | 0.47 |
| 3773 | PRP6_HUMAN | Pre-mRNA-processing factor 6 | O94906 |  |  | 1.56 |
| 3774 | MRM3_HUMAN | rRNA methyltransferase 3, mitochondrial | Q9HC36 |  |  | 0.56 |
| 3775 | UBE2H_HUMAN | Ubiquitin-conjugating enzyme E2 H | P62256 |  |  |  |
| 3776 | STAU1_HUMAN | Double-stranded RNA-binding protein Staufen homolog 1 | O95793 |  |  | 1.24 |
| 3777 | MA2B2_HUMAN | Epididymis-specific alpha-mannosidase | Q9Y2E5 |  |  | 0.64 |
| 3778 | VTI1B_HUMAN | Vesicle transport through interaction with t-SNAREs homolog 1B | Q9UEU0 |  |  | 1.29 |
| 3779 | S12A7_HUMAN | Solute carrier family 12 member 7 | Q9Y666 |  |  | 0.75 |
| 3780 | AT2L2_HUMAN | 5-phosphohydroxy-L-lysine phospho-lyase | Q8IUZ5 |  |  | 1.15 |
| 3781 | RS27L_HUMAN | 40S ribosomal protein S27-like | Q71UM5 |  |  | 0.62 |
| 3782 | UN45A_HUMAN | Protein unc-45 homolog A | Q9H3U1 |  |  | 0.89 |
| 3783 | CNRP1_HUMAN | CB1 cannabinoid receptor-interacting protein 1 | Q96F85 |  |  | 0.69 |
| 3784 | VPS41_HUMAN | Vacuolar protein sorting-associated protein 41 homolog | P49754 |  |  | 1.70 |
| 3785 | ARHGC_HUMAN | Rho guanine nucleotide exchange factor 12 | Q9NZN5 |  |  | 1.26 |
| 3786 | PMGE_HUMAN | Bisphosphoglycerate mutase | P07738 |  |  | 0.50 |
| 3787 | CYH3_HUMAN | Cytohesin-3 | O43739 |  |  | 0.77 |
| 3788 | EXOC1_HUMAN | Exocyst complex component 1 | Q9NV70 |  |  |  |
| 3789 | CUED2_HUMAN | CUE domain-containing protein 2 | Q9H467 |  |  | 1.65 |
| 3790 | TF65_HUMAN | Transcription factor p65 | Q04206 |  |  | 0.38 |
| 3791 | EFHD1_HUMAN | EF-hand domain-containing protein D1 | Q9BUP0 |  |  | 0.71 |
| 3792 | DEGS1_HUMAN | Sphingolipid delta(4)-desaturase DES1 | O15121 |  |  | 1.18 |
| 3793 | CNPY3_HUMAN | Protein canopy homolog 3 | Q9BT09 |  |  | 1.92 |
| 3794 | RBM25_HUMAN | RNA-binding protein 25 | P49756 |  |  | 0.80 |
| 3795 | CBPB2_HUMAN | Carboxypeptidase B2 | Q96IY4 |  |  | 1.25 |
| 3796 | UGDH_HUMAN | UDP-glucose 6-dehydrogenase | O60701 |  |  | 1.35 |
| 3797 | C2D1A_HUMAN | Coiled-coil and C2 domain-containing protein 1A | Q6P1N0 |  |  | 1.12 |
| 3798 | RBM14_HUMAN | RNA-binding protein 14 | Q96PK6 |  |  | 0.64 |
| 3799 | RNPS1_HUMAN | RNA-binding protein with serine-rich domain 1 | Q15287 |  |  | 1.18 |
| 3800 | HCLS1_HUMAN | Hematopoietic lineage cell-specific protein | P14317 |  |  | 1.26 |
| 3801 | RS4Y1_HUMAN | 40S ribosomal protein S4, Y isoform 1 | P22090 |  |  | 0.94 |
| 3802 | NRBP_HUMAN | Nuclear receptor-binding protein | Q9UHY1 |  |  | 0.57 |
| 3803 | SYNE3_HUMAN | Nesprin-3 | Q6ZMZ3 |  |  | 0.77 |
| 3804 | COMD2_HUMAN | COMM domain-containing protein 2 | Q86X83 |  |  | 0.82 |
| 3805 | CX6A1_HUMAN | Cytochrome c oxidase subunit 6A1, mitochondrial | P12074 |  |  | 0.96 |
| 3806 | DHR11_HUMAN | Dehydrogenase/reductase SDR family member 11 | Q6UWP2 |  |  | 1.03 |
| 3807 | CDC5L_HUMAN | Cell division cycle 5-like protein | Q99459 |  |  | 1.83 |
| 3808 | GRAM4_HUMAN | GRAM domain-containing protein 4 | Q6IC98 |  |  | 0.96 |
| 3809 | UAP1L_HUMAN | UDP-N-acetylhexosamine pyrophosphorylase-like protein 1 | Q3KQV9 |  |  |  |
| 3810 | UAP1_HUMAN | UDP-N-acetylhexosamine pyrophosphorylase | Q16222 |  |  | 1.83 |
| 3811 | GNL1_HUMAN | Guanine nucleotide-binding protein-like 1 | P36915 |  |  | 1.09 |
| 3812 | DNJB5_HUMAN | DnaJ homolog subfamily B member 5 | O75953 |  |  |  |
| 3813 | DGUOK_HUMAN | Deoxyguanosine kinase, mitochondrial | Q16854 |  |  | 0.59 |
| 3814 | IQGA2_HUMAN | Ras GTPase-activating-like protein IQGAP2 | Q13576 |  |  |  |
| 3815 | NCK2_HUMAN | Cytoplasmic protein NCK2 | O43639 |  |  | 0.90 |
| 3816 | PTH_HUMAN | Probable peptidyl-tRNA hydrolase | Q86Y79 |  |  | 0.72 |
| 3817 | VPS50_HUMAN | Syndetin | Q96JG6 |  |  | 1.88 |
| 3818 | MRCKB_HUMAN | Serine/threonine-protein kinase MRCK beta | Q9Y5S2 |  |  | 0.67 |
| 3819 | PVR_HUMAN | Poliovirus receptor | P15151 |  |  | 0.79 |
| 3820 | PPID_HUMAN | Peptidyl-prolyl cis-trans isomerase D | Q08752 |  |  | 5.29 |
| 3821 | ODF2L_HUMAN | Outer dense fiber protein 2-like | Q9ULJ1 |  |  | 0.50 |
| 3822 | DKC1_HUMAN | H/ACA ribonucleoprotein complex subunit 4 | O60832 |  |  |  |
| 3823 | CCDC6_HUMAN | Coiled-coil domain-containing protein 6 | Q16204 |  |  | 1.13 |
| 3824 | CXA5_HUMAN | Gap junction alpha-5 protein | P36382 |  |  | 0.92 |
| 3825 | XRP2_HUMAN | Protein XRP2 | O75695 |  |  | 1.99 |
| 3826 | BAP18_HUMAN | Chromatin complexes subunit BAP18 | Q8IXM2 |  |  | 1.04 |
| 3827 | HMGB2_HUMAN | High mobility group protein B2 | P26583 |  |  | 2.66 |
| 3828 | SYNM_HUMAN | Probable asparagine--tRNA ligase, mitochondrial | Q96I59 |  |  | 0.99 |
| 3829 | LRC57_HUMAN | Leucine-rich repeat-containing protein 57 | Q8N9N7 |  |  | 1.19 |
| 3830 | AUP1_HUMAN | Ancient ubiquitous protein 1 | Q9Y679 |  |  |  |
| 3831 | CHTOP_HUMAN | Chromatin target of PRMT1 protein | Q9Y3Y2 |  |  | 1.09 |
| 3832 | RBM4_HUMAN | RNA-binding protein 4 | Q9BWF3 |  |  | 0.62 |
| 3833 | JAM1_HUMAN | Junctional adhesion molecule A | Q9Y624 |  |  | 0.95 |
| 3834 | SELM_HUMAN | Selenoprotein M | Q8WWX9 |  |  | 0.91 |
| 3835 | GMFG_HUMAN | Glia maturation factor gamma | O60234 |  |  | 0.66 |
| 3836 | GHC1_HUMAN | Mitochondrial glutamate carrier 1 | Q9H936 |  |  |  |
| 3837 | GHC2_HUMAN | Mitochondrial glutamate carrier 2 | Q9H1K4 |  |  |  |
| 3838 | RIPL1_HUMAN | RILP-like protein 1 | Q5EBL4 |  |  |  |
| 3839 | SGPL1_HUMAN | Sphingosine-1-phosphate lyase 1 | O95470 |  |  | 0.90 |
| 3840 | MTNA_HUMAN | Methylthioribose-1-phosphate isomerase | Q9BV20 |  |  | 0.86 |
| 3841 | SMHD1_HUMAN | Structural maintenance of chromosomes flexible hinge domain-containing protein 1 | A6NHR9 |  |  | 1.63 |
| 3842 | MKRN2_HUMAN | Probable E3 ubiquitin-protein ligase makorin-2 | Q9H000 |  |  | 0.26 |
| 3843 | ITPK1_HUMAN | Inositol-tetrakisphosphate 1-kinase | Q13572 |  |  | 1.02 |
| 3844 | RPAB1_HUMAN | DNA-directed RNA polymerases I, II, and III subunit RPABC1 | P19388 |  |  | 0.80 |
| 3845 | OS9_HUMAN | Protein-9 | Q13438 |  |  | 1.19 |
| 3846 | CHSP1_HUMAN | Calcium-regulated heat stable protein 1 | Q9Y2V2 |  |  | 0.69 |
| 3847 | GBG8_HUMAN | Guanine nucleotide-binding protein G(I)/G(S)/G(O) subunit gamma-8 | Q9UK08 |  |  | 0.95 |
| 3848 | AT2B1_HUMAN | Plasma membrane calcium-transporting ATPase 1 | P20020 |  |  | 2.19 |
| 3849 | SYVN1_HUMAN | E3 ubiquitin-protein ligase synoviolin | Q86TM6 |  |  | 0.84 |
| 3850 | NTAN1_HUMAN | Protein N-terminal asparagine amidohydrolase | Q96AB6 |  |  | 1.41 |
| 3851 | PCKGM_HUMAN | Phosphoenolpyruvate carboxykinase [GTP], mitochondrial | Q16822 |  |  | 0.84 |
| 3852 | GCYA3_HUMAN | Guanylate cyclase soluble subunit alpha-3 | Q02108 |  |  | 1.22 |
| 3853 | COA7_HUMAN | Cytochrome c oxidase assembly factor 7 | Q96BR5 |  |  | 0.93 |
| 3854 | TM165_HUMAN | Transmembrane protein 165 | Q9HC07 |  |  |  |
| 3855 | CS052_HUMAN | Uncharacterized protein C19orf52 | Q9BSF4 |  |  | 0.83 |
| 3856 | MBNL1_HUMAN | Muscleblind-like protein 1 | Q9NR56 |  |  | 0.83 |
| 3857 | SEPT6_HUMAN | Septin-6 | Q14141 |  |  | 0.99 |
| 3858 | STX17_HUMAN | Syntaxin-17 | P56962 |  |  | 0.65 |
| 3859 | SMCE1_HUMAN | SWI/SNF-related matrix-associated actin-dependent regulator of chromatin subfamily E member 1 | Q969G3 |  |  | 0.80 |
| 3860 | MYOME_HUMAN | Myomegalin | Q5VU43 |  |  | 0.91 |
| 3861 | GCP2_HUMAN | Gamma-tubulin complex component 2 | Q9BSJ2 |  |  | 1.23 |
| 3862 | VPS16_HUMAN | Vacuolar protein sorting-associated protein 16 homolog | Q9H269 |  |  | 2.01 |
| 3863 | PI51A_HUMAN | Phosphatidylinositol 4-phosphate 5-kinase type-1 alpha | Q99755 |  |  | 1.09 |
| 3864 | CATC_HUMAN | Dipeptidyl peptidase 1 | P53634 |  |  | 1.05 |
| 3865 | GNS_HUMAN | N-acetylglucosamine-6-sulfatase | P15586 |  |  | 0.89 |
| 3866 | COX18_HUMAN | Mitochondrial inner membrane protein COX18 | Q8N8Q8 |  |  | 0.71 |
| 3867 | IBP3_HUMAN | Insulin-like growth factor-binding protein 3 | P17936 |  |  | 1.20 |
| 3868 | STAM1_HUMAN | Signal transducing adapter molecule 1 | Q92783 |  |  | 0.43 |
| 3869 | CTL1_HUMAN | Choline transporter-like protein 1 | Q8WWI5 |  |  | 0.57 |
| 3870 | CC124_HUMAN | Coiled-coil domain-containing protein 124 | Q96CT7 |  |  |  |
| 3871 | ERGI2_HUMAN | Endoplasmic reticulum-Golgi intermediate compartment protein 2 | Q96RQ1 |  |  | 0.91 |
| 3872 | DHRS1_HUMAN | Dehydrogenase/reductase SDR family member 1 | Q96LJ7 |  |  | 0.88 |
| 3873 | HS71L_HUMAN | Heat shock 70 kDa protein 1-like | P34931 |  |  |  |
| 3874 | MYH16_HUMAN | Putative uncharacterized protein MYH16 | Q9H6N6 |  |  | 2.47 |
| 3875 | SAFB2_HUMAN | Scaffold attachment factor B2 | Q14151 |  |  | 1.30 |
| 3876 | CTGE5_HUMAN | cTAGE family member 5 | O15320 |  |  | 0.98 |
| 3877 | SYNCI_HUMAN | Syncoilin | Q9H7C4 |  |  |  |
| 3878 | KI13B_HUMAN | Kinesin-like protein KIF13B | Q9NQT8 |  |  | 0.25 |
| 3879 | FKB15_HUMAN | FK506-binding protein 15 | Q5T1M5 |  |  | 1.04 |
| 3880 | BMP2K_HUMAN | BMP-2-inducible protein kinase | Q9NSY1 |  |  | 1.19 |
| 3881 | CCDC8_HUMAN | Coiled-coil domain-containing protein 8 | Q9H0W5 |  |  | 0.69 |
| 3882 | ADCK5_HUMAN | Uncharacterized aarF domain-containing protein kinase 5 | Q3MIX3 |  |  | 1.70 |
| 3883 | ATLA2_HUMAN | Atlastin-2 | Q8NHH9 |  |  | 0.59 |
| 3884 | TMF1_HUMAN | TATA element modulatory factor | P82094 |  |  | 0.43 |
| 3885 | SI1L2_HUMAN | Signal-induced proliferation-associated 1-like protein 2 | Q9P2F8 |  |  | 0.83 |
| 3886 | S23IP_HUMAN | SEC23-interacting protein | Q9Y6Y8 |  |  |  |
| 3887 | TRAF6_HUMAN | TNF receptor-associated factor 6 | Q9Y4K3 |  |  | 2.00 |
| 3888 | RETST_HUMAN | All-trans-retinol 13,14-reductase | Q6NUM9 |  |  | 0.79 |
| 3889 | GAS7_HUMAN | Growth arrest-specific protein 7 | O60861 |  |  | 0.67 |
| 3890 | UB2G1_HUMAN | Ubiquitin-conjugating enzyme E2 G1 | P62253 |  |  | 1.04 |
| 3891 | HMGN3_HUMAN | High mobility group nucleosome-binding domain-containing protein 3 | Q15651 |  |  | 1.29 |
| 3892 | TBC15_HUMAN | TBC1 domain family member 15 | Q8TC07 |  |  | 0.78 |
| 3893 | PRP31_HUMAN | U4/U6 small nuclear ribonucleoprotein Prp31 | Q8WWY3 |  |  | 0.70 |
| 3894 | YMEL1_HUMAN | ATP-dependent zinc metalloprotease YME1L1 | Q96TA2 |  |  | 1.02 |
| 3895 | MET17_HUMAN | Methyltransferase-like protein 17, mitochondrial | Q9H7H0 |  |  | 0.37 |
| 3896 | SCYL2_HUMAN | SCY1-like protein 2 | Q6P3W7 |  |  | 1.16 |
| 3897 | WHRN_HUMAN | Whirlin | Q9P202 |  |  | 3.23 |
| 3898 | UVRAG_HUMAN | UV radiation resistance-associated gene protein | Q9P2Y5 |  |  |  |
| 3899 | DYR1A_HUMAN | Dual specificity tyrosine-phosphorylation-regulated kinase 1A | Q13627 |  |  |  |
| 3900 | SLX4I_HUMAN | Protein SLX4IP | Q5VYV7 |  |  | 0.81 |
| 3901 | SQSTM_HUMAN | Sequestosome-1 | Q13501 |  |  | 0.81 |
| 3902 | WIPI2_HUMAN | WD repeat domain phosphoinositide-interacting protein 2 | Q9Y4P8 |  |  | 0.78 |
| 3903 | SNX16_HUMAN | Sorting nexin-16 | P57768 |  |  | 0.29 |
| 3904 | FBXL8_HUMAN | F-box/LRR-repeat protein 8 | Q96CD0 |  |  | 0.73 |
| 3905 | NAA50_HUMAN | N-alpha-acetyltransferase 50 | Q9GZZ1 |  |  |  |
| 3906 | KDM1A_HUMAN | Lysine-specific histone demethylase 1A | O60341 |  |  | 1.80 |
| 3907 | IMPA3_HUMAN | Inositol monophosphatase 3 | Q9NX62 |  |  |  |
| 3908 | EXOS4_HUMAN | Exosome complex component RRP41 | Q9NPD3 |  |  | 0.93 |
| 3909 | STK39_HUMAN | STE20/SPS1-related proline-alanine-rich protein kinase | Q9UEW8 |  |  | 0.78 |
| 3910 | GBP2_HUMAN | Guanylate-binding protein 2 | P32456 |  |  | 1.10 |
| 3911 | LS14A_HUMAN | Protein LSM14 homolog A | Q8ND56 |  |  | 2.16 |
| 3912 | HABP2_HUMAN | Hyaluronan-binding protein 2 | Q14520 |  |  | 1.25 |
| 3913 | DNJB2_HUMAN | DnaJ homolog subfamily B member 2 | P25686 |  |  | 1.03 |
| 3914 | WBP11_HUMAN | WW domain-binding protein 11 | Q9Y2W2 |  |  | 0.90 |
| 3915 | ZCCHV_HUMAN | Zinc finger CCCH-type antiviral protein 1 | Q7Z2W4 |  |  | 0.99 |
| 3916 | T2FA_HUMAN | General transcription factor IIF subunit 1 | P35269 |  |  | 0.86 |
| 3917 | EHBP1_HUMAN | EH domain-binding protein 1 | Q8NDI1 |  |  |  |
| 3918 | PRDC1_HUMAN | Phosphoribosyltransferase domain-containing protein 1 | Q9NRG1 |  |  | 2.95 |
| 3919 | KI21A_HUMAN | Kinesin-like protein KIF21A | Q7Z4S6 |  |  |  |
| 3920 | IF4E2_HUMAN | Eukaryotic translation initiation factor 4E type 2 | O60573 |  |  | 0.76 |
| 3921 | SMRD2_HUMAN | SWI/SNF-related matrix-associated actin-dependent regulator of chromatin subfamily D member 2 | Q92925 |  |  | 0.78 |
| 3922 | GBG10_HUMAN | Guanine nucleotide-binding protein G(I)/G(S)/G(O) subunit gamma-10 | P50151 |  |  | 0.54 |
| 3923 | LYRM9_HUMAN | LYR motif-containing protein 9 | A8MSI8 |  |  | 0.01 |
| 3924 | S41A3_HUMAN | Solute carrier family 41 member 3 | Q96GZ6 |  |  | 0.55 |
| 3925 | S12A2_HUMAN | Solute carrier family 12 member 2 | P55011 |  |  | 0.88 |
| 3926 | RF1ML_HUMAN | Peptide chain release factor 1-like, mitochondrial | Q9UGC7 |  |  |  |
| 3927 | EAA1_HUMAN | Excitatory amino acid transporter 1 | P43003 |  |  |  |
| 3928 | TM192_HUMAN | Transmembrane protein 192 | Q8IY95 |  |  | 1.14 |
| 3929 | OSGP2_HUMAN | Probable tRNA N6-adenosine threonylcarbamoyltransferase, mitochondrial | Q9H4B0 |  |  |  |
| 3930 | IKKA_HUMAN | Inhibitor of nuclear factor kappa-B kinase subunit alpha | O15111 |  |  | 0.98 |
| 3931 | CCD96_HUMAN | Coiled-coil domain-containing protein 96 | Q2M329 |  |  | 0.86 |
| 3932 | DPOG2_HUMAN | DNA polymerase subunit gamma-2, mitochondrial | Q9UHN1 |  |  | 0.90 |
| 3933 | CHM4A_HUMAN | Charged multivesicular body protein 4a | Q9BY43 |  |  | 1.00 |
| 3934 | ITB3_HUMAN | Integrin beta-3 | P05106 |  |  | 0.90 |
| 3935 | TRI47_HUMAN | Tripartite motif-containing protein 47 | Q96LD4 |  |  | 0.96 |
| 3936 | BOREA_HUMAN | Borealin | Q53HL2 |  |  | 0.67 |
| 3937 | PPM1G_HUMAN | Protein phosphatase 1G | O15355 |  |  | 1.11 |
| 3938 | PAF1_HUMAN | RNA polymerase II-associated factor 1 homolog | Q8N7H5 |  |  |  |
| 3939 | ZN512_HUMAN | Zinc finger protein 512 | Q96ME7 |  |  | 1.86 |
| 3940 | NUMBL_HUMAN | Numb-like protein | Q9Y6R0 |  |  | 0.55 |
| 3941 | HYCCI_HUMAN | Hyccin | Q9BYI3 |  |  | 0.72 |
| 3942 | SPF30_HUMAN | Survival of motor neuron-related-splicing factor 30 | O75940 |  |  | 1.22 |
| 3943 | NFYC_HUMAN | Nuclear transcription factor Y subunit gamma | Q13952 |  |  | 0.33 |
| 3944 | HRSL3_HUMAN | HRAS-like suppressor 3 | P53816 |  |  | 1.12 |
| 3945 | TBC9B_HUMAN | TBC1 domain family member 9B | Q66K14 |  |  | 0.87 |
| 3946 | AGAP2_HUMAN | Arf-GAP with GTPase, ANK repeat and PH domain-containing protein 2 | Q99490 |  |  | 0.83 |
| 3947 | GPAT1_HUMAN | Glycerol-3-phosphate acyltransferase 1, mitochondrial | Q9HCL2 |  |  |  |
| 3948 | HID1_HUMAN | Protein HID1 | Q8IV36 |  |  | 0.82 |
| 3949 | PHLD_HUMAN | Phosphatidylinositol-glycan-specific phospholipase D | P80108 |  |  | 0.76 |
| 3950 | WDR18_HUMAN | WD repeat-containing protein 18 | Q9BV38 |  |  | 1.28 |
| 3951 | OGFR_HUMAN | Opioid growth factor receptor | Q9NZT2 |  |  | 0.40 |
| 3952 | ADA10_HUMAN | Disintegrin and metalloproteinase domain-containing protein 10 | O14672 |  |  | 3.84 |
| 3953 | ATL4_HUMAN | ADAMTS-like protein 4 | Q6UY14 |  |  | 2.00 |
| 3954 | RAF1_HUMAN | RAF proto-oncogene serine/threonine-protein kinase | P04049 |  |  | 0.73 |
| 3955 | SC5A1_HUMAN | Sodium/glucose cotransporter 1 | P13866 |  |  | 0.55 |
| 3956 | HYPK_HUMAN | Huntingtin-interacting protein K | Q9NX55 |  |  | 0.93 |
| 3957 | TM38B_HUMAN | Trimeric intracellular cation channel type B | Q9NVV0 |  |  | 0.59 |
| 3958 | ADXL_HUMAN | Adrenodoxin-like protein, mitochondrial | Q6P4F2 |  |  | 0.80 |
| 3959 | RM18_HUMAN | 39S ribosomal protein L18, mitochondrial | Q9H0U6 |  |  | 0.72 |
| 3960 | P5I11_HUMAN | Tumor protein p53-inducible protein 11 | O14683 |  |  | 1.22 |
| 3961 | IFT25_HUMAN | Intraflagellar transport protein 25 homolog | Q9Y547 |  |  | 0.75 |
| 3962 | KISHA_HUMAN | Protein kish-A | Q8TBQ9 |  |  | 0.99 |
| 3963 | SPTN2_HUMAN | Spectrin beta chain, non-erythrocytic 2 | O15020 |  |  | 0.83 |
| 3964 | A1ATR_HUMAN | Putative alpha-1-antitrypsin-related protein | P20848 |  |  | 1.89 |
| 3965 | CC180_HUMAN | Coiled-coil domain-containing protein 180 | Q9P1Z9 |  |  | 1.04 |
| 3966 | LV102_HUMAN | Ig lambda chain V-I region HA | P01700 |  |  |  |
| 3967 | RHBL4_HUMAN | Rhomboid-related protein 4 | Q8TEB9 |  |  | 1.00 |
| 3968 | DCNL2_HUMAN | DCN1-like protein 2 | Q6PH85 |  |  | 1.43 |
| 3969 | A16L1_HUMAN | Autophagy-related protein 16-1 | Q676U5 |  |  | 0.32 |
| 3970 | ESPL1_HUMAN | Separin | Q14674 |  |  | 0.27 |
| 3971 | NLRC4_HUMAN | NLR family CARD domain-containing protein 4 | Q9NPP4 |  |  | 1.22 |
| 3972 | TMED1_HUMAN | Transmembrane emp24 domain-containing protein 1 | Q13445 |  |  | 0.98 |
| 3973 | TREM1_HUMAN | Triggering receptor expressed on myeloid cells 1 | Q9NP99 |  |  |  |
| 3974 | MZT2B_HUMAN | Mitotic-spindle organizing protein 2B | Q6NZ67 |  |  |  |
| 3975 | TCEA3_HUMAN | Transcription elongation factor A protein 3 | O75764 |  |  | 1.64 |
| 3976 | TNR6_HUMAN | Tumor necrosis factor receptor superfamily member 6 | P25445 |  |  | 0.71 |
| 3977 | CP20A_HUMAN | Cytochrome P450 20A1 | Q6UW02 |  |  | 0.91 |
| 3978 | FA63B_HUMAN | Protein FAM63B | Q8NBR6 |  |  | 1.76 |
| 3979 | YIPF5_HUMAN | Protein YIPF5 | Q969M3 |  |  | 0.80 |
| 3980 | APBB1_HUMAN | Amyloid beta A4 precursor protein-binding family B member 1 | O00213 |  |  | 1.72 |
| 3981 | SYCP3_HUMAN | Synaptonemal complex protein 3 | Q8IZU3 |  |  | 0.61 |
| 3982 | UBP11_HUMAN | Ubiquitin carboxyl-terminal hydrolase 11 | P51784 |  |  | 1.02 |
| 3983 | PROC_HUMAN | Vitamin K-dependent protein C | P04070 |  |  | 1.74 |
| 3984 | BMP15_HUMAN | Bone morphogenetic protein 15 | O95972 |  |  | 0.44 |
| 3985 | OXR1_HUMAN | Oxidation resistance protein 1 | Q8N573 |  |  | 1.31 |
| 3986 | DHX32_HUMAN | Putative pre-mRNA-splicing factor ATP-dependent RNA helicase DHX32 | Q7L7V1 |  |  | 1.28 |
| 3987 | CWC15_HUMAN | Spliceosome-associated protein CWC15 homolog | Q9P013 |  |  |  |
| 3988 | EMRE_HUMAN | Essential MCU regulator, mitochondrial | Q9H4I9 |  |  |  |
| 3989 | TB10B_HUMAN | TBC1 domain family member 10B | Q4KMP7 |  |  | 1.07 |
| 3990 | MOT8_HUMAN | Monocarboxylate transporter 8 | P36021 |  |  | 1.26 |
| 3991 | T106B_HUMAN | Transmembrane protein 106B | Q9NUM4 |  |  |  |
| 3992 | SRSF9_HUMAN | Serine/arginine-rich splicing factor 9 | Q13242 |  |  | 0.79 |
| 3993 | WBS16_HUMAN | Williams-Beuren syndrome chromosomal region 16 protein | Q96I51 |  |  | 0.89 |
| 3994 | AP1S2_HUMAN | AP-1 complex subunit sigma-2 | P56377 |  |  | 1.26 |
| 3995 | CE290_HUMAN | Centrosomal protein of 290 kDa | O15078 |  |  | 2.64 |
| 3996 | ADA28_HUMAN | Disintegrin and metalloproteinase domain-containing protein 28 | Q9UKQ2 |  |  | 0.75 |
| 3997 | GLSL_HUMAN | Glutaminase liver isoform, mitochondrial | Q9UI32 |  |  | 0.70 |
| 3998 | STXB6_HUMAN | Syntaxin-binding protein 6 | Q8NFX7 |  |  |  |
| 3999 | NUP53_HUMAN | Nucleoporin NUP53 | Q8NFH5 |  |  | 0.69 |
| 4000 | AP1S1_HUMAN | AP-1 complex subunit sigma-1A | P61966 |  |  | 0.73 |
| 4001 | CE295_HUMAN | Centrosomal protein of 295 kDa | Q9C0D2 |  |  | 9.09 |
| 4002 | SHRM2_HUMAN | Protein Shroom2 | Q13796 |  |  | 1.01 |
| 4003 | CD37L_HUMAN | Hsp90 co-chaperone Cdc37-like 1 | Q7L3B6 |  |  | 0.57 |
| 4004 | SPAG7_HUMAN | Sperm-associated antigen 7 | O75391 |  |  |  |
| 4005 | CE051_HUMAN | UPF0600 protein C5orf51 | A6NDU8 |  |  | 1.11 |
| 4006 | GPC5B_HUMAN | G-protein coupled receptor family C group 5 member B | Q9NZH0 |  |  | 1.04 |
| 4007 | HUMMR_HUMAN | Protein MGARP | Q8TDB4 |  |  |  |
| 4008 | ARIP4_HUMAN | Helicase ARIP4 | Q9Y4B4 |  |  |  |
| 4009 | Z804A_HUMAN | Zinc finger protein 804A | Q7Z570 |  |  | 0.91 |
| 4010 | RAPH1_HUMAN | Ras-associated and pleckstrin homology domains-containing protein 1 | Q70E73 |  |  | 0.69 |
| 4011 | OGFD2_HUMAN | 2-oxoglutarate and iron-dependent oxygenase domain-containing protein 2 | Q6N063 |  |  | 1.12 |
| 4012 | PSMG1_HUMAN | Proteasome assembly chaperone 1 | O95456 |  |  | 0.77 |
| 4013 | CDN1B_HUMAN | Cyclin-dependent kinase inhibitor 1B | P46527 |  |  | 0.59 |
| 4014 | SZRD1_HUMAN | SUZ domain-containing protein 1 | Q7Z422 |  |  | 0.92 |
| 4015 | BRD4_HUMAN | Bromodomain-containing protein 4 | O60885 |  |  | 1.41 |
| 4016 | CHERP_HUMAN | Calcium homeostasis endoplasmic reticulum protein | Q8IWX8 |  |  | 1.06 |
| 4017 | GLYR1_HUMAN | Putative oxidoreductase GLYR1 | Q49A26 |  |  | 0.77 |
| 4018 | DDX28_HUMAN | Probable ATP-dependent RNA helicase DDX28 | Q9NUL7 |  |  | 1.34 |
| 4019 | ACM2_HUMAN | Muscarinic acetylcholine receptor M2 | P08172 |  |  |  |
| 4020 | TLDC1_HUMAN | TLD domain-containing protein 1 | Q6P9B6 |  |  | 0.58 |
| 4021 | SRR_HUMAN | Serine racemase | Q9GZT4 |  |  | 1.70 |
| 4022 | K1143_HUMAN | Uncharacterized protein KIAA1143 | Q96AT1 |  |  | 0.55 |
| 4023 | LFA3_HUMAN | Lymphocyte function-associated antigen 3 | P19256 |  |  | 1.00 |
| 4024 | HMGN4_HUMAN | High mobility group nucleosome-binding domain-containing protein 4 | O00479 |  |  | 0.42 |
| 4025 | SBSN_HUMAN | Suprabasin | Q6UWP8 |  |  | 1.10 |
| 4026 | CHCH1_HUMAN | Coiled-coil-helix-coiled-coil-helix domain-containing protein 1 | Q96BP2 |  |  | 1.19 |
| 4027 | IF4E3_HUMAN | Eukaryotic translation initiation factor 4E type 3 | Q8N5X7 |  |  | 0.61 |
| 4028 | PRTN3_HUMAN | Myeloblastin | P24158 |  |  |  |
| 4029 | RUXF_HUMAN | Small nuclear ribonucleoprotein F | P62306 |  |  | 0.71 |
| 4030 | SMIM4_HUMAN | Small integral membrane protein 4 | Q8WVI0 |  |  | 0.40 |
| 4031 | PRAF2_HUMAN | PRA1 family protein 2 | O60831 |  |  | 0.55 |
| 4032 | FIP1_HUMAN | Pre-mRNA 3'-end-processing factor FIP1 | Q6UN15 |  |  | 0.86 |
| 4033 | KITM_HUMAN | Thymidine kinase 2, mitochondrial | O00142 |  |  |  |
| 4034 | CSMT1_HUMAN | Protein CCSMST1 | Q4G0I0 |  |  | 1.21 |
| 4035 | TIM22_HUMAN | Mitochondrial import inner membrane translocase subunit Tim22 | Q9Y584 |  |  |  |
| 4036 | RARR2_HUMAN | Retinoic acid receptor responder protein 2 | Q99969 |  |  | 0.40 |
| 4037 | VMA21_HUMAN | Vacuolar ATPase assembly integral membrane protein VMA21 | Q3ZAQ7 |  |  | 1.20 |
| 4038 | CY24A_HUMAN | Cytochrome b-245 light chain | P13498 |  |  | 0.54 |
| 4039 | RM34_HUMAN | 39S ribosomal protein L34, mitochondrial | Q9BQ48 |  |  | 0.63 |
| 4040 | TRIA1_HUMAN | TP53-regulated inhibitor of apoptosis 1 | O43715 |  |  | 0.62 |
| 4041 | CN142_HUMAN | Uncharacterized protein C14orf142 | Q9BXV9 |  |  | 1.34 |
| 4042 | PIOS1_HUMAN | Protein PIGBOS1 | A0A0B4J2F0 |  |  | 1.57 |
| 4043 | PR40A_HUMAN | Pre-mRNA-processing factor 40 homolog A | O75400 |  |  | 1.00 |
| 4044 | SCG1_HUMAN | Secretogranin-1 | P05060 |  |  | 1.32 |
| 4045 | PISD_HUMAN | Phosphatidylserine decarboxylase proenzyme | Q9UG56 |  |  | 0.65 |
| 4046 | GMPR2_HUMAN | GMP reductase 2 | Q9P2T1 |  |  | 0.76 |
| 4047 | ERAP2_HUMAN | Endoplasmic reticulum aminopeptidase 2 | Q6P179 |  |  | 1.42 |
| 4048 | TPRGL_HUMAN | Tumor protein p63-regulated gene 1-like protein | Q5T0D9 |  |  | 0.90 |
| 4049 | WASL_HUMAN | Neural Wiskott-Aldrich syndrome protein | O00401 |  |  | 0.71 |
| 4050 | RT63_HUMAN | Ribosomal protein 63, mitochondrial | Q9BQC6 |  |  | 0.89 |
| 4051 | PLPL6_HUMAN | Neuropathy target esterase | Q8IY17 |  |  |  |
| 4052 | CAD19_HUMAN | Cadherin-19 | Q9H159 |  |  | 1.16 |
| 4053 | IBP5_HUMAN | Insulin-like growth factor-binding protein 5 | P24593 |  |  |  |
| 4054 | PEX14_HUMAN | Peroxisomal membrane protein PEX14 | O75381 |  |  |  |
| 4055 | UBCP1_HUMAN | Ubiquitin-like domain-containing CTD phosphatase 1 | Q8WVY7 |  |  |  |
| 4056 | PAPD1_HUMAN | Poly(A) RNA polymerase, mitochondrial | Q9NVV4 |  |  |  |
| 4057 | RAB8A_HUMAN | Ras-related protein Rab-8A | P61006 |  |  | 0.69 |
| 4058 | L2GL1_HUMAN | Lethal(2) giant larvae protein homolog 1 | Q15334 |  |  |  |
| 4059 | ARL15_HUMAN | ADP-ribosylation factor-like protein 15 | Q9NXU5 |  |  | 0.85 |
| 4060 | ARSB_HUMAN | Arylsulfatase B | P15848 |  |  | 0.93 |
| 4061 | OAS3_HUMAN | 2'-5'-oligoadenylate synthase 3 | Q9Y6K5 |  |  | 0.51 |
| 4062 | UBFL1_HUMAN | Upstream-binding factor 1-like protein 1 | P0CB47 |  |  | 1.04 |
| 4063 | H13_HUMAN | Histone H1.3 | P16402 |  |  | 0.53 |
| 4064 | BIRC6_HUMAN | Baculoviral IAP repeat-containing protein 6 | Q9NR09 |  |  | 0.68 |
| 4065 | PDZD2_HUMAN | PDZ domain-containing protein 2 | O15018 |  |  | 0.88 |
| 4066 | LAMA3_HUMAN | Laminin subunit alpha-3 | Q16787 |  |  | 1.03 |
| 4067 | TM186_HUMAN | Transmembrane protein 186 | Q96B77 |  |  | 0.32 |
| 4068 | ETUD1_HUMAN | Elongation factor Tu GTP-binding domain-containing protein 1 | Q7Z2Z2 |  |  | 0.80 |
| 4069 | TBC13_HUMAN | TBC1 domain family member 13 | Q9NVG8 |  |  | 1.02 |
| 4070 | FUCO2_HUMAN | Plasma alpha-L-fucosidase | Q9BTY2 |  |  | 1.06 |
| 4071 | PIGU_HUMAN | Phosphatidylinositol glycan anchor biosynthesis class U protein | Q9H490 |  |  | 0.93 |
| 4072 | NECP1_HUMAN | Adaptin ear-binding coat-associated protein 1 | Q8NC96 |  |  | 1.40 |
| 4073 | RAB4B_HUMAN | Ras-related protein Rab-4B | P61018 |  |  | 0.97 |
| 4074 | SUCHY_HUMAN | Succinate--hydroxymethylglutarate CoA-transferase | Q9HAC7 |  |  | 1.28 |
| 4075 | DJC21_HUMAN | DnaJ homolog subfamily C member 21 | Q5F1R6 |  |  | 2.24 |
| 4076 | ACBD6_HUMAN | Acyl-CoA-binding domain-containing protein 6 | Q9BR61 |  |  | 0.63 |
| 4077 | FRMD3_HUMAN | FERM domain-containing protein 3 | A2A2Y4 |  |  |  |
| 4078 | HEM4_HUMAN | Uroporphyrinogen-III synthase | P10746 |  |  | 0.88 |
| 4079 | AHSP_HUMAN | Alpha-hemoglobin-stabilizing protein | Q9NZD4 |  |  | 0.66 |
| 4080 | DPTOR_HUMAN | DEP domain-containing mTOR-interacting protein | Q8TB45 |  |  | 0.99 |
| 4081 | CXCL7_HUMAN | Platelet basic protein | P02775 |  |  | 0.78 |
| 4082 | FA26E_HUMAN | Protein FAM26E | Q8N5C1 |  |  | 0.82 |
| 4083 | FABPL_HUMAN | Fatty acid-binding protein, liver | P07148 |  |  | 0.40 |
| 4084 | TM160_HUMAN | Transmembrane protein 160 | Q9NX00 |  |  |  |
| 4085 | CREB1_HUMAN | Cyclic AMP-responsive element-binding protein 1 | P16220 |  |  |  |
| 4086 | WASH6_HUMAN | WAS protein family homolog 6 | Q9NQA3 |  |  |  |
| 4087 | YJ005_HUMAN | Uncharacterized protein FLJ45252 | Q6ZSR9 |  |  | 0.92 |
| 4088 | K1C28_HUMAN | Keratin, type I cytoskeletal 28 | Q7Z3Y7 |  |  | 0.91 |
| 4089 | FADD_HUMAN | FAS-associated death domain protein | Q13158 |  |  | 0.69 |
| 4090 | DNS2A_HUMAN | Deoxyribonuclease-2-alpha | O00115 |  |  | 0.74 |
| 4091 | F185A_HUMAN | Protein FAM185A | Q8N0U4 |  |  | 0.91 |
| 4092 | LV001_HUMAN | Ig lambda chain V region 4A | P04211 |  |  | 0.92 |
| 4093 | ARHG2_HUMAN | Rho guanine nucleotide exchange factor 2 | Q92974 |  |  | 0.83 |
| 4094 | ELP1_HUMAN | Elongator complex protein 1 | O95163 |  |  | 0.78 |
| 4095 | ILKAP_HUMAN | Integrin-linked kinase-associated serine/threonine phosphatase 2C | Q9H0C8 |  |  | 1.25 |
| 4096 | CHD4_HUMAN | Chromodomain-helicase-DNA-binding protein 4 | Q14839 |  |  | 0.63 |
| 4097 | SIA4A_HUMAN | CMP-N-acetylneuraminate-beta-galactosamide-alpha-2,3-sialyltransferase 1 | Q11201 |  |  | 0.77 |
| 4098 | TRAK1_HUMAN | Trafficking kinesin-binding protein 1 | Q9UPV9 |  |  | 0.49 |
| 4099 | TBB4A_HUMAN | Tubulin beta-4A chain | P04350 |  |  |  |
| 4100 | KDIS_HUMAN | Kinase D-interacting substrate of 220 kDa | Q9ULH0 |  |  | 0.90 |
| 4101 | ALDOB_HUMAN | Fructose-bisphosphate aldolase B | P05062 |  |  | 0.91 |
| 4102 | KBTBB_HUMAN | Kelch repeat and BTB domain-containing protein 11 | O94819 |  |  | 0.80 |
| 4103 | TANC1_HUMAN | Protein TANC1 | Q9C0D5 |  |  |  |
| 4104 | HV206_HUMAN | Ig heavy chain V-II region WAH | P01824 |  |  | 0.57 |
| 4105 | ABCB6_HUMAN | ATP-binding cassette sub-family B member 6, mitochondrial | Q9NP58 |  |  | 0.70 |
| 4106 | ERLEC_HUMAN | Endoplasmic reticulum lectin 1 | Q96DZ1 |  |  |  |
| 4107 | PDCL3_HUMAN | Phosducin-like protein 3 | Q9H2J4 |  |  | 1.10 |
| 4108 | ARBK1_HUMAN | Beta-adrenergic receptor kinase 1 | P25098 |  |  | 0.65 |
| 4109 | TM55A_HUMAN | Type 2 phosphatidylinositol 4,5-bisphosphate 4-phosphatase | Q8N4L2 |  |  | 0.92 |
| 4110 | MEF2A_HUMAN | Myocyte-specific enhancer factor 2A | Q02078 |  |  | 1.07 |
| 4111 | MEF2C_HUMAN | Myocyte-specific enhancer factor 2C | Q06413 |  |  |  |
| 4112 | STABP_HUMAN | STAM-binding protein | O95630 |  |  | 1.32 |
| 4113 | HNRLL_HUMAN | Heterogeneous nuclear ribonucleoprotein L-like | Q8WVV9 |  |  |  |
| 4114 | MYPR_HUMAN | Myelin proteolipid protein | P60201 |  |  | 0.61 |
| 4115 | TPPC5_HUMAN | Trafficking protein particle complex subunit 5 | Q8IUR0 |  |  |  |
| 4116 | TOM5_HUMAN | Mitochondrial import receptor subunit TOM5 homolog | Q8N4H5 |  |  | 0.80 |
| 4117 | NMD3_HUMAN | 60S ribosomal export protein NMD3 | Q96D46 |  |  | 0.86 |
| 4118 | CD38_HUMAN | ADP-ribosyl cyclase/cyclic ADP-ribose hydrolase 1 | P28907 |  |  | 0.50 |
| 4119 | SL9A9_HUMAN | Sodium/hydrogen exchanger 9 | Q8IVB4 |  |  |  |
| 4120 | CCD91_HUMAN | Coiled-coil domain-containing protein 91 | Q7Z6B0 |  |  | 1.64 |
| 4121 | MLF2_HUMAN | Myeloid leukemia factor 2 | Q15773 |  |  | 1.31 |
| 4122 | KLH40_HUMAN | Kelch-like protein 40 | Q2TBA0 |  |  |  |
| 4123 | CATF_HUMAN | Cathepsin F | Q9UBX1 |  |  | 0.96 |
| 4124 | SCN2B_HUMAN | Sodium channel subunit beta-2 | O60939 |  |  | 0.62 |
| 4125 | RISC_HUMAN | Retinoid-inducible serine carboxypeptidase | Q9HB40 |  |  | 0.70 |
| 4126 | RENBP_HUMAN | N-acylglucosamine 2-epimerase | P51606 |  |  | 0.82 |
| 4127 | CROCC_HUMAN | Rootletin | Q5TZA2 |  |  | 1.51 |
| 4128 | CROL2_HUMAN | Putative ciliary rootlet coiled-coil protein-like 2 protein | Q8IVE0 |  |  | 1.33 |
| 4129 | SPS2_HUMAN | Selenide, water dikinase 2 | Q99611 |  |  | 0.48 |
| 4130 | TPPC9_HUMAN | Trafficking protein particle complex subunit 9 | Q96Q05 |  |  | 0.99 |
| 4131 | RAI14_HUMAN | Ankycorbin | Q9P0K7 |  |  | 0.88 |
| 4132 | UBE2Z_HUMAN | Ubiquitin-conjugating enzyme E2 Z | Q9H832 |  |  | 0.69 |
| 4133 | CTL2_HUMAN | Choline transporter-like protein 2 | Q8IWA5 |  |  | 0.80 |
| 4134 | BORG5_HUMAN | Cdc42 effector protein 1 | Q00587 |  |  | 1.19 |
| 4135 | TRM11_HUMAN | tRNA (guanine(10)-N2)-methyltransferase homolog | Q7Z4G4 |  |  | 1.76 |
| 4136 | TM230_HUMAN | Transmembrane protein 230 | Q96A57 |  |  |  |
| 4137 | HABP4_HUMAN | Intracellular hyaluronan-binding protein 4 | Q5JVS0 |  |  |  |
| 4138 | FETUB_HUMAN | Fetuin-B | Q9UGM5 |  |  |  |
| 4139 | KV116_HUMAN | Ig kappa chain V-I region Roy | P01608 |  |  | 0.98 |
| 4140 | TXND9_HUMAN | Thioredoxin domain-containing protein 9 | O14530 |  |  | 1.03 |
| 4141 | MGT5A_HUMAN | Alpha-1,6-mannosylglycoprotein 6-beta-N-acetylglucosaminyltransferase A | Q09328 |  |  | 0.82 |
| 4142 | SELH_HUMAN | Selenoprotein H | Q8IZQ5 |  |  | 1.57 |
| 4143 | CSK22_HUMAN | Casein kinase II subunit alpha' | P19784 |  |  | 1.07 |
| 4144 | FUK_HUMAN | L-fucose kinase | Q8N0W3 |  |  | 1.12 |
| 4145 | OR6N2_HUMAN | Olfactory receptor 6N2 | Q8NGY6 |  |  |  |
| 4146 | PGFRB_HUMAN | Platelet-derived growth factor receptor beta | P09619 |  |  | 0.58 |
| 4147 | TMED5_HUMAN | Transmembrane emp24 domain-containing protein 5 | Q9Y3A6 |  |  |  |
| 4148 | NAA20_HUMAN | N-alpha-acetyltransferase 20 | P61599 |  |  |  |
| 4149 | DSCR3_HUMAN | Down syndrome critical region protein 3 | O14972 |  |  | 0.95 |
| 4150 | NOA1_HUMAN | Nitric oxide-associated protein 1 | Q8NC60 |  |  | 0.56 |
| 4151 | ARLY_HUMAN | Argininosuccinate lyase | P04424 |  |  | 0.60 |
| 4152 | FA45A_HUMAN | Protein FAM45A | Q8TCE6 |  |  | 2.03 |
| 4153 | SDSL_HUMAN | Serine dehydratase-like | Q96GA7 |  |  | 0.55 |
| 4154 | RANB3_HUMAN | Ran-binding protein 3 | Q9H6Z4 |  |  |  |
| 4155 | SPRY7_HUMAN | SPRY domain-containing protein 7 | Q5W111 |  |  | 1.04 |
| 4156 | TPSN_HUMAN | Tapasin | O15533 |  |  | 0.83 |
| 4157 | CORIN_HUMAN | Atrial natriuretic peptide-converting enzyme | Q9Y5Q5 |  |  | 2.35 |
| 4158 | NU160_HUMAN | Nuclear pore complex protein Nup160 | Q12769 |  |  | 0.32 |
| 4159 | 4EBP1_HUMAN | Eukaryotic translation initiation factor 4E-binding protein 1 | Q13541 |  |  | 1.09 |
| 4160 | MENTO_HUMAN | MLN64 N-terminal domain homolog | O95772 |  |  | 0.78 |
| 4161 | ABCBA_HUMAN | ATP-binding cassette sub-family B member 10, mitochondrial | Q9NRK6 |  |  | 0.88 |
| 4162 | TNIK_HUMAN | TRAF2 and NCK-interacting protein kinase | Q9UKE5 |  |  |  |
| 4163 | M4K4_HUMAN | Mitogen-activated protein kinase kinase kinase kinase 4 | O95819 |  |  | 0.66 |
| 4164 | PGPI_HUMAN | Pyroglutamyl-peptidase 1 | Q9NXJ5 |  |  |  |
| 4165 | HTAI2_HUMAN | Oxidoreductase HTATIP2 | Q9BUP3 |  |  |  |
| 4166 | UB2R1_HUMAN | Ubiquitin-conjugating enzyme E2 R1 | P49427 |  |  |  |
| 4167 | CIAO1_HUMAN | Probable cytosolic iron-sulfur protein assembly protein CIAO1 | O76071 |  |  |  |
| 4168 | CHPT1_HUMAN | Cholinephosphotransferase 1 | Q8WUD6 |  |  | 0.56 |
| 4169 | PDIA5_HUMAN | Protein disulfide-isomerase A5 | Q14554 |  |  | 1.66 |
| 4170 | GSAP_HUMAN | Gamma-secretase-activating protein | A4D1B5 |  |  |  |
| 4171 | SCO2_HUMAN | Protein SCO2 homolog, mitochondrial | O43819 |  |  |  |
| 4172 | ARRB1_HUMAN | Beta-arrestin-1 | P49407 |  |  |  |
| 4173 | AAKB1_HUMAN | 5'-AMP-activated protein kinase subunit beta-1 | Q9Y478 |  |  | 0.67 |
| 4174 | IL33_HUMAN | Interleukin-33 | O95760 |  |  | 0.48 |
| 4175 | MIC25_HUMAN | MICOS complex subunit MIC25 | Q9BRQ6 |  |  |  |
| 4176 | EI2BD_HUMAN | Translation initiation factor eIF-2B subunit delta | Q9UI10 |  |  | 1.25 |
| 4177 | SYAM_HUMAN | Alanine--tRNA ligase, mitochondrial | Q5JTZ9 |  |  | 0.73 |
| 4178 | F227A_HUMAN | Protein FAM227A | F5H4B4 |  |  | 0.74 |
| 4179 | LYVE1_HUMAN | Lymphatic vessel endothelial hyaluronic acid receptor 1 | Q9Y5Y7 |  |  | 0.94 |
| 4180 | MELT_HUMAN | Ventricular zone-expressed PH domain-containing protein homolog 1 | Q14D04 |  |  | 0.95 |
| 4181 | RPTOR_HUMAN | Regulatory-associated protein of mTOR | Q8N122 |  |  |  |
| 4182 | CETN2_HUMAN | Centrin-2 | P41208 |  |  | 0.81 |
| 4183 | TENS3_HUMAN | Tensin-3 | Q68CZ2 |  |  | 0.35 |
| 4184 | PX11B_HUMAN | Peroxisomal membrane protein 11B | O96011 |  |  |  |
| 4185 | GPC4_HUMAN | Glypican-4 | O75487 |  |  |  |
| 4186 | NEMF_HUMAN | Nuclear export mediator factor NEMF | O60524 |  |  |  |
| 4187 | BSDC1_HUMAN | BSD domain-containing protein 1 | Q9NW68 |  |  |  |
| 4188 | SMIM1_HUMAN | Small integral membrane protein 1 | B2RUZ4 |  |  |  |
| 4189 | ARX_HUMAN | Homeobox protein ARX | Q96QS3 |  |  | 2.48 |
| 4190 | SURF6_HUMAN | Surfeit locus protein 6 | O75683 |  |  |  |
| 4191 | PLOD3_HUMAN | Procollagen-lysine,2-oxoglutarate 5-dioxygenase 3 | O60568 |  |  | 1.91 |
| 4192 | F134B_HUMAN | Reticulophagy receptor FAM134B | Q9H6L5 |  |  | 1.08 |
| 4193 | PRP4B_HUMAN | Serine/threonine-protein kinase PRP4 homolog | Q13523 |  |  | 0.74 |
| 4194 | COXM2_HUMAN | COX assembly mitochondrial protein 2 homolog | Q9NRP2 |  |  |  |
| 4195 | NOXA1_HUMAN | NADPH oxidase activator 1 | Q86UR1 |  |  | 2.40 |
| 4196 | AT131_HUMAN | Manganese-transporting ATPase 13A1 | Q9HD20 |  |  | 1.80 |
| 4197 | NFIA_HUMAN | Nuclear factor 1 A-type | Q12857 |  |  | 1.19 |
| 4198 | NFIB_HUMAN | Nuclear factor 1 B-type | O00712 |  |  | 0.87 |
| 4199 | PKHH3_HUMAN | Pleckstrin homology domain-containing family H member 3 | Q7Z736 |  |  | 0.77 |
| 4200 | SRP09_HUMAN | Signal recognition particle 9 kDa protein | P49458 |  |  | 0.75 |
| 4201 | COMD7_HUMAN | COMM domain-containing protein 7 | Q86VX2 |  |  | 0.91 |
| 4202 | FOSL1_HUMAN | Fos-related antigen 1 | P15407 |  |  | 1.20 |
| 4203 | NCOA5_HUMAN | Nuclear receptor coactivator 5 | Q9HCD5 |  |  |  |
| 4204 | I2BPL_HUMAN | Interferon regulatory factor 2-binding protein-like | Q9H1B7 |  |  |  |
| 4205 | CCZ1_HUMAN | Vacuolar fusion protein CCZ1 homolog | P86791 |  |  |  |
| 4206 | ZRAB2_HUMAN | Zinc finger Ran-binding domain-containing protein 2 | O95218 |  |  | 1.49 |
| 4207 | PLS3_HUMAN | Phospholipid scramblase 3 | Q9NRY6 |  |  | 1.14 |
| 4208 | TOR1B_HUMAN | Torsin-1B | O14657 |  |  | 1.68 |
| 4209 | WLS_HUMAN | Protein wntless homolog | Q5T9L3 |  |  | 1.48 |
| 4210 | HOIL1_HUMAN | RanBP-type and C3HC4-type zinc finger-containing protein 1 | Q9BYM8 |  |  | 1.41 |
| 4211 | TAF4_HUMAN | Transcription initiation factor TFIID subunit 4 | O00268 |  |  |  |
| 4212 | SMYD5_HUMAN | SET and MYND domain-containing protein 5 | Q6GMV2 |  |  | 1.18 |
| 4213 | WIPI3_HUMAN | WD repeat domain phosphoinositide-interacting protein 3 | Q5MNZ6 |  |  |  |
| 4214 | COG5_HUMAN | Conserved oligomeric Golgi complex subunit 5 | Q9UP83 |  |  | 0.57 |
| 4215 | SHP1L_HUMAN | SHC SH2 domain-binding protein 1-like protein | Q9BZQ2 |  |  |  |
| 4216 | SNR27_HUMAN | U4/U6.U5 small nuclear ribonucleoprotein 27 kDa protein | Q8WVK2 |  |  | 0.78 |
| 4217 | PERM_HUMAN | Myeloperoxidase | P05164 |  |  | 0.82 |
| 4218 | LPIN1_HUMAN | Phosphatidate phosphatase LPIN1 | Q14693 |  |  | 0.99 |
| 4219 | MEX3A_HUMAN | RNA-binding protein MEX3A | A1L020 |  |  |  |
| 4220 | B2CL2_HUMAN | Bcl-2-like protein 2 | Q92843 |  |  |  |
| 4221 | MLF1_HUMAN | Myeloid leukemia factor 1 | P58340 |  |  | 1.20 |
| 4222 | HEM3_HUMAN | Porphobilinogen deaminase | P08397 |  |  | 0.74 |
| 4223 | RTF1_HUMAN | RNA polymerase-associated protein RTF1 homolog | Q92541 |  |  | 0.55 |
| 4224 | ABD12_HUMAN | Monoacylglycerol lipase ABHD12 | Q8N2K0 |  |  |  |
| 4225 | DIP2B_HUMAN | Disco-interacting protein 2 homolog B | Q9P265 |  |  |  |
| 4226 | CSRP2_HUMAN | Cysteine and glycine-rich protein 2 | Q16527 |  |  |  |
| 4227 | UBE4A_HUMAN | Ubiquitin conjugation factor E4 A | Q14139 |  |  |  |
| 4228 | LRRK2_HUMAN | Leucine-rich repeat serine/threonine-protein kinase 2 | Q5S007 |  |  | 0.71 |
| 4229 | CPN2_HUMAN | Carboxypeptidase N subunit 2 | P22792 |  |  |  |
| 4230 | NARFL_HUMAN | Cytosolic Fe-S cluster assembly factor NARFL | Q9H6Q4 |  |  | 0.97 |
| 4231 | NARF_HUMAN | Nuclear prelamin A recognition factor | Q9UHQ1 |  |  | 1.09 |
| 4232 | PPR3B_HUMAN | Protein phosphatase 1 regulatory subunit 3B | Q86XI6 |  |  | 0.91 |
| 4233 | AGO1_HUMAN | Protein argonaute-1 | Q9UL18 |  |  | 1.07 |
| 4234 | MYO5B_HUMAN | Unconventional myosin-Vb | Q9ULV0 |  |  | 0.67 |
| 4235 | RBY1D_HUMAN | RNA-binding motif protein, Y chromosome, family 1 member D | P0C7P1 |  |  |  |
| 4236 | PSME4_HUMAN | Proteasome activator complex subunit 4 | Q14997 |  |  | 1.62 |
| 4237 | SMAD5_HUMAN | Mothers against decapentaplegic homolog 5 | Q99717 |  |  |  |
| 4238 | ABHD2_HUMAN | Abhydrolase domain-containing protein 2 | P08910 |  |  |  |
| 4239 | PDPK1_HUMAN | 3-phosphoinositide-dependent protein kinase 1 | O15530 |  |  | 2.39 |
| 4240 | PDPK2_HUMAN | Putative 3-phosphoinositide-dependent protein kinase 2 | Q6A1A2 |  |  | 0.65 |
| 4241 | VP37B_HUMAN | Vacuolar protein sorting-associated protein 37B | Q9H9H4 |  |  | 0.82 |
| 4242 | AAMP_HUMAN | Angio-associated migratory cell protein | Q13685 |  |  | 0.94 |
| 4243 | ZDHC5_HUMAN | Palmitoyltransferase ZDHHC5 | Q9C0B5 |  |  | 1.05 |
| 4244 | KDM4C_HUMAN | Lysine-specific demethylase 4C | Q9H3R0 |  |  |  |
| 4245 | TICN1_HUMAN | Testican-1 | Q08629 |  |  |  |
| 4246 | RO52_HUMAN | E3 ubiquitin-protein ligase TRIM21 | P19474 |  |  |  |
| 4247 | NCOR2_HUMAN | Nuclear receptor corepressor 2 | Q9Y618 |  |  | 1.14 |
| 4248 | HV105_HUMAN | Ig heavy chain V-I region WOL | P01760 |  |  | 4.01 |
| 4249 | GLTD2_HUMAN | Glycolipid transfer protein domain-containing protein 2 | A6NH11 |  |  | 4.44 |
| 4250 | ZN275_HUMAN | Zinc finger protein 275 | Q9NSD4 |  |  | 1.63 |
| 4251 | NUP62_HUMAN | Nuclear pore glycoprotein p62 | P37198 |  |  | 0.96 |
| 4252 | NAGAB_HUMAN | Alpha-N-acetylgalactosaminidase | P17050 |  |  |  |
| 4253 | DECR2_HUMAN | Peroxisomal 2,4-dienoyl-CoA reductase | Q9NUI1 |  |  | 0.66 |
| 4254 | MGN2_HUMAN | Protein mago nashi homolog 2 | Q96A72 |  |  | 1.56 |
| 4255 | DHX30_HUMAN | Putative ATP-dependent RNA helicase DHX30 | Q7L2E3 |  |  | 0.69 |
| 4256 | CABL1_HUMAN | CDK5 and ABL1 enzyme substrate 1 | Q8TDN4 |  |  | 0.71 |
| 4257 | UBA5_HUMAN | Ubiquitin-like modifier-activating enzyme 5 | Q9GZZ9 |  |  | 0.86 |
| 4258 | DNJC8_HUMAN | DnaJ homolog subfamily C member 8 | O75937 |  |  | 0.15 |
| 4259 | 2B1D_HUMAN | HLA class II histocompatibility antigen, DRB1-13 beta chain | Q5Y7A7 |  |  |  |
| 4260 | ST1A1_HUMAN | Sulfotransferase 1A1 | P50225 |  |  | 0.95 |
| 4261 | COG6_HUMAN | Conserved oligomeric Golgi complex subunit 6 | Q9Y2V7 |  |  | 0.76 |
| 4262 | SMCO1_HUMAN | Single-pass membrane and coiled-coil domain-containing protein 1 | Q147U7 |  |  |  |
| 4263 | CELF2_HUMAN | CUGBP Elav-like family member 2 | O95319 |  |  | 1.13 |
