# Supplementary material for: In-depth proteomic profiling of left ventricular tissues in human end-stage dilated cardiomyopathy
Source: Oncotarget. 2017 Feb 25;8(29):48321–32. doi: 10.18632/oncotarget.15689 (PMC5564650; doi:10.18632/oncotarget.15689)
Supplement: Supplementary file 3 [file oncotarget-08-48321-s003.docx]

**Table S2.** **The differentially expressed proteins in end-stage DCM versus normal LV**

| **Number** | **ID** | **Description** | **Accession** | **Ratio 1** | **Ratio 2** | **Ratio 3** | **Average** | **p-value** |
| --- | --- | --- | --- | --- | --- | --- | --- | --- |
| 1 | 1433G_HUMAN | 14-3-3 protein gamma | P61981 | 0.60 | 0.68 | 0.91 | 0.73 | 0.05 |
| 2 | 68MP_HUMAN | 6.8 kDa mitochondrial proteolipid | P56378 | 0.79 | 0.74 | 0.68 | 0.74 | 0.00 |
| 3 | ABCD3_HUMAN | ATP-binding cassette sub-family D member 3 | P28288 | 1.19 |  | 1.28 | 1.24 | 0.01 |
| 4 | ADT3_HUMAN | ADP/ATP translocase 3 | P12236 | 0.19 | 0.74 | 0.45 | 0.46 | 0.03 |
| 5 | AIMP2_HUMAN | Aminoacyl tRNA synthase complex-interacting multifunctional protein 2 | Q13155 | 0.77 |  | 0.77 | 0.77 | 0.00 |
| 6 | AL7A1_HUMAN | Alpha-aminoadipic semialdehyde dehydrogenase | P49419 | 0.84 | 0.73 | 0.65 | 0.74 | 0.01 |
| 7 | APOC1_HUMAN | Apolipoprotein C-I | P02654 |  | 1.33 | 1.20 | 1.26 | 0.01 |
| 8 | ARVC_HUMAN | Armadillo repeat protein deleted in velo-cardio-facial syndrome | O00192 | 0.62 |  | 0.79 | 0.71 | 0.02 |
| 9 | ATAD1_HUMAN | ATPase family AAA domain-containing protein 1 | Q8NBU5 | 1.94 |  | 1.45 | 1.70 | 0.03 |
| 10 | BCAM_HUMAN | Basal cell adhesion molecule | P50895 | 0.64 | 0.84 | 0.87 | 0.78 | 0.04 |
| 11 | BCS1_HUMAN | Mitochondrial chaperone BCS1 | Q9Y276 | 0.80 | 0.69 | 0.93 | 0.81 | 0.05 |
| 12 | BID_HUMAN | BH3-interacting domain death agonist | P55957 | 1.49 | 1.94 | 1.58 | 1.67 | 0.01 |
| 13 | CA2D1_HUMAN | Voltage-dependent calcium channel subunit alpha-2/delta-1 | P54289 | 0.83 | 0.86 | 0.76 | 0.81 | 0.00 |
| 14 | CADH2_HUMAN | Cadherin-2 | P19022 | 0.61 |  | 0.80 | 0.71 | 0.02 |
| 15 | CALD1_HUMAN | Caldesmon | Q05682 | 1.24 | 1.33 | 1.07 | 1.21 | 0.05 |
| 16 | CAN2_HUMAN | Calpain-2 catalytic subunit | P17655 | 1.18 | 1.41 | 1.16 | 1.25 | 0.04 |
| 17 | CATL1_HUMAN | Cathepsin L1 | P07711 | 0.26 | 0.69 | 0.70 | 0.55 | 0.04 |
| 18 | CAV2_HUMAN | Caveolin-2 | P51636 | 0.51 | 0.59 |  | 0.55 | 0.00 |
| 19 | CCS_HUMAN | Copper chaperone for superoxide dismutase | O14618 | 2.73 | 2.91 |  | 2.82 | 0.00 |
| 20 | CD36_HUMAN | Platelet glycoprotein 4 | P16671 | 0.67 | 0.61 | 0.91 | 0.73 | 0.04 |
| 21 | CD9_HUMAN | CD9 antigen | P21926 | 0.50 | 0.82 | 0.74 | 0.69 | 0.03 |
| 22 | CHDH_HUMAN | Choline dehydrogenase, mitochondrial | Q8NE62 | 0.87 | 0.80 | 0.80 | 0.82 | 0.00 |
| 23 | CLIC5_HUMAN | Chloride intracellular channel protein 5 | Q9NZA1 | 0.72 |  | 0.86 | 0.79 | 0.03 |
| 24 | CNN3_HUMAN | Calponin-3 | Q15417 | 0.77 |  | 0.76 | 0.76 | 0.00 |
| 25 | COASY_HUMAN | Bifunctional coenzyme A synthase | Q13057 | 1.89 | 1.53 | 1.23 | 1.55 | 0.05 |
| 26 | COCA1_HUMAN | Collagen alpha-1(XII) chain | Q99715 | 0.61 | 0.67 | 0.90 | 0.73 | 0.04 |
| 27 | CRYAB_HUMAN | Alpha-crystallin B chain | P02511 |  | 0.67 | 0.48 | 0.57 | 0.01 |
| 28 | CSN8_HUMAN | COP9 signalosome complex subunit 8 | Q99627 | 0.72 | 0.75 | 0.69 | 0.72 | 0.00 |
| 29 | CTND1_HUMAN | Catenin delta-1 | O60716 | 0.69 | 0.78 | 0.78 | 0.75 | 0.00 |
| 30 | CUL1_HUMAN | Cullin-1 | Q13616 | 0.70 |  | 0.64 | 0.67 | 0.00 |
| 31 | CY1_HUMAN | Cytochrome c1, heme protein, mitochondrial | P08574 | 0.31 |  | 0.65 | 0.48 | 0.03 |
| 32 | CYBR1_HUMAN | Cytochrome b reductase 1 | Q53TN4 | 0.43 | 0.57 | 0.86 | 0.62 | 0.04 |
| 33 | D2HDH_HUMAN | D-2-hydroxyglutarate dehydrogenase, mitochondrial | Q8N465 | 0.63 | 0.69 | 0.31 | 0.55 | 0.02 |
| 34 | DHB11_HUMAN | Estradiol 17-beta-dehydrogenase 11 | Q8NBQ5 | 1.27 | 1.51 | 1.39 | 1.39 | 0.01 |
| 35 | DLRB1_HUMAN | Dynein light chain roadblock-type 1 | Q9NP97 | 1.29 | 1.54 | 1.17 | 1.34 | 0.04 |
| 36 | DPYL3_HUMAN | Dihydropyrimidinase-related protein 3 | Q14195 | 1.39 | 1.14 | 1.16 | 1.23 | 0.05 |
| 37 | EF2_HUMAN | Elongation factor 2 | P13639 | 0.89 |  | 0.76 | 0.82 | 0.03 |
| 38 | ELOC_HUMAN | Transcription elongation factor B polypeptide 1 | Q15369 | 0.08 | 0.61 | 0.65 | 0.44 | 0.04 |
| 39 | ENDD1_HUMAN | Endonuclease domain-containing 1 protein | O94919 | 0.55 | 0.48 | 0.40 | 0.48 | 0.00 |
| 40 | ENOA_HUMAN | Alpha-enolase | P06733 | 0.78 |  | 0.79 | 0.78 | 0.00 |
| 41 | ENOPH_HUMAN | Enolase-phosphatase E1 | Q9UHY7 |  | 0.66 | 0.80 | 0.73 | 0.01 |
| 42 | ENPL_HUMAN | Endoplasmin | P14625 | 0.70 |  | 0.87 | 0.79 | 0.04 |
| 43 | EST2_HUMAN | Cocaine esterase | O00748 | 0.45 | 0.60 | 0.82 | 0.63 | 0.03 |
| 44 | FBLN3_HUMAN | EGF-containing fibulin-like extracellular matrix protein 1 | Q12805 | 1.19 | 1.38 | 1.11 | 1.23 | 0.05 |
| 45 | FGF1_HUMAN | Fibroblast growth factor 1 | P05230 |  | 0.52 | 0.64 | 0.58 | 0.00 |
| 46 | FLII_HUMAN | Protein flightless-1 homolog | Q13045 | 0.82 |  | 0.67 | 0.74 | 0.02 |
| 47 | GBG7_HUMAN | Guanine nucleotide-binding protein G(I)/G(S)/G(O) subunit gamma-7 | O60262 | 0.63 | 0.77 | 0.90 | 0.77 | 0.04 |
| 48 | GLGB_HUMAN | 1,4-alpha-glucan-branching enzyme | Q04446 |  | 1.84 | 1.34 | 1.59 | 0.05 |
| 49 | GLPC_HUMAN | Glycophorin-C | P04921 |  | 1.34 | 1.74 | 1.54 | 0.03 |
| 50 | HAP28_HUMAN | 28 kDa heat- and acid-stable phosphoprotein | Q13442 | 0.42 | 0.52 | 0.85 | 0.60 | 0.04 |
| 51 | HCD2_HUMAN | 3-hydroxyacyl-CoA dehydrogenase type-2 | Q99714 | 1.94 |  | 1.70 | 1.82 | 0.00 |
| 52 | HHATL_HUMAN | Protein-cysteine N-palmitoyltransferase HHAT-like protein | Q9HCP6 | 0.82 | 0.79 |  | 0.81 | 0.00 |
| 53 | HNRH1_HUMAN | Heterogeneous nuclear ribonucleoprotein H | P31943 |  | 0.74 | 0.84 | 0.79 | 0.01 |
| 54 | HNRPQ_HUMAN | Heterogeneous nuclear ribonucleoprotein Q | O60506 | 1.85 | 1.67 | 1.29 | 1.61 | 0.02 |
| 55 | HSPB1_HUMAN | Heat shock protein beta-1 | P04792 | 1.31 | 1.38 | 1.14 | 1.27 | 0.02 |
| 56 | HV303_HUMAN | Ig heavy chain V-III region VH26 | P01764 | 0.78 | 0.84 | 0.83 | 0.82 | 0.00 |
| 57 | IGBP1_HUMAN | Immunoglobulin-binding protein 1 | P78318 | 0.75 | 0.88 | 0.84 | 0.82 | 0.01 |
| 58 | ISCA1_HUMAN | Iron-sulfur cluster assembly 1 homolog, mitochondrial | Q9BUE6 | 1.37 | 1.36 | 1.47 | 1.40 | 0.00 |
| 59 | IST1_HUMAN | IST1 homolog | P53990 | 0.60 | 0.61 | 0.87 | 0.69 | 0.03 |
| 60 | K1C10_HUMAN | Keratin, type I cytoskeletal 10 | P13645 | 1.51 | 1.33 | 1.20 | 1.35 | 0.02 |
| 61 | K1C9_HUMAN | Keratin, type I cytoskeletal 9 | P35527 | 0.79 | 0.68 | 0.72 | 0.73 | 0.00 |
| 62 | KAD1_HUMAN | Adenylate kinase isoenzyme 1 | P00568 | 0.47 |  | 0.76 | 0.61 | 0.04 |
| 63 | LETM1_HUMAN | LETM1 and EF-hand domain-containing protein 1, mitochondrial | O95202 | 0.56 |  | 0.63 | 0.60 | 0.00 |
| 64 | LIS1_HUMAN | Platelet-activating factor acetylhydrolase IB subunit alpha | P43034 | 0.61 |  | 0.54 | 0.57 | 0.00 |
| 65 | LKHA4_HUMAN | Leukotriene A-4 hydrolase | P09960 |  | 0.83 | 0.82 | 0.82 | 0.00 |
| 66 | LRC47_HUMAN | Leucine-rich repeat-containing protein 47 | Q8N1G4 | 1.29 | 1.19 | 1.16 | 1.22 | 0.01 |
| 67 | LV105_HUMAN | Ig lambda chain V-I region NEWM | P01703 | 0.75 | 0.87 |  | 0.81 | 0.02 |
| 68 | MCCB_HUMAN | Methylcrotonoyl-CoA carboxylase beta chain, mitochondrial | Q9HCC0 |  | 1.29 | 1.17 | 1.23 | 0.02 |
| 69 | MIA40_HUMAN | Mitochondrial intermembrane space import and assembly protein 40 | Q8N4Q1 | 0.57 | 0.76 | 0.84 | 0.72 | 0.03 |
| 70 | MK14_HUMAN | Mitogen-activated protein kinase 14 | Q16539 | 0.84 | 0.77 | 0.80 | 0.80 | 0.00 |
| 71 | MMAB_HUMAN | Cob(I)yrinic acid a,c-diamide adenosyltransferase, mitochondrial | Q96EY8 | 1.24 | 1.26 | 1.16 | 1.22 | 0.00 |
| 72 | MTCH1_HUMAN | Mitochondrial carrier homolog 1 | Q9NZJ7 | 1.15 |  | 1.27 | 1.21 | 0.02 |
| 73 | MTPN_HUMAN | Myotrophin | P58546 | 0.82 | 0.69 | 0.78 | 0.76 | 0.00 |
| 74 | MVP_HUMAN | Major vault protein | Q14764 |  | 0.73 | 0.86 | 0.80 | 0.03 |
| 75 | MYH14_HUMAN | Myosin-14 | Q7Z406 | 0.64 | 0.91 | 0.76 | 0.77 | 0.04 |
| 76 | MYH7B_HUMAN | Myosin-7B | A7E2Y1 | 0.86 | 0.52 | 0.73 | 0.70 | 0.04 |
| 77 | MYLK_HUMAN | Myosin light chain kinase, smooth muscle | Q15746 | 0.65 | 0.74 | 0.87 | 0.75 | 0.02 |
| 78 | NAC1_HUMAN | Sodium/calcium exchanger 1 | P32418 |  | 0.75 | 0.86 | 0.81 | 0.02 |
| 79 | NLTP_HUMAN | Non-specific lipid-transfer protein | P22307 | 1.21 | 1.64 | 1.41 | 1.42 | 0.03 |
| 80 | NRP1_HUMAN | Neuropilin-1 | O14786 | 0.72 |  | 0.88 | 0.80 | 0.05 |
| 81 | OPA1_HUMAN | Dynamin-like 120 kDa protein, mitochondrial | O60313 | 1.47 |  | 1.52 | 1.50 | 0.00 |
| 82 | PAK4_HUMAN | Serine/threonine-protein kinase PAK 4 | O96013 | 0.15 | 0.64 | 0.59 | 0.46 | 0.03 |
| 83 | PDIA4_HUMAN | Protein disulfide-isomerase A4 | P13667 | 1.24 | 1.21 | 1.17 | 1.21 | 0.00 |
| 84 | PFD6_HUMAN | Prefoldin subunit 6 | O15212 | 0.83 | 0.92 | 0.73 | 0.83 | 0.03 |
| 85 | PGK1_HUMAN | Phosphoglycerate kinase 1 | P00558 | 1.53 |  | 1.80 | 1.66 | 0.01 |
| 86 | PGRP2_HUMAN | N-acetylmuramoyl-L-alanine amidase | Q96PD5 | 0.67 | 0.75 | 0.67 | 0.70 | 0.00 |
| 87 | PLIN4_HUMAN | Perilipin-4 | Q96Q06 | 1.26 |  | 1.25 | 1.25 | 0.00 |
| 88 | PLST_HUMAN | Plastin-3 | P13797 | 1.28 | 1.33 | 1.29 | 1.30 | 0.00 |
| 89 | PP2BB_HUMAN | Serine/threonine-protein phosphatase 2B catalytic subunit beta isoform | P16298 | 0.44 |  | 0.44 | 0.44 | 0.00 |
| 90 | PPIA_HUMAN | Peptidyl-prolyl cis-trans isomerase A | P62937 | 0.69 |  | 0.86 | 0.78 | 0.04 |
| 91 | PSD13_HUMAN | 26S proteasome non-ATPase regulatory subunit 13 | Q9UNM6 | 0.72 | 0.72 | 0.91 | 0.78 | 0.03 |
| 92 | PTRD1_HUMAN | Putative peptidyl-tRNA hydrolase PTRHD1 | Q6GMV3 | 0.26 | 0.53 | 0.68 | 0.49 | 0.01 |
| 93 | RAC1_HUMAN | Ras-related C3 botulinum toxin substrate 1 | P63000 | 1.34 | 1.39 | 1.09 | 1.28 | 0.04 |
| 94 | RCN1_HUMAN | Reticulocalbin-1 | Q15293 | 0.49 | 0.62 | 0.81 | 0.64 | 0.02 |
| 95 | RL23_HUMAN | 60S ribosomal protein L23 | P62829 | 1.58 | 1.46 | 1.25 | 1.43 | 0.01 |
| 96 | RL27A_HUMAN | 60S ribosomal protein L27a | P46776 | 0.64 | 0.65 | 0.63 | 0.64 | 0.00 |
| 97 | RL35A_HUMAN | 60S ribosomal protein L35a | P18077 | 0.68 | 0.79 | 0.62 | 0.70 | 0.00 |
| 98 | RL5_HUMAN | 60S ribosomal protein L5 | P46777 | 1.09 | 1.38 | 1.37 | 1.28 | 0.04 |
| 99 | RMD3_HUMAN | Regulator of microtubule dynamics protein 3 | Q96TC7 | 0.91 | 0.69 | 0.75 | 0.78 | 0.03 |
| 100 | RRAS2_HUMAN | Ras-related protein R-Ras2 | P62070 | 0.74 | 0.62 | 0.92 | 0.76 | 0.05 |
| 101 | RS23_HUMAN | 40S ribosomal protein S23 | P62266 | 0.54 | 0.74 | 0.80 | 0.69 | 0.02 |
| 102 | RT30_HUMAN | 28S ribosomal protein S30, mitochondrial | Q9NP92 | 0.70 | 0.55 |  | 0.62 | 0.01 |
| 103 | S10A1_HUMAN | Protein S100-A1 | P23297 | 0.86 | 0.78 | 0.70 | 0.78 | 0.01 |
| 104 | S27A1_HUMAN | Long-chain fatty acid transport protein 1 | Q6PCB7 | 1.11 | 1.53 | 1.42 | 1.35 | 0.05 |
| 105 | SAR1B_HUMAN | GTP-binding protein SAR1b | Q9Y6B6 | 1.21 | 1.14 | 1.34 | 1.23 | 0.02 |
| 106 | SCAM3_HUMAN | Secretory carrier-associated membrane protein 3 | O14828 |  | 0.69 | 0.66 | 0.67 | 0.00 |
| 107 | SIAS_HUMAN | Sialic acid synthase | Q9NR45 | 1.34 |  | 1.59 | 1.46 | 0.01 |
| 108 | SNX3_HUMAN | Sorting nexin-3 | O60493 | 0.81 | 0.75 | 0.79 | 0.78 | 0.00 |
| 109 | SODC_HUMAN | Superoxide dismutase [Cu-Zn] | P00441 |  | 1.38 | 1.76 | 1.57 | 0.03 |
| 110 | TALDO_HUMAN | Transaldolase | P37837 |  | 0.81 | 0.73 | 0.77 | 0.00 |
| 111 | TBB4B_HUMAN | Tubulin beta-4B chain | P68371 | 0.79 |  | 0.74 | 0.76 | 0.00 |
| 112 | TCPH_HUMAN | T-complex protein 1 subunit eta | Q99832 | 0.76 | 0.69 |  | 0.73 | 0.00 |
| 113 | TCPZ_HUMAN | T-complex protein 1 subunit zeta | P40227 | 0.86 | 0.75 |  | 0.81 | 0.02 |
| 114 | TGM2_HUMAN | Protein-glutamine gamma-glutamyltransferase 2 | P21980 | 1.27 |  | 1.13 | 1.20 | 0.03 |
| 115 | THTR_HUMAN | Thiosulfate sulfurtransferase | Q16762 | 0.81 |  | 0.56 | 0.69 | 0.04 |
| 116 | TIM14_HUMAN | Mitochondrial import inner membrane translocase subunit TIM14 | Q96DA6 | 0.75 | 0.59 | 0.53 | 0.62 | 0.00 |
| 117 | TIM8A_HUMAN | Mitochondrial import inner membrane translocase subunit Tim8 A | O60220 | 0.41 | 0.83 | 0.67 | 0.64 | 0.04 |
| 118 | UB2L3_HUMAN | Ubiquitin-conjugating enzyme E2 L3 | P68036 | 0.69 | 0.86 |  | 0.77 | 0.04 |
| 119 | UBP5_HUMAN | Ubiquitin carboxyl-terminal hydrolase 5 | P45974 | 1.45 |  | 1.31 | 1.38 | 0.01 |
| 120 | UCHL3_HUMAN | Ubiquitin carboxyl-terminal hydrolase isozyme L3 | P15374 | 1.58 | 1.27 | 1.35 | 1.40 | 0.01 |
| 121 | UFD1_HUMAN | Ubiquitin fusion degradation protein 1 homolog | Q92890 | 0.69 | 0.86 | 0.71 | 0.75 | 0.01 |
| 122 | VIGLN_HUMAN | Vigilin | Q00341 | 0.61 | 0.90 | 0.66 | 0.72 | 0.03 |
| 123 | XIRP1_HUMAN | Xin actin-binding repeat-containing protein 1 | Q702N8 | 0.72 |  | 0.79 | 0.75 | 0.00 |
| 124 | YBOX3_HUMAN | Y-box-binding protein 3 | P16989 | 0.79 | 0.86 | 0.79 | 0.81 | 0.00 |
| 125 | ZYX_HUMAN | Zyxin | Q15942 | 1.41 | 1.64 |  | 1.53 | 0.01 |
