# Supplementary material for: In-depth proteomic profiling of left ventricular tissues in human end-stage dilated cardiomyopathy
Source: Oncotarget. 2017 Feb 25;8(29):48321–32. doi: 10.18632/oncotarget.15689 (PMC5564650; doi:10.18632/oncotarget.15689)
Supplement: Supplementary file 4 [file oncotarget-08-48321-s004.docx]

**Table S3. 41 predicted secretome list and 10 of which have been reported in plasma/serum by literature retrieval**

| **Number** | **ID** | **Accession** | **Protein names** | **NN-score** | **Papers** |
| --- | --- | --- | --- | --- | --- |
| 1 | GBG7_HUMAN | O60262 | Guanine nucleotide-binding protein G(I)/G(S)/G(O) subunit gamma-7 | 0.609 |  |
| 2 | KAD1_HUMAN | P00568 | Adenylate kinase isoenzyme 1 | 0.674 | MAPRE1 as a Plasma Biomarker for Early-Stage Colorectal Cancer and Adenomas;;;Blood metal levels and related antioxidant enzyme activities in patients with ataxia telangiectasia |
| 3 | HV323_HUMAN | P01764 | Immunoglobulin heavy variable 3-23 | 0.839 |  |
| 4 | CRYAB_HUMAN | P02511 | Alpha-crystallin B chain | 0.86 |  |
| 5 | APOC1_HUMAN | P02654 | Apolipoprotein C-I | 0.864 | Discovery of serum biomarkers implicated in the onset and progression of serous ovarian cancer in a rat model using iTRAQ technique |
| 6 | GLPC_HUMAN | P04921 | Glycophorin-C | 0.945 | Anti-Ge2: further evidence for lack of clinical significance. |
| 7 | FGF1_HUMAN | P05230 | Fibroblast growth factor 1 | 0.666 |  |
| 8 | CD36_HUMAN | P16671 | Platelet glycoprotein 4 | 0.847 | Soluble CD36 in plasma and urine: A plausible prognostic marker for diabetic nephropathy |
| 9 | YBOX3_HUMAN | P16989 | Y-box-binding protein 3 | 0.803 |  |
| 10 | RL35A_HUMAN | P18077 | 60S ribosomal protein L35a | 0.615 |  |
| 11 | S10A1_HUMAN | P23297 | Protein S100-A1 | 0.822 |  |
| 12 | NAC1_HUMAN | P32418 | Sodium/calcium exchanger 1 | 0.761 |  |
| 13 | RL27A_HUMAN | P46776 | 60S ribosomal protein L27a | 0.614 |  |
| 14 | CAV2_HUMAN | P51636 | Caveolin-2 | 0.686 |  |
| 15 | CA2D1_HUMAN | P54289 | Voltage-dependent calcium channel subunit alpha-2/delta-1 | 0.73 |  |
| 16 | BID_HUMAN | P55957 | BH3-interacting domain death agonist | 0.705 |  |
| 17 | RRAS2_HUMAN | P62070 | Ras-related protein R-Ras2 | 0.824 |  |
| 18 | RL23_HUMAN | P62829 | 60S ribosomal protein L23 | 0.85 |  |
| 19 | COASY_HUMAN | Q13057 | Bifunctional coenzyme A synthase | 0.825 |  |
| 20 | RCN1_HUMAN | Q15293 | Reticulocalbin-1 | 0.773 |  |
| 21 | CNN3_HUMAN | Q15417 | Calponin-3 | 0.851 |  |
| 22 | THTR_HUMAN | Q16762 | Thiosulfate sulfurtransferase | 0.643 |  |
| 23 | PTRD1_HUMAN | Q6GMV3 | Putative peptidyl-tRNA hydrolase | 0.616 | Effects of Intravenous Administration of Taurocholate on Liver and Serum Thiosulfate Sulfurtransferase Activities in Cholestatic Rat |
| 24 | DHB11_HUMAN | Q8NBQ5 | Estradiol 17-beta-dehydrogenase 11 | 0.75 |  |
| 25 | UFD1_HUMAN | Q92890 | Ubiquitin fusion degradation protein 1 homolog | 0.649 |  |
| 26 | PGRP2_HUMAN | Q96PD5 | N-acetylmuramoyl-L-alanine amidase | 0.664 | Host Protein Biomarkers Identify Active Tuberculosis in HIV Uninfected and Co-infected Individuals |
| 27 | ISCA1_HUMAN | Q9BUE6 | Iron-sulfur cluster assembly 1 homolog | 0.655 |  |
| 28 | HHATL_HUMAN | Q9HCP6 | Protein-cysteine N-palmitoyltransferase HHAT-like protein | 0.747 |  |
| 29 | RT30_HUMAN | Q9NP92 | 28S ribosomal protein S30 | 0.688 |  |
| 30 | DLRB1_HUMAN | Q9NP97 | Dynein light chain roadblock-type 1 | 0.694 |  |
| 31 | SIAS_HUMAN | Q9NR45 | Sialic acid synthase | 0.925 |  |
| 32 | ENOPH_HUMAN | Q9UHY7 | Enolase-phosphatase E1 | 0.602 |  |
| 33 | SODC_HUMAN | P00441 | Superoxide dismutase [Cu-Zn] | 0.699 | MAPRE1 as a Plasma Biomarker for Early-Stage Colorectal Cancer and Adenomas;;;Blood metal levels and related antioxidant enzyme activities in patients with ataxia telangiectasia |
| 34 | HSPB1_HUMAN | P04792 | Heat shock protein beta-1 | 0.648 | COPD and levels of Hsp70 (HSPA1A) and Hsp27 (HSPB1) in plasma and lymphocytes among coal workers: a case-control study |
| 35 | FBLN3_HUMAN | Q12805 | EGF-containing fibulin-like extracellular matrix protein 1 | 0.74 | Fibulin-3,serum, obese women |
| 36 | S27A1_HUMAN | Q6PCB7 | Long-chain fatty acid transport protein 1 | 0.719 |  |
| 37 | LRC47_HUMAN | Q8N1G4 | Leucine-rich repeat-containing protein 47 | 0.737 |  |
| 38 | ATAD1_HUMAN | Q8NBU5 | ATPase family AAA domain-containing protein 1 | 0.829 |  |
| 39 | MMAB_HUMAN | Q96EY8 | Cob(I)yrinic acid a,c-diamide adenosyltransferase | 0.611 |  |
| 40 | HCD2_HUMAN | Q99714 | 3-hydroxyacyl-CoA dehydrogenase type-2 | 0.844 |  |
| 41 | MTCH1_HUMAN | Q9NZJ7 | Mitochondrial carrier homolog 1 | 0.665 | Mitochondrial carrier homolog 1 (Mtch1) antibodies in neuro-Behcet's disease |
