# Supplementary material for: In-depth proteomic profiling of left ventricular tissues in human end-stage dilated cardiomyopathy
Source: Oncotarget. 2017 Feb 25;8(29):48321–32. doi: 10.18632/oncotarget.15689 (PMC5564650; doi:10.18632/oncotarget.15689)
Supplement: Supplementary file 5 [file oncotarget-08-48321-s005.docx]

**Table S4. The rough study status of 20 enriched proteins in cellular assembly and organization network and 19 enriched proteins in cell cycle network in main cardiovascular disease MI, I/R, As, CS and DCM.**

**9 of 20 proteins in "cellular assembly and organization" network have been studied in I/R, As, MI, CS or DCM disease by literature retrieval**

| **Number** | **ID** | **Gene** | **I/R** | **As** | **MI** | **CS** | **DCM** |
| --- | --- | --- | --- | --- | --- | --- | --- |
| 1 | CALD1_HUMAN | CALD1 | The Effect of Ischemia/Reperfusion on Rabbit Bladder-Role of Rho-kinase and Smooth Muscle Regulatory Proteins | Caldesmon as a Therapeutic Target for Proliferative Vascular Diseases |  |  |  |
| 2 | CAV2_HUMAN | CAV2 | Intralipid protects the heart in late pregnancy against ischemia/reperfusion injury via Caveolin2/STAT3/GSK-3beta pathway. |  | Late gadolinium enhancement assessed by cardiac magnetic resonance imaging in heart transplant recipients with different stages of cardiac allograft vasculopathy |  |  |
| 3 | CLIC5_HUMAN | CLIC5 | Molecular identity of cardiac mitochondrial chloride intracellular channel proteins |  |  |  |  |
| 4 | MTPN_HUMAN | MTPN |  |  |  | Myotrophin is a more powerful predictor of major adverse cardiac events following acute coronary syndrome than N-terminal pro-B-type natriuretic peptide |  |
| 5 | MVP_HUMAN | MVP |  | Major Vault Protein Regulates Class A Scavenger Receptor-mediated Tumor Necrosis Factor-alpha Synthesis and Apoptosis in Macrophages |  |  |  |
| 6 | PAK4_HUMAN | PAK4 |  |  | MicroRNA-24 Regulates Vascularity After Myocardial Infarction |  |  |
| 7 | PPIA_HUMAN | PPIA | Metabolic Adaptation to a Disruption in Oxygen Supply during Myocardial Ischemia and Reperfusion Is Underpinned by Temporal and Quantitative Changes in the Cardiac Proteome | Cyclophilin A is an inflammatory mediator that promotes atherosclerosis in apolipoprotein E-deficient mice | A search for cyclophilin-A gene (PPIA) variation and its contribution to the risk of atherosclerosis and myocardial infarction | PPIA rs6850: A > G single-nucleotide polymorphism is associated with raised plasma cyclophilin A levels in patients with coronary artery disease |  |
| 8 | S10A1_HUMAN | S100A1 | Translocation of S100A1 calcium binding protein during heart surgery |  | S100A1, a new marker for acute myocardial ischemia |  | Targeting S100A1 in heart failure |
| 9 | ZYX_HUMAN | ZYX |  | Aorta protein networks in marginal and acute zinc deficiency | Quantitative proteomics reveals differential regulation of protein expression in recipient myocardium after trilineage cardiovascular cell transplantation | Oligonucleotide Microarray and QRT-PCR Study of Adhesion Protein Gene Expression in Acute Coronary Syndrome Patients | Mutations in the muscle LIM protein and alpha-actinin-2 genes in dilated cardiomyopathy and endocardial fibroelastosis |

**6 of 19 proteins in cell cycle network have been studied in I/R, As, MI, CS or DCM disease by literature retrieval**

| **Number** | **ID** | **Gene** | **I/R** | **As** | **MI** | **CS** | **DCM** |
| --- | --- | --- | --- | --- | --- | --- | --- |
| 1 | ABCD3_HUMAN | ABCD3 | ATP-binding cassette transporters in immortalised human brain microvascular endothelial cells in normal and hypoxic conditions. |  |  |  |  |
| 2 | CRYAB_HUMAN | CRYAB | alpha B-Crystallin Improves Murine Cardiac Function and Attenuates Apoptosis in Human Endothelial Cells Exposed to Ischemia-Reperfusion |  | Cardioprotective Role of P38 MAPK During Myocardial Infarction Via Parallel Activation of alpha-Crystallin B and Nrf2 |  |  |
| 3 | RL27A_HUMAN | RPL27A |  | Gene expression profiles of U937 human macrophages exposed to Chlamydophila pneumoniae and/or low density lipoprotein in five study models using differential display and real-time RT-PCR |  |  |  |
| 4 | CD9_HUMAN | CD9 | Hepatoprotective effect of exosomes from human-induced pluripotent stem cell-derived mesenchymal stromal cells against hepatic ischemia-reperfusion injury in rats | Platelet phagocytosis and processing of beta-amyloid precursor protein as a mechanism of macrophage activation in atherosclerosis |  | Crossreactivity of human versus swine platelet surface antigens is similar for glycoproteins Ib and IIIa, but not for the glycoprotein IIb/IIIa complex |  |
| 5 | NRP1_HUMAN | NRP1 | Transient and bilateral increase in Neuropilin-1, Fer kinase and collapsin response mediator proteins within membrane rafts following unilateral occlusion of the middle cerebral artery in mouse | he expanding role of neuropilin: regulation of transforming growth factor- and platelet-derived growth factor signaling in the vasculature |  | Hand2 Is an Essential Regulator for Two Notch-Dependent Functions within the Embryonic Endocardium |  |
| 6 | EF2_HUMAN | eEF2 | Proteins regulating cap-dependent translation are downregulated during total knee arthroplasty | Proteomics analysis of human umbilical vein endothelial cells treated with resveratrol | AKIP1, a Cardiac Hypertrophy Induced Protein that Stimulates Cardiomyocyte Growth via the Akt Pathway |  |  |
