# Supplementary material for: In-depth proteomic profiling of left ventricular tissues in human end-stage dilated cardiomyopathy
Source: Oncotarget. 2017 Feb 25;8(29):48321–32. doi: 10.18632/oncotarget.15689 (PMC5564650; doi:10.18632/oncotarget.15689)
Supplement: Supplementary file 6 [file oncotarget-08-48321-s006.docx]

**Supplementary Table S5** The transition peak areas and ratio (DCM/Control) from a S100A1 peptide and two unique eEF2 peptides in every sample.

| Protein name | Peptide sequence | Transition | Peak area | | Ratio | Peak area | | Ratio | Peak area | | Ratio | CV |
| --- | --- | --- | --- | --- | --- | --- | --- | --- | --- | --- | --- | --- |
|  |  |  | DCM1 | N1 | DCM1/N1 | DCM2 | N2 | DCM2/N2 | DCM3 | N3 | DCM3/N3 |  |
| S100A1 | ELLQTELSGFLDAQK | y8 | 469758 | 708982 | 0.66 | 411526 | 503469 | 0.82 | 627261 | 766104 | 0.82 | 21.45% |
|  |  | y7 | 376226 | 671762 | 0.56 | 313106 | 425561 | 0.74 | 525041 | 504320 | 1.04 |  |
|  |  | y5 | 126672 | 131354 | 0.96 | 99676 | 149004 | 1.25 | 195989 | 157050 | 0.67 |  |
|  |  | y4 | 191581 | 225883 | 0.85 | 137321 | 230773 | 0.60 | 290505 | 224824 | 1.29 |  |
| eEF2 | GGGQIIPTAR | y6 | 86181 | 138101 | 0.62 | 33222 | 118625 | 0.28 | 79682 | 192887 | 0.41 | 6.27% |
|  |  | y5 | 190292 | 256756 | 0.74 | 74761 | 295452 | 0.25 | 183014 | 287165 | 0.64 |  |
|  |  | y4 | 315468 | 428682 | 0.74 | 157827 | 430304 | 0.37 | 352843 | 580487 | 0.61 |  |
|  |  | b4 | 80471 | 92150 | 0.87 | 65925 | 131613 | 0.50 | 78384 | 107723 | 0.73 |  |
|  |  | b5 | 71636 | 84883 | 0.84 | 35039 | 60216 | 0.58 | 42566 | 78643 | 0.54 |  |
|  |  | b6 | 14016 | 18428 | 0.76 | 13756 | 25696 | 0.54 | 19726 | 18168 | 1.09 |  |
|  | GVQYLNEIK | y7 | 19726 | 70609 | 0.28 | 7787 | 19985 | 0.39 | 23879 | 60226 | 0.40 | 31.22% |
|  |  | y6 | 50872 | 259915 | 0.20 | 23879 | 60226 | 0.40 | 85663 | 240166 | 0.36 |  |
|  |  | y4 | 31405 | 101236 | 0.31 | 40490 | 40749 | 0.99 | 50613 | 141216 | 0.36 |  |

CV represents variation from three group of samples.
